# A Simulation and Small-Group Pediatric Emergency Medicine Course for Generalist Healthcare Providers: Gastrointestinal and Nutrition Emergencies

**DOI:** 10.21980/J8WH2K

**Published:** 2024-10-31

**Authors:** Adeola Adekunbi Kosoko, Alicia E Genisca, Nicholas A Peoples, Connor Tompkins, Ryan Sorensen, Joy Mackey

**Affiliations:** * McGovern Medical School at the University of Texas Health Science Center at Houston, Department of Emergency Medicine, Houston, TX; ^ The Warren Alpert Medical School of Medicine Brown University/Hasbro Children’s Hospital, Departments of Emergency Medicine and Pediatrics, Providence, RI; † The Baylor College of Medicine, Houston, TX; ** Henry J.N. Taub Baylor College of Medicine, Department of Emergency Medicine, Houston TX

## Abstract

**Audience and Type of Curriculum:**

This is a review curriculum utilizing multiple methods of education to enhance the skills of generalist healthcare providers in low- and middle-income countries (LMICs) in the identification and stabilization of pediatric respiratory emergencies. Our audience of implementation was Belizean generalist providers (nurses and physicians).

**Length of Curriculum:**

8–10 hours

**Introduction:**

Early recognition and stabilization of critical pediatric patients can improve outcomes. Compared with resource-rich systems, many low-resource settings (i.e., LMICs) rely on generalists to provide most pediatric acute care. We created a curriculum for general practitioners comprising multiple educational modules focused on identifying and stabilizing pediatric emergencies. Our aim was to develop an educational framework to update and teach generalists on the recommendations and techniques of optimally evaluating and managing pediatric nutritional and gastrointestinal emergencies: bowel obstructions, gastroenteritis, and malnutrition.

**Educational Goals:**

The aim of this curriculum is to increase learners’ proficiency in identifying and stabilizing acutely ill pediatric patients with gastrointestinal medical or surgical disease or complications of malnutrition. This module focuses on the diagnosis and management of gastroenteritis, acute bowel obstruction, and deficiencies of feeding and nutrition. The target audience for this curriculum is generalist physicians and nurses in limited-resource settings.

**Educational Methods:**

The educational strategies used in this curriculum include didactic lectures, medical simulation, and small-group sessions.

**Research Methods:**

We evaluated written pretests before and posttests after intervention and retested participants four months later to evaluate for knowledge retention. Participants provided qualitative feedback on the module.

**Results:**

We taught 21 providers. Eleven providers completed the pretest/posttest and eight completed the retest. The mean test scores improved from 8.3 ± 1.7 in the pretest to 12.2 ± 2.6 in the posttest (mean difference: 1.4, *P*=0.027). The mean test score at pretest was 8.3 ± 2.3, which increased to 10.8 ± 3.0 at retest (mean difference: 2.5, *P*=0.060). Seven (71.4%) and four (28.5%) participants found the course “extremely useful” and “very useful,” respectively (n=11).

**Discussion:**

This curriculum may be an effective and welcome training tool for Belizean generalist providers. There was a statistically significant improvement in the test performance but not in retesting, possibly due to our small sample size and high attrition rate. Evaluation of other modules in this curriculum, application of this curriculum in other locations, and measuring clinical practice interventions will be included in future investigations.

**Topics:**

Medical simulation, rapid cycle deliberate practice (RCDP), Belize, gastrointestinal, nutrition, emergency, gastroenteritis, acute bowel obstruction, Belize, low- and middle-income country (LMIC), collaboration, global health.

## USER GUIDE

**Table:** 

List of Resources:	
Abstract	1
User Guide	3
Appendix A: Curriculum Chart	9
Appendix B: Example Schedule	12
Appendix C: Pretest Questions	13
Appendix D: Pretest Answers	18
Appendix E: Diarrhea and Dehydration Lecture Synopsis	23
Appendix F: Diarrhea and Dehydration Lecture	33
Appendix G: Diarrhea and Dehydration Simulation Case	34
Appendix H: Small Bowel Obstruction Lecture Synopsis	53
Appendix I: Small Bowel Obstruction Lecture	65
Appendix J: Small Bowel Obstruction Simulation Case	66
Appendix K: Malnutrition Lecture Synopsis	89
Appendix L: Malnutrition Lecture	98
Appendix M: Malnutrition Small Group Discussion	99
Appendix N: Posttest Questions	111
Appendix O: Posttest Answers	116

**Learner Audience:**

Medical Students, Interns, Junior Residents, Senior Residents, General Practitioners (physicians, nurses)

**Length of Curriculum:**

The entire course was designed to be presented over about eight to ten hours total. It could be completed in a day, but we divided the course into two days.

- There are two simulation sessions, each lasting about 45 minutes.
- There is one small group session, with about 45 minutes of discussion.
- There are three didactic lectures, each lasting about one hour each.
- Most participants used about 20–30 minutes to complete each of the written tests.

**Topics:**

Medical simulation, rapid cycle deliberate practice (RCDP), Belize, gastrointestinal, nutrition, emergency, gastroenteritis, acute bowel obstruction, Belize, low- and middle-income country (LMIC), collaboration, global health.

**Objectives:**

By the end of this course, learners will:

- Rapidly assess and initiate emergency interventions for a child with acute diarrhea and dehydration.
- Become more familiar with medical and surgical gastrointestinal and nutrition pathophysiology including interventions unique to pediatric populations.
- Identify diagnostic criteria for gastroenteritis, various types of bowel obstruction, and malnutrition in a pediatric patient.
- Improve communication and teamwork when managing an acutely ill pediatric patient.

Diarrhea and Dehydration Lecture and Simulation Objectives:

- Identify viral causes and bacterial causes of gastroenteritis
- Classify the degree of dehydration in the child presenting with diarrhea
- Identify a well-nourished child from a malnourished child in order to determine best management of dehydration
- Outline World Health Organization Plans A, B, and C for treatment of dehydration
- Choose the appropriate treatment strategy for viral and bacterial gastroenteritis
- Anticipate complications of gastroenteritis

Bowel Obstruction Lecture and Simulation Objectives:

- Evaluate the child presenting with vomiting and abdominal pain
- Give a differential diagnosis for acute bowel obstruction in a child
- Determine if a child has a dangerous cause of vomiting
- Medically manage a child with acute bowel obstruction
- Order appropriate imaging and refer children requiring further management

Feeding and Nutrition Lecture and Small Group Objectives

- Identify malnutrition and its forms
- Identify malnutrition emergencies
- Initiate treatment for malnutrition emergencies

### Brief introduction

An estimated 80% of deaths in children below five years of age in limited-resource settings, particularly low- and middle-income countries (LMICs), are avoidable.^1^ Practitioners in many LMICs often lack targeted training for the care of acutely ill children.^2^,^3^ Most physicians in Belize are Belizean nationals who were trained abroad and returned home to practice, or they are immigrants (Fig. 1) to Belize, including volunteers from the Cuban brigade. Moreover, Belize, like many other LMICs, does not generally have national guidelines on most pediatric patient care topics. Thus, caregivers do not regularly subscribe to any consensus of patient care for any given topic. Instead, individual experience, local patterns, guidelines set by other countries, and occasionally international guidelines tend to form the basis of patterns of care (eg, World Health Organization Integrated Management of Childhood Illness guidelines vs American Academy of Pediatric guidelines or other international groups). Interventions including triage concepts, specific emergency care courses, and the use of clinical practice guidelines have been suggested to improve emergency outcomes and provide evidence-based patient care. Other studies have shown that targeted multidisciplinary and multicultural team training can be effective in stressful situations.^4^,^5^

This module is one of a multiple-part curriculum developed to expand the training of providers in LMICs on pediatric emergency medicine topics. We have successfully taught the first module of this curriculum, which refreshes learners on respiratory emergencies.^9^

In LMICs, generally, national epidemiological data regarding pediatric gastroenterological conditions are often limited to nutrition issues and diarrheal diseases.^10^ Currently, more than one in five children aged less than five worldwide are affected by growth stunting, and 7.5% of children are affected by wasting.^11^ Intestinal obstruction is one of the most common causes of acute abdomen surgical emergencies. Intussusception is the leading cause of pediatric bowel obstruction around the world, but other common forms of acquired bowel obstruction include incarcerated hernias and malrotation of the bowel with midgut volvulus and foreign bodies. Acute gastrointestinal (GI) illnesses and abdominal pain were in the top five pediatric complaints that presented for emergency care during the needs assessment our team conducted at the national government and referral hospital in Belize City, Karl Heusner Memorial Hospital Authority (KHMHA).

### Problem identification, general and targeted needs assessment

In 2015, we conducted a formal needs assessment of KHMHA which identified a desire for improved care for the acutely ill child. In collaboration with the KHMHA leadership, we focused on provider education and identified appropriate subject matter based on review of the Accident and Emergency (A&E) Department patient logbook, reviewing patient volumes and diagnoses, and by providing formal interviews and surveys with staff and administration to discover areas of strengths and weaknesses in pediatric emergency care. Stakeholders included physicians, nurses, and administrators representing the hospital as a whole, in addition to those representing the A&E department and the pediatrics department.

We decided to use an integrated approach to the curriculum that would incorporate both active (hands-on and interactive engagement with learning material) and passive learning (exposure to learning material). Though the local learners were more familiar with passive learning techniques (eg, reading and didactic lectures),^6^ simulation-based medical education (active learning) provides the opportunity to reproducibly practice high-risk scenarios in a safe learning environment, and active techniques are becoming more popular in medical education. Medical simulation exercises can improve clinical knowledge, procedural skills, confidence, teamwork, and effective communication. We chose rapid cycle deliberate practice (RCDP) as our medical simulation format, specifically chosen for this population because of its suitability for those less familiar with medical simulation and for those with the goal of attaining mastery of a topic or skill.^5^ RCDP is an instructional method for simulation-based learning that incorporates multiple shorter repetitions of cases with intermixed feedback. It has been useful in improving key performance measures.^6^ Small-group clinical cases (active learning) facilitate critical thinking rather than encouraging simple memorization, reveal the relevance to clinical practice of the material being taught, and integrate multiple concepts in one session.^7^,^8^ By revisiting previous content and demonstrating clinical connections in our approach, we wanted to enhance the learning experience.

This curriculum was designed based on the 2015 formal needs assessment of the KHMHA A&E and pediatrics departments and on core pediatric emergency competencies from the American Board of Emergency Medicine, the American Academy of Pediatrics, and the care recommendations made by the World Health Organization. The aim is that it can ultimately be applied in similarly sourced LMICs. It is intended to be an integrated curriculum regarding the target populations of nurses and physicians with various levels of training. We wanted to teach various health professionals to work collaboratively. This module includes two low-fidelity simulation teaching scenarios using RCDP (small bowel obstruction and gastroenteritis) and one small-group clinical discussion (malnutrition), and a pre- and post- multiple-choice written test. Due to the general lack of participant experience with simulation-based learning and the goal of providing timely feedback, we chose RCDP with opportunities to immediately apply feedback and debriefing for our given scenarios.^7^ The small-group exercises are intended to foster active learning and fill gaps in understanding potentially left by the didactic sessions. We felt the intricacies of identifying and resuscitating a malnourished child lends itself better for teaching small group learning rather than simulation. The written testing consisted of multiple-choice evaluations of the topics covered during the module. This study received institutional review board approval from UT Health and Baylor College of Medicine with approval of the KHMHA administration.

### Goals of the curriculum

The aim of this curriculum is to increase learners’ proficiency and understanding in identifying and stabilizing acutely ill pediatric patients. This module focuses on the diagnosis and management of pediatric diarrhea and dehydration, acute bowel obstruction, and issues of feeding and nutrition.

### Objectives of the curriculum

By the end of this course, learners will:

- Rapidly assess and initiate emergency interventions for a child with acute diarrhea and dehydration.
- Become more familiar with medical and surgical gastrointestinal and nutrition pathophysiology including interventions unique to pediatric populations.
- Identify diagnostic criteria for gastroenteritis, various types of bowel obstruction, and malnutrition in a pediatric patient.
- Improve communication and teamwork when managing an acutely ill pediatric patient.

Diarrhea and Dehydration Lecture and Simulation Objectives:

- Identify viral causes and bacterial causes of gastroenteritis
- Classify the degree of dehydration in the child presenting with diarrhea
- Identify a well-nourished child from a malnourished child in order to determine best management of dehydration
- Outline World Health Organization Plans A, B, and C for treatment of dehydration
- Choose the appropriate treatment strategy for viral and bacterial gastroenteritis
- Anticipate complications of gastroenteritis

Bowel Obstruction Lecture and Simulation Objectives:

- Evaluate the child presenting with vomiting and abdominal pain
- Give a differential diagnosis for acute bowel obstruction in a child
- Determine if a child has a dangerous cause of vomiting
- Medically manage a child with acute bowel obstruction
- Order appropriate imaging and refer children requiring further management

Feeding and Nutrition Lecture and Small Group Objectives

- Identify malnutrition and its forms
- Identify malnutrition emergencies
- Initiate treatment for malnutrition emergencies

### Educational Strategies

Please see the separate Curriculum Chart document of linked objectives and educational strategies.

### Equipment/Environment

The following were required to carry out the module:

- A large room (with a capacity of at least 50 people) with multiple tables and ample floor space, or multiple rooms if available
- A computer and projector setup
- For each group of three to five learners, one equipment setup includes the following:
  ○ A low-fidelity full-body simulation mannequin. If available, higher-fidelity mannequins can be used (we used MegaCode Kid, and Laerdal ALS Baby mannequins)
  ○ An intravenous arm task trainer (if the mannequin is not equipped) or equipment to practice IV placement amongst learners
  ○ Lower extremity capable of intraosseous (IO) insertion (if mannequin is not equipped)
  ○ A medical resuscitation setup including the standard resuscitation equipment available in the A&E department (intravenous line starter kits, intravenous fluids, IO drill, IO needles, medical tape, bag valve mask, mock medications, etc.)

### Personnel

Personnel needed are as follows:

- One simulation instructor/debriefing facilitator per every group of three to six learners.
- One actor/assistant per every group of three to six learners.

### Results and tips for successful implementation

#### Implementation

This module was conducted at KHMHA over two days (total of nine hours). On day one, multiple-choice pretesting (Appendices C, D), two didactic lectures (Appendices E, F, K, L), one simulation session (Appendix G), and a small group discussion (Appendix M) were carried out. The participants were divided into groups of three to six members depending on the number of facilitators and simulation materials. Each simulation scenario was repeated using RCDP for up to 45 minutes per session. The participants took turns acting out different roles within each scenario with facilitators. On day two, we completed the didactic lectures (Appendices H, I), the simulation session (Appendix J), and written multiple-choice posttests (Appendices N, O).

#### Assessment

Though there is opportunity for improvisation in the order of the didactic lectures, small group activity, and simulation activities, the module must begin with the written pretest assessment (Appendices C and D). An instructor should always present a didactic lecture (Appendices E, F, K, L, H, I) to the entire class before any group-based activities. After the lecture, the participants were assigned into groups of three to six people, ideally incorporating learners of differing backgrounds and experiences equally into each group in order to challenge and encourage communication and teamwork. Though there was not a formal process of determining the groups, we encouraged them to be heterogenous in their composition by considering their role in healthcare (nurses, physicians), their years of experience, those practicing in the A&E, and those practicing in the pediatrics department. Each group then performed the corresponding small-group clinical case discussion, or the simulation scenario as indicated (Appendices G, J, M). The RCDP format was used in each simulation session, wherein debriefing was integrated regularly into each performance round, guided by the critical actions of each case and the feedback cues (Table 2). Two topics (i.e., diarrhea and dehydration and feeding and nutrition) were presented on the first day, and the other topic (i.e., small bowel obstruction) was presented on the second day. After all topics were covered, a multiple-choice posttest was administered to evaluate the learners (Appendices N and O).

The participants were invited to provide anonymous feedback on the module itself and on the instructors. After four months and before the next iteration of this longitudinal course was offered, the providers who participated in this gastrointestinal module again completed the posttest (Appendices N and O) to evaluate knowledge retention after the intervention.

#### Quantitative Methods

A t-test was used to compare the pretest and posttest scores to measure the impact of the training sessions on the knowledge of the participants. To determine knowledge retention, a t-test was again used to compare the pretest and retest scores, the latter of which were obtained four months after the training module. The results of the hypothesis testing were considered statistically significant at *P*<0.05. Stata SE version 15.1 (StataCorp, College Station, TX, USA) was used for all statistical analyses.

#### Qualitative Methods

We used a feedback questionnaire of open-ended questions to elicit feedback from the participants regarding areas in which we could improve the training. Each entry was reviewed for words or phrases representing one main idea (open codes), and open codes that represent related ideas were categorized into main themes.

#### Debriefing

The simulation sessions are meant to be performed using RCDP format. This means that rather than a traditional longer debriefing session after the simulation is performed from beginning to end, these cases allow for fixed stops and for discussion of the case and learner feedback to be integrated into each performance round. The stops are guided by the critical actions of each case and feedback cues.

### Evaluation and Feedback

#### Demographic Characteristics

A total of 21 learners participated in this module of the course. Only 11 participants completed the testing and demographic information collection, including five women (45%) and six men (55%). The participants were ten physicians and one nurse. The participants were trained in several countries (Figure 1). The participants had been in practice for a mean of 11.4 years and a median of 7.5 years (range: 2–29 years). The participants were asked how comfortable they felt in the management of pediatric patient care in general prior to the intervention. On a scale from 1 (“extremely uncomfortable”) to 5 (“extremely comfortable”), the mean rating of the participants was 3.8 (range: 3–5).

#### Test Scores

The participants completed a scored test before (pretest) and after (posttest) the training to determine their baseline knowledge and the impact of the curriculum on their knowledge. A total of 11 providers completed both the pretest and the posttest. At baseline (pretest), the mean (standard deviation, SD) test score was 8.3 (1.7). After the training (posttest), the mean (SD) test score increased significantly to 12.2 (2.6) (mean difference: 1.4, *P*=0.027).

We then examined the participants’ knowledge retention by administering the posttest again (retest) four months later. Eight providers participated in both the pretest and the retest (Table 1). At baseline (pretest), the mean (SD) test score was 8.3 (2.3). Four months after training (retest), the mean (SD) test score increased significantly to 10.8 (3.0) (mean difference: 2.5). However, this difference did not attain statistical significance (*P*=0.060).

#### Participant Evaluation

The participants reported how useful they found the training on a scale from 1 (“not useful”) to 5 (“extremely useful”). The median rating among the participants was 5.0 (range: 4–5). In addition, after receiving participant feedback on the potential improvements of training, common ideas (codes) in the participants’ comments were combined into thematic categories. Three open codes were identified from the open-ended questions. These open codes were organized into one thematic category: “Increase Exposure to Training.” Table 2 presents the details of the open codes and themes. Belize is like many LMICs in that pediatric patient care is usually provided by general practitioners. In collaboration with the administration of KHMHA, we identified a need to improve the care of acutely ill pediatric patients. Hence, we created a curriculum to provide general practitioners with the knowledge and skills required for the care of acutely ill children. This curriculum is unique in its multimodal approach and interdisciplinary inclusion.

This study shows that this curriculum format and content may be an appropriate, effective, and a welcome means of teaching the relevant concepts of acute GI illnesses and nutrition; we were able to use a similar format previously to teach concepts of acute respiratory illnesses. The participants described value in this module of the curriculum, rating it as “extremely useful.” Overall, despite the low sample size, there was statistically significant improvement from the pretest to posttest scores which was retained on retest scores several months later (see Table 1). The costs of implementing this module are minimal. This module can be presented independently of the larger pediatric curriculum over a seven-hour period; hence, it requires minimal time commitment from the instructor and learners. This is helpful when considering the staffing needs and educational leave time available for an emergency department. A limitation of this study is the small number of participants. Owing to scheduling constraints and high turnover in staff, we were able to provide this module only to hospital staff available at the time of the course offering. Fewer nurses were available to participate in this module because there was another nurse training course occurring at the same time. A larger number of participants in the hospital or even from the local community may have improved the power of the study and may have led to statistical significance of a retest. An additional limitation of this study is that it was designed according to the needs assessment of one hospital in Belize. Although the development of this curriculum was based on the findings of the largest healthcare center in Belize (i.e., KHMHA), we found that the resources and structure of the KHMHA are not dissimilar to other LMICs.^3^ We strongly believe that, with only minor adaptations, this curriculum would also be useful in other LMICs, particularly for providers in the same geographic region who regularly care for acutely ill or injured children.

The participants provided valuable feedback for improvement. The general feedback that we obtained was very encouraging in that, overall, the participants wanted more teaching (eg, more topics and content, increased time and frequency of training, and more materials to prepare for the module). Compared to the previous module, many participants were understanding of the anticipated time course of the curriculum but unabashedly wanted more opportunities to learn.

Future efforts will be necessary to evaluate the application of concepts taught in the module as it applies to actual patient care. Moreover, as another measure of this curriculum and module’s efficacy, we would like to evaluate the clinical practices of those who participated compared to those who did not. For example, we measured outcomes of our methods of teaching asthma in a prior module which indeed led to some local changes in clinical practice approaches to asthma care.^12^ Additionally, to assess the generalizability of the module and curriculum, we would like to target other regional hospitals for training.

This module enhances the performance of generalist practitioners without altering the scope of their practice and provides a refresher for the management of core acute pediatric GI-related and nutrition illnesses. This curriculum offers a framework for variably trained providers in disseminating practical knowledge and standardizing clinical practice, which, when applied, we anticipate will improve medical care and patient outcomes for a vulnerable pediatric population.

### Associated Content

Appendix A: Curriculum Chart

Appendix B: Example Schedule

Appendix C: Pretest Questions

Appendix D: Pretest Answers

Appendix E: Diarrhea and Dehydration Lecture Synopsis

Appendix F: Diarrhea and Dehydration Lecture

Appendix G: Diarrhea and Dehydration Simulation Case

Appendix H: Small Bowel Obstruction Lecture Synopsis

Appendix I: Small Bowel Obstruction Lecture

Appendix J: Small Bowel Obstruction Simulation Case

Appendix K: Malnutrition Lecture Synopsis

Appendix L: Malnutrition Lecture

Appendix M: Malnutrition Small Group Discussion

Appendix N: Posttest Questions

Appendix O: Posttest Answers

## Figures and Tables

**Figure:**
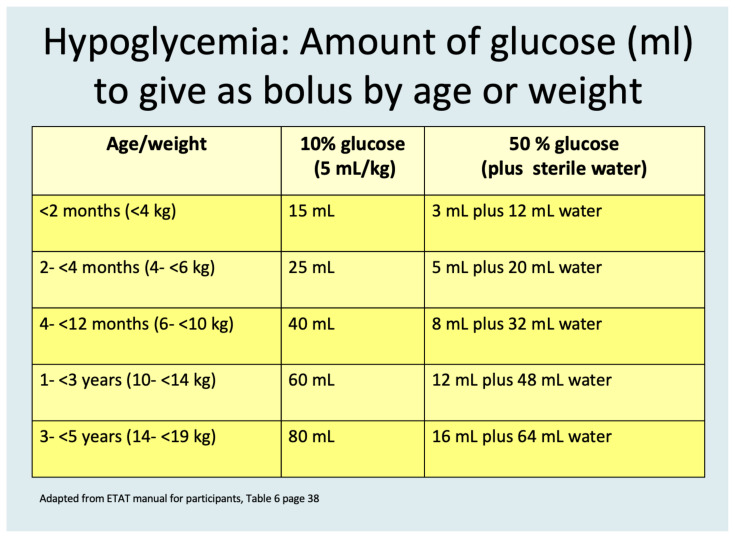
Table Courtesy of Heather Crouse, MD used with permission

**Figure f2-jetem-9-4-c1:**
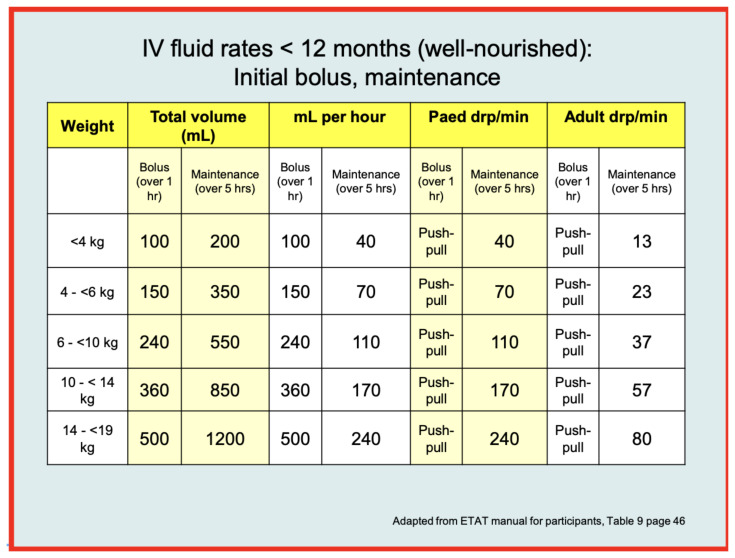
Table Courtesy of Heather Crouse, MD used with permission

**Figure f5-jetem-9-4-c1:**
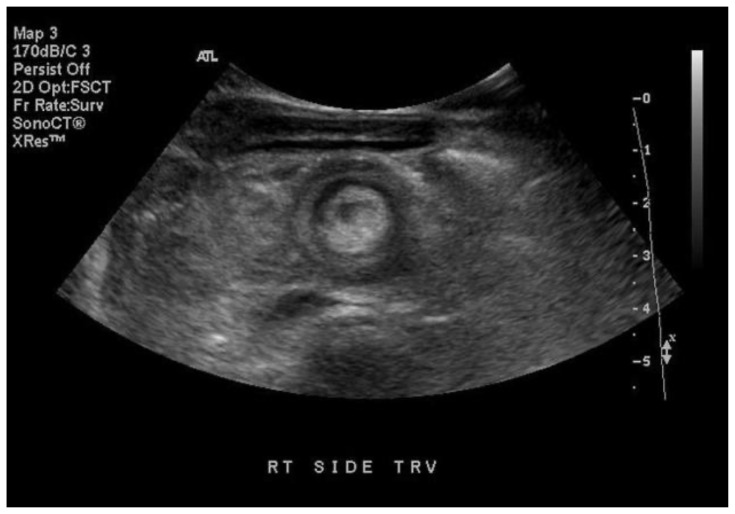
Transverse US shows concentric alternating echogenic and hypoechoic bowel walls. Positive “target sign.” Consistent with ileocolic intussusception.

## DIDACTICS AND HANDS-ON CURRICULUM

### Appendix A Curriculum Chart

**Table:**

Topic	Recommended Educational Strategy	Educational Content	Objectives	Learners	Timing, Resources Needed (Space, Instructors, Equipment, Citations of JETem pubs or other literature)	Recommended Assessment, Milestones Addressed
Pediatric Diarrhea and Dehydration	Lecture describing the presentation and management of pediatric diarrhea and dehydration (Appendix E, F) Simulation session immediately following lecture (Appendix G) For a full description of this session, see Kosoko A, et al. A pediatric emergency medicine refresher course for generalist healthcare providers: Gastrointestinal Emergencies, JETem 2023.	Diagnosis, management, and disposition of the dehydrated child due to diarrhea and gastroenteritis	The learner will be able to identify common viral causes and bacterial causes of gastroenteritis, classify the degree of dehydration in the child presenting with diarrhea, identify a well-nourished child from a malnourished child, outline World Health Organization Plans A, B, and C for treatment of dehydration, choose the appropriate treatment strategy for viral and bacterial gastroenteritis, and anticipate complications of diarrhea and dehydration.	Generalist physician and nurses	Lecture 60 minutes. 1 instructor. Equipment: PowerPoint capable computer, projector, screen. Simulation Session 45 minutes (simulation session). 1 instructor per 5–6 learners. Equipment: A large room with multiple tables and ample floor space, or multiple rooms if available.	Milestone: PC1, PC2, PC3, PC4, PC5, PC6, PC7, MK, ICS1, ICS2 Assessment: Module pretest, posttest, and retest (Appendix C, D, N, O) Simulation: In-person feedback given in real-time; rapid cycle deliberate practice
Pediatric Malnutrition	Lecture describing the presentation and management of difficulties with nutrition of the young child (Appendix K, L) Small group discussion session immediately following lecture (Appendix M) For a full description of this session, see Kosoko A, et al. A Pediatric Emergency Medicine Refresher Course for Generalist Healthcare Providers: Gastrointestinal Emergencies, JETem 2023.	Diagnosis, management, and disposition of the child with malnutrition Appendix, C, D, K, L, M, N, O	The learner will demonstrate the ability to identify malnutrition and its forms, identify malnutrition emergencies and initiate treatment for malnutrition emergencies.	Generalist physician and nurses	Lecture 60 minutes. 1 instructor. Equipment: PowerPoint capable computer, projector, screen. Small Group Discussion 45 minutes. 1 instructor per 3–5 learners. Equipment: A large room with multiple tables and ample floor space, or multiple rooms if available.	Milestone: PC1, PC2, PC3, PC4, PC5, PC6, PC7, MK, ICS1, ICS2 Assessment: Module pretest, posttest, and retest (Appendix C, D, N, O) Small Group Discussion: In-person feedback in real time
Pediatric Small Bowel Obstruction	Lecture describing the presentation and management of pediatric small bowel obstruction (Appendix H, I) Simulation session immediately following lecture (Appendix J) For a full description of this session, see Kosoko A, et al. A Pediatric Emergency Medicine Refresher Course for Generalist Healthcare Providers: Gastrointestinal Emergencies, JETem 2023.	Diagnosis, management, and disposition of the child with small bowel obstruction	The learner will demonstrate the ability to evaluate the child presenting with vomiting and abdominal pain, recognize the clinical presentation of small bowel obstruction, develop a differential diagnosis for acute bowel obstruction in a child, differentiate dangerous causes of vomiting from less concerning causes of vomiting, obtain appropriate studies to evaluate for common causes of pediatric bowel obstruction, and medically manage a child with acute bowel obstruction.	Generalist physician and nurses	Lecture 60 minutes. 1 instructor. Equipment: PowerPoint capable computer, projector, screen. Simulation Session 45 minutes (simulation session). 1 instructor per 5–6 learners. Equipment: A large room with multiple tables and ample floor space, or multiple rooms if available.	Milestone: PC1, PC2, PC3, PC4, PC5, PC6, PC7, MK, ICS1, ICS2 Assessment: Module pretest, posttest, and retest (Appendix C, D, N, O) Simulation: In-person feedback given in real-time; rapid cycle deliberate practice

### Appendix B Example Schedule

[Table t3-jetem-9-4-c1]

**Table t3-jetem-9-4-c1:**

Day 1 GROUP 1	Day 2 GROUP 2	Day 3 GROUP 1	Day 4 GROUP 2
7:30–8:00Introduction	7:30–8:00 Introduction	7:30–8:30 Pediatric Small Bowel Obstruction Lecture	7:30–8:30 Pediatric Small Bowel Obstruction Lecture
8:00–8:30 Pre-Test	8:00–8:30 Pre-Test	8:30–9:30 Pediatric Small Bowel Obstruction Simulation	8:30–9:30 Pediatric Small Bowel Obstruction Simulation
8:30–9:30 Pediatric Diarrhea & Dehydration Lecture	8:30–9:30 Pediatric Diarrhea & Dehydration Lecture	9:30–10:00 Post-Test	9:30–10:00 Post-Test
9:30–10:00 Break	9:30–10:00 Break		
10:00–11:00 Pediatric Diarrhea & Dehydration Simulation	10:00–11:00 Pediatric Diarrhea & Dehydration Simulation		
11:00–12:00 Pediatric Malnutrition Lecture	11:00–12:00 Pediatric Malnutrition Lecture		
12:00–1:00 Pediatric Malutrition Small Group	12:00–1:00 Pediatric Malutrition Small Group		

### Appendix C Pretest Questions

Name: _______________________

1. What is the most common cause of small bowel obstruction in children?
  - Adhesions
  - Hernia
  - Intussusception
  - Midgut volvulus
2. Which of the following statements is false?
  *Reductive adaptation* describes the process in malnutrition in which the body compensates for lack of food by:
  - Decreasing gastrointestinal secretions
  - Decreasing lymphocyte & interleukin production so it is easier for patients to have a fever
  - Reducing arterial blood pressure and favoring central circulation
  - Reducing hemoglobin synthesis
3. An 11-month-old boy is brought in by his parents for vomiting and crying on and off all afternoon. They are concerned that he is in pain. He had one episode of diarrhea yesterday. On exam he is awake and interactive. He begins to pull his legs up and cry for several minutes before relaxing again. Vital signs are blood pressure 96/75mmHg, pulse 98 beats/minute, respirations 32 breaths/minute, and temperature 37.2°C (99°F). Which of the following tests would have the greatest specificity?
  - Complete blood count
  - Contrast enema
  - Plain abdominal x-rays
  - Upper gastrointestinal series
4. Mary is 2-year-old girl brought in by her grandmother because she thinks the child has worms – “her belly is big.” On exam, Mary says “no” when you ask her to come to you. Her breathing is not labored. Her hands are cool to touch, strong pulse and she has a capillary refill of less than 3 seconds. She is alert and active. You notice she has very puffy eyes and swollen feet. Her hair is a light brown and broken off in many areas of her scalp. Her triage vital signs show a temperature of 34.8°C. You have decided to admit her. Of the following, which would not be an appropriate next step in her care:
  - Cover her head
  - Have her sit in the sun while waiting in the queue
  - Initiate nutritional recovery
  - Wrap her in a blanket
5. An unvaccinated 2-year-old male presents with 3 days of non-bloody diarrhea after having 3 days of intermittent non-bloody, non-bilious vomiting. He has been taking fluids well but has had decreased solid intake. There has been no fever. He attends daycare. There has been no travel. The most likely pathogen causing his symptoms is:
  - *Clostridium difficile*
  - *Proteus mirabilus*
  - Rotavirus
  - *Salmonella*
6. A 3-year-old child presents for vomiting and fever. On exam, you find a round, 3 cm tender mass at the left groin. An x-ray obtained shows enlarged bowel loops and air-fluid levels at that same location in the groin.
  - Admit for nasogastric tube decompression
  - Colonoscopy for sigmoid reduction
  - Emergent surgical consultation
  - Incision and drainage
7. In a well-appearing, afebrile 8-year-old female who is presenting with crampy abdominal pain and diarrhea for 5 days, which of the following is the preferred management approach?
  - Oral rehydration and symptomatic outpatient therapy
  - Oral rehydration, laboratory tests, and empiric antibiotic therapy
  - Laboratory tests, empiric antibiotic therapy, and intravenous fluids
  - Laboratory tests, x-ray, empiric antibiotic therapy
8. Karl is an 8-month-old male brought in for multiple episodes of non-bloody diarrhea starting 2 days ago. You notice that he is crying without tears and his breathing is not labored. His hands are warm to touch, a strong pulse and capillary refill of 5 seconds. He is alert. His ribs are easily visible, and he has a “saggy pants” appearance to his posterior. His weight is 6 kg. What is the next best step in management of this patient?
  - Place a nasogastric tube and give ReSoMal 30cc every 30 minutes for 2 hours
  - Place a nasogastric tube and start F-75 as soon as possible
  - Start an IV and give D5½ normal saline 60–90 cc over 1 hour
  - Start an IV and give 120cc over 1 hour
9. A 10-year-old female presents for vomiting after having visited a friend's birthday party. Which of the following statements regarding *Staphylococcal* food poisoning is correct?
  - A single person ingesting food cannot be the only person of a group affected
  - Food contaminated by Staphylococcus has a bitter, spicy taste
  - Profuse, bloody diarrhea is the distinguishing characteristic
  - Symptoms begin within 1–6 hours of ingestion
10. A newborn boy presents with projectile vomiting and is always hungry to eat more after vomiting. The vomit is non-bilious, and an olive shaped mass is palpable in the patient’s abdomen. The patient has been treated with erythromycin for an infection. What is the best initial diagnostic test?
  - Computed tomography of abdomen
  - Hypokalemic, hypochloremic metabolic alkalosis
  - Ultrasound pylorus
  - X-ray of the abdomen
11. Which of the following could indicate a malrotation of the midgut and volvulus on x-ray of the abdomen?
  - Corkscrew sign on x-ray abdomen
  - Double bubble sign on x-ray abdomen
  - Whirlpool sign on ultrasound
  - All the above
12. He has had vomiting and diarrhea for 3 days. On exam, he moans, his breathing is not labored and his capillary refill is 4 seconds. His hands are warm and his pulse is strong. He is awake but listless. You check his blood glucose and it is 44 mg/dL. What is the next appropriate step?
  - Place an IV and give him 15cc of D50
  - Place an IV and give him 30cc of D10
  - Place a nasogastric tube and give him 150cc of D10
  - Place a nasogastric tube and give him 30cc of D25
13. A 3-year-old male presents to the emergency department with frequent diarrhea for 3 days. On exam, he has sunken eyes and skin pinch is 2 seconds. He is more irritable than usual, but is alert, and when offered liquids, drinks eagerly. How would you classify his degree of dehydration?
  - Mild dehydration
  - Moderate dehydration
  - No dehydration
  - Severe dehydration
14. Which of the following statements about nutritional recovery is true?
  - Diuretics should be used to decrease the peripheral edema seen in severe malnutrition
  - F-75 provides complete nutrition for patients with malnutrition
  - Patient presenting with severe malnutrition should be empirically treated with antibiotics
  - Refeeding syndrome causes rhabdomyolysis and cardiac infarcts secondary to hyponatremia
15. A 6-year-old girl presents to the emergency department with poor urine output. She had bloody diarrhea a few last weeks with fevers that have now resolved. On exam, she is pale and ill appearing. Your next step is:
  - Administer an IV isotonic fluid bolus of 40 mL/kg
  - Check labs including full blood count, electrolytes, and renal function
  - Discharge home with oral rehydration instructions
  - Send stool cultures and begin antibiotics

### Appendix D Pretest Answers

1. What is the most common cause of small bowel obstruction in children?
  - Adhesions
  - Hernia
  - **Intussusception**
  - Midgut volvulus
2. Which of the following statements is false?
  *Reductive adaptation* describes the process in malnutrition in which the body compensates for lack of food by:
  - Decreasing gastrointestinal secretions
  - **Decreasing lymphocyte & interleukin production so it is easier for patients to have a fever**
  - Reducing arterial blood pressure and favoring central circulation
  - Reducing hemoglobin synthesis
3. An 11-month-old boy is brought in by his parents for vomiting and crying on and off all afternoon. They are concerned that he is in pain. He had one episode of diarrhea yesterday. On exam he is awake and interactive. He begins to pull his legs up and cry for several minutes before relaxing again. Vital signs are blood pressure 96/75mmHg, pulse 98 beats/minute, respirations 32 breaths/minute, and temperature 37.2°C (99°F). Which of the following tests would have the greatest specificity?
  - Complete blood count
  - **Contrast enema**
  - Plain abdominal x-rays
  - Upper gastrointestinal series
4. Mary is 2-year-old girl brought in by her grandmother because she thinks the child has worms – “her belly is big.” On exam, Mary says “no” when you ask her to come to you. Her breathing is not labored. Her hands are cool to touch, strong pulse and she has a capillary refill of less than 3 seconds. She is alert and active. You notice she has very puffy eyes and swollen feet. Her hair is a light brown and broken off in many areas of her scalp. Her triage vital signs show a temperature of 34.8°C. You have decided to admit her. Of the following, which would not be an appropriate next step in her care:
  - Cover her head
  - **Have her sit in the sun while waiting in the queue**
  - Initiate nutritional recovery
  - Wrap her in a blanket
5. An unvaccinated 2-year-old male presents with 3 days of non-bloody diarrhea after having 3 days of intermittent non-bloody, non-bilious vomiting. He has been taking fluids well but has had decreased solid intake. There has been no fever. He attends daycare. There has been no travel. The most likely pathogen causing his symptoms is:
  - *Clostridium difficile*
  - *Proteus mirabilus*
  - **Rotavirus**
  - *Salmonella*
6. A 3-year-old child presents for vomiting and fever. On exam, you find a round, 3 cm tender mass at the left groin. An x-ray obtained shows enlarged bowel loops and air-fluid levels at that same location in the groin.
  - Admit for nasogastric tube decompression
  - Colonoscopy for sigmoid reduction
  - **Emergent surgical consultation**
  - Incision and drainage
7. In a well-appearing, afebrile 8-year-old female who is presenting with crampy abdominal pain and diarrhea for 5 days, which of the following is the preferred management approach?
  - **Oral rehydration and symptomatic outpatient therapy**
  - Oral rehydration, laboratory tests, and empiric antibiotic therapy
  - Laboratory tests, empiric antibiotic therapy, and intravenous fluids
  - Laboratory tests, x-ray, empiric antibiotic therapy
8. Karl is an 8-month-old male brought in for multiple episodes of non-bloody diarrhea starting 2 days ago. You notice that he is crying without tears and his breathing is not labored. His hands are warm to touch, a strong pulse and capillary refill of 5 seconds. He is alert. His ribs are easily visible, and he has a “saggy pants” appearance to his posterior. His weight is 6 kg. What is the next best step in management of this patient?
  - **Place a nasogastric tube and give ReSoMal 30cc every 30 minutes for 2 hours**
  - Place a nasogastric tube and start F-75 as soon as possible
  - Start an IV and give D5½ normal saline 60–90 cc over 1 hour
  - Start an IV and give 120cc over 1 hour
9. A 10-year-old female presents for vomiting after having visited a friend's birthday party. Which of the following statements regarding *Staphylococcal* food poisoning is correct?
  - A single person ingesting food cannot be the only person of a group affected
  - Food contaminated by Staphylococcus has a bitter, spicy taste
  - Profuse, bloody diarrhea is the distinguishing characteristic
  - **Symptoms begin within 1–6 hours of ingestion**
10. A newborn boy presents with projectile vomiting and is always hungry to eat more after vomiting. The vomit is non-bilious, and an olive shaped mass is palpable in the patient’s abdomen. The patient has been treated with erythromycin for an infection. What is the best initial diagnostic test?
  - Computed tomography of abdomen
  - Hypokalemic, hypochloremic metabolic alkalosis
  - **Ultrasound pylorus**
  - X-ray of the abdomen
11. Which of the following could indicate a malrotation of the midgut and volvulus on x-ray of the abdomen?
  - Corkscrew sign on x-ray abdomen
  - Double bubble sign on x-ray abdomen
  - Whirlpool sign on ultrasound
  - **All the above**
12. He has had vomiting and diarrhea for 3 days. On exam, he moans, his breathing is not labored and his capillary refill is 4 seconds. His hands are warm and his pulse is strong. He is awake but listless. You check his blood glucose and it is 44 mg/dL. What is the next appropriate step?
  - Place an IV and give him 15cc of D50
  - Place an IV and give him 30cc of D10
  - Place a nasogastric tube and give him 150cc of D10
  - **Place a nasogastric tube and give him 30cc of D25**
13. A 3-year-old male presents to the emergency department with frequent diarrhea for 3 days. On exam, he has sunken eyes and skin pinch is 2 seconds. He is more irritable than usual, but is alert, and when offered liquids, drinks eagerly. How would you classify his degree of dehydration?
  - Mild dehydration
  - **Moderate dehydration**
  - No dehydration
  - Severe dehydration
14. Which of the following statements about nutritional recovery is true?
  - Diuretics should be used to decrease the peripheral edema seen in severe malnutrition
  - F-75 provides complete nutrition for patients with malnutrition
  - **Patient presenting with severe malnutrition should be empirically treated with antibiotics**
  - Refeeding syndrome causes rhabdomyolysis and cardiac infarcts secondary to hyponatremia
15. A 6-year-old girl presents to the emergency department with poor urine output. She had bloody diarrhea a few last weeks with fevers that have now resolved. On exam, she is pale and ill appearing. Your next step is:
  - Administer an IV isotonic fluid bolus of 40 mL/kg
  - **Check labs including full blood count, electrolytes, and renal function**
  - Discharge home with oral rehydration instructions
  - Send stool cultures and begin antibiotics

### Appendix E Diarrhea and Dehydration Lecture Synopsis

**Title**: Diarrhea and Dehydration Lecture

**Target Audience:** generalist healthcare providers in low- and middle-income countries

**Educational Methods:** PowerPoint didactic lecture

**Time required for implementation:** about 60 minutes

**Equipment/Environment/Personnel:**

- *A medium-sized room (with a capacity of at least 25 people) with multiple tables and ample floor space (to split into groups), or multiple rooms if available*
- *A computer with PowerPoint capability and projector setup*
- *Personnel needed: one lecturer*

**Learning Objectives:**

The learner will be able to:

1. Identify viral causes and bacterial causes of gastroenteritis
2. Classify the degree of dehydration in the child presenting with diarrhea
3. Identify a well-nourished child from a malnourished child in order to determine best management of dehydration
4. Outline World Health Organization Plans A, B, and C for treatment of dehydration
5. Choose the appropriate treatment strategy for viral and bacterial gastroenteritis
6. Anticipate complications of gastroenteritis

**Lecture Script** [Table t4-jetem-9-4-c1]

**Table t4-jetem-9-4-c1:**

Slide 1	Title slide. “We are going to talk about a ubiquitous subject, diarrhea in kids.” Engage the audience asking for a show of hands who has treated pediatric diarrhea. Ask the audience to describe any remarkable cases they remember, any challenges with diagnosis or management, and why some patients went home versus which patients were admitted.
Slide 2	Key
Slide 3	By the end of this lecture, you should be able to: Identify viral causes and bacterial causes of gastroenteritis Classify the degree of dehydration in the child presenting with diarrhea Identify a well-nourished child from a malnourished child to determine best management of dehydration Outline World Health Organization Plans A, B, and C for treatment of dehydration Choose the appropriate treatment strategy for viral and bacterial gastroenteritis Anticipate complications of gastroenteritis
Slide 4	An introduction to a common presentation of a child with gastroenteritis. Read case aloud. Use this as an opportunity to discuss differential diagnosis, how sick child seems, what are the ways you can treat diarrhea (reassurance, oral rehydration, intravenous hydration, etc.) with the audience.
Slide 5	This case likely describes gastroenteritis. What is gastroenteritis? Technically, defined as 3 or more loose stools in 24 hours. It can be accompanied by other gastrointestinal (GI) symptoms such as vomiting, abdominal pain, or fever. Typical symptoms for acute gastroenteritis may last ***up to two weeks.*** The majority of cases are viral.
Slide 6	This slide discusses distinguishing between viral and bacterial causes of diarrhea. There is more concern for bacterial source if there is bloody diarrhea, high fever, or the child has a risk factor (eg, travel, contaminated foods, or water sick contacts).
Slide 7	For children under the age of 5, the most common etiologies of diarrhea included Rotavirus, *E*. coli, *Shigella* and *Cryptosporidium* in a study of over 20,000 children presenting for treatment in African and Southeast Asia (The Gambia, Mali, Mozambique, Kenya, Bangladesh, India, Pakistan). Liu J, Platts-Mills JA, Juma J, et al. Use of quantitative molecular diagnostic methods to identify causes of diarrhoea in children: a reanalysis of the GEMS case-control study. *Lancet*. 2016;388(10051):1291–1301. doi:10.1016/S0140-6736(16)31529-X
Slide 8	The two most common causes of viral gastroenteritis include rotavirus and norovirus. Rotavirus is most common in younger children and historically has been the most common cause of severe enough diarrhea that caregivers seek medical care, although rates of rotavirus diarrhea are (thankfully) decreasing due to vaccination campaigns. Norovirus is common across all ages and tends to be associated with outbreaks (eg, contaminated food, water). Rivera-Dominguez G, Ward R. Pediatric Gastroenteritis. In: *StatPearls*. Treasure Island (FL): StatPearls Publishing; April 3, 2023.
Slide 9	Bacterial disease is more common in older children. Presentation will usually include bloody stools, high fever, and severe abdominal pain. The most common pathogens include *E*. coli, *Shigella*, *Campylobacter*, and *Yersinia*. Suspicion of particular bacteria will help guide antibiotic choices.
Slide 10	Food-borne gastroenteritis (“food poisoning”) is another common cause of diarrhea disease in children. Two major pathogens are *S.* aureus and *Bacillus* which produce enterotoxins which contaminate food and/or water. Due to the toxins, symptoms usually develop within hours after ingestion: acute onset nausea, vomiting, and diarrhea. Typically, the symptoms are self-limited and only require supportive care. *S. aureus* usually shows quicker onset than *B. cereus* but both tend to present within 16 hours Rivera-Dominguez G, Ward R. Pediatric Gastroenteritis. In: *StatPearls*. Treasure Island (FL): StatPearls Publishing; April 3, 2023.
Slide 11	What are some questions you want to ask when a child presents with diarrhea? Request responses from the audience, and then review the list.
Slide 12	A rapid, focused exam can assess for shock or severe dehydration. It is important to also check for signs of anemia or liver dysfunction such as pallor, jaundice, or bruising.
Slide 13	Children do not typically present in shock the way adults or adolescents do. They tend to reach a tipping point and then crash when they can no longer compensate. Do not ignore delayed capillary refill, cool extremities, or a fast and weak pulse. One easy way to examine a child for this: touch is/her hand. If it is warm and capillary refill is normal (<2 seconds), the child is unlikely in shock. Remember that unlike adults, children will not be hypotensive until late in a shock state.
Slide 14	If you identify shock, STOP and TREAT immediately. Do not continue with your assessment.
Slide 15	Diarrhea accounts for more than 1/10 neonatal deaths worldwide! While common, it has significant mortality if danger signs are not recognized and treated. If a child is showing warning signs of severe illness such as shock, treatment should be initiated immediately. Liu L, Johnson HL, Cousens S, et al. Global, regional, and national causes of child mortality: an updated systematic analysis for 2010 with time trends since 2000 [published correction appears in Lancet. 2012 Oct 13;380(9850):1308]. *Lancet*. 2012;379(9832):2151–2161. doi:10.1016/S0140-6736(12)60560-1
Slide 16	Two of the most immediate and quick physical exam findings for severe dehydration include the eyes and the skin. Sunken eyes can indicate severe dehydration but could also help identify malnutrition (should discuss chronicity of appearance with parents). A skin pinch which is slow to return to normal is a sign that can also indicate severe dehydration.
Slide 17	This table is from the World Health Organization (WHO) Integrated management of childhood illness guidelines for assessing the degree of pediatric dehydration. You can see it progresses from left to right indicating no dehydration to severe dehydration. We can use these findings to objectively classify the degree of dehydration and select the appropriate treatment plan (Plan A, B or C).
Slide 18	Of note, if a child presents with a history of diarrhea or vomiting and is lethargic, please check their blood sugar early in the clinical course. Such children may be unable to keep up with their metabolic needs and may have altered mentation due to hypoglycemia. If you cannot check a blood sugar, err on the side of empirically administering glucose. Listed are the values of blood glucose that would indicate hypoglycemia.
Slide 19	How do you give glucose? An easy rule of thumb for intravenous intervention is the “Rule of 50s” wherein depending on the concentration of dextrose you have available, you can determine the appropriate pediatric weight-based dosing of glucose: **Dose (mL/kg) × Concentration = 50** Therefore: D50%: 1 mL/kg D10%: 5 mL/kg D5%: 10 mL/kg The instructor can work through some examples of appropriate calculations with the learners.
Slide 20	It is critical to be able to assess the severity of dehydration because this determines your treatment plan.
Slide 21	The WHO divides diarrhea into three treatment plans based on severity of dehydration. Plan A = not dehydrated. These children can receive supportive care at home for diarrhea. Plan B = moderately dehydrated. These children should to be given fluids using the enteric system (orally or by gastric tube) in clinic or the emergency department and reassessed for appropriate disposition. Plan C= severely dehydrated. These children need to be transferred to a capable hospital and given intravenous (IV) fluids (if well nourished) and reassessed.
Slide 22	Plan A: if there are less than 2 features from Columns B or C, there is no evidence of dehydration.
Slide 23	This child can be managed at home, fed normally, and be given Oral Rehydration Solution (ORS) to take after each stool to compensate for losses.
Slide 24	Plan B: If there are 2 or more features from column B, then the child has some (moderate) dehydration and needs to be monitored in the clinic or emergency department.
Slide 25	This child should be started on oral or gastric fluids in clinic or in the emergency setting (75 ml/kg total over four hours) and then reassessed.
Slide 26	If you do not know the child’s weight, this table provides an estimate of the fluid to be given over the 4 hours based on the child’s age.
Slide 27	Plan C: If there are 2 or more features from column B, then the child has severe dehydration and needs to be referred to a capable hospital.
Slide 28	The heart of Plan C is IV fluids (for well-nourished children). This often requires a hospital setting. You will give an initial bolus (rate depends on child’s age) followed by maintenance fluids for the next five hours (see table).
Slide 29	How can you tell if a child is malnourished? Three main signs are wasting, edema, and decreased upper arm circumference. It is important to identify malnourished children not only because they need different treatment for dehydration but also because some of the typical signs of dehydration (eg, sunken eyes, delayed skin pinch, lethargy) are not reliable indicators of dehydration because they could just as well be due to malnutrition. Because they may have poor cardiac function associated with their chronic malnourishment, these children are susceptible to heart failure and could die if given rapid, unnecessary IV fluids.
Slide 30	If you have assessed the child and there are no signs of malnutrition, you can then begin IV fluids for severe dehydration. You will give 30 cc/kg over the first 0.5–1 hour (depending on age, see table) and another 70 cc/kg over the next 2.5–5 hours (again depending on age). Reassess the severely dehydrated child every 15–30 minutes and add oral rehydration solution as soon as the child will take it.
Slide 31	For malnourished children, you DO NOT GIVE IV FLUIDS UNLESS THEY ARE IN SHOCK. Why? Because their cardiac function is presumed poor, as is their protein status. Malnourished children given IV fluids too quickly can become fluid overloaded and die. Instead, start oral or gastric fluids with oral rehydration solution (ORS, Re-So-Mal) at 5 ml/kg every 0.5 hour for first 2 hours and then every 0.5–1 hour for the next 4 hours. These children need regular and frequent reassessment.
Slide 32	To summarize, for severe dehydration (i.e., Plan C) you are going to reassess frequently, give IV fluids if well-nourished, or oral or gastric fluids if malnourished. Well-nourished children can add ORS by mouth as soon as the child will take it. You then reevaluate/reclassify in 3 hours (older child) or 6 hours (infant).
Slide 33	What if you can’t obtain IV access? Another option for resuscitation is to place a nasogastric (NG) tube and start hydration that way as 10 ml/kg in a well-nourished child every 30 minutes with frequent reassessment. If you notice abdominal distention, slow down the rate of fluids resuscitation. Like the IV fluid plan previously, reassess and reclassify after 6 hours.
Slide 34	How do you place an NG tube? Have an audience member who is familiar explain to the group. The facilitator should be prepared to describe how to place an NG tube: Measure the approximate distance depth of insertion from the nose to the ear to the epigastrium. Insert the NG tube through one of the patient’s nostrils using lubricant. Gently advance the NG tube through the nasopharynx. Continue to advance the NG tube. You can ask the patient to swallow some sips of water to facilitate advancement of the tube. Avoid giving patients a drink if their swallow is deemed unsafe due to the risk of aspiration. Once you reach the desired nasogastric tube insertion length, fix the NG tube to the nose with a dressing. Confirm placement by aspirating gastric contents, listening for borborygmi and/or getting a radiograph.
Slide 35	For all children, encourage breastfeeding if they were previously doing so before they became ill. Make sure caregivers know how to mix the ORS and administer it. Providers and caregivers can slowly reintroduce foods after several hours in older children and consider zinc supplementation. Ensure caregivers know how to mix Oral Rehydration Solution (ORS) and how much to give: Clean hands, utensils & water Mix 1 packet of ORS with 1L of clean water OR Mix 3.5g salt and 40g sugar in 1L of clean water (if ORS packets unavailable) Give ½ L/day to babies or toddlers or 1L/day to older children
Slide 36	Let’s briefly discuss some of the other therapies that can be helpful with diarrhea.
Slide 37	Probiotics have a modest effect on recovery, and they are most effective among patients who may have previously taken antibiotics contributing to the presentation of diarrhea. They may shorten the duration of illness. Guarino A, Lo Vecchio A, Canani RB. Probiotics as prevention and treatment for diarrhea. *Curr Opin Gastroenterol*. 2009;25(1):18–23. doi:10.1097/MOG.0b013e32831b4455
Slide 38	Zinc supplementation can be useful in areas where zinc deficiency or moderate malnutrition are high in children greater than 6 months of age. However, adding zinc may only shorten duration of diarrheal illness by about 1 day on average. Lazzerini M, Wanzira H. Oral zinc for treating diarrhoea in children. *Cochrane Database Syst Rev*. 2016;12(12):CD005436. Published 2016 Dec 20. doi:10.1002/14651858.CD005436.pub5
Slide 39	Zinc can be given in an oral electrolyte solution or as a tablet. It is administered as 10 mg (1/2 tablet) for children aged < 6 mo or 20 mg (1 tablet) in children >6 mo.
Slide 40	In general, antibiotics are not indicated for an acute diarrheal illness in children and can cause more severe disease, such as hemolytic uremic syndrome (HUS). DO NOT give antibiotics unless a specific pathogen has been isolated or highly suspected. Most children will improve with supportive care alone and time. Bajait C, Thawani V. Role of zinc in pediatric diarrhea. *Indian J Pharmacol.* 2011 May;43(3):232.
Slide 41	Hemolytic Uremic Syndrome (HUS) is a potentially fatal complication of bacterial enteritis. It is most common seen with *E*. coli O157:H7. It usually begins about a week after diarrheal onset and presents with a triad of: 1) hemolytic anemia, 2) thrombocytopenia, and 3) acute renal failure. Cody EM, Dixon BP. Hemolytic uremic syndrome. *Pediatric Clinics*. 2019 Feb 1;66(1):235–46.
Slide 42	The treatment for HUS is mostly supportive. If the child is anemic, you can transfuse packed red blood cells. If the child is thrombocytopenic, only transfuse if there is active bleeding, the patient needs to be medically optimized for an invasive procedure, or perhaps if platelets count is <10k. Recognize that the child may have impaired renal function and may not tolerate high volume fluids and show signs of fluid overload. What does that look like? Edema, jugular venous distention, hepatomegaly, splenomegaly, dyspnea, pulmonary basilar rales. In significant renal failure, some patients may even need emergency hemodialysis. Cody EM, Dixon BP. Hemolytic uremic syndrome. *Pediatric Clinics*. 2019 Feb 1;66(1):235–46.
Slide 43	Let us revisit our case. Ask audience to classify degree of dehydration and explain their decision-making.
Slide 44	Discuss case management, and review protocol for evaluating diarrhea and dehydration in children. Evaluate for signs of shock: Normal vitals, strong pulses Evaluate nutrition: Not malnourished Assess degree of dehydration: Sunken eyes, slow skin pinch, but alert and eagerly drinking Which Plan does child fall into? -Plan B: Some dehydration What is the treatment? −75 mL/kg over 4 hours of ORS You reassess in 4 hours; child is improved. Now what do you do? -Go to plan A, add zinc Cody EM, Dixon BP. Hemolytic uremic syndrome. *Pediatric Clinics*. 2019 Feb 1;66(1):235–46.
Slide 45	In summary: Diarrhea may have many causes, with viral gastroenteritis being the most common. Rehydration should be dependent on degree of dehydration and nutrition status. WHO Plans A, B, and C can be used to guide rehydration of well-nourished children with diarrhea and dehydration. Treatment of diarrhea is primarily supportive. HUS is a life-threatening complication.
Slide 46	References are provided here. Any questions?

**References:**

- Kotloff KL, Nataro JP, Blackwelder WC, et al. Burden and aetiology of diarrhoeal disease in infants and young children in developing countries (the Global Enteric Multicenter Study, GEMS): a prospective, case-control study. *Lancet*. 2013;382(9888):209–222. doi:10.1016/S0140-6736(13)60844-2
- Liu L, Johnson HL, Cousens S, et al. Global, regional, and national causes of child mortality: an updated systematic analysis for 2010 with time trends since 2000. *The Lancet*. 2012 Jun 9;379(9832):2151–61.
- Rivera-Dominguez G, Ward R. Pediatric Gastroenteritis. In: *StatPearls*. Treasure Island (FL): StatPearls Publishing; April 3, 2023.
- Pocket Book of Hospital Care for Children: Guidelines for the Management of Common Childhood Illnesses. 2nd edition. (World Health Organization, 2013).
- Guarino A, Lo Vecchio A, Canani RB. Probiotics as prevention and treatment for diarrhea. *Curr Opin Gastroenterol*. 2009;25(1):18–23. doi:10.1097/MOG.0b013e32831b4455
- Lazzerini M, Ronfani L. Oral zinc for treating diarrhoea in children. Cochrane Database Syst Rev. 2013;(1):CD005436. Published 2013 Jan 31. doi:10.1002/14651858.CD005436.pub4
- Bajait C, Thawani V. Role of zinc in pediatric diarrhea. *Indian J Pharmacol*. 2011 May;43(3):232. Cody EM, Dixon BP. Hemolytic uremic syndrome. *Pediatric Clinics*. 2019 Feb 1;66(1):235–46.

### Appendix F Diarrhea and Dehydration Lecture

Please see associated PowerPoint file

### Appendix G Diarrhea and Dehydration Simulation Case

**Case Title:** Pediatric Hypovolemic Shock Rapid Cycle Deliberate Practice (RCDP) Case

**Time required for implementation:** ~ 45 minutes for multiple rounds of RCDP

**Recommended number of learners per instructor/case:**

- One simulation instructor/debriefing facilitator per group of three to six learners
  ○ This person should be well-versed in the medical theory taught by the simulations presented
- One actor/assistant per group of three to six learners

**Learner responsible content:** Appendix C. Diarrhea and Dehydration

**Objectives:**

By the end of the session, learners should be able to:

Cognitive:

1. Recognize hypovolemic shock in a child
2. Categorize severe dehydration
3. Understand the need for rapid treatment and reassessment
4. Identify complications of severe gastroenteritis

Technical:

1. Perform a rapid initial assessment
2. Perform peripheral IV line or IO placement
3. Calculate glucose dosing for a pediatric patient

Behavioral:

1. Communicate clear leadership roles with delegation of roles
2. Perform early interventions for hypovolemic shock

**Abbreviations** [Table t5-jetem-9-4-c1]

**Table t5-jetem-9-4-c1:**

AED =	automatic external defibrillator
BP =	blood pressure
Bpm =	beats per minute
BVM =	bag valve mask
CPR =	cardiopulmonary resuscitation
EKG =	electrocardiogram
EMS =	emergency medical services
GCS =	Glasgow Coma Scale
HR =	heart rate
IO =	intraosseous
IV =	intravenous
PIV =	percutaneous intravenous
LR =	Ringer’s lactate
NS =	normal saline
O2 =	oxygen
PEA =	pulseless electrical activity
Pt =	patient
RCDP =	rapid cycle deliberate practice
RR =	respiratory rate
O_2_Sat =	oxygen saturation
SpO2 =	oxygen saturation
T =	temperature

**References:**

1. Kotloff KL, Nataro JP, Blackwelder WC, et al. Burden and aetiology of diarrhoeal disease in infants and young children in developing countries (the Global Enteric Multicenter Study, GEMS): a prospective, case-control study. *Lancet.* 2013;382(9888):209–222. doi:10.1016/S0140-6736(13)60844-2
2. Pocket Book of Hospital Care for Children: Guidelines for the Management of Common Childhood Illnesses. 2nd edition. (World Health Organization, 2013).
3. Lazzerini M, Ronfani L. Oral zinc for treating diarrhoea in children. Cochrane Database Syst Rev. 2013;(1):CD005436. Published 2013 Jan 31. doi:10.1002/14651858.CD005436.pub4
4. Guarino A, Lo Vecchio A, Canani RB. Probiotics as prevention and treatment for diarrhea. *Curr Opin Gastroenterol*. 2009;25(1):18–23. doi:10.1097/MOG.0b013e32831b4455

**Case Title:** Pediatric Pneumonia RCDP Practice Case

**Case description & diagnosis (short synopsis):**

Marcus, a 6-month-old male is brought into the emergency department with his mother. He has been having two days of diarrhea with some vomiting. He has unstable vital signs due to hypovolemic shock. He requires rapid fluid resuscitation. He will deteriorate if not treated expeditiously. If appropriate and time sensitive therapy occurs, he will have improvement of his vital signs and general appearance. If inappropriate or delayed interventions, the patient will decompensate to pulseless electrical activity (PEA) due to hypovolemia.

**Equipment or props needed:**

Setup for All Rounds:

- Room configuration: typical emergency bed/stretcher (or table)
- For each group of three to five learners, one equipment setup includes the following:
  ○ A low-fidelity full-body simulation mannequin. If available, higher-fidelity mannequins can be used (we used a MegaCode Kid, and Laerdal ALS Baby mannequins)
  ○ An intravenous arm task trainer (if the mannequin is not equipped)
  ○ Lower extremity capable of intraosseous (IO) insertion (if mannequin is not equipped)
  ○ A medical resuscitation setup including the standard resuscitation equipment available in the emergency department (intravenous line starter kits, intravenous fluids, IO drill, IO needles, medical tape, bag valve mask, mock medications, etc.)
- Personnel:
  ○ Simulation instructor/debriefer
  ○ Actors: patient’s mother (Maria), inpatient pediatrician (telephone voice)
  ○ Demonstration items needed for debriefing: same as equipment

**Ideal Scenario Flow:**

Marcus is brought to a bed with his mother. The learners are expected to obtain a full set of vital signs and recognize that his vital signs are unstable. They should also perform an initial survey. The learners should interview the mother and obtain a history of vomiting and diarrhea suggestive of severe gastroenteritis. Participants should be concerned for shock and severe dehydration, and they should assess for malnutrition before giving fluids and obtaining vascular access for labs and other potential medication administration. The learners should give an appropriate weight-based bolus of fluids.

After their interventions, learners should reassess the patient including a reassessment of vital signs. They should recognize persistent shock and order a second bolus of IV fluids. They should also reassess mental status and consider hypoglycemia, either checking blood glucose or empirically giving dextrose at the appropriate dose. They will need to interpret any laboratory studies which they obtain. No imaging is necessarily indicated in this case. The participants should end the case with a handoff to the inpatient physician – summarizing the case using appropriate medical terminology.

If inappropriate or delayed interventions, she will decompensate to Pulseless Electrical Activity (PEA) arrest due to hypovolemia.

**Critical Actions:**

Round 1:

□ Rapid assessment of airway, breathing, and circulation
□ Check vital signs
□ Recognize abnormal vital signs
□ Obtain IV/IO access
□ Take basic history from the mother

Round 2:

□ Recognize hypovolemic shock
□ Estimate patient weight
□ Give appropriate parenteral fluid bolus
□ Reassess patient after the bolus

Round 3:

□ Recognize continued hypotension and shock state
□ Administer another parenteral fluid bolus
□ Check blood glucose for altered mental status
□ Administer parenteral dextrose

**Expected Endpoint of the Scenario:** maximum 10-minute time limit per round, but facilitator can stop at any point during scenario

**Possible distractors within scenario**: high acuity patient, family would like antibiotics

**Optional Challenges for Higher Level Learners**:

- Considering other types of shock
- PEA arrest due to hypovolemic shock

**Roles of Participants/Trainees:** usual roles within the emergency department

**Roles of Actors:**

Mother (Maria): provides medical history, primary caretaker of patient

Inpatient Physician (by instructor voice): takes handoff for transition of care from learners

**Anticipated Management Errors:**

*Failure to identify a lethargic patient: Because this is a low-fidelity simulation and the patient is not interacting directly, learners may not recognize that the patient is lethargic and may require glucose. It may be useful for the mother to indicate that she is concerned about the child's alertness if the learners do not explicitly evaluate the child’s neurologic status.*

**Chief complaint:** “Two days of vomiting and diarrhea”[Table t6-jetem-9-4-c1]

**Table t6-jetem-9-4-c1:**

**Initial Vitals for all rounds:**	HR 200	BP 50/34	RR 40	Temp 36.5 °C
SpO2 98%	Weight 7 kg			

**Initial Physical Exam for all rounds:**

**General appearance:** Patient is somnolent but arousable. Pale. Dry mucous membranes

**Initial Assessment:**

- **Airway/Breathing:** Airway open. Lungs with shallow respirations, clear bilaterally
- **Circulation:** No murmur, weak central and peripheral pulses. Capillary refill 4–5 seconds, extremities cool
- **Other:** Poor skin turgor, dry mucous membranes, eyes sunken. Abdomen soft, bowel sounds hyperactive. Lethargic, brief cry to interventions but minimally active on exam.

#### ROUND 1: Initial assessment of patient

**Objectives introduced this round**:

1. Communicate clear leadership roles with delegation of roles
2. Perform a rapid initial assessment
3. Check and analyze vital signs
4. Perform peripheral IV line or IO placement

**Prompt for team:** “You have been called to evaluate a sick infant. The patient is a 6-month male with two days of vomiting and diarrhea. His mother reports he is very sleepy today.”

**Pertinent history (if asked):**

History of Present Illness: The child has been having 8–10 unformed, liquid stools each day the past two days. A few hours ago, he started vomiting. This prompted his mother to bring him for evaluation. He does not seem interested in eating or taking from the breast or the bottle. He is sleepier today. No recent antibiotic use. Two of his older cousins have been having vomiting and diarrhea, but they are four and five years old. Mother is unsure the last time she saw the child make urine explicitly.

Review of Systems:

Positive: nonbloody, nonbilious emesis, nonbloody diarrhea

Negative: fever, night sweats, weight loss, lymphadenopathy, ecchymosis, rash, vision change, rhinorrhea, sinus pain, epistaxis, dental problems, weight change, palpitations, syncope, edema, cyanosis, orthopnea, hemoptysis, nausea, vomiting, diarrhea, blood in stool, appetite change, abdominal pain, dysuria, frequency, urgency, hematuria, joint pain or swelling, muscle pain, back pain, headache, weakness

Medications: none

Medical & Surgical History: none

FH: Two of his older cousins have been having vomiting and diarrhea

Social Hx: lives at home with mother, does not go to daycare[Table t7-jetem-9-4-c1]

**Table t7-jetem-9-4-c1:**

**Vital signs** (need to be obtained):	HR 200	BP 50/34	RR 40	Temp 36.5 °C
SpO2 98%	Weight 7 kg			

**Physical exam findings (if asked):**

**General appearance:** Patient is somnolent but arousable. Pale. Dry mucous membranes

**Initial Assessment:**

- **Airway/Breathing:** Airway open. Lungs with shallow respirations, clear bilaterally
- **Circulation:** No murmur, weak central and peripheral pulses. Capillary refill 4–5 seconds, extremities cool
- **Other:** Poor skin turgor, dry mucous membranes, eyes sunken. Abdomen soft, bowel sounds hyperactive. Lethargic, brief cry to interventions but minimally active on exam.

**Expected Actions:**

□ Rapid assessment of airway, breathing, and circulation
□ Check vital signs
□ Recognize abnormal vital signs
□ Obtain IV/IO access
□ Take basic history from the mother

**End Round 1.**

**Refer to expected actions and teaching points below:** [Table t8-jetem-9-4-c1]

**Table t8-jetem-9-4-c1:**

Expected	Action Teaching	Point Result
Rapid assessment of airway, breathing, and circulation	When presented with ill patient, assess early and immediately intervene if emergent situation found	This patient’s circulatory status is unstable: tachycardia, hypotension, delayed capillary refill This indicates shock
Check vital signs and recognize abnormal vital signs	Document complete set initial vitals HR for age – greater than 180 bpm is abnormal SBP for age – less than 70mmHg is abnormal	
Obtain IV/IO access for resuscitation	Try PIV first, particularly if the patient is awake. Strongly consider IO if critically ill and unable to obtain PIV, particularly if altered mental status/coma	If re-doing round, can make PIV unattainable
Take basic history from the mother	History is important to management. Focus should be on the past medical & surgical history and medications. Try to obtain information while performing other tasks. This can be achieved with appropriate role delegation.	The history provided suggests severe gastroenteritis and dehydration

#### ROUND 2: Recognition and initial management of shock

**Objectives introduced this round:**

1. Recognize/Diagnose hypovolemic shock
2. Differentiate a well-nourished child from a malnourished child
3. Assess success of resuscitation efforts
4. Parenteral fluid bolus for shock

**Prompt for team:** “You have been called to evaluate a sick infant. The patient is a 6-month male with 2 days of vomiting and diarrhea. His mother reports he is very sleepy today.”

**Pertinent history (if asked):**

History of Present Illness: The child has been having 8–10 unformed, liquid stools each day the past 2 days. A few hours ago, he started vomiting. This prompted his mother to bring him for evaluation. He does not seem interested in eating or taking from the breast or the bottle. He is sleepier today. No recent antibiotic use. Two of his older cousins have been having vomiting and diarrhea but they are 4 and 5 years old. Mother is unsure the last time she saw the child make urine explicitly.

Review of Systems:

Positive: nonbloody, nonbilious emesis, nonbloody diarrhea.

Negative: fever, night sweats, weight loss, lymphadenopathy, ecchymosis, rash, vision change, rhinorrhea, sinus pain, epistaxis, dental problems, weight change, palpitations, syncope, edema, cyanosis, orthopnea, hemoptysis, nausea, vomiting, diarrhea, blood in stool, appetite change, abdominal pain, dysuria, frequency, urgency, hematuria, joint pain or swelling, muscle pain, back pain, headache, weakness

Medications: none

Medical & Surgical History: none

FH: Two of his older cousins have been having vomiting and diarrhea

Social Hx: lives at home with mother, does not go to daycare[Table t9-jetem-9-4-c1]

**Table t9-jetem-9-4-c1:**

**Vital signs** (need to be obtained):	HR 200	BP 50/34	RR 40
Temp 36.5 °C	SpO2 98%	Weight 7 kg	

**Physical exam findings (if asked):**

**General appearance:** Patient is somnolent but arousable. Pale. Dry mucous membranes

**Initial Assessment:**

- **Airway/Breathing:** Airway open. Lungs with shallow respirations, clear bilaterally
- **Circulation:** No murmur, weak central and peripheral pulses. Capillary refill 4–5 seconds, extremities cool
- **Other:** Poor skin turgor, dry mucous membranes, eyes sunken. Abdomen soft, bowel sounds hyperactive. Lethargic, brief cry to interventions but minimally active on exam.

**Scenario Progression #1:**

After the first IV fluid bolus, vital signs are HR 195, BP 60/39, RR 40, SpO2 98% on room air

**Expected Actions:**

**The group should continue to work on skills for which they received feedback in the previous round. In addition, they should:**

□ Diagnose hypovolemic shock
□ Evaluate for malnutrition
□ Estimate weight of the child
□ Administer parenteral fluids
□ Assess group communication

**End Round 2.**

Review initial exam, reinforce importance of rapid initiation of IV fluids in shock, appropriate fluid volume for pediatric patients, and immediate reassessment after fluids. Discuss learners’ differential diagnosis and the different types of shock. Discuss how the initial management might be different if the child was malnourished.

Refer to expected actions and teaching points below:[Table t10-jetem-9-4-c1]

**Table t10-jetem-9-4-c1:**

Expected Action	Teaching Point	Result
Diagnose hypovolemic shock	A patient with large-volume diarrhea with resultant hypotension, lethargy, tachycardia, and cool skin suggests hypovolemic shock.	Patient is in shock from severe dehydration and poor oral intake.
Evaluate for malnutrition	Dehydration and malnutrition can present similarly. One should inquire to the normal appearance of the child with the caretaker. Edema, wasting, or decreased upper arm circumference are the easiest ways to identify malnutrition in the acute setting.	This child’s presentation is more suggestive of dehydration than malnutrition.
Estimate weight	An age-based or height-based system for systemic estimation of weight is indicated for appropriate pediatric weight-based dosing.	
Give parenteral fluids	The treatment of hypovolemic shock is aggressive fluid resuscitation. Rapid bolus, 20 mL/kg push over 5 min or less. Only isotonic fluids should be given as a bolus (e.g., ringer’s lactate or normal saline). If evidence of malnutrition, 10 mL/kg bolus.	20mL/kg × 6kg = 120mL initial fluid bolus
Clear communication	Has one person been designated the “team lead?” Is there closed-loop communication? When the leader gives a clear request, and second team member recites back the interpretation of the request and completion of the request. Mental modeling. The team leader shares his/her thought process for greater group understanding of the resuscitation. (e.g., “I think this patient has hypovolemic shock and so we will treat with IV fluid boluses.”	The team should each reflect their roles as members of the team and how communication can be improved.

#### ROUND 3: Laboratory and imaging studies

**Objectives introduced this round:**

1. Reassess a critically ill child after fluid bolus
2. Recognize need for continued resuscitation
3. Identify hypoglycemia as a complication of hypovolemic shock and altered mental status
4. Appropriately treat hypoglycemia in a critically ill child
5. Choose appropriate disposition for patient

**Prompt for team:** “You have been called to evaluate a sick infant. The patient is a 6-month male with two days of vomiting and diarrhea. His mother reports he is very sleepy today.”

**Pertinent history (if asked):**

History of Present Illness: The child has been having 8–10 unformed, liquid stools each day the past two days. A few hours ago, he started vomiting. This prompted his mother to bring him for evaluation. He does not seem interested in eating or taking from the breast or the bottle. He is sleepier today. No recent antibiotic use. Two of his older cousins have been having vomiting and diarrhea but they are four and five years old. Mother is unsure the last time she saw the child make urine explicitly.

Review of Systems:

Positive: nonbloody, nonbilious emesis, nonbloody diarrhea

Negative: fever, night sweats, weight loss, lymphadenopathy, ecchymosis, rash, vision change, rhinorrhea, sinus pain, epistaxis, dental problems, weight change, palpitations, syncope, edema, cyanosis, orthopnea, hemoptysis, nausea, vomiting, diarrhea, blood in stool, appetite change, abdominal pain, dysuria, frequency, urgency, hematuria, joint pain or swelling, muscle pain, back pain, headache, weakness

Medications: none

Medical & Surgical History: none

FH: Two of his older cousins have been having vomiting and diarrhea

Social Hx: lives at home with mother, does not go to daycare[Table t11-jetem-9-4-c1]

**Table t11-jetem-9-4-c1:**

**Vital signs** (need to be obtained):		HR 200	BP 50/34	RR 40	Temp 36.5 °C
SpO2 98%	Weight 7 kg				

**Physical exam findings (if asked):**

**General appearance:** Patient is somnolent but arousable. Pale. Dry mucous membranes

**Initial Assessment:**

- **Airway/Breathing:** Airway open. Lungs with shallow respirations, clear bilaterally
- **Circulation:** No murmur, weak central and peripheral pulses. Capillary refill 4–5 seconds, extremities cool
- **Other:** Poor skin turgor, dry mucous membranes, eyes sunken. Abdomen soft, bowel sounds hyperactive. Lethargic, brief cry to interventions but minimally active on exam.

**Scenario Progression #1:**

After the first IV fluid bolus, vital signs are HR 195, BP 60/39, RR 40, SpO2 98% on room air

**Scenario progression #2:**

If the team checks glucose, the result is 1.8 mmol/L (32 mg/dL).

If an appropriate dextrose bolus is given and it is rechecked, repeat will be 4.2 mmol/L (75 mg/dL).

**After glucose updated physical exam:**

AIRWAY/BREATHING: airway open, normal respirations, lungs clear bilaterally

CIRCULATION: no murmur, weak peripheral pulse, stronger central pulse. Cap refill 4 sec, extremities cool.

OTHER: more active on exam (if dextrose given), cries during interventions.

**Instructor should ad lib a discussion with the learners as though an admitting physician.**

**Expected Actions:**

**The group should continue to work on skills for which they received feedback in the previous round. In addition, they should:**

□ Recognize persistent unstable vital signs
□ Give a second IV fluid bolus
□ Check glucose
□ Administer parenteral glucose
□ Admit/Transfer the patient

**End Round 3.**

Review the initial exam, reinforce the importance of rapid initial assessment, checking vital signs and obtaining IV/IO access in a critically ill patient. Review the definition of shock and how to evaluate for signs of severe dehydration. Review initial management of shock in malnourished children compared to well-nourished children. Review the need for reassessment and search for alternate causes of lethargy (eg, hypoglycemia). Recognize the need for glucose and additional fluid administration.

Refer to expected actions and teaching points below:[Table t12-jetem-9-4-c1]

**Table t12-jetem-9-4-c1:**

Expected Action	Teaching Point	Result
Reassess after bolus	Always recheck vital signs, physical exam for perfusion (pulses, hand warmth), and respiratory status after giving a bolus to evaluate for success or complications of intervention.	Heart rate and blood pressure have improved slightly but are not normal for child’s age. There are no signs of cardiac malfunction worsened by IV fluid bolus.
Repeat fluid bolus	Administer another parenteral bolus of fluid, 20 mL/kg, being sure to reassess after each bolus. In patients with hypovolemic shock, it is not uncommon for a patient to receive a total fluid bolus of 60 mL/kg of isotonic fluid.	Vital signs, physical exam, perfusion, and mentation improve after total 40 mL/kg isotonic fluid.
Check glucose	Hypoglycemia is a potential complication of a patient with gastroenteritis and poor oral intake. Children may present with lethargy, listlessness, or may even present with seizure. <3.3 mmol/L (60 mg/dL) – 3.9 mmol/L (70 mg/dL) is often considered low. Children are typically symptomatic < 2.8 mmol/L (50 mg/dL) – 3 mmol/L (54 mg/dL).	The patient is hypoglycemic, causing his lethargy 1.8 mmol/L (32 mg/dL).
Administer parenteral dextrose	Dextrose can be administered peripherally in any concentration; however, the higher concentration of glucose (50%) may compromise a peripheral intravenous line. The “rule of 50” can be used to help remember appropriate glucose dosing for pediatric hypoglycemia: 1 mL/kg of D50 2 mL/kg of D25 5 mL/kg of D10	This patient weighs 6 kg. He could receive: 1 mL/kg of D50 × 6 kg = 6 mL 2 mL/kg of D25 × 6 kg = 12 mL 5 mL/kg of D10 × 6 kg = 30 mL
Appropriate disposition	The patient’s hemodynamic status is improving, but he will need continued hemodynamic monitoring. This might be accomplished in a pediatric critical care unit or telemetry. Hospital capability will need to be discussed with the admitting physician.	

#### ROUND 4: Total Case

If time allows, restart from the beginning of the scenario and run the full scenario without interruption.

**END Round 4.**

**END OF RCDP Session.**

**Final debriefing and feedback**

- Praise learners for tasks accomplished well
- Provide areas for continued improvement

Management of hypoglycemia: “Rule of 50”

**50 ÷ percent dextrose = mL/kg needed for dose**

Examples:

50 ÷ 50% dextrose = 1 mL/kg dose
50 ÷ 25% dextrose = 2 mL/kg dose
50 ÷ 10% dextrose = 5 mL/kg dose
50 ÷ 5% dextrose = 10 mL/kg dose

Can give D50 through IO. Should not give more than D10 PIV. Can mix D10 from D50 by mixing four parts NS or sterile water with one-part D50.[Fig f2-jetem-9-4-c1]

### Appendix H Small Bowel Obstruction Lecture Synopsis

**Title:** Pediatric Bowel Obstruction Lecture

**Target Audience:** generalist healthcare providers in low- and middle-income countries

**Educational Methods:** PowerPoint didactic lecture

**Time required for implementation:** about 60 minutes

**Equipment/Environment/Personnel:**

- *A large room (with a capacity of at least 50 people) with multiple tables and ample floor space, or multiple rooms if available*
- *A computer with PowerPoint capability and projector setup*
- *Personnel needed: one lecturer*

**Learning Objectives:**

The learner will be able to:

1. Evaluate the child presenting with vomiting and abdominal pain
2. Give a differential diagnosis for acute bowel obstruction in a child
3. Determine if a child has a dangerous cause of vomiting
4. Medically manage a child with acute bowel obstruction
5. Order appropriate imaging and refer children requiring further management

**Lecture Script**:[Table t13-jetem-9-4-c1]

**Table t13-jetem-9-4-c1:**

Slide 1	Title slide.
Slide 2	Introduce the acronym key.
Slide 3	The instructor should read the case aloud or have one of the learners in the class read the case. The instructor should ask the learners to ponder the questions asked.
Slide 4	The instructor should read the case aloud or have one of the learners in the class read the case. The instructor should ask the learners to ponder the questions asked.
Slide 5	Review objectives of the lecture.
Slide 6	Subtitle slide.
Slide 7	Describe the signs and symptoms of bowel obstruction that can be interpreted from the history and physical exam. If it is a more advanced class, the instructor can have the learners call out what they believe are signs and symptoms of bowel obstruction.
Slide 8	The instructor should review the details about the complaint of vomiting that would be associated with small bowel obstruction.
Slide 9	Review the characteristics and types of bowel pain.
Slide 10	Laboratory studies cannot in of themselves diagnose a bowel obstruction. Hypochloremia or hypochloremia may result from vomiting. Elevated serum lactate may result from vomiting but could result from bowel ischemia with or without obstruction. White blood cell counts may be elevated for a myriad of reasons in any patient presenting to the emergency setting. Lab studies may be useful to risk stratify for likelihood or severity of illness and may also be useful for preparation for operative intervention.
Slide 11	Imaging is necessary for the diagnosis of bowel obstructions. Unlike adult patients who often receive computed tomography (CT scans) the diagnosis of pediatric bowel obstructions are predominantly made by: X-rays (radiographs) – theoretically, more than one view may help confirm presence of disease and identify artifact. Ultrasound is becoming a mainstay for pediatric diagnoses with great specificity without exposure to ionizing radiation. However, the utility of ultrasound is usually limited by the proficiency of the ultrasonographer. Barium studies help augment plain film radiographs or fluoroscopy, but, often require the assistance of a radiologist for optimal timing of the contrast relative to the study. The preferred imaging study will depend on the leading differential diagnosis regarding the cause of the obstruction which will be discussed in the following slides. Ikenberry SO, Jue TL, Anderson M, et al. Management of ingested foreign bodies and food impactions. *Gastrointest. Endosc*. 2011;73 (6): 1085–91 Uyemura MC. Foreign body ingestion in children. *Am Fam Physician*. 2005;72 (2): 287–91. Kay M, Wyllie R. Pediatric foreign bodies and their management. Curr *Gastroenterol Rep*. 2005;7 (3): 212–8. Hull NC, Kim HHR, Phillips GS, Lee EY. Neonatal and pediatric bowel obstruction. *Radiol Clin North Am*. 2022;60(1):131–148. Carroll AG, Kavanagh RG, Ni Leidhin C, Cullinan NM, Lavelle LP, Malone DE. Comparative effectiveness of imaging modalities for the diagnosis of intestinal obstruction in neonates and infants: *Acad Radiol*. 2016;23(5):559–568. Tsitsiou Y, Calle-Toro JS, Zouvani A, Andronikou S. Diagnostic decision-making tool for imaging term neonatal bowel obstruction. *Clin Radiol*. 2021 Mar;76(3):163–171. Hryhorczuk A, Lee EY, Eisenberg RL. Bowel obstructions in older children. *AJR Am J Roentgenol*. 2013;201(1). Slide 12 Subtitle slide.
Slide 13	The peak incidence of ingested foreign bodies (FB) is between six months and six years, during which time children are far more likely to use their mouths to explore the world. Half are asymptomatic and will pass the foreign body through the GI system without incident. Coins are the common FB ingested in the USA, but internationally, food-related products (i.e., bones) occur with more frequency. X-rays identify about 65% of radiodense FBs; this is important when interpreting images especially if a plastic or other radiolucent FB ingestion is suspected.
Slide 14	A foreign body can cause partial or complete GI obstruction at any level of the GI tract. However, the most common location of obstruction is within the esophagus at the thoracic inlet of a child. Once a foreign body has reached the stomach, it will generally pass without incident. However, there are important red flags which could indicate a more urgent foreign body removal due to their increased risk for bowel perforation: Sharp objects Caustic items (eg, pills, button batteries) Strong magnets can cause pressure necrosis when more than one is ingested Endoscopic removal is the mainstay for foreign body removal when indicated.
Slide 15	Subtitle slide.
Slide 16	An overview of pyloric stenosis. The etiology of infantile hypertrophic pyloric stenosis is unknown and is probably multifactorial (genetic and environmental factors). Classical History: The infant with pyloric stenosis has nonbilious vomiting or regurgitation, which may become projectile (i.e., forceful away from the body compared to spitting up which is more dribbling from the mouth or nose). The infant is often hungry after vomiting. Emesis may be intermittent initially or occur after each feeding. Emesis should not be bilious as the obstruction is proximal to the common bile duct (proximal to the ligament of Trietz). As the obstruction becomes more severe, the infant begins to show signs of dehydration and malnutrition, such as poor weight gain, weight loss, malnourishment, decreased urinary output, lethargy, and could ultimately lead to shock.
Slide 17	Evaluating pyloric stenosis. Classic Physical Exam: Palpation of the abdomen might yield a firm, nontender, mobile 1–2cm olive-shaped mass at the right upper quadrant (RUQ). Signs of dehydration: depressed fontanelles, dry mucosa, decreased tearing, poor skin turgor, lethargy. Classic signs/symptoms that we discuss from textbooks are less common in present day due to early detection using ultrasound. However, nonbilious, nonbloody, forceful emesis is relatively consistent. Maheshwari P, Abograra A, Shamam O. Sonographic evaluation of gastrointestinal obstruction in infants: a pictorial essay. *J Pediatr Surg*. 2009;44(10):2037–2042. doi:10.1016/j.jpedsurg.2009.05.019 Gerrie SK, Navarro OM. Imaging features of neonatal bowel obstruction. [published correction appears in *Radiographics*. 2023 Sep;43(9):e239010]. *Radiographics*. 2023;43(8):e230035.
Slide 18	Ultrasonography is the first line imaging modality of choice when evaluating a child for infantile hypertrophic pyloric stenosis. It is both highly sensitive (90–99%) and specific (97–100%) in the hands of a *qualified sonographer*. The pylorus should be viewed in both longitudinal and transverse planes. The sonographic hallmark of infantile hypertrophic pyloric stenosis is a thickened pyloric muscle. Maheshwari P, Abograra A, Shamam O. Sonographic evaluation of gastrointestinal obstruction in infants: a pictorial essay. *J Pediatr Surg*. 2009;44(10):2037–2042. doi:10.1016/j.jpedsurg.2009.05.019
Slide 19	The **antral nipple sign** refers to redundant pyloric mucosa protruding into the gastric antrum. The **target sign (donut sign)** describes indentation of the pylorus into the fluid-filled antrum.
Slide 20	**String sign (double track sign)** - a string of contrast material coursing through the mucosal intersticesor by several linear tracts of contrast material separated by the intervening mucosa. The **mushroom sign** (**umbrella sign**) the impression made by the hypertrophic pylorus on the duodenal cap.
Slide 21	X-ray can identify air in the stomach and intestines but is often not sensitive or specific without contrast.
Slide 22	Overview of pyloric stenosis management. If the child is ill-appearing, clinically dehydrated, or has significant electrolyte abnormality, resuscitation with intravenous fluids is the first intervention which should take place. Medical resuscitation is necessary prior to operative intervention. There are some cases where gastroenterologists or surgeons may opt for dilatation procedures of the pylorus; however, this procedure has an higher likelihood of failure. Pyloromyotomy is currently the mainstay definitive intervention. It can be performed open or by laparotomy which would be determined by the surgeon performing the procedure. Bissonnette B, Sullivan PJ. Pyloric stenosis. *Can J Anaesth*. 1991;38(5):668–676. doi:10.1007/BF03008206
Slide 23	Subtitle slide.
Slide 24	Discuss the three kinds of gut volvulus. However, midgut volvulus is the common pediatric type. The others involving the large bowel and are more common in adults. Midgut volvulus – a complication of malrotated bowel about the superior mesenteric vessel bundle. Notably, the patient will have ***bilious vomiting***.
Slide 25	Malrotation of the midgut is anormal twisting around the superior mesenteric vessels which could ultimately result in infarction of the gut. The malrotated bowel itself does not cause any significant problems, but the narrow axis by which the gut is suspended can cause bowel obstruction during peristalsis or from obstruction of the lumen, or obstruction of the flow of the mesenteric vessels. Most patients with midgut malrotation develop volvulus (obstruction) within the first week of life. Bilious vomiting is the initial symptom. However, obstruction and strangulation can occur at any age. Coste AH, Anand S, Nada H, Ahmad H. Midgut volvulus. In: *StatPearls*. Treasure Island (FL): StatPearls Publishing; September 12, 2022. https://www.ncbi.nlm.nih.gov/books/NBK441962/
Slide 26	X-ray of the abdomen is a great screen for malrotation. One can look for the listed findings. Though an XR without contrast can show items like the “double-bubble” sign, an XR with contrast will give more detail to suggesting the diagnosis. However, a doppler US of the mesenteric vessels will better describe whether there is resultant volvulus and compromise of the mesenteric vessels. A final diagnosis would often be made by computer tomography or magnetic resonance imaging. Coste AH, Anand S, Nada H, Ahmad H. Midgut volvulus. In: *StatPearls*. Treasure Island (FL): StatPearls Publishing; September 12, 2022. https://www.ncbi.nlm.nih.gov/books/NBK441962/
Slide 27	XR plain film (without contrast) may show: **Double Bubble** is distention of the proximal duodenum and stomach decreased gas or gasless distal abdomen stereotypically seen with duodenal atresia as well. **Corkscrew sign** is a spiral appearance of the distal duodenum and proximal jejunum; loops twist on a shortened bowel mesentery → corkscrew appearance.
Slide 28	Discuss midgut volvulus ultrasounds. **Whirlpool sign** – where the structure twists upon itself; swirling of the mesentery and the superior mesenteric vessels; swirl is clockwise (on ultrasound) and counter-clockwise on CT.
Slide 29	Medical resuscitation of fluid or electrolyte deficiencies is the first indicated intervention. Early surgical consultation will often result in operative repair: Ladd procedure. Importantly, the procedure does not restore normal anatomic positioning; rather, it removes the obstructive bands causative of volvulus.
Slide 30	Subtitle slide.
Slide 31	Introduce the signs of intussusception: Fontanelles, dry mucosa, decreased tearing, poor skin turgor, lethargy. Sudden onset and resolution colicky pain. Often draw up both legs. Can appear healthy in between paroxysms of pain. Can become progressively irritable and lethargic with progression of disease. Lethargy or altered mental status alone is the presenting complaint of many children diagnosed with bowel intussusception. Stools can become bloody and mucoid (currant jelly). Jain S, Haydel MJ. Child intussusception. In: *StatPearls*. Treasure Island (FL): StatPearls Publishing; April 10, 2023. https://www.ncbi.nlm.nih.gov/books/NBK431078/
Slide 32	Show the incidence of intussusception worldwide.
Slide 33	Discuss the rotavirus vaccine and intussusception. There was a 5-year retrospective study conducted in Panama that found an average rate of one case per 3300 infants younger than one year of age, with substantial yearly variations ranging from 1:2500 to 1:5000. RotaTeq™ (RV5; Merck & Co. Inc., USA) and Rotarix™ (RV1, GlaxoSmithKline, Belgium) vaccines, developed to prevent rotavirus diarrhea in children under five years old, were both introduced into many national immunization programs in 2006. Many countries in Latin America and the Caribbean have included either RV5 or RV1 in their routine childhood vaccination programs. Findings are around an average rate of one case per 3300 infants younger than one year of age, with substantial yearly variations ranging from 1:2500 to 1:5000. The benefits from rotavirus vaccination greatly exceed the risk of intussusception, especially in developing regions such as Latin America.
Slide 34	Ultrasound is currently generally the preferred means of diagnosis of intussusception. Discuss the ultrasound findings of intussusception. Ultrasound is often the first choice of imaging for diagnosis of intussusception. Sensitivity depends on skill of ultrasonographer. Ultrasound is highly specific and sensitive. The **target sign** is two rings of low echogenicity separated by a hyperechoic ring. Hryhorczuk AL, Strouse PJ. Validation of US as a first-line diagnostic test for assessment of pediatric ileocolic intussusception. *Pediatr Radiol*. 2009;39(10):1075–9. Li XZ, Wang H, Song J, Liu Y, Lin YQ, Sun ZX. Ultrasonographic diagnosis of intussusception in children: a systematic review and meta-analysis. *J Ultrasound Med*. 2021;40(6):1077–1084.
Slide 35	Discuss the primary means of diagnosis by region of intussusception. Data from Central and South America were from one prospective research study that might not reflect clinical practice under routine conditions.
Slide 36	Barium or air enemas can be both therapeutic and diagnostic. Decision of type of enema is usually made in conjunction with radiologist and/or surgeon.
Slide 37	Describe the procedure and types of enemas.
Slide 38	Operative management for enemas is dependent on local practice. In some regions, it is the first line. In other areas, surgical intervention is reserved for failed enemas. Ileo-cecal intussusception requires intervention of some sort. Ileo-ileal intussusception does not require intervention if it is small (<2.3 cm) because it will likely resolve by itself.
Slide 39	Discuss the treatment of intussusception by region.
Slide 40	Subtitle slide.
Slide 41	Discuss the signs and symptoms of small bowel adhesions which cause small bowel obstructions. Often in patients who have an history of some abdominal or pelvic operation. Pain is often vaguely described or cramping in nature. Anorexia, nausea, vomiting. Decreased bowel movements. Symptoms can spontaneously resolve especially if causing a partial bowel obstruction.
Slide 42	Most pediatric small bowel obstructions can be identified on plain film x-ray. Contrast may be added for more information about the type of obstruction. Chotai, P. Pediatric small bowel obstruction workup. Medscape. March 12, 2024. https://emedicine.medscape.com/article/930411-workup#c7
Slide 43	Ultrasound is becoming more prevalent in the diagnosis of bowel obstruction in pediatric patients. However, sensitivity is dependent on user proficiency. Chotai, P. Pediatric small bowel obstruction workup. Medscape. 2024, March 12. https://emedicine.medscape.com/article/930411-workup#c7
Slide 44	Management of small bowel obstructions due to adhesions is generally supportive with bowel rest. Identify and optimize any hemodynamic insufficiencies or electrolyte anomalies. Nasogastric tube (NG) use will be discussed further on the next slide If the patient is septic or critically ill, begin broad spectrum antibiotics and discuss operative intervention with a surgeon.
Slide 45	Nasogastric tubes were once placed routinely for small bowel obstructions. However, recent literature has shown that they may not be as useful routinely and may actually cause more complications if placed routinely.
Slide 46	Subtitle slide.
Slide 47	Discuss abdominal hernias. Abdominal hernias are distinguished primarily based on location and content. Seventy-five to eighty per cent of all hernias are inguinal. Physical exam is often more helpful than imaging studies. Hebra A. Pediatric hernias. Medscape. 2024, February 16. https://emedicine.medscape.com/article/932680-overview
Slide 48	Discuss congenital hernias. Failed closure of the umbilical ring usually resolves or is repaired at two to four years old. Umbilical hernias commonly contain fat, mesentery, or small and/or large bowel. Troullioud Lucas AG, Bamarni S, Panda SK, et al. Pediatric Umbilical Hernia. [Updated 2023 Nov 18]. In: StatPearls [Internet]. Treasure Island (FL): StatPearls Publishing; 2024 Jan-. Available from: https://www.ncbi.nlm.nih.gov/books/NBK459294/
Slide 49	Present the complications of a hernia. It is important to be able to accurately describe a hernia. An incarcerated hernia is simply stuck – like a person in prison (incarcerated). It is difficult to reduce and may be painful and cause nausea, vomiting, or decreased bowel movements because of the functional bowel obstruction. A strangulated hernia is a surgical emergency because it is dying bowel causing systemic signs in addition to all of the signs of incarceration.
Slide 50	Most incarcerated hernias can be reduced in the emergency setting with proper analgesia and proper technique by manual reduction using a slow, constant pressure. If it remains a difficult task or the patient is in significant pain, or systemic signs are present, an early surgical consultation is warranted.
Slide 51	Revisit Case 1. Would anyone consider this case differently after this talk?
Slide 52	Continue revisiting Case 1 with answers.
Slide 53	Revisit Case 2. Would anyone consider this case differently after this talk?
Slide 54	Continue revisiting Case 2 with answers.

**References:**

- Hebra A. Pediatric hernias. Medscape. 2024, February 16. https://emedicine.medscape.com/article/932680-overview
- Bissonnette B, Sullivan PJ. Pyloric stenosis. *Can J Anaesth*. 1991;38(5):668–676. doi:10.1007/BF03008206
- Shaoul R, Enav B, Steiner Z, Mogilner J, Jaffe M. Clinical presentation of pyloric stenosis: the change is in our hands. *Isr Med Assoc J.* 2004;6(3):134–137.
- Carroll AG, Kavanagh RG, Ni Leidhin C, Cullinan NM, Lavelle LP, Malone DE. Comparative effectiveness of imaging modalities for the diagnosis of intestinal obstruction in neonates and infants. *Acad. Radiol*. 2016;23(5):559–568.
- Chotai, P. Pediatric small bowel obstruction workup. Medscape. March 12, 2024. https://emedicine.medscape.com/article/930411-workup#c7
- Coste AH, Anand S, Nada H, Ahmad H. Midgut volvulus. In: *StatPearls*. Treasure Island (FL): StatPearls Publishing; September 12, 2022. https://www.ncbi.nlm.nih.gov/books/NBK441962/
- Langer JC. Intestinal Rotation Abnormalities and Midgut Volvulus. *Surgical Clinics of North America*. 2017;97(1):147–159. doi:10.1016/j.suc.2016.08.011.
- Gerrie SK, Navarro OM. Imaging Features of neonatal bowel obstruction [published correction appears in *Radiographics*. 2023 Sep;43(9):e239010]. *Radiographics*. 2023;43(8):e230035.
- Hryhorczuk A, Lee EY, Eisenberg RL. Bowel obstructions in older children. *AJR Am J Roentgenol*. 2013;201(1).
- Hryhorczuk AL, Strouse PJ. Validation of US as a first-line diagnostic test for assessment of pediatric ileocolic intussusception. *Pediatr Radiol*. 2009;39(10):1075–9.
- Hull NC, Kim HH, Phillips GS, Lee EY. Neonatal and pediatric bowel obstruction. *Radiol Clin North Am*. 2022;60(1):131–148.
- Ikenberry SO, Jue TL, Anderson M, et al. Management of ingested foreign bodies and food impactions. *Gastrointest. Endosc*. 2011;73 (6): 1085–91.
- Jain S, Haydel MJ. Child intussusception. In: *StatPearls*. Treasure Island (FL): StatPearls Publishing; April 10, 2023. https://www.ncbi.nlm.nih.gov/books/NBK431078/
- Jiang J, Jiang B, Parashar U, Nguyen T, Bines J, Patel MM. Childhood intussusception: a literature review. *PLoS ONE*. 2013;8(7). doi:10.1371/journal.pone.0068482.
- Kay M, Wyllie R. Pediatric foreign bodies and their management. *Curr Gastroenterol Rep*. 2005;7 (3): 212–8.
- Li XZ, Wang H, Song J, Liu Y, Lin YQ, Sun ZX. Ultrasonographic diagnosis of intussusception in children: a systematic review and meta-analysis. *J Ultrasound Med*. 2021;40(6):1077–1084.
- Maheshwari P, Abograra A, Shamam O. Sonographic evaluation of gastrointestinal obstruction in infants: a pictorial essay. *J Pediatr Surg*. 2009;44(10):2037–2042. doi:10.1016/j.jpedsurg.2009.05.019
- Troullioud Lucas AG, Bamarni S, Panda SK, et al. Pediatric Umbilical Hernia. [Updated 2023 Nov 18]. In: StatPearls [Internet]. Treasure Island (FL): StatPearls Publishing; Jan 2024. Available from: https://www.ncbi.nlm.nih.gov/books/NBK459294
- Tsitsiou Y, Calle-Toro JS, Zouvani A, Andronikou S. Diagnostic decision-making tool for imaging term neonatal bowel obstruction. *Clin Radiol*. 2021 Mar;76(3):163–171.
- Uyemura MC. Foreign body ingestion in children. *Am Fam Physician*. 2005;72 (2): 287–91.

### Appendix I Small Bowel Obstruction Lecture

Please see associated PowerPoint file

### Appendix J Small Bowel Obstruction Simulation Case

**Case Title:** Pediatric Bowel Obstruction Rapid Cycle Deliberate Practice (RCDP) Case

**Time required for implementation:** ~ 45 minutes for multiple rounds of RCDP

**Recommended number of learners per instructor/case:**

- One simulation instructor/debriefing facilitator per group of three to six learners
  ○ This person should be well-versed in the medical theory taught by the simulations presented
  ○ This person should have experience with pediatric resuscitations and general pediatric care
- One actors/assistant per group of three to six learners

**Learner responsible content:** Appendix C. Small Bowel Obstruction Lecture

**Objectives:**

By the end of the session, learners should be able to:

Cognitive:

1. Recognize an acute surgical abdomen (bowel obstruction)
2. Recognize septic shock
3. Understand the need for rapid treatment and reassessment
4. Consider various causes of bowel obstruction

Technical:

1. Perform a rapid initial assessment
2. Perform peripheral IV line or IO placement

Behavioral:

1. Communicate clear leadership roles with delegation of roles for better teamwork
2. Perform early interventions for septic shock/bowel obstruction
3. Administer appropriate antibiotics, fluid resuscitation +/− vasopressors for a patient with septic shock secondary to bowel obstruction
4. Consult appropriate specialist and communicate emergent nature of situation

**Abbreviations** [Table t14-jetem-9-4-c1]

**Table t14-jetem-9-4-c1:**

AED =	automatic external defibrillator
BP =	blood pressure
Bpm =	beats per minute
BVM =	bag valve mask
CPR =	cardiopulmonary resuscitation
EKG =	electrocardiogram
EMS =	emergency medical services
FH =	family history
GCS =	Glasgow Coma Scale
HR =	heart rate
Hx =	history
IO =	intraosseous
IV =	intravenous
LR =	Ringer’s lactate
NS =	normal saline
O2 =	oxygen
PEA =	pulseless electrical activity
Pt =	patient
RCDP =	rapid cycle deliberate practice
ROS =	review of symptoms
RR =	respiratory rate
RUQ =	right upper quadrant
SBP =	systolic blood pressure
SIRS =	systemic inflammatory response syndrome
O_2_Sat =	oxygen saturation
T =	temperature

**References:**

1. Jain S, Haydel MJ. Child intussusception. In: *StatPearls*. Treasure Island (FL): StatPearls Publishing; April 10, 2023.

**Case Title:** Pediatric Small Bowel Obstruction RCDP Practice Case

**Case description & diagnosis (short synopsis):**

John is brought into the emergency department hastily. The patient has unstable vital signs. He requires rapid resuscitation with fluids, antibiotics, +/− vasopressors and surgical intervention. He will deteriorate if not treated expeditiously. If appropriate and time sensitive therapy occurs, he will have improvement of his vital signs and general appearance. If inappropriate or delayed interventions, the patient will decompensate to pulseless electrical activity (PEA) arrest due to septic shock from perforated bowel obstruction.

**Equipment or props needed:**

Setup for All Rounds:

- Room configuration: typical emergency bed/stretcher (or table)
- For each group of 3three to five learners, one equipment setup includes the following:
  ○ A low-fidelity full-body simulation mannequin. If available, higher-fidelity mannequins can be used (we used a MegaCode Kid, and Laerdal ALS Baby mannequins)
  ○ An intravenous arm task trainer (if the mannequin is not equipped)
  ○ Lower extremity capable of intraosseous (IO) insertion (if mannequin is not equipped)
  ○ A medical resuscitation setup including the standard resuscitation equipment available in the emergency department (intravenous line starter kits, intravenous fluids, IO drill, IO needles, medical tape, bag valve mask, mock medications, etc.)
- Personnel:
  ○ Simulation instructor/debriefer
  ○ Actors: patient’s grandmother (Sheila), inpatient pediatrician
  ○ Demonstration items needed for debriefing: same as equipment

**Ideal Scenario Flow:**

John is brought to the emergency bed. The learners are expected to obtain a full set of vital signs and recognize that his vital signs are unstable. They should also perform a rapid initial assessment. The grandmother will reveal that the patient’s history suggests intussusception, and the concern currently is intussusception with gut ischemia. Participants should be concerned for hypovolemic shock/septic shock, and they should give supplemental O2 and obtain vascular access for labs and medication administration. The learners should give an appropriate weight-based dose of isotonic fluids rapidly and initiate broad-spectrum antibiotics early. They can be presented the laboratory studies and imaging at any point after the learners have ordered them. They will also need to interpret the laboratory studies that they obtained. Learners should be concerned for a complicated small bowel obstruction with perforation as the source of septic shock by history, physical exam, and radiograph. The patient needs an emergency surgical consultation for operative intervention.

After their interventions, they should reassess the patient including a reassessment of vital signs.

If inappropriate or delayed interventions, she will decompensate to Pulseless Electrical Activity (PEA) arrest due to metabolic acidosis and sepsis.

The participants should end the case with a handoff to the surgeon – summarizing the case using proper medical terminology.

**Critical Actions:**

Round 1:

□ Rapid assessment of airway, breathing, and circulation
□ Check vital signs and recognize abnormal vital signs
□ Obtain IV/IO access
□ Take basic history from the patent
□ Recognize septic shock (and etiology)

Round 2:

□ Give rapid fluids
□ Give antibiotics early
□ Reassess after IV fluid boluses and antibiotics
□ Recognize continued hypotension

Round 3:

□ Obtain and interpret laboratory studies
□ Interpret x-ray abdomen
□ Interpret the intussusception identified on ultrasound as the cause of bowel ischemia and septic shock
□ Surgical consultation

**Expected Endpoint of the Scenario:** maximum 10-minute time limit per round, but facilitator can stop at any point during scenario

**Possible distractors within scenario**: high acuity patient

**Optional Challenges for Higher Level Learners**:

- Airway intubation due to severe sepsis
- Considering other types of shock
- PEA arrest due to septic shock

**Roles of Participants/Trainees:** usual roles within the emergency department

**Roles of Actors:**

Grandmother: provide medical history, primary caretaker of patient

Inpatient Physician, Surgeon (by instructor voice): take handoff for transition of care from learners

**Anticipated Management Errors:**

*Failure to identify bowel obstruction causative of septic shock: Because this is a low-fidelity simulation and the patient is not interacting directly, learners may not recognize that the patient has abdominal pain and an acute abdomen causative of sepsis. It is useful for the instructors to clearly indicate that there is an acute change in vital signs and that the patient has deterioration of vital signs even if the provider does not check. Similarly, it is important for the instructors to indicate abdominal pain so that the learners do not simply presume the patient is having hypovolemic shock due to miscellaneous viral gastrointestinal illness.*

**Chief complaint:** “being sleepy”[Table t15-jetem-9-4-c1]

**Table t15-jetem-9-4-c1:**

**Initial Vitals for all rounds:**		HR 150 bpm	BP 70/50 mmHg	RR 20 breaths/min
Temp 38 °C	SpO2 95%	Wt: 14 kg		

**Initial Physical Exam for all rounds:**

**General appearance:** Patient is somnolent but arousable. Pale. Dry mucous membranes

**Initial Assessment:**

- **Airway/Breathing:** Airway open. Moans and opens eyes in response to voice. Lungs clear to auscultation bilaterally.
- **Circulation:** Tachycardic (HR 150 bpm). No murmur, weak peripheral pulses. Capillary refill 4–5 seconds, extremities cool to touch.
- **Mental Status:** Somnolent but arousable to voice, non-focal.

#### ROUND 1: Initial Assessment of Patient

**Objectives introduced this round**:

1. Communicate clear leadership roles with delegation of roles
2. Perform a rapid initial assessment
3. Perform peripheral IV line or IO placement

**Prompt for team:** “A 2-year-old boy presents to the A&E with complaint of ‘being sleepy’ per his grandmother.”

**Pertinent history (if asked):**

History of Present Illness: The child has not been himself today. Last night he was not eating much, and today has not eaten at all. Occasionally he had been complaining his “tummy hurts” when he was awake enough to talk (he’s mostly been sleeping). No sick contacts. No recent illness. Last night, his grandmother gave him paracetamol (per recommended dosing on the bottle) because he felt feverish. He does not have nausea or vomiting. Stools are “darker” than normal and are softer in texture. Has not been as active as usual for the past day. Last urination five hours ago was dark.

ROS:

Positive: fever, fatigue, abdominal pain, appetite change, change in stool.

Negative: night sweats, weight loss, lymphadenopathy, ecchymosis, rash, edema, cyanosis, orthopnea, trauma, headache, neck pain, cough.

Medications: paracetamol

Medical & Surgical Hx: none

Family History (FH): Mother has asthma, Grandmother has type 2 diabetes.

Social Hx: Lives at home with his grandmother and brother.[Table t16-jetem-9-4-c1]

**Table t16-jetem-9-4-c1:**

**Vital signs** (need to be obtained):	HR 150 bpm	BP 70/50 mmHg	RR 20 breaths/min
Temp 38 °C	SpO2 95%	Wt: 14 kg	

**Physical exam findings (if asked):**

**Initial Assessment:**

- **Airway/Breathing:** Airway open. Moans and opens eyes in response to voice. Lungs clear to auscultation bilaterally.
- **Circulation:** Tachycardic (HR 150 bpm). No murmur, weak peripheral pulses. Capillary refill 4–5 seconds, extremities cool to touch.
- **Abdominal:** nondistended, high pitched bowel sounds, guarding (involuntary) diffusely, mass felt in RUQ, if diaper examined see “currant jelly” stool.
- **Neuro:** Somnolent but arousable to voice, otherwise non-focal.
- **Other:** Somnolent but arousable. Pale. Dry mucous membranes. No trauma.

**Expected Actions:**

□ Rapid assessment of airway, breathing, and circulation
□ Check vital signs and recognize abnormal vital signs
□ Obtain IV/IO access
□ Take basic history from the patent
□ Recognize septic shock (and etiology)

**End Round 1.**

**Refer to expected actions and teaching points below:** [Table t17-jetem-9-4-c1]

**Table t17-jetem-9-4-c1:**

Expected Action	Teaching Point	Result
Rapid assessment of airway, breathing, and circulation	When presented with ill patient, assess early, and immediately intervene if emergent situation found	This patient’s circulatory status is unstable: tachycardia, hypotension, delayed capillary refill
Check vital signs and recognize abnormal vital signs	Document initial vitals HR for age – greater than 130bpm is abnormal SBP for age – less than 75mmHg is abnormal	
Obtain IV/IO access for resuscitation	Try PIV first, particularly if the patient is awake. Strongly consider IO if critically ill and unable to obtain PIV, particularly if altered mental status/coma.	If re-doing round, can make PIV unattainable: those who are expected to place an IO may practice. Those who are not, can consult someone who is proficient.
Take basic history from the patient	History is important to management and should not be neglected even if the child is critically ill. This will guide management. Try to obtain information while performing other tasks.	The history provided suggests an acute abdominal process. The intermittent nature of the abdominal pain and the changes of the quality of stool suggest complicated intussusception
Recognize septic shock (and etiology)	The patient meets SIRS criteria with likely intraabdominal source for septic shock	

#### ROUND 2: Recognition and Initial Management of Shock

**Objectives introduced this round:**

1. Recognize an acute surgical abdomen (bowel obstruction)
2. Recognize septic shock
3. Perform early interventions for septic shock/bowel obstruction
4. Understand the need for rapid treatment and reassessment
5. Administer appropriate antibiotics, fluid resuscitation +/− vasopressors

**Restart scenario from beginning.**

**Prompt for team:** “A 2-year-old boy presents to the A&E with complaint of ‘being sleepy’ per his grandmother.”

**Pertinent history (if asked):**

History of Present Illness: The child has not been himself today. Last night he was not eating much, and today has not eaten at all. Occasionally he has been complaining his “tummy hurts” when he was awake enough to talk (he’s mostly been sleeping). No sick contacts. No recent illness. Last night, his grandmother gave him paracetamol (per recommended dosing on the bottle) because he felt feverish. He does not have nausea or vomiting. Stools are “darker” than normal and are softer in texture. Has not been as active as usual for the past day. Last urination five hours ago was dark.

ROS:

Positive: fever, fatigue, abdominal pain, appetite change, change in stool.

Negative: night sweats, weight loss, lymphadenopathy, ecchymosis, rash, edema, cyanosis, orthopnea, trauma, headache, neck pain, cough.

Medications: paracetamol

Medical & Surgical Hx: none

FH: Mother has asthma, Grandmother has type 2 diabetes.

Social Hx: Lives at home with his grandmother and brother.[Table t18-jetem-9-4-c1]

**Table t18-jetem-9-4-c1:**

**Vital signs** (need to be obtained):		HR 150 bpm	BP 70/50 mmHg	RR 20 breaths/min
Temp 38°C	SpO2 95%	Wt: 14 kg		

**Physical exam findings (if asked):**

- **Airway/Breathing:** Airway open. Moans and opens eyes in response to voice. Lungs clear to auscultation bilaterally.
- **Circulation:** Tachycardic (HR 150 bpm). No murmur, weak peripheral pulses. Capillary refill 4–5 seconds, extremities cool to touch.
- **Abdominal:** nondistended, high pitched bowel sounds, guarding (involuntary) diffusely, mass felt in RUQ, if diaper examined see “currant jelly” stool.
- **Neuro:** Somnolent but arousable to voice, otherwise non-focal.
- **Other:** Somnolent but arousable. Pale. Dry mucous membranes. No trauma

**Expected actions after verbal prompt:**

□ Give rapid fluids
□ Give antibiotics early
□ Reassess after IV fluid boluses and antibiotics
□ Recognize continued hypotension

**END Round 2.**

Review initial exam, reinforce importance of rapid initiation of IVF in shock, appropriate fluid volume for pediatric patients, and immediate reassessment after fluids. Discuss learners’ differential diagnosis.

Refer to expected actions and teaching points below:[Table t19-jetem-9-4-c1]

**Table t19-jetem-9-4-c1:**

Expected Action	Teaching Point	Result
Recognize septic shock (& etiology)	In a hypotensive, tachycardic patient with fever, strongly consider septic shock	The history provided with shock would suggest a complicated abdominal process, such as ischemic bowel due to bowel obstruction due to intussusception
Give rapid fluids	The patient should receive up to two isotonic intravenous boluses (20 cc/kg) with a reassessment of the patient’s hemodynamic status after each bolus	20 cc/kg → SBP 75mmHg 40 cc/kg → SBP 80 mmHg
Give antibiotics early	Necessary intervention for treating septic shock The presumed source for this patient is ischemic bowel from small bowel obstruction. Antibiotics should cover against a broad spectrum of pathogens but especially gram negative bacteria	One recommendation: Ceftriaxone (25–50 mg/kg q24h) and metronidazole (15 mg/kg, then 7.5 mg/kg q12h) Improved SBP to 80mmHg
Reassess after IV fluid boluses and antibiotics	Always recheck vitals, perfusion, respiratory status, and mental status after performing interventions in a critically ill patient	Improved vital signs, but not within normal limits
Recognize continued hypotension	Recheck vitals after every major intervention during resuscitation Consider vasopressor supports	Local vasopressor options vary, and nursing practices regarding routes of administration also vary PIVs can be used temporarily for vasopressor administration IOs are a rapid means of vasopressor administration

#### ROUND 3: Laboratory and Imaging Studies

**Objectives introduced this round:**

1. Consider various causes of bowel obstruction
2. Consult appropriate specialist and communicate emergent nature of situation
3. Choose appropriate disposition for patient

**Restart from the beginning of the scenario.**

**Prompt for team:** “A 2-year-old boy presents to the A&E with complaint of ‘being sleepy’ per his grandmother.”

**Pertinent history (if asked):**

History of Present Illness: The child has not been himself today. Last night he was not eating much, and today has not eaten at all. Occasionally he has been complaining his “tummy hurts” when he was awake enough to talk (he’s mostly been sleeping). No sick contacts. No recent illness. Last night, his grandmother gave him paracetamol (per recommended dosing on the bottle) because he felt feverish. He does not have nausea or vomiting. Stools are “darker” than normal and are softer in texture. Has not been as active as usual for the past day. Last urination five hours ago was dark.

ROS:

Positive: fever, fatigue, abdominal pain, appetite change, change in stool.

Negative: night sweats, weight loss, lymphadenopathy, ecchymosis, rash, edema, cyanosis, orthopnea, trauma, headache, neck pain, cough.

Medications: paracetamol

Medical & Surgical Hx: none

FH: Mother has asthma, Grandmother has type 2 diabetes.

Social Hx: Lives at home with his grandmother and brother.[Table t20-jetem-9-4-c1]

**Table t20-jetem-9-4-c1:**

**Vital signs** (need to be obtained):		HR 150 bpm	BP 70/50 mmHg	RR 20 breaths/min
Temp 38 °C	SpO2 95%	Wt: 14 kg		

**Physical exam findings (if asked):**

**Initial Assessment:**

- **Airway/Breathing:** Airway open. Moans and opens eyes in response to voice. Lungs clear to auscultation bilaterally.
- **Circulation:** Tachycardic (HR 150 bpm). No murmur, weak peripheral pulses. Capillary refill 4–5 seconds, extremities cool to touch.
- **Abdominal:** nondistended, high pitched bowel sounds, guarding (involuntary) diffusely, mass felt in RUQ, if diaper examined see “currant jelly” stool.
- **Neuro:** Somnolent but arousable to voice, otherwise non-focal.
- **Other:** Somnolent but arousable. Pale. Dry mucous membranes. No trauma

**Expected actions:**

□ Obtain and interpret laboratory studies
□ Interpret x-ray abdomen
□ Interpret the intussusception identified on ultrasound as the cause of bowel ischemia and septic shock
□ Surgical consultation

**END Round 3.**

Review ongoing management including fluids. Providers should be able to advocate for an appropriate level of care for a critically ill patient. Septic shock requires early fluid replacement and antibiotic administration for best outcomes.

Refer to expected actions and teaching points below:[Table t21-jetem-9-4-c1]

**Table t21-jetem-9-4-c1:**

Expected Action	Teaching Point	Result
Obtain and interpret laboratory studies	There is no singular laboratory test or test pattern that is diagnostic of a bowel obstruction or bowel ischemia Lactic acidosis (if obtained) could suggest bowel ischemia but could also reflect episodes of vomiting Elevated white blood cell count is nonspecific	This patient has an elevated WBC and has pre-renal acute renal failure.
Interpret x-ray abdomen	If the x-ray does not show highly suggestive signs or definitive signs of bowel obstruction, a clinician should seek another more specific test (eg, ultrasound or computer tomography) based on concern by history and physical exam	This x-ray is generally nonspecific, but the arrows are pointing toward soft tissue mass within right lower quadrant which suggests intussusception
Interpret the intussusception identified on ultrasound as the cause of bowel ischemia and septic shock.	Ileo-ileo intussusception does not typically require reduction; however, if there is concern for bowel ischemia, the patient should be evaluated for surgical reduction Ileo-colic intussusception often requires reduction by enema (air or barium) or surgical reduction.	Transverse US shows concentric alternating echogenic and hypoechoic bowel walls. Positive “target sign”
Surgical consultation	Radiologists or surgeons usually perform air enemas or barium enemas in a hemodynamically stable child A surgeon should be consulted for a hemodynamically unstable patient in septic shock for operative reduction	

#### ROUND 4: Total Case

If time allows, restart from the beginning of the scenario and run the full scenario without interruption.

**END Round 4.**

**END OF RCDP Session.**

**Final debriefing and feedback**

- Praise learners for tasks accomplished well
- Provide areas for continued improvement.

*Abdomen X-Ray*

Greif E, Intussusception. Case study, Radiopaedia.org (Accessed on 29 Oct 2024) https://doi.org/10.53347/rID-29373[Fig f3-jetem-9-4-c1]

*Electrocardiogram*

Burns E, et al, Sinus Tachycardia, lifeinthefastlane.com (Accessed on 29 Oct 2024) http://lifeinthefastlane.com/ecg-library/[Fig f4-jetem-9-4-c1]

*Abdominal Ultrasound*

Greif E, Intussusception. Case study, Radiopaedia.org (Accessed on 29 Oct 2024) https://doi.org/10.53347/rID-29373[Fig f5-jetem-9-4-c1][Table t22-jetem-9-4-c1][Table t23-jetem-9-4-c1][Table t24-jetem-9-4-c1][Table t25-jetem-9-4-c1]

**Table t22-jetem-9-4-c1:**

*Complete blood count (CBC)*	
White blood count (WBC)	18.7 × 1000/mm^3^
Hemoglobin (Hgb)	12.3 g/dL
Hematocrit (HCT)	36.9%
Platelet (Plt)	325 × 1000/mm^3^
Segmented Neutrophils	66.7 × 1000/mm^3^
Bands	1.76%
Lymphocytes	25.8 × 1000/mm^3^

**Table t23-jetem-9-4-c1:**

*Comprehensive Metabolic Panel (CMP)*	
Sodium	139 mEq/L
Potassium	3.9 mEq/L
Chloride	110 mEq/L
Bicarbonate (HCO_3_)	26 mEq/L
Glucose	93 mg/dL
Blood Urea Nitrogen (BUN)	44 mg/dL
Creatinine (Cr)	1.4 mg/dL
Calcium	9.0 mg/dL
Total bilirubin	0.6 mg/dL
Alkaline phosphatase	60 units/L
Aspartate aminotransferase (AST)	105 units/L
Alanine aminotransferase (ALT)	95 units/L
Albumin	3.6g/dL

**Table t24-jetem-9-4-c1:**

*Lactic Acid*	3.2 mEq/L

**Table t25-jetem-9-4-c1:**

*Urinalysis (UA)*	
Color	Yellow
Appearance	Clear
Specific Gravity	1.310
pH	7.0
Glucose	Negative
Bilirubin	Negative
Ketones	2
Protein	1+
Leukocyte esterase	1+
Nitrites	negative
White blood cells (WBC)	1 WBCs/high powered field (HPF)
Red blood cells (RBC)	0 RBCs/HPF
Squamous epithelial cells	0–5 cells/HPF

### Appendix K Malnutrition Lecture Synopsis

**Title:** Malnutrition Lecture

**Target Audience:** generalist healthcare providers in low- and middle-income countries

**Educational Methods:** PowerPoint didactic lecture

**Time required for implementation:** about 60 minutes

**Equipment/Environment/Personnel:**

- *A large room (with a capacity of at least 50 people) with multiple tables and ample floor space, or multiple rooms if available*
- *A computer with PowerPoint capability and projector setup*
- *Personnel needed: one lecturer*

**Learning Objectives:**

The learner will be able to:

1. Identify malnutrition and its forms
2. Identify malnutrition emergencies
3. Initiate treatment for malnutrition emergencies

**Lecture Script**:[Table t26-jetem-9-4-c1]

**Table t26-jetem-9-4-c1:**

Slide 1	Title Slide “We are now going to discuss malnutrition.”
Slide 2	Goals - opportunity to review objectives of lecture.
Slide 3	Acronym Key – point out any unusual or less commonly known abbreviations. Refer here if an acronym encountered later is unclear.
Slide 4	Malnutrition has various forms – what are some of the forms that you know about? Marasmus, Kwashiorkor, Stunting and Obesity. Please have the audience describe what are physical findings in the different forms. Marasmus: “skin and bones” or “saggy pants” appearance. Kwashiorkor: protuberant abdomen, extremity and facial swelling, “flaky paint” appearance of skin. Stunting: short stature for age. Obesity: increased weight for age. In this lecture, we will focus on children aged 6 months to 5 years old.
Slide 5	There are several forms of malnutrition: Underweight refers to low weight for age and stunting refers to low height for age. Stunting is associated with developmental delay. Wasting refers to low weight for height, which has a high risk for morbidity and mortality. Although the focus of the lecture is the underweight, it is important to recognize that overweight and obesity is also a form of malnutrition.
Slide 6	More about malnutrition types.
Slide 7	This map shows the most current prevalence of wasting, low weight for height, worldwide.
Slide 8	This map shows the most current prevalence of stunting, low height for age, worldwide.
Slide 9	This graph shows the most current prevalence of overweight, excessive weight for height, worldwide.
Slide 10	In order to compensate for the lack of nutrition, the body enters what is called reductive adaptation: there is reduced metabolic demands on the organs, tissues and cells. Muscle and fat are metabolized in order for the body to perform essential functions. Overall, there is reduced physical activity to decrease demands on the body.
Slide 11	Reductive adaptation impacts every system of the body. Here are just a few.
Slide 12	One major effect of reductive adaptation is disruption in the Na^+^/K^+^ balance. The Na^+^/K^+^/ATP pump malfunctions in malnutrition, leading to increased intracellular sodium and extracellular potassium. Increased intracellular sodium leads to increased total body water. Excess extracellular potassium is lost in the urine, leading to hypokalemia. There is loss of phosphate, calcium, magnesium, and zinc in the urine.
Slide 13	History is key to providing clues about a patient’s nutritional status. It’s important to know about the child’s feeding history (What do they eat? Don’t forget to ask about breastfeeding). Have they had any gastrointestinal losses such as vomiting or diarrhea? Do they have a chronic cough that would make you suspicious of tuberculosis? And what is their developmental history? Have they had any delays that would make them think about stunting?
Slide 14	Physical exam is also key. Vital signs are always important. Hypothermia, hypotension, tachypnea, tachycardia can indicate malnourishment. It is important to ensure that you adequately expose the child in order to look for signs of malnutrition, such as wasting or edema. Anthropometric measurements are critical as they will allow the provider to classify the type of malnutrition the patient has.
Slide 15	The World Health Organization has created standardized anthropometric tables that are separated by gender. There are tables with standard deviations (z-scores) for weight for age, length/height for age (length is used for children <2 years of age), weight for length. Moderate Acute Malnutrition (MAM) is defined by a z-score between −2 and −3 standard deviations from the average anthropometric measurement. Severe Acute Malnutrition (SAM) is a z-score <−3 standard deviations from the average anthropometric measurement. Another way to measure malnutrition is to use the mean upper arm circumference (MUAC). This is measured by measuring the circumference of the midpoint of the upper arm (between the shoulder and the elbow). If this measurement is < 11.5 cm, the patient has SAM. An additional definition of severe acute malnutrition is solely based on the appearance of the child. If they have visible wasting or edema, they are considered to have SAM. Risk of death SAM is nine times higher than that of those −1 standard deviations from the average anthropometric measurement.
Slide 16	There are many features of malnutrition that can be found on exam. On the exam of the head, one may notice a wide or prematurely closed fontanelle in an infant. Flag sign refers to alternating light and dark hair which corresponds to time of good nutrition and poor nutrition. Children can also have fragile, brittle hair.
Slide 17	Eyes may show signs of Vitamin A deficiency such as xerophthalmia (abnormal dryness of the eyes) which lead to Bitot spots (buildup of keratin in the eyes secondary to dryness). The conjunctiva may be pale due to anemia or may show scleral icterus due to liver dysfunction. The ears may have secretions secondary to ear infection.
Slide 18	In the mouth, one may notice stomatitis, bleeding gums, oral candidiasis, poor dentition. The color of the tongue and oral mucosa may also be pale, indicating anemia.
Slide 19	The skin can be dry. For children with edema one may notice a type of dermatitis described as “flaky paint” because it looks like paint peeling. Bruising may also be noticed. Rachitic rosary (expansion of anterior ribs at the costochondral junction) and Harrison’s sulcus (groove at lower end of rib cage due to soft bones) can be seen with Vitamin D and calcium deficiency. Crackles and rhonchi on lung auscultation are signs of pneumonia.
Slide 20	On cardiac exam, one may notice a flow murmur from anemia. The abdomen may be distended (ascites) or have tender hepatomegaly. On extremity exam, may note palmar pallor indicating anemia, poor capillary refill (from hypothermia or poor circulation), or wrist widening, leg bowing, or double malleoli from Vitamin D deficiency
Slide 21	The following laboratory studies and imaging can be helpful in the evaluation of malnutrition: Hemoglobin for anemia Liver function tests for liver dysfunction and hypoalbuminemia HIV as a cause for malnutrition Tuberculin skin testing as a cause for malnutrition Blood cultures for concern for sepsis Chest x-ray for occult pneumonia or tuberculosis or signs of rickets (Vitamin D deficiency) Electrolytes are not initially helpful as they may be deranged and lead to inappropriate therapy
Slide 22	Now we will discuss the treatment of malnutrition.
Slide 23	Treatment of malnutrition is based on whether the patient falls into one of three categories: The first is SAM with complications hypothermia, hypoglycemia, severe dehydration, shock, infection, or severe anemia, wasting, edema, or failure of appetite (meaning the child has been offered something to take orally and refuses). This requires inpatient care. The second category is SAM w/o complications which is managed as an outpatient. And the third category is MAM, which is managed with supplementary feeding. We will discuss the treatment of SAM with complications.
Slide 24	SAM with complications or failure of the appetite test (the patient has anorexia) requires inpatient care as they are at high risk for already having or developing life-threatening complications. These patients need careful evaluation and special treatment with regular feeding and monitoring. This occurs in two phases: stabilization and rehabilitation.
Slide 25	Stabilization has two components. The first is the prevention and treatment of acute complications.
Slide 26	Hypothermia is considered an axillary temperature of <35 °C or rectal <35.5 °C. Treatment consists of starting to feed the child immediately and warming the child. One must take care not to position the child near any draft.
Slide 27	Hypoglycemia is considered <3 mmol/L (54 mg/dL) in a child with severe malnutrition. If one is unable to check for hypoglycemia, it is assumed that a child with severe malnutrition has hypoglycemia, especially if they have signs and symptoms of hypoglycemia. If the child is conscious, they should be given enteral feeding, oral or nasogastric (NG) of 50 ml of D10 and F75 therapeutic milk should be started. If the child is not conscious, they should receive 5 ml/kg of intravenous D10 followed by 50 ml of D10 via NG. F75 therapeutic milk should be given as soon as the child is conscious.
Slide 28	It’s important when assessing dehydration in a patient to ask whether this is a well nourished child that is dehydrated OR a malnourished child that is dehydrated. What are the typical signs to use to diagnose dehydration (DHN) in a well-nourished child? Why can’t we use that in a malnourished child? Lethargy may be related to malnutrition. Eyes may be sunken because of decreased fat behind the eyes. Skin pinch may be delayed because there is less fat and muscle under the skin. Here are the signs of dehydration that should be used to assess dehydration in malnourished children.
Slide 29	For malnourished patients with severe dehydration, it preferred to use the enteral route for hydration unless the patient is in shock. Use ReSoMal (Rehydration Solution for Malnutrition) for rehydration. ReSoMal is specially formulated for malnourished children and preferred to oral rehydration solution (ORS) as it has lower sodium and higher potassium.
Slide 30	WHO defines shock in pediatric patients as capillary refill ≥3 sec **AND** cool extremities **AND** weak pulses. Must have all three to be considered shock. Other signs of shock include tachycardia, low blood pressure, and low urine output
Slide 31	For shock in malnourished patients, the fluids given must be dextrose-containing fluids given slowly: D5 ½ Hartmann’s, D5 ½ Darrow’s solution, or D5 ½ normal saline. It is important to monitor heart and respiratory rate every five minutes to detect fluid overload. If no IV available, one can do oral fluid resuscitation for shock using ReSoMal.
Slide 32	This flowchart shows how to manage a malnourished child with shock (lecturer should lead participants through this chart).
Slide 33	Severe anemia is defined as hemoglobin < 4 g/dL or 6 g/dL with respiratory symptoms. This can lead to heart failure if not addressed. Treatment is to 10 cc/kg of whole blood over three hours.
Slide 34	Children with malnutrition are assumed to have infection. SAM with complications should be empirically started on broad spectrum antibiotics: Benzylpenicillin or Ampicillin × 2 days followed by Amoxicillin × 5 days PLUS Gentamicin × 7 days. Specific infections should be treated such as malaria or parasitic worms. Measles vaccination should be given if not vaccinated or vaccinated before 9 months.
Slide 35	The second component of stabilization is nutritional recuperation. Specialized therapeutic milk, F75, is started gradually.
Slide 36	A dreaded and deadly complication of nutritional recuperation is refeeding syndrome: metabolic and hormonal changes that occur at the initiation of feeding that lead to metabolic derangements. It is associated with fluid and electrolyte shifts.
Slide 37	Refeeding syndrome is characterized by hypophosphatemia but also includes decreased potassium and magnesium. This can lead to several complications.
Slide 38	When a child is gaining weight without complications and has a good appetite, they can enter the transition phase. The therapeutic feeding changes either to F-100 therapeutic milk or Ready-to-Use-Therapeutic-Food (eg, Plumpy Nut or Vita Nut). This differs from F75 in that it has higher calories and protein. The daily caloric goal increases as well. Patients should be observed for vomiting and diarrhea as signs of intolerance.
Slide 39	The second phase of inpatient care for SAM with complications is rehabilitation. At this time, the quantity of F100 or RUTF will increase and the child can start to eat home foods.
Slide 40	Children who are less than 6 months old with SAM should be admitted to a hospital. Other causes such as congenital diseases must also be considered. Their nutritional recuperation consists of diluted F100 milk using the supplementary suckling technique.
Slide 41	How do we treat patients who have SAM and no complications?
Slide 42	SAM without complications is managed in the outpatient setting. Patients are given RUTF and started on empiric antibiotics. Measles vaccination is given if the patient has not received it previously.
Slide 43	And lastly, the treatment for MAM.
Slide 44	MAM is also managed in the outpatient setting. Children are referred to supplementary feeding programs that take place at local health centers or schools. There are also programs that deliver supplemental foods to families.
Slide 45	There are many types of malnutrition. Children with SAM with complications should be admitted to the hospital.
Slide 46	References

**References:**

- Caballero B. *Encyclopedia of human nutrition*. Elsevier; 2005 Jul 20.
- Levels and trends in child malnutrition: UNICEF/WHO/The World Bank Group joint child malnutrition estimates: key findings of the 2021 edition. (World Health Organization, 2021). At: https://www.who.int/publications/i/item/9789240025257
- Saunders J, Smith T. Malnutrition: causes and consequences. *Clin Med*. 2010 Dec;10(6):624.
- Saunders J, Smith T, Stroud M. Malnutrition and undernutrition. *Medicine*. 2015 Feb 1;43(2):112–8.
- Fischer M, JeVenn A, Hipskind P. Evaluation of muscle and fat loss as diagnostic criteria for malnutrition. *Nutr Clin Pract*.2015;30:239–248
- Esper DH. Utilization of nutrition-focused physical assessment in identifying micronutrient deficiencies. *Nutr Clin Pract*.2015;30:194–202.
- Radler DR, Lister T. Nutrient deficiencies associated with nutrition-focused physical findings of the oral cavity. *Nutr Clin Pract.* 2013;28:710–721.13.
- Dibaise M, Tarleton SM. Hair, nails, and skin: differentiating cutaneous manifestations of micronutrient deficiency. *Nutr Clin Pract*. 2019;34:490–503
- Management of Severe Malnutrition: A Manual for Physicians and Other Senior Health Workers (World Health Organization, 1999). At: https://www.who.int/publications/i/item/9241545119
- Manary MJ, Sandige HL. Management of acute moderate and severe childhood malnutrition. *BMJ.* 2008 Nov 13;337.
- Guideline: Updates on the management of severe acute malnutrition in infants & children (World Health Organization, 2013). At: https://www.who.int/publications/i/item/9789241506328
- Emergency Triage Assessment and Treatment (ETAT): Participant Manual. (World Health Organization, 2005). At: https://www.who.int/publications/i/item/9241546875 https://www.who.int/publications/i/item/9241546875
- Updated guideline: paediatric emergency triage, assessment and treatment. (World Health Organization, 2016). At: https://www.ncbi.nlm.nih.gov/books/NBK350528/

### Appendix L Malnutrition Lecture

Please see associated PowerPoint file

### Appendix M Malnutrition Small Group Discussion

**Equipment/Environment/Personnel:**

- A large room (with a capacity of at least 50 people) with multiple tables and ample floor space, or multiple rooms if available
  ○ For each group of 3 to 5 learners, one setup includes 3 to 5 seats set up in a circle
- One small group facilitator per group of 3 to 5 learners

**Recommended pre-reading for instructor:**

- Malnutrition. *Pocket Book of Hospital Care for Children: Guidelines for the Management of Common Childhood Illnesses* 2nd edition. Geneva: World Health Organization; 2013:*157.*
- Available from:
- https://www.ncbi.nlm.nih.gov/books/NBK154450/

**Abbreviations:** [Table t27-jetem-9-4-c1]

**Table t27-jetem-9-4-c1:**

A&E –	Accident and Emergency department
BP –	Blood Pressure
HIV –	Human Immunodeficiency Virus
HR –	Heart Rate
IO –	intraosseous cannulation
IV –	intravenous
NG –	Nasogastric
O2 Sat –	Oxygen Saturation
ReSoMal –	Rehydration Solution for Malnutrition
SAM –	Severe Acute Malnutrition
T –	Temperature
WHO –	World Health Organization

**Tips for successful implementation:**

- The facilitator should read the prompts (**in bold**) to the learners.
- Following the prompts are cues that can be given from the facilitator.
- Because the providers are from varying disciplines and with different backgrounds and experiences, discussion can be facilitated by asking participants to speak on their experiences with similar patients.
  ○ It is beneficial to have various disciplines represented in each group, rather than having the nurses be in one group and physicians in another. It can help lead to more robust discussion.
  ○ Nurses may not be very comfortable in advanced medical decision-making but they may feel comfortable in making triage decisions or sharing tips/tricks in achieving goals with pediatric patients.
  ○ The facilitator should ask follow-up questions to elicit clinical areas where practitioners feel that they perform well and areas in which they can improve, utilizing the group to help understand each other’s difficulties and to problem-solve together.
  ○ Some participants may be very confident and talkative, but because this is meant to be a group exercise, it may be useful to initially have any member of the group respond, but then to selectively encourage those who are more reserved to give their responses.

**Case Studies**

**Case 1:** Malnutrition and Dehydration.

You are called to see a 3-year-old girl in the A&E for “low energy” after 1 week of vomiting and diarrhea. Her mother is with her.

**Question Prompts:**

1. What signs help you triage the patient?
  - A – Airway
  - B – Breathing
  - C – Circulation
  - D – Disability

The child is laying in her mother’s arms.

Her eyes are open, and her breathing pattern is regular and comfortable.

She appears tired.

Her arms and legs are thin, with loose skin folds over the axilla.

She is irritable and cries when you touch her.

Her hands and feet are cold, capillary refill is 4–5 seconds, and her radial pulse is weak.

2. What is the child’s condition?
  - Shock, SAM
3. What should you do?
  - More history, keep warm, restore volume
  - Start IV fluids
4. What if you are unable to start an IV? What are your other options?
  - Use an IO or NG.
5. How do you place an IO?
  - 2–3 fingerbreadths below the tibial tuberosity on the medial aspect
6. How do you place an NG tube?
  - Measure from the nares to ear to xiphoid process.
7. How much fluid would you give via NG?
  - 5 mL/kg of ReSoMal orally or NG every 30 minutes for 2 hours.
8. You are able to establish an IV. How much fluid will you give the child?
  - Weigh the child.
  - Establish IV access.
  - Give 10–15 mL/kg of D5 ½ Hartmann’s, D5 ½ Darrow’s, or D5 ½ NS over 1 hour.
9. If the child weighs 10 kg, how much fluid should she get?
  - 100–150 ml.
10. What is the child at risk for while receiving fluids? What do you need to monitor during volume repletion?
  - Heart failure
  - Monitor heart rate and respiratory rate every 5 minutes.
11. How can you tell if these complications are developing?
  - The heart rate and respiratory rate increase while receiving fluids.
12. What should you do if that occurs?
  - Stop IV fluids.
13. If the child does not develop these complications, what should you do next?
  - Evaluate the child for improvement.

The child is moving her arms and legs and is trying to sit up.

Her cap refill is 2 seconds.

Her hands and feet are still slightly cool to touch.

Her radial pulse is 2+.

14. What do you do next?
  - Switch to maintenance oral or NG fluids (ReSoMal)
  - Initiate refeeding with F-75
15. Why is the best maintenance fluid choice in severe dehydration ReSoMal, instead of the standard ORS?
  - A malnourished child already has excess intracellular sodium and decreased extracellular potassium. ReSoMal is higher in potassium and lower in sodium content.
16. How do you calculate maintenance fluid rate?
  - 4 mL/kg/hr for the first 10 kg + 2 mL/kg/hr for the next 10 kg + 1 mL/kg/hr for the remaining kg.
17. This population is also at risk of severe anemia (nutritional deficiencies, bacteremia, parasitic worms, HIV-- all place children at risk). What is the danger of severe anemia?
  - Decreased oxygen carrying capacity.
18. Discuss the indications for blood transfusion in this population.
  - Hemoglobin <4, or <6 with respiratory distress, or no improvement in shock after the first fluid bolus.
19. What is the correct quantity of whole blood to give?
  - 10 mL/kg of whole blood over 3 hours.

**Case 2:** Malnutrition and Sepsis.

You are called to see a 3-year-old girl in the A&E for fever. Her mother is with her.

1. What signs help you triage the patient?
  - A – Airway
  - B – Breathing
  - C – Circulation
  - D – Disability

The child is laying in her mother’s arms. She is crying. Her extremities are very thin with loose skin folds over the axilla. Her face and trunk feel hot but her hands and feet are cool. Capillary refill is 4–5 seconds and her pulse is weak. Her vital signs are temperature 39.4 °C, pulse 130, respiratory rate 35, SpO2 98%.

1. What is the child’s condition?
  - Septic shock in the setting of malnutrition.

Severely malnourished children require heightened awareness for the presence of bacterial infections for several reasons:

1. Depressed immune system leading to greater risk.
2. Diagnosis may be difficult because these children may be unable to mount a fever or exhibit other common signs of infection.

2. What should you do?
  - Restore volume
  - Give oxygen
  - Find source of infection
  - Treat infection
3. How should you restore volume?
  - Establish vascular access.
  - Give 10–15 mL/kg of D5 ½ NS over one hour.
4. How should you search for infection?
  - Obtain labs:
    - CBC
    - Blood culture
5. How should you treat infection?
  - Give antipyretic (paracetamol) and broad-spectrum antibiotics.
6. What antibiotics would you give if available?
  - Ampicillin or benzylpenicillin PLUS gentamicin.
7. How often should you re-evaluate the patient?
  - Every 5 minutes.

Your interventions are performed. You obtain another set of vital signs:[Table t28-jetem-9-4-c1]

**Table t28-jetem-9-4-c1:**

T: 37.6 °C	HR: 125	RR 30

Her hands and feet remain cool to touch. Cap refill is 4 seconds.

8. What is the child’s condition?
  - She is unchanged.
9. What should you do next?
  - Give a blood transfusion of 10 mL/kg over 3 hours.
10. Discuss the indication for blood transfusion in this child and why.
  - No improvement of shock after 1 h of IV fluid therapy, to increase oxygen carrying capacity to the tissues.
11. How often should you reassess? What are you worried may happen?
  - Every 5 minutes. Her malnourished state puts her at risk for heart failure.

After the transfusion the child’s hands and feet are warm.

Cap refill is 3 seconds. Her repeat vital signs are:[Table t29-jetem-9-4-c1]

**Table t29-jetem-9-4-c1:**

T: 37.8 °C	HR: 98	RR: 20	Spo2: 99%

12. What should you do next?
  - Switch to maintenance oral or NG fluids (ReSoMal). Initiate refeeding with F-75.
  - Continue antibiotics. Follow up on lab results.

**Case 3:** Malnutrition and hypoglycemia.

You are called to see a 3-year-old girl in A&E for seizure. Her mother is with her and states the child had full body shaking at home; she is no longer shaking but is very sleepy.

1. What signs help you triage the patient
  - Airway
  - Breathing
  - Circulation
  - Disability

The girl’s body is limp and cool.

She has regular and even respirations.

She responds only to painful stimuli.

Her body is swollen throughout, and her belly is big.

When you press on her legs, your fingers make indentations that take several seconds to go away.

2. What do you think is the cause of her current state?
  - Could be hypoglycemia, electrolyte abnormality, or infection that caused her seizure. She is also severely malnourished given her full body edema.
3. What should you do next?
  - Search for the cause of her symptoms – obtain a fingerstick glucose.

Her fingerstick glucose is 25.

4. How do you correct her hypoglycemia?
  - Establish IV access and give 5 mL/kg of IV 10% glucose.

The child is now more alert and starts to cry.

She is moving all extremities spontaneously.

5. What do you do next?
  - Repeat a glucose measurement.

The repeat glucose is 77.

6. What do you do next?
  - Begin to treat her malnutrition using the F-75 formula.
7. What is the safest method for giving this diet in a malnourished child?
  - Frequent, small volume feeds.

**Case 4:** Malnutrition and recuperation.

You are called to see a 3-year-old girl in A&E for “swelling.”

1. What do you want to know?
  - Airway
  - Breathing
  - Circulation
  - Disability
  - Overall physical appearance

The child is standing on her own and cries when you approach.

She has significant edema to the periorbital region, cheeks, abdomen, and extremities.

There are hyperpigmented peeling lesions on her legs.

Her hands and feet are warm with capillary refill of 3 seconds.

2. What state is this child in?
  - Malnutrition.
3. How do you assess the degree of malnutrition?
  - WHO weight-for-height anthropometric tables or mid-upper arm circumference.
4. The child has a weight for height <−3 z score. What are your next steps for the child?
  - Look for malnutrition emergencies: hypothermia, hypoglycemia, dehydration, infection/sepsis, severe anemia. She should be started immediately on antibiotics.

*Of note – do NOT treat this child’s edema with diuretics. The child is edematous not because total body water is high, but because of low body protein content leading to incorrect distribution of total body water.

Her T is 37 °C and blood glucose is 65.

She has no recent loss or worsening of sunken eyes.

She has no palmar pallor.

5. What electrolyte problems could develop during feeding in this malnourished child? What are symptoms of these electrolyte problems?
  - Hypokalemia – weakness, fatigue, muscle cramps. If severe can lead to cardiac dysfunction (arrhythmias)
  - Hypophosphatemia – weakness, confusion
  - Hypomagnesemia – weakness, arrhythmias, tremors
6. How often are you able to check electrolyte levels at your facility?
  - Audience response.

*Of note, diarrhea can also occur with refeeding due to osmotic effects in the gut. This can worsen electrolyte deficiencies, especially hypokalemia.

**References:**

1. *Management of Severe Malnutrition: A Manual for Physicians and Other Senior Health Workers* (World Health Organization, 1999). At: https://www.who.int/publications/i/item/9241545119
2. Guideline: updates on the management of severe acute malnutrition in infants & children (World Health Organization, 2013). At: https://www.who.int/publications/i/item/9789241506328
3. *Emergency Triage Assessment and Treatment (ETAT): Participant Manual*. (World Health Organization, 2005). At: https://iris.who.int/bitstream/handle/10665/43386/9241546875_eng.pdf
4. https://iris.who.int/handle/10665/204463 (World Health Organization, 2016).
5. Pocket Book of Hospital Care for Children: Guidelines for the Management of Common Childhood Illnesses. 2nd edition. (World Health Organization, 2013).

### Appendix N Posttest Questions

Name: _______________________

1. Mary is 2-year-old girl brought in by her grandmother because she thinks the child has worms – “her belly is big.” On exam, Mary says “no” when you ask her to come to you. Her breathing is not labored. Her hands are cool to touch, strong pulse, and she has a capillary refill of less than 3 seconds. She is alert and active. You notice she has very puffy eyes and swollen feet. Her hair is a light brown and broken off in many areas of her scalp. Her triage vital signs show a temperature of 34.8°C. You have decided to admit her. Of the following, which would not be an appropriate next step in her care:
  - Cover her head
  - Have her sit in the sun while waiting in the queue
  - Initiate nutritional recovery
  - Wrap her in a blanket
2. In a well-appearing, afebrile 8-year-old female who is presenting with crampy abdominal pain and diarrhea for 5 days, which of the following is the preferred management approach?
  - Oral rehydration and symptomatic outpatient therapy
  - Oral rehydration, laboratory tests, and empiric antibiotic therapy
  - Laboratory tests, empiric antibiotic therapy, and intravenous fluids
  - Laboratory tests, x-ray, empiric antibiotic therapy
3. Michael is an 18-month-old male weighing 15 kg because he is not eating and is not as active as normal. He has had vomiting and diarrhea for 3 days. On exam, he moans, his breathing is not labored, and his capillary refill is 4 seconds. His hands are warm and his pulse is strong. He is awake but listless. You check his blood glucose and it is 44 mg/dL. What is the next appropriate step?
  - Place an IV and give him 15 cc of D50
  - Place an IV and give him 30 cc of D10
  - Place a nasogastric tube and give him 150 cc of D10
  - Place a nasogastric tube and give him 30 cc of D25
4. Which of the following statements about nutritional recovery is true?
  - Diuretics should be used to decrease the peripheral edema seen in severe malnutrition
  - F-75 provides complete nutrition for patients with malnutrition
  - Patient presenting with severe malnutrition should be empirically treated with antibiotics
  - Refeeding syndrome causes rhabdomyolysis and cardiac infarcts secondary to hyponatremia
5. A 10-year-old female presents for vomiting after having visited a friend's birthday party. Which of the following statements regarding *Staphylococcal* food poisoning is correct?
  - A single person ingesting food cannot be the only person of a group affected
  - Food contaminated by Staphylococcus has a bitter, spicy taste
  - Profuse, bloody diarrhea is the distinguishing characteristic
  - Symptoms begin within 1–6 hours of ingestion
6. A newborn boy presents with projectile vomiting and is always hungry to eat more after vomiting. The vomit is non-bilious, and an olive shaped mass is palpable in the patient’s abdomen. The patient has been treated with erythromycin for an infection. What is the best initial diagnostic test?
  - Computed tomography of abdomen
  - Hypokalemic, hypochloremic metabolic alkalosis
  - Ultrasound pylorus
  - X-ray of the abdomen
7. Which of the following could indicate a malrotation of the midgut and volvulus on x-ray of the abdomen?
  - Corkscrew sign on x-ray abdomen
  - Double bubble sign on x-ray abdomen
  - Whirlpool sign on ultrasound
  - All the above
8. What is the most common cause of small bowel obstruction in children?
  - Adhesions
  - Hernia
  - Intussusception
  - Midgut volvulus
9. An 11-month-old boy is brought in by his parents for vomiting and crying on and off all afternoon. They are concerned that he is in pain. He had one episode of diarrhea yesterday. On exam he is awake and interactive. He begins to pull his legs up and cry for several minutes before relaxing again. Vital signs are blood pressure 96/75mmHg, pulse 98 beats/minute, respirations 32 breaths/minute, and temperature 37.2°C (99°F). Which of the following tests would have the greatest specificity?
  - Complete blood count
  - Contrast enema
  - Plain abdominal x-rays
  - Upper gastrointestinal series
10. A 6-year-old girl presents to the emergency department with poor urine output. She had bloody diarrhea a few last weeks with fevers that have now resolved. On exam, she is pale and ill appearing. Your next step is:
  - Administer an IV isotonic fluid bolus of 40 mL/kg
  - Check labs including full blood count, electrolytes, and renal function
  - Discharge home with oral rehydration instructions
  - Send stool cultures and begin antibiotics
11. An unvaccinated 2-year-old male presents with 3 days of non-bloody diarrhea after having 3 days of intermittent non-bloody, non-bilious vomiting. He has been taking fluids well but has had decreased solid intake. There has been no fever. He attends daycare. There has been no travel. The most likely pathogen causing his symptoms is:
  - *Clostridium difficile*
  - *Proteus mirabilus*
  - Rotavirus
  - *Salmonella*
12. Which of the following statements is false?
  *Reductive adaptation* describes the process in malnutrition in which the body compensates for lack of food by:
  - Decreasing gastrointestinal secretions
  - Decreasing lymphocyte & interleukin production so it is easier for patients to have a fever
  - Reducing arterial blood pressure and favoring central circulation
  - Reducing hemoglobin synthesis
13. A 3-year-old child presents for vomiting and fever. On exam, you find a round, 3 cm tender mass at the left groin. An x-ray obtained shows enlarged bowel loops and air-fluid levels at that same location in the groin.
  - Admit for nasogastric tube decompression
  - Colonoscopy for sigmoid reduction
  - Emergent surgical consultation
  - Incision and drainage
14. A 3-year-old male presents to the emergency department with frequent diarrhea for 3 days. On exam, he has sunken eyes and skin pinch is 2 seconds. He is more irritable than usual, but is alert, and when offered liquids, drinks eagerly. How would you classify his degree of dehydration?
  - Mild dehydration
  - Moderate dehydration
  - No dehydration
  - Severe dehydration
15. Karl is an 8-month-old male brought in for multiple episodes of non-bloody diarrhea starting 2 days ago. You notice that he is crying without tears and his breathing is not labored. His hands are warm to touch, a strong pulse, and capillary refill of 5 seconds. He is alert. His ribs are easily visible, and he has a “saggy pants” appearance to his posterior. His weight is 6 kg. What is the next best step in management of this patient?
  - Place a nasogastric tube and give ReSoMal 30 cc every 30 minutes for 2 hours
  - Place a nasogastric tube and start F-75 as soon as possible
  - Start an IV and give D5 ½ normal saline 60–90 cc over 1 hour
  - Start an IV and give 120 cc over 1 hour

### Appendix O Posttest Answers

1. Mary is 2-year-old girl brought in by her grandmother because she thinks the child has worms – “her belly is big.” On exam, Mary says “no” when you ask her to come to you. Her breathing is not labored. Her hands are cool to touch, strong pulse, and she has a capillary refill of less than 3 seconds. She is alert and active. You notice she has very puffy eyes and swollen feet. Her hair is a light brown and broken off in many areas of her scalp. Her triage vital signs show a temperature of 34.8°C. You have decided to admit her. Of the following, which would not be an appropriate next step in her care:
  - Cover her head
  - **Have her sit in the sun while waiting in the queue**
  - Initiate nutritional recovery
  - Wrap her in a blanket
2. In a well-appearing, afebrile 8-year-old female who is presenting with crampy abdominal pain and diarrhea for 5 days, which of the following is the preferred management approach?
  - **Oral rehydration and symptomatic outpatient therapy**
  - Oral rehydration, laboratory tests, and empiric antibiotic therapy
  - Laboratory tests, empiric antibiotic therapy, and intravenous fluids
  - Laboratory tests, x-ray, empiric antibiotic therapy
3. Michael is an 18-month-old male weighing 15 kg because he is not eating and is not as active as normal. He has had vomiting and diarrhea for 3 days. On exam, he moans, his breathing is not labored, and his capillary refill is 4 seconds. His hands are warm and his pulse is strong. He is awake but listless. You check his blood glucose and it is 44 mg/dL. What is the next appropriate step?
  - Place an IV and give him 15 cc of D50
  - Place an IV and give him 30 cc of D10
  - Place a nasogastric tube and give him 150 cc of D10
  - **Place a nasogastric tube and give him 30 cc of D25**
4. Which of the following statements about nutritional recovery is true?
  - Diuretics should be used to decrease the peripheral edema seen in severe malnutrition
  - F-75 provides complete nutrition for patients with malnutrition
  - **Patient presenting with severe malnutrition should be empirically treated with antibiotics**
  - Refeeding syndrome causes rhabdomyolysis and cardiac infarcts secondary to hyponatremia
5. A 10-year-old female presents for vomiting after having visited a friend's birthday party. Which of the following statements regarding *Staphylococcal* food poisoning is correct?
  - A single person ingesting food cannot be the only person of a group affected
  - Food contaminated by Staphylococcus has a bitter, spicy taste
  - Profuse, bloody diarrhea is the distinguishing characteristic
  - **Symptoms begin within 1–6 hours of ingestion**
6. A newborn boy presents with projectile vomiting and is always hungry to eat more after vomiting. The vomit is non-bilious, and an olive shaped mass is palpable in the patient’s abdomen. The patient has been treated with erythromycin for an infection. What is the best initial diagnostic test?
  - Computed tomography of abdomen
  - Hypokalemic, hypochloremic metabolic alkalosis
  - **Ultrasound pylorus**
  - X-ray of the abdomen
7. Which of the following could indicate a malrotation of the midgut and volvulus on x-ray of the abdomen?
  - Corkscrew sign on x-ray abdomen
  - Double bubble sign on x-ray abdomen
  - Whirlpool sign on ultrasound
  - **All the above**
8. What is the most common cause of small bowel obstruction in children?
  - Adhesions
  - Hernia
  - **Intussusception**
  - Midgut volvulus
9. An 11-month-old boy is brought in by his parents for vomiting and crying on and off all afternoon. They are concerned that he is in pain. He had one episode of diarrhea yesterday. On exam he is awake and interactive. He begins to pull his legs up and cry for several minutes before relaxing again. Vital signs are blood pressure 96/75mmHg, pulse 98 beats/minute, respirations 32 breaths/minute, and temperature 37.2°C (99°F). Which of the following tests would have the greatest specificity?
  - Complete blood count
  - **Contrast enema**
  - Plain abdominal x-rays
  - Upper gastrointestinal series
10. A 6-year-old girl presents to the emergency department with poor urine output. She had bloody diarrhea a few last weeks with fevers that have now resolved. On exam, she is pale and ill appearing. Your next step is:
  - Administer an IV isotonic fluid bolus of 40 mL/kg
  - **Check labs including full blood count, electrolytes, and renal function**
  - Discharge home with oral rehydration instructions
  - Send stool cultures and begin antibiotics
11. An unvaccinated 2-year-old male presents with 3 days of non-bloody diarrhea after having 3 days of intermittent non-bloody, non-bilious vomiting. He has been taking fluids well but has had decreased solid intake. There has been no fever. He attends daycare. There has been no travel. The most likely pathogen causing his symptoms is:
  - *Clostridium difficile*
  - *Proteus mirabilus*
  - **Rotavirus**
  - *Salmonella*
12. Which of the following statements is false?
  *Reductive adaptation* describes the process in malnutrition in which the body compensates for lack of food by:
  - Decreasing gastrointestinal secretions
  - **Decreasing lymphocyte & interleukin production so it is easier for patients to have a fever**
  - Reducing arterial blood pressure and favoring central circulation
  - Reducing hemoglobin synthesis
13. A 3-year-old child presents for vomiting and fever. On exam, you find a round, 3 cm tender mass at the left groin. An x-ray obtained shows enlarged bowel loops and air-fluid levels at that same location in the groin.
  - Admit for nasogastric tube decompression
  - Colonoscopy for sigmoid reduction
  - **Emergent surgical consultation**
  - Incision and drainage
14. A 3-year-old male presents to the emergency department with frequent diarrhea for 3 days. On exam, he has sunken eyes and skin pinch is 2 seconds. He is more irritable than usual, but is alert, and when offered liquids, drinks eagerly. How would you classify his degree of dehydration?
  - Mild dehydration
  - **Moderate dehydration**
  - No dehydration
  - Severe dehydration
15. Karl is an 8-month-old male brought in for multiple episodes of non-bloody diarrhea starting 2 days ago. You notice that he is crying without tears and his breathing is not labored. His hands are warm to touch, a strong pulse, and capillary refill of 5 seconds. He is alert. His ribs are easily visible, and he has a “saggy pants” appearance to his posterior. His weight is 6 kg. What is the next best step in management of this patient?
  - **Place a nasogastric tube and give ReSoMal 30 cc every 30 minutes for 2 hours**
  - Place a nasogastric tube and start F-75 as soon as possible
  - Start an IV and give D5 ½ normal saline 60–90 cc over 1 hour
  - Start an IV and give 120 cc over 1 hour
