# Supplementary material for: A Simulation and Small-Group Pediatric Emergency Medicine Course for Generalist Healthcare Providers: Gastrointestinal and Nutrition Emergencies
Source: J Educ Teach Emerg Med. 2024 Oct 31;9(4):C1–C120. doi: 10.21980/J8WH2K (PMC11537732; doi:10.21980/J8WH2K)
Supplement: Supplementary file 1 — Please see associated PowerPoint file [file 9-4-C1-Appendix_F.pptx]

## Slide 1
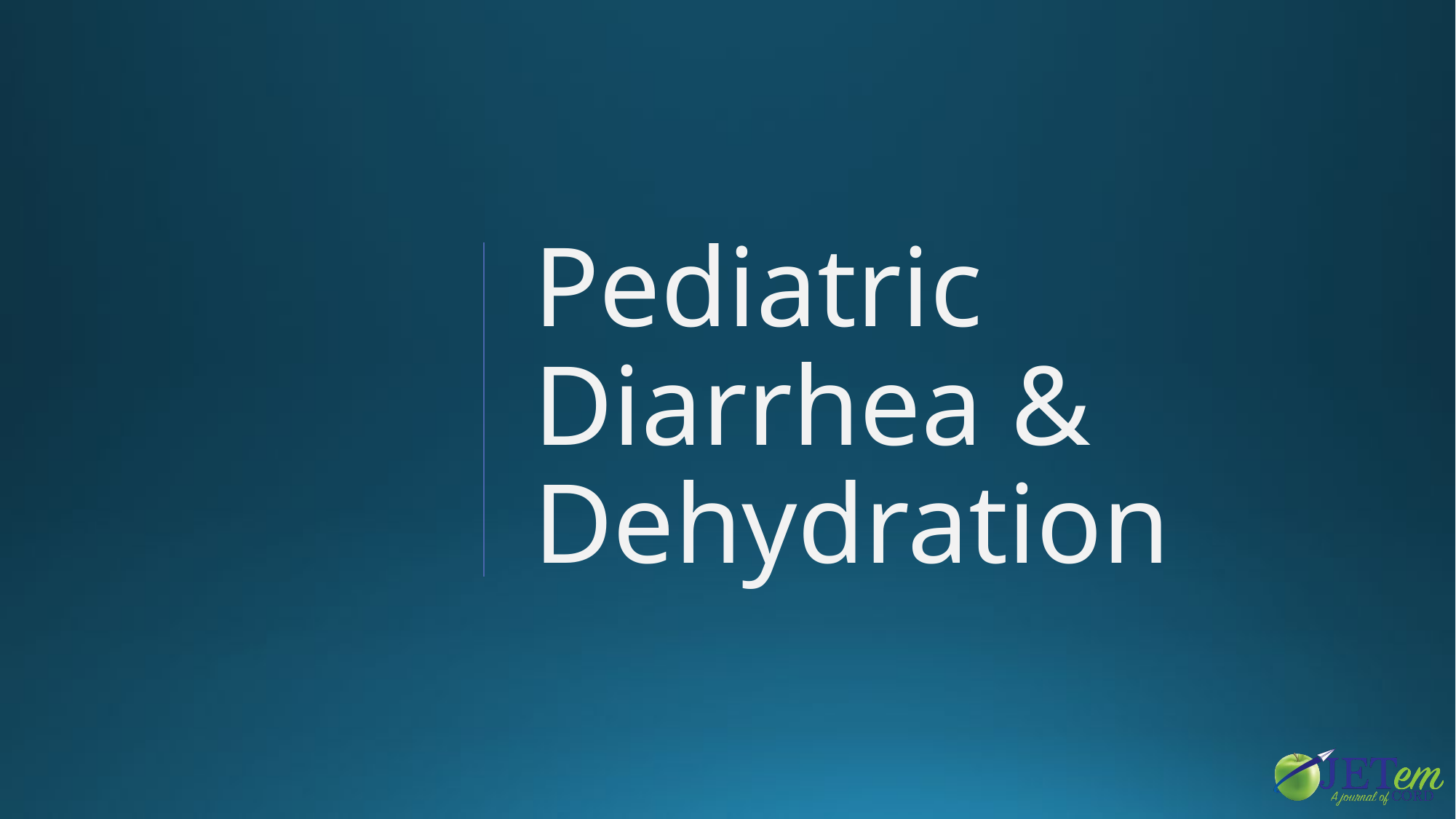

# Pediatric Diarrhea & Dehydration

## Slide 2
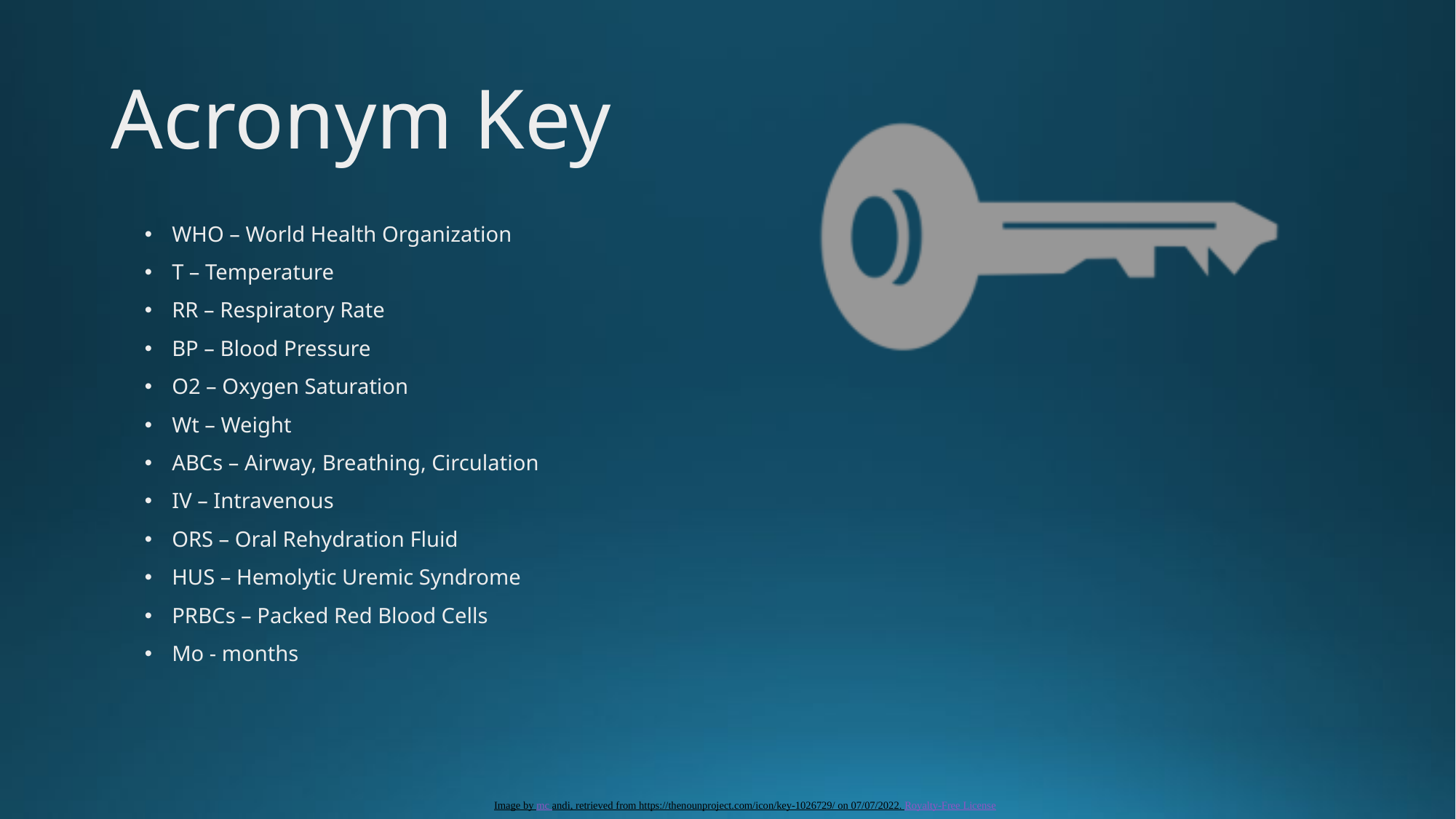

# Acronym Key
WHO – World Health Organization
T – Temperature
RR – Respiratory Rate
BP – Blood Pressure
O2 – Oxygen Saturation
Wt – Weight
ABCs – Airway, Breathing, Circulation
IV – Intravenous
ORS – Oral Rehydration Fluid
HUS – Hemolytic Uremic Syndrome
PRBCs – Packed Red Blood Cells
Mo - months
Image by mc andi, retrieved from https://thenounproject.com/icon/key-1026729/ on 07/07/2022. Royalty-Free License

## Slide 3
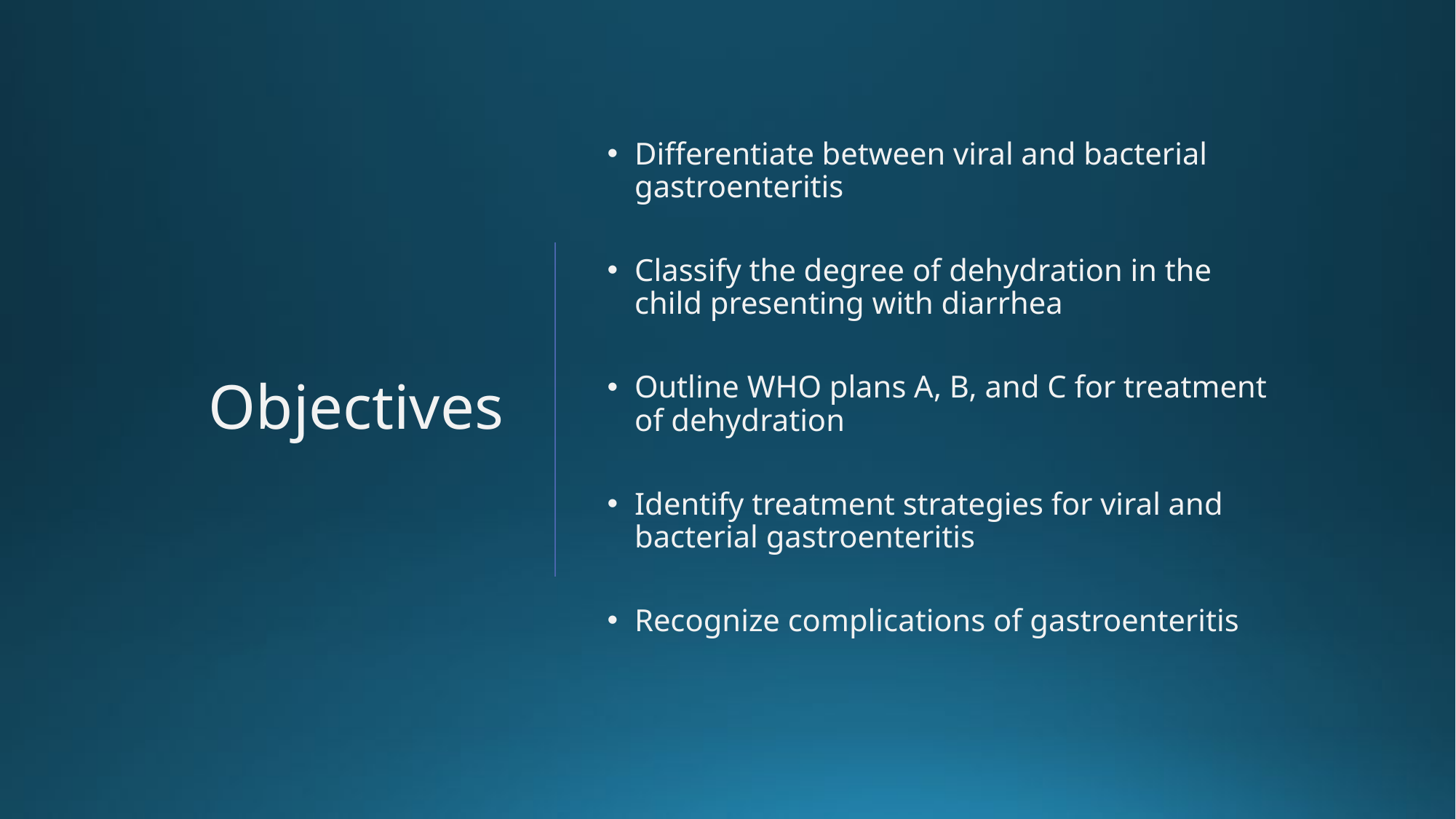

# Objectives
Differentiate between viral and bacterial gastroenteritis
Classify the degree of dehydration in the child presenting with diarrhea
Outline WHO plans A, B, and C for treatment of dehydration
Identify treatment strategies for viral and bacterial gastroenteritis
Recognize complications of gastroenteritis

## Slide 4
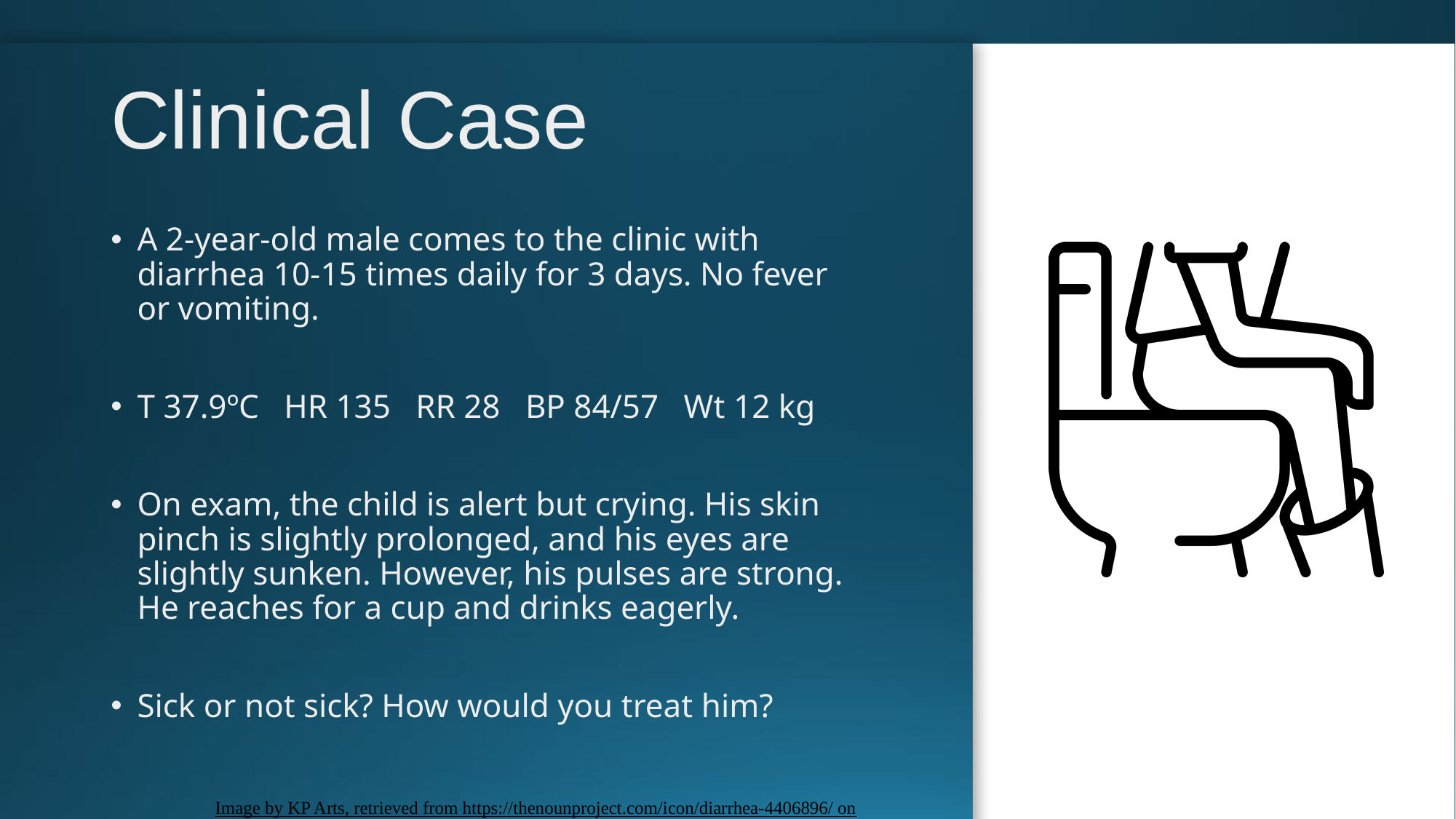

# Clinical Case
A 2-year-old male comes to the clinic with diarrhea 10-15 times daily for 3 days. No fever or vomiting.
T 37.9ºC HR 135 RR 28 BP 84/57 Wt 12 kg
On exam, the child is alert but crying. His skin pinch is slightly prolonged, and his eyes are slightly sunken. However, his pulses are strong. He reaches for a cup and drinks eagerly.
Sick or not sick? How would you treat him?
Image by KP Arts, retrieved from https://thenounproject.com/icon/diarrhea-4406896/ on 2/4/23. Royalty-Free License

## Slide 5
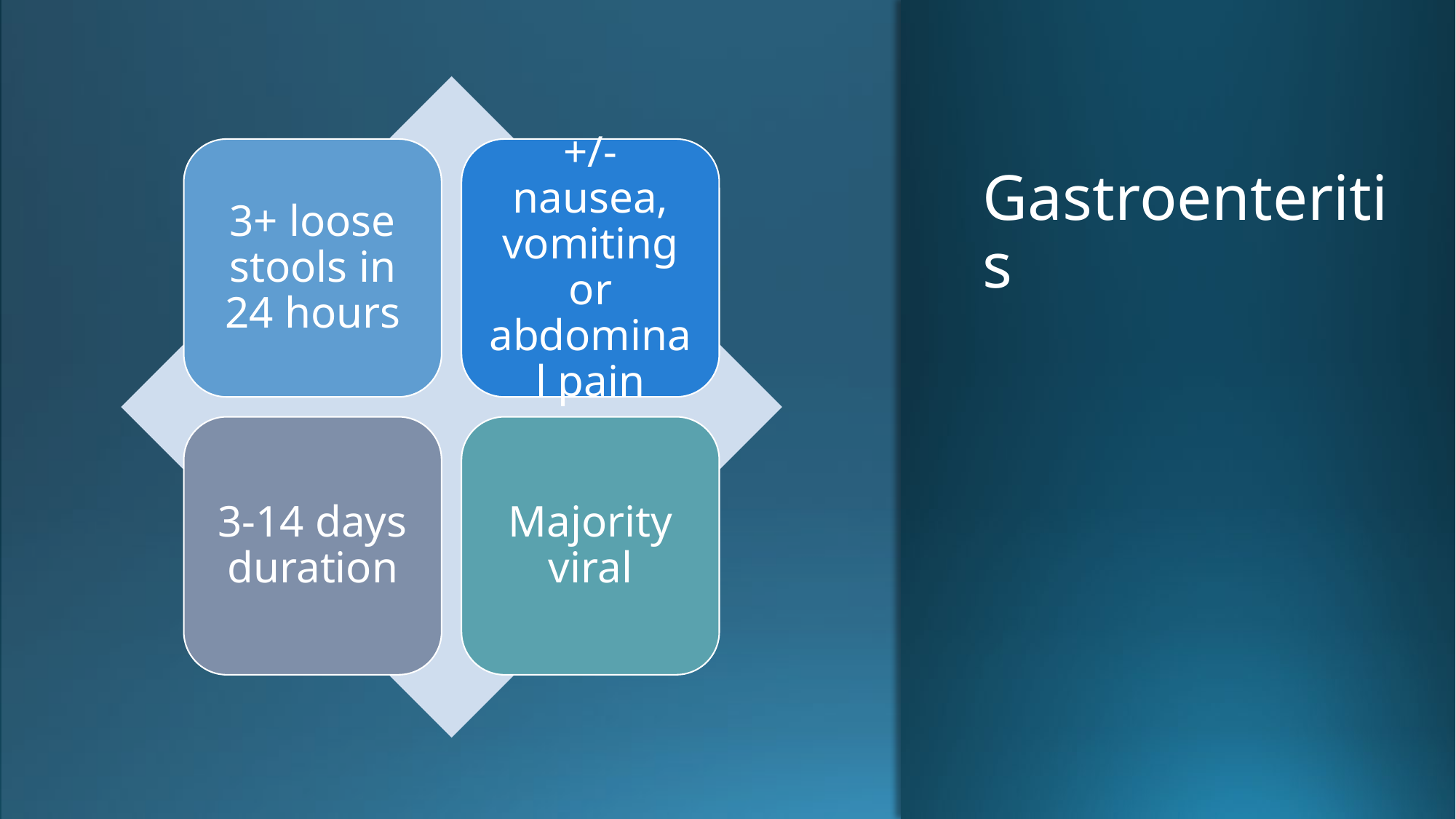

3+ loose stools in 24 hours
+/- nausea, vomiting or abdominal pain
3-14 days duration
Majority viral
# Gastroenteritis

## Slide 6
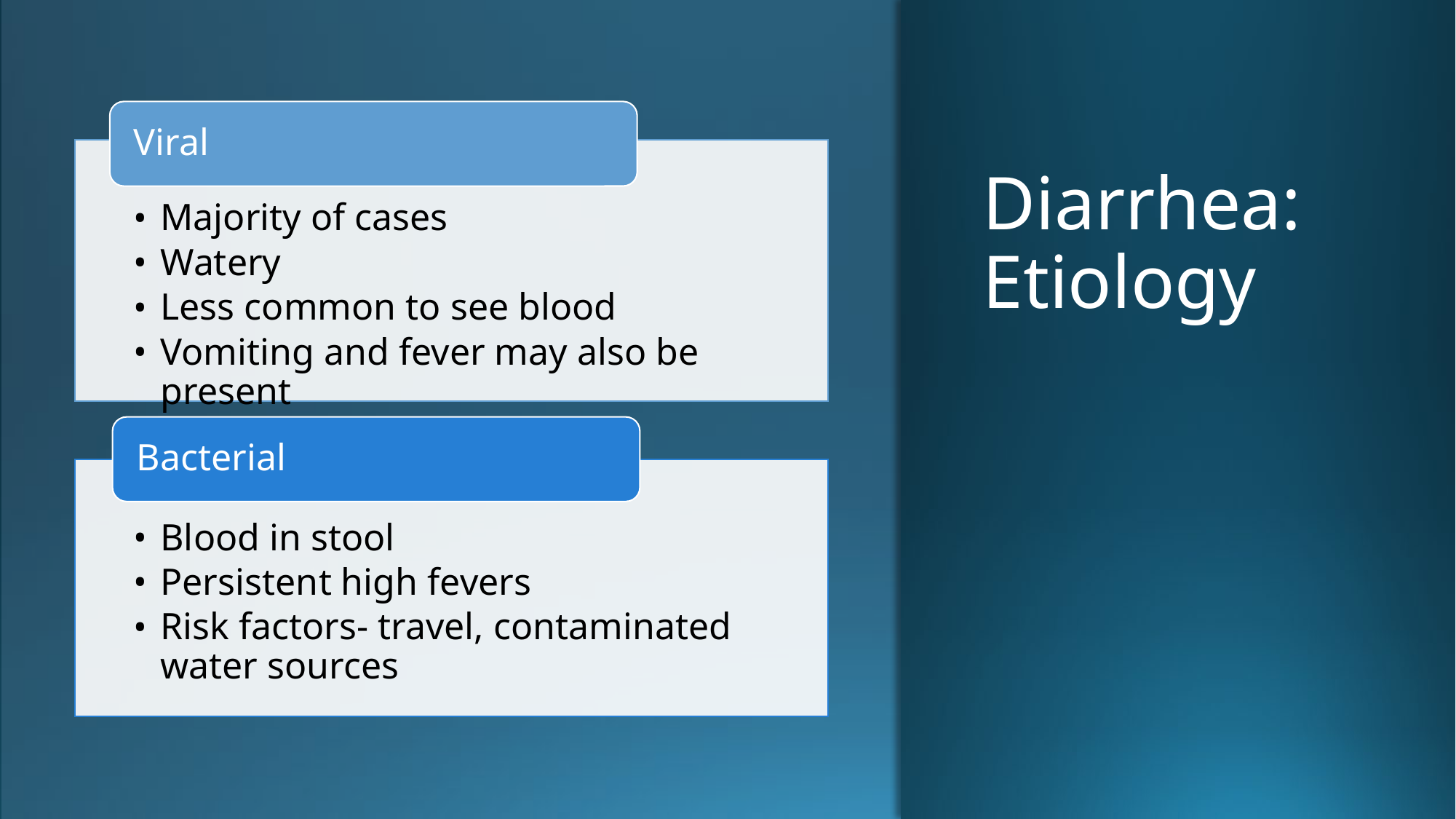

Viral
Majority of cases
Watery
Less common to see blood
Vomiting and fever may also be present
Bacterial
Blood in stool
Persistent high fevers
Risk factors- travel, contaminated water sources
# Diarrhea: Etiology

## Slide 7
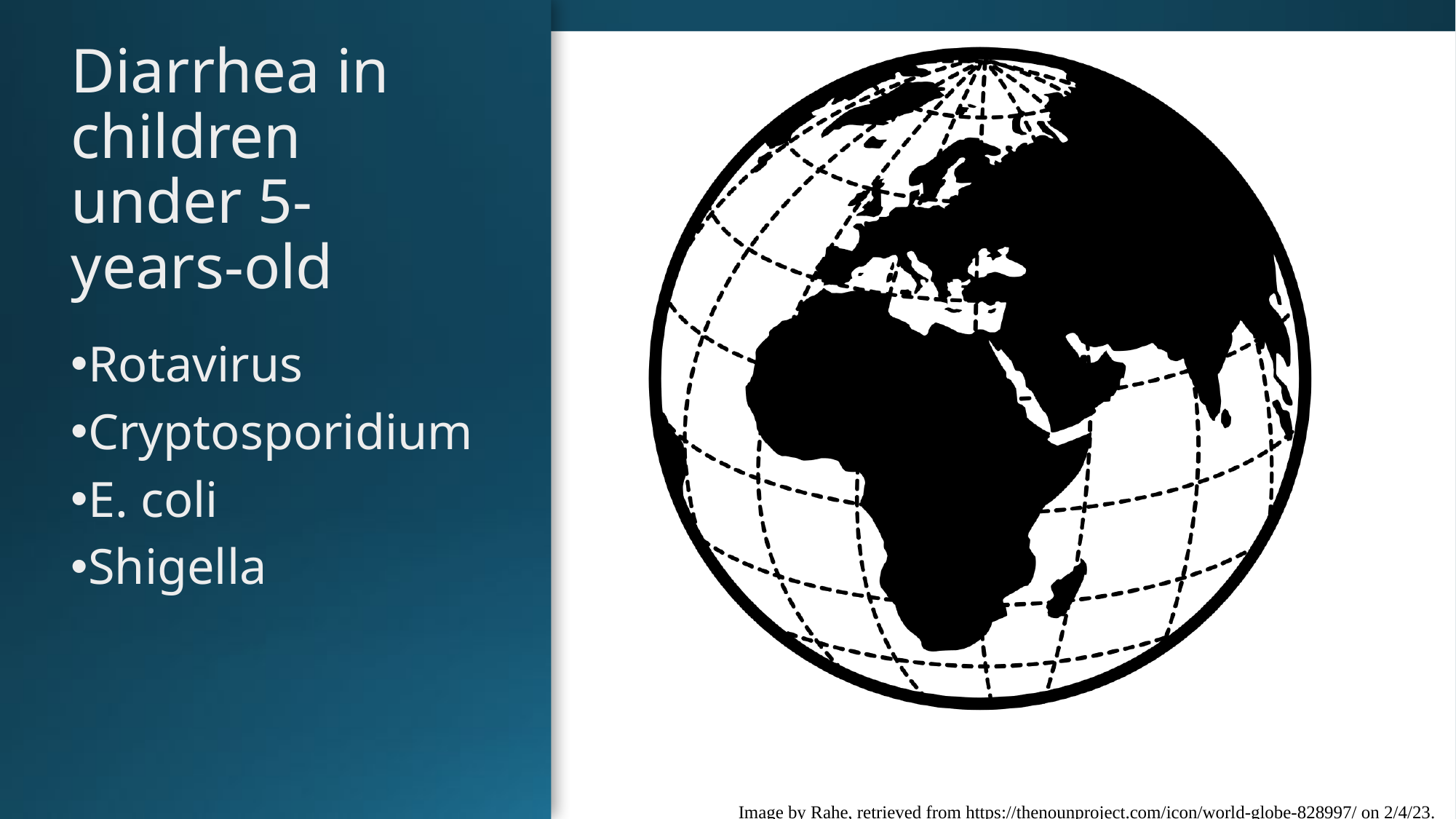

Diarrhea in children under 5-years-old
Rotavirus
Cryptosporidium
E. coli
Shigella
Image by Rahe, retrieved from https://thenounproject.com/icon/world-globe-828997/ on 2/4/23. Royalty-Free License

## Slide 8
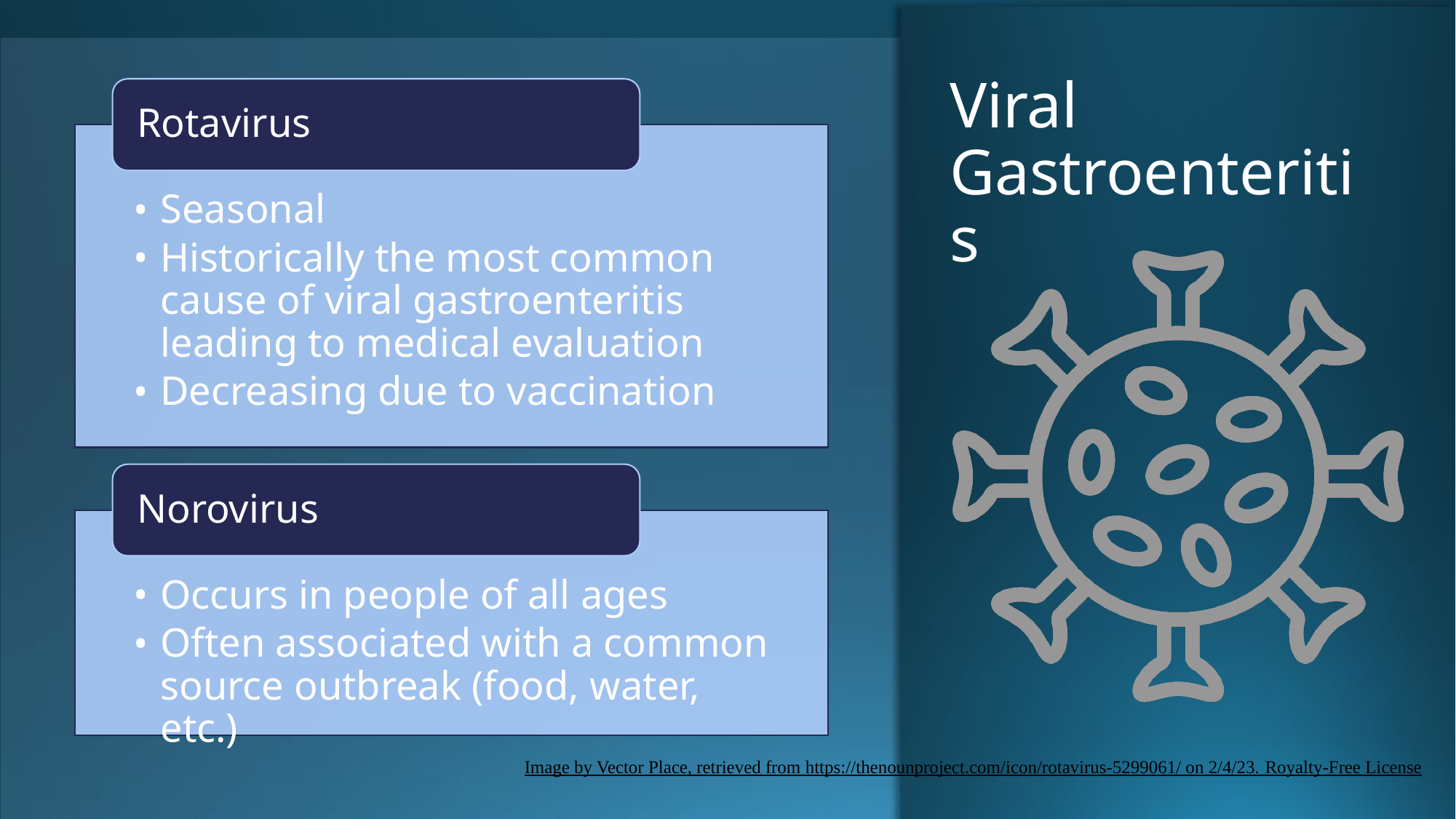

# Viral Gastroenteritis
Rotavirus
Seasonal
Historically the most common cause of viral gastroenteritis leading to medical evaluation
Decreasing due to vaccination
Norovirus
Occurs in people of all ages
Often associated with a common source outbreak (food, water, etc.)
Image by Vector Place, retrieved from https://thenounproject.com/icon/rotavirus-5299061/ on 2/4/23. Royalty-Free License

## Slide 9
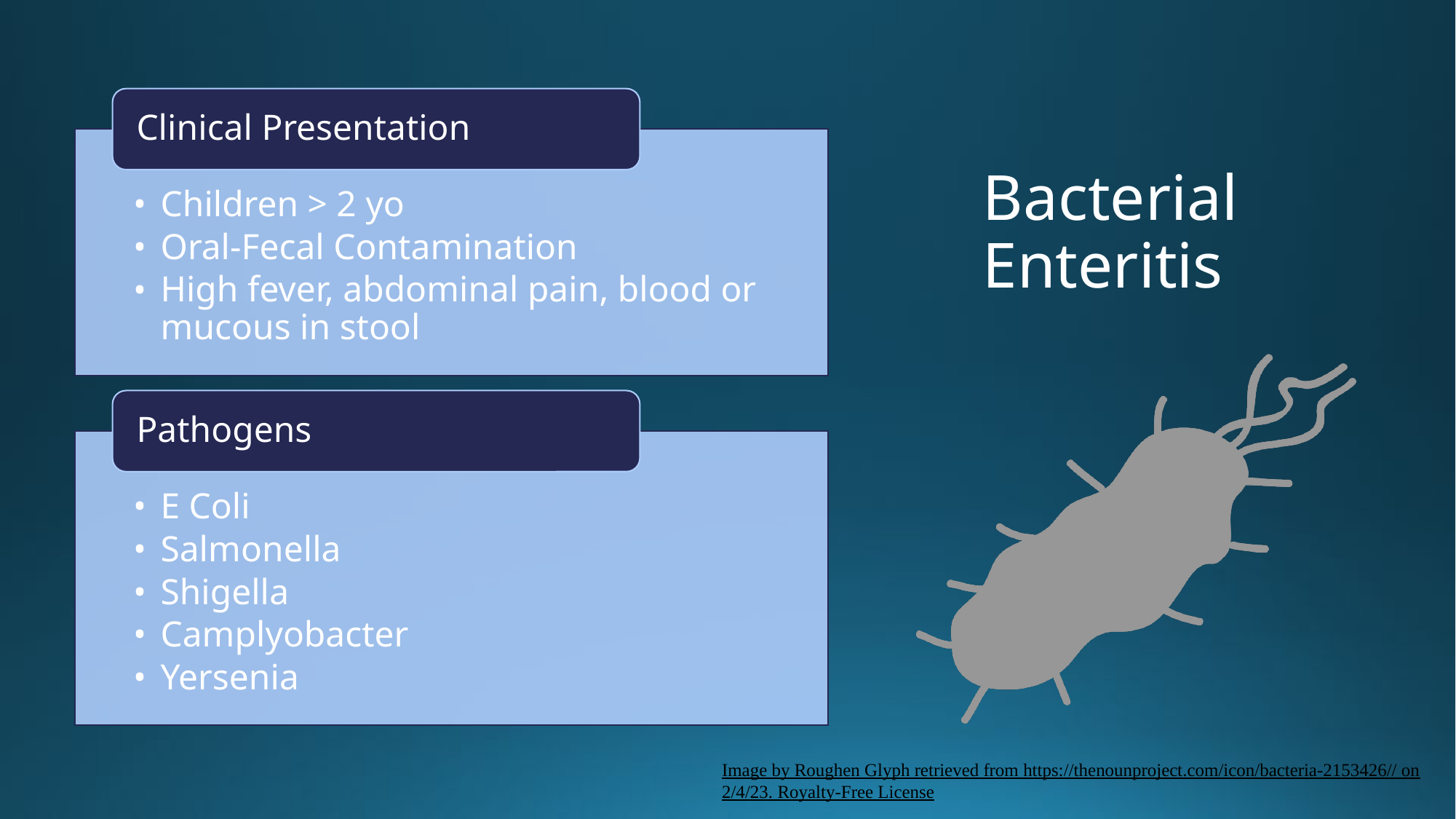

Clinical Presentation
Children > 2 yo
Oral-Fecal Contamination
High fever, abdominal pain, blood or mucous in stool
Pathogens
E Coli
Salmonella
Shigella
Camplyobacter
Yersenia
# Bacterial Enteritis
Image by Roughen Glyph retrieved from https://thenounproject.com/icon/bacteria-2153426// on 2/4/23. Royalty-Free License

## Slide 10
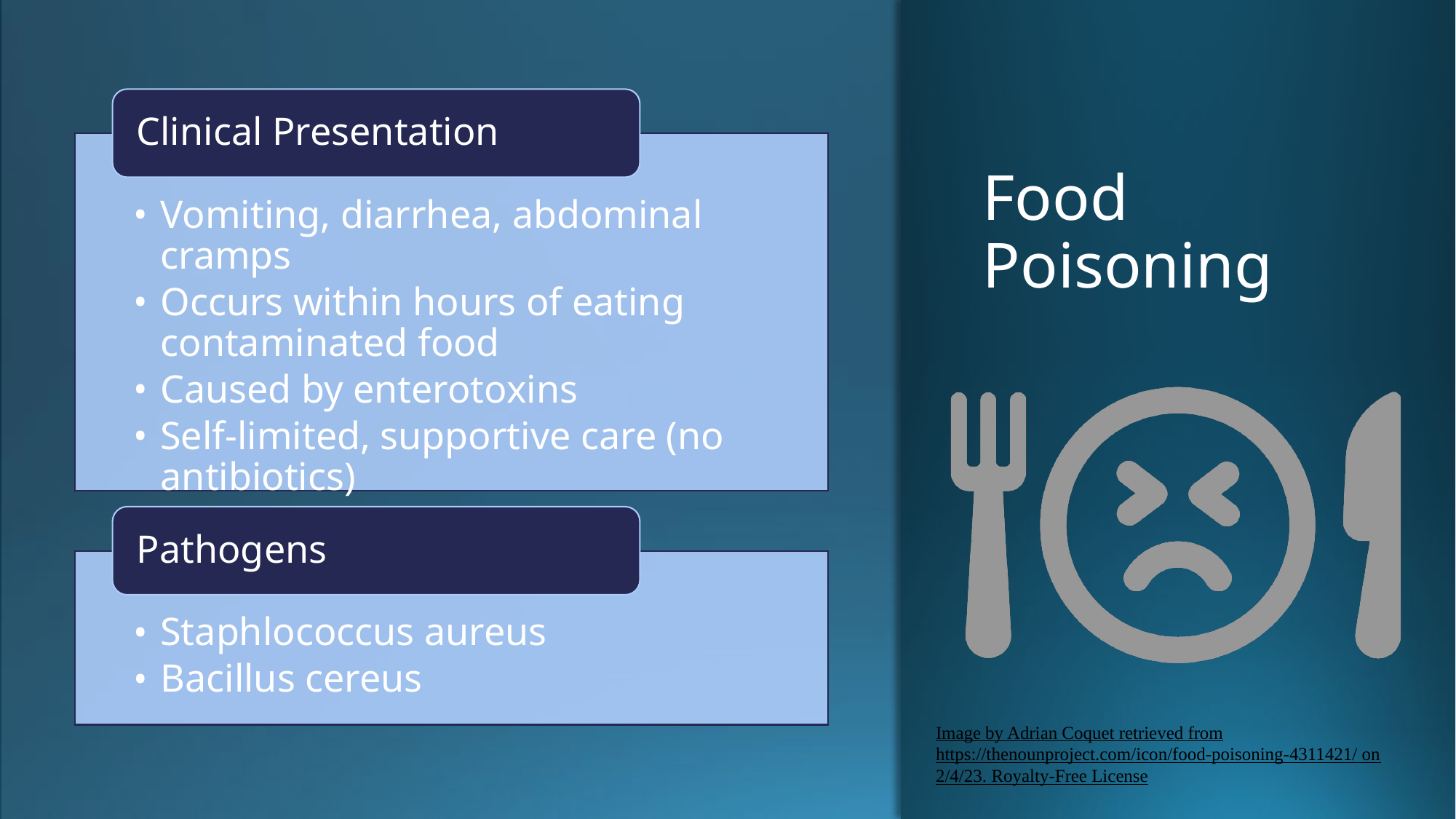

Clinical Presentation
Vomiting, diarrhea, abdominal cramps
Occurs within hours of eating contaminated food
Caused by enterotoxins
Self-limited, supportive care (no antibiotics)
Pathogens
Staphlococcus aureus
Bacillus cereus
# Food Poisoning
Image by Adrian Coquet retrieved from https://thenounproject.com/icon/food-poisoning-4311421/ on 2/4/23. Royalty-Free License

## Slide 11
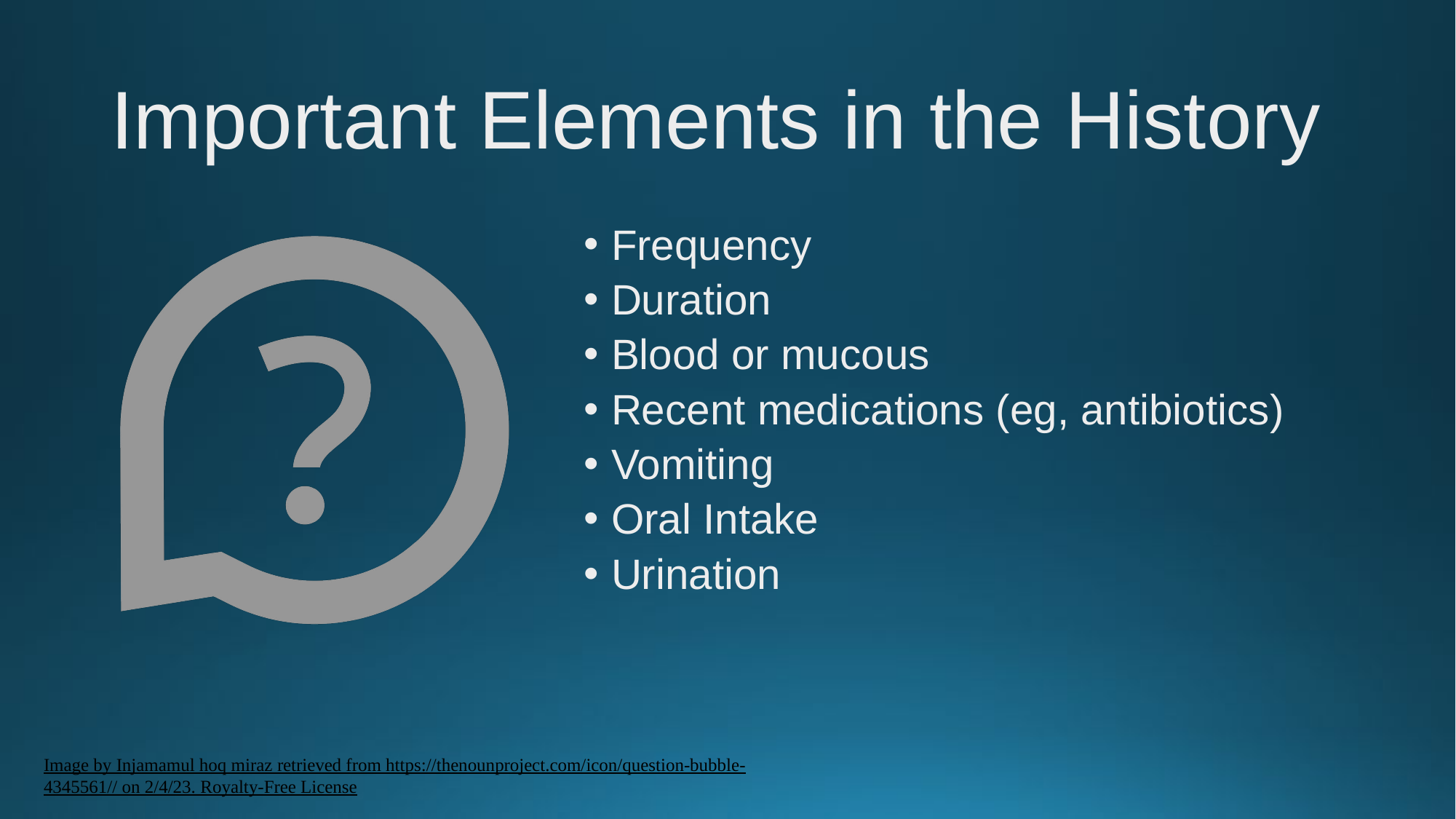

# Important Elements in the History
Frequency
Duration
Blood or mucous
Recent medications (eg, antibiotics)
Vomiting
Oral Intake
Urination
Image by Injamamul hoq miraz retrieved from https://thenounproject.com/icon/question-bubble-4345561// on 2/4/23. Royalty-Free License

## Slide 12
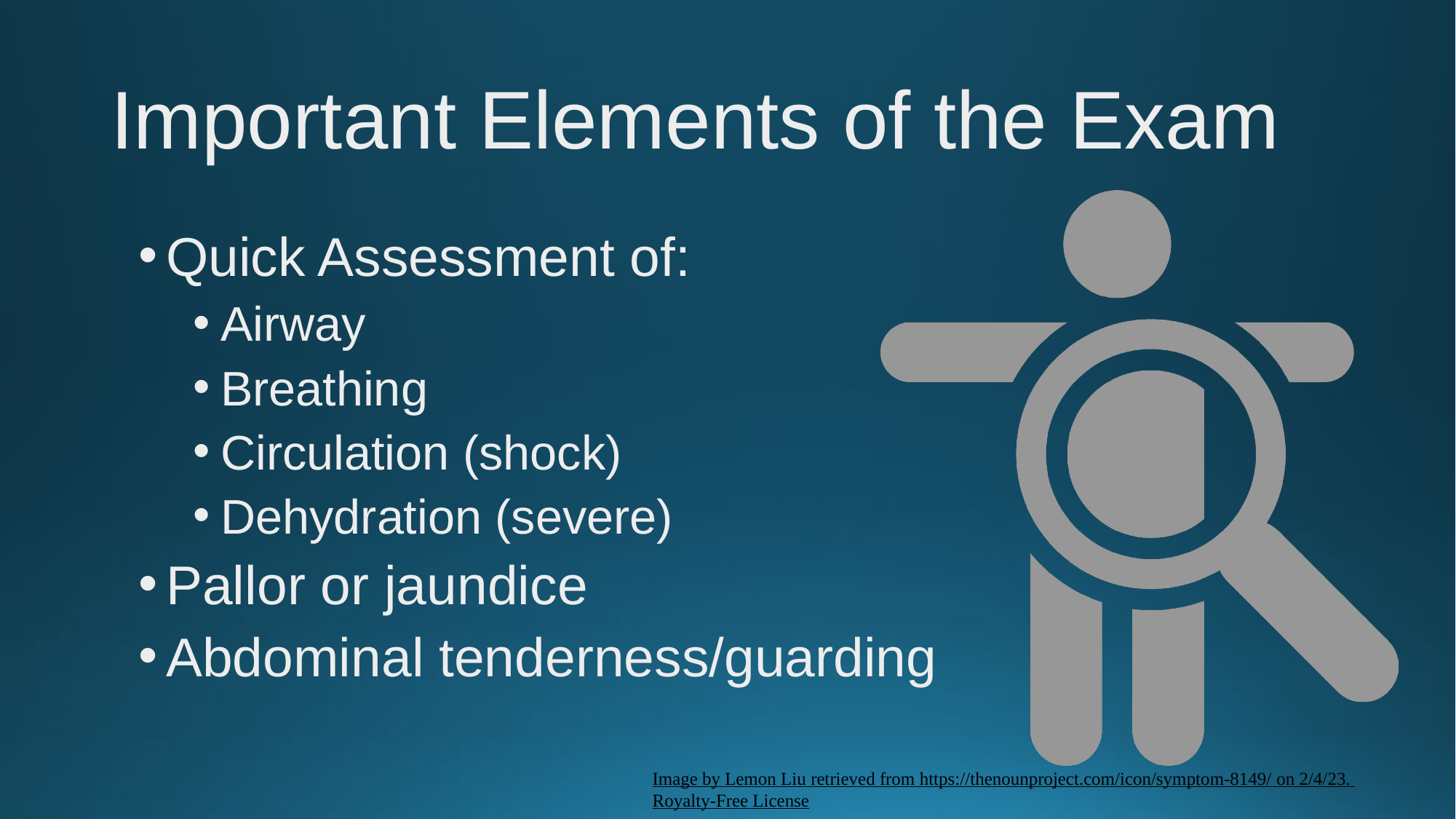

# Important Elements of the Exam
Quick Assessment of:
Airway
Breathing
Circulation (shock)
Dehydration (severe)
Pallor or jaundice
Abdominal tenderness/guarding
Image by Lemon Liu retrieved from https://thenounproject.com/icon/symptom-8149/ on 2/4/23. Royalty-Free License

## Slide 13
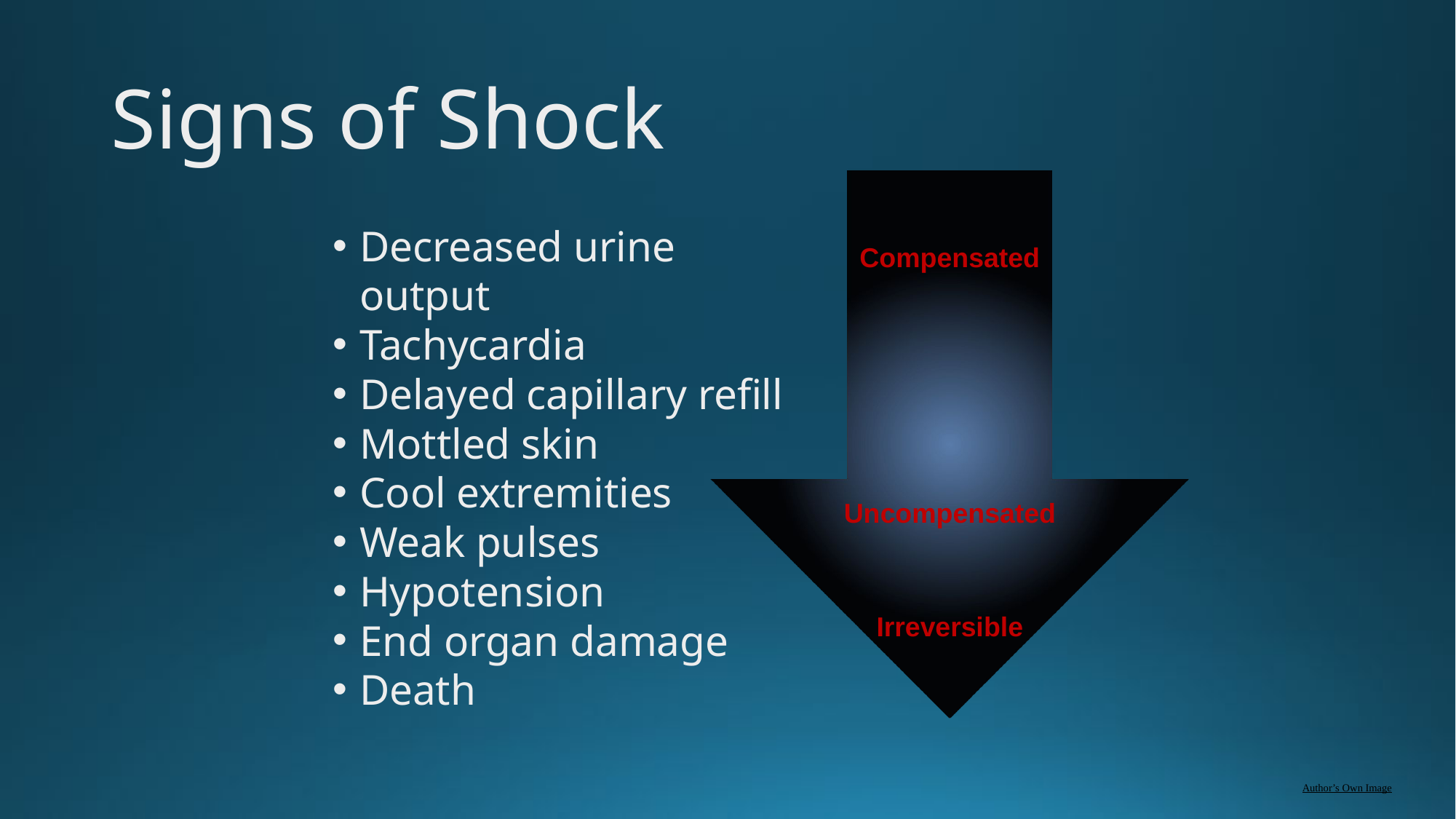

# Signs of Shock
Decreased urine output
Tachycardia
Delayed capillary refill
Mottled skin
Cool extremities
Weak pulses
Hypotension
End organ damage
Death
Compensated
Uncompensated
Irreversible
Author’s Own Image

## Slide 14
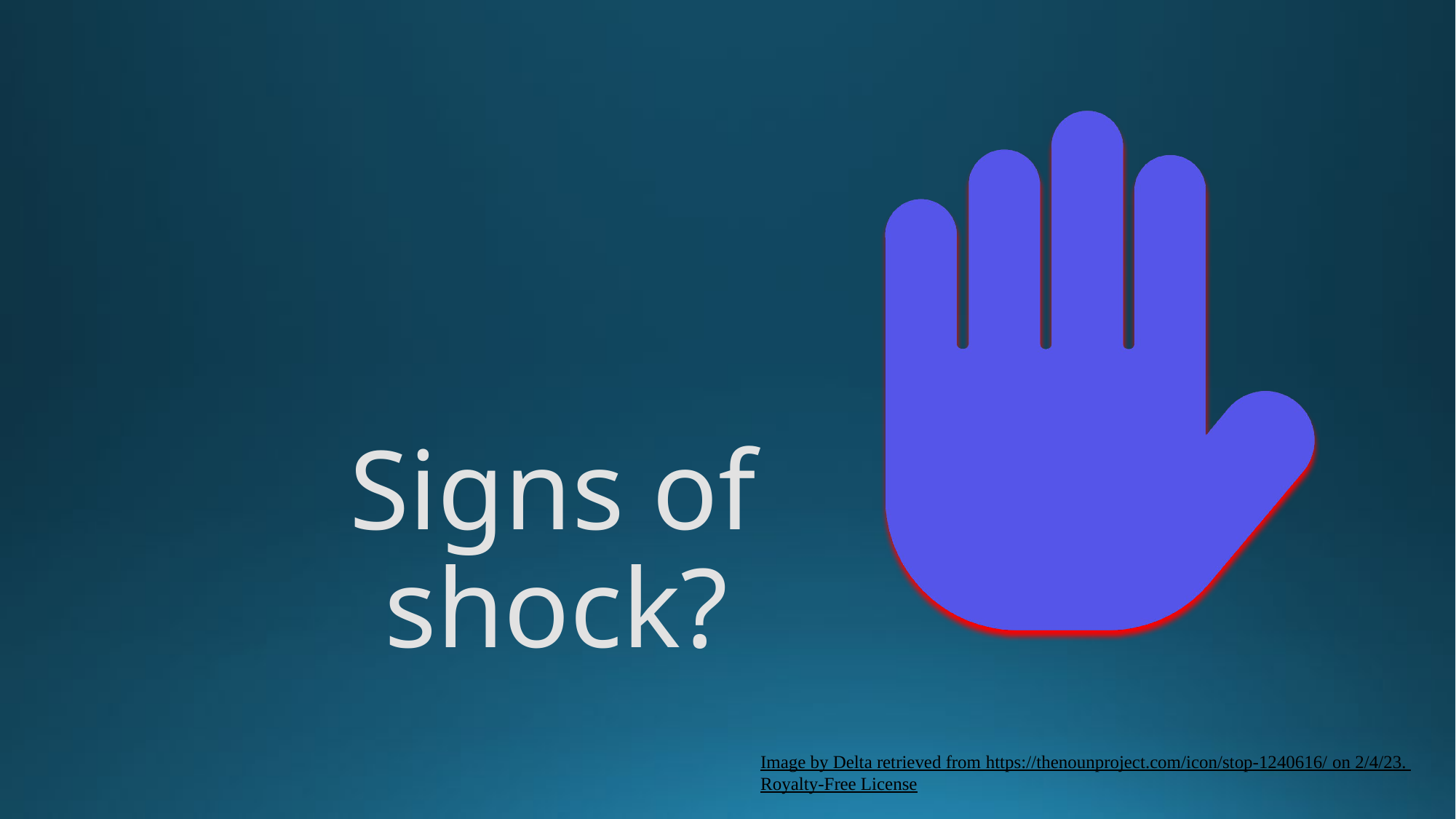

# Signs of shock?
Image by Delta retrieved from https://thenounproject.com/icon/stop-1240616/ on 2/4/23. Royalty-Free License

## Slide 15
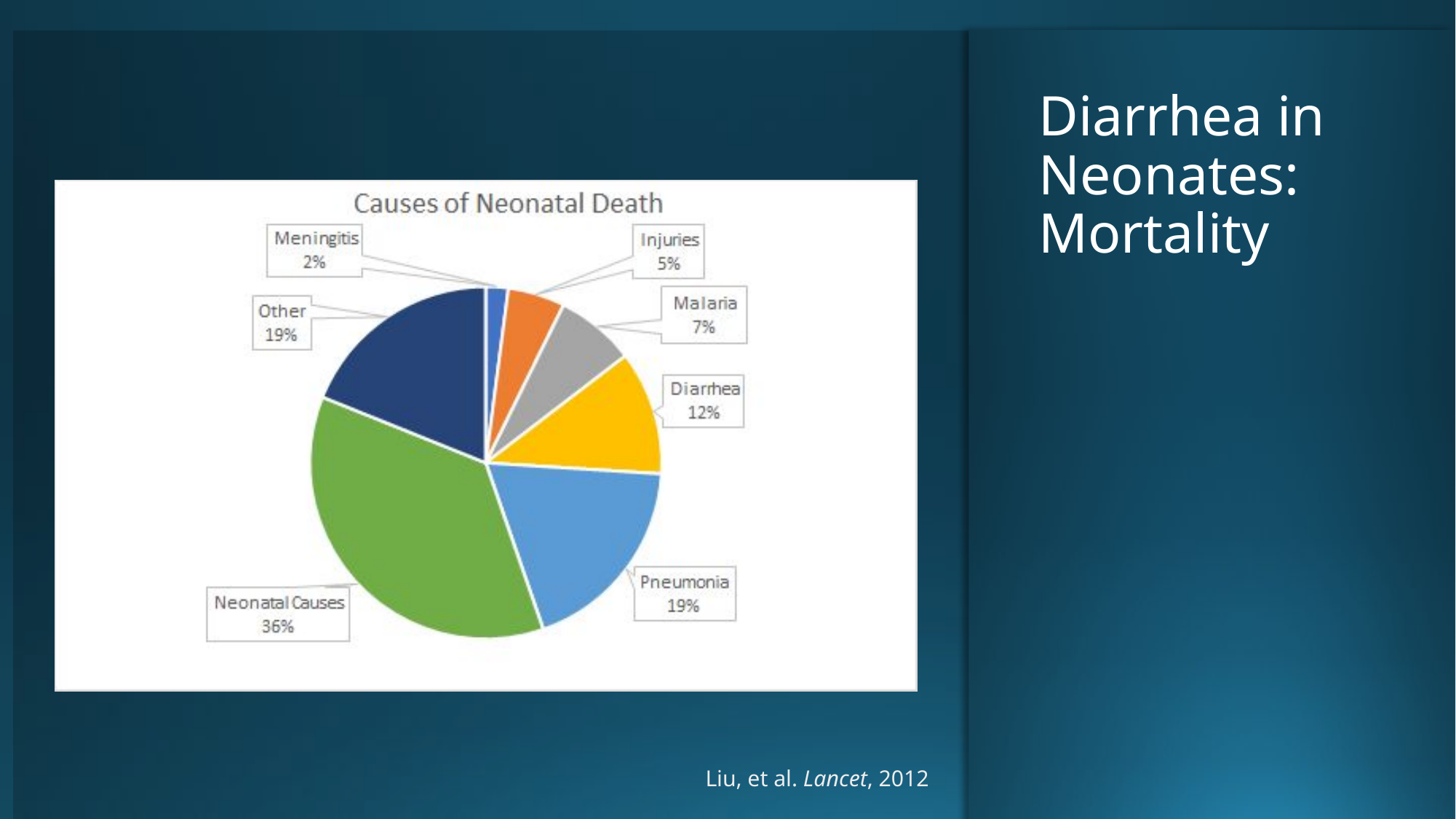

# Diarrhea in Neonates: Mortality
Liu, et al. Lancet, 2012

## Slide 16
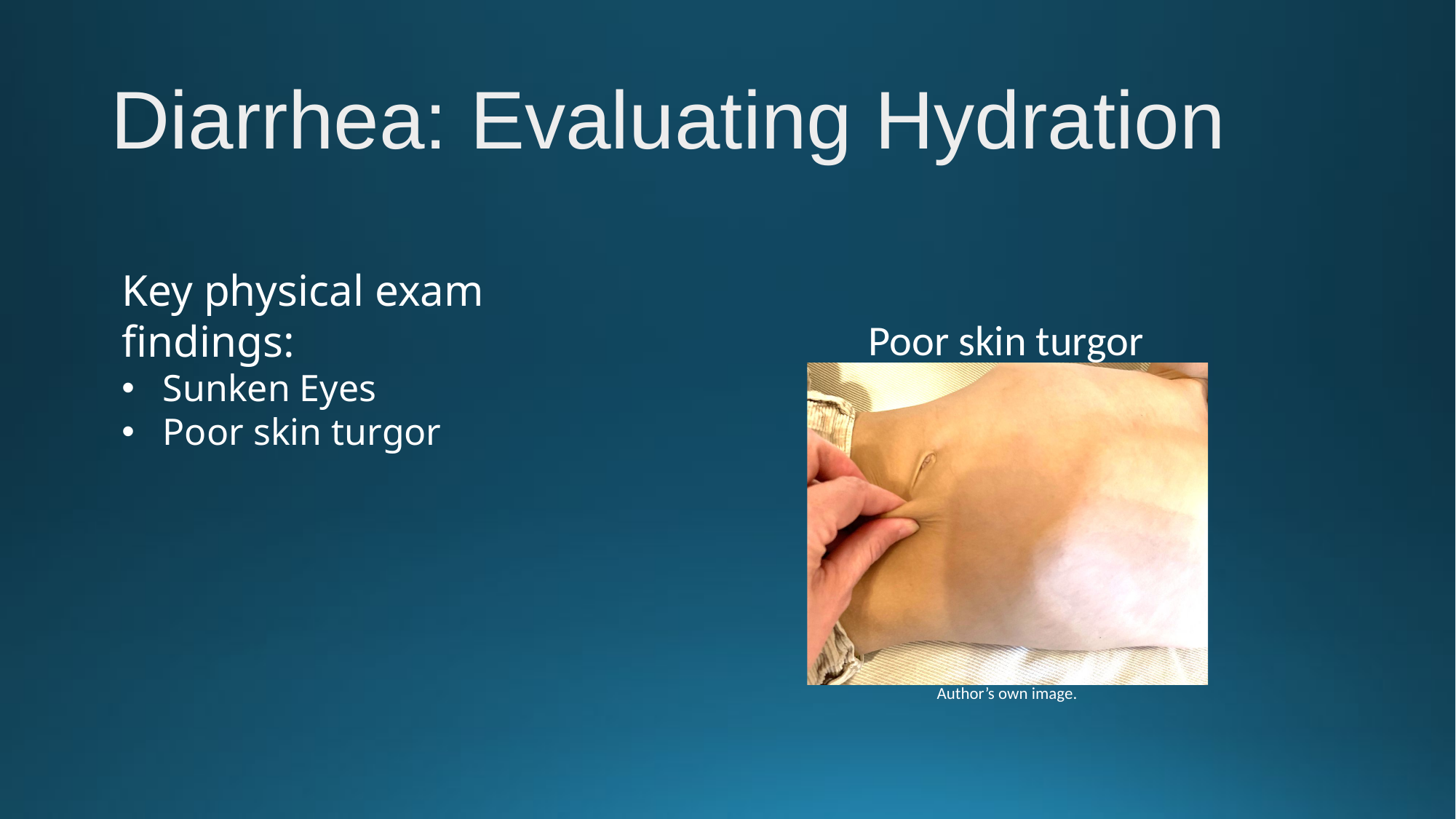

# Diarrhea: Evaluating Hydration
Key physical exam findings:
Sunken Eyes
Poor skin turgor
Poor skin turgor
Author’s own image.

## Slide 17
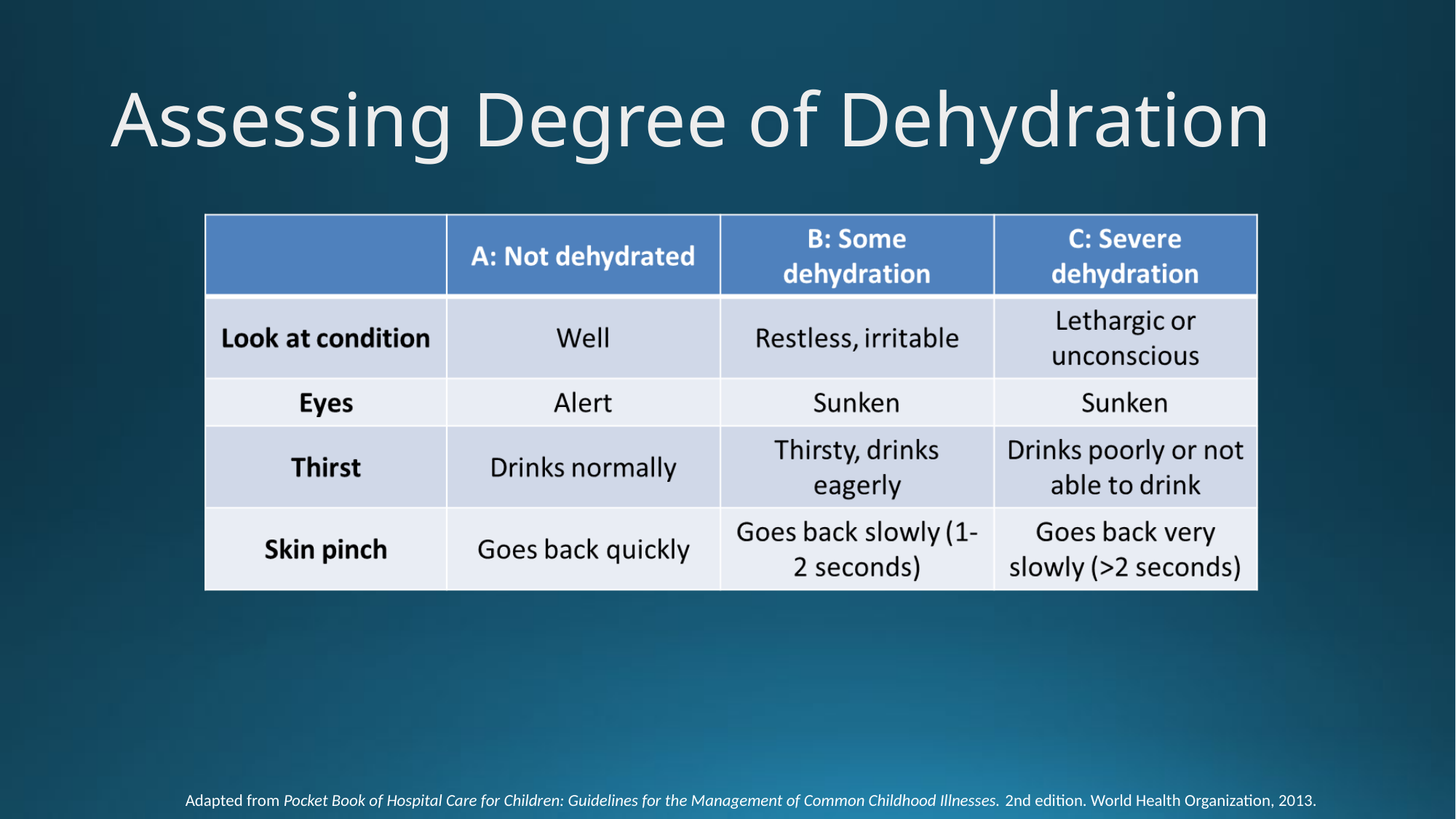

# Assessing Degree of Dehydration
Adapted from Pocket Book of Hospital Care for Children: Guidelines for the Management of Common Childhood Illnesses. 2nd edition. World Health Organization, 2013.

## Slide 18
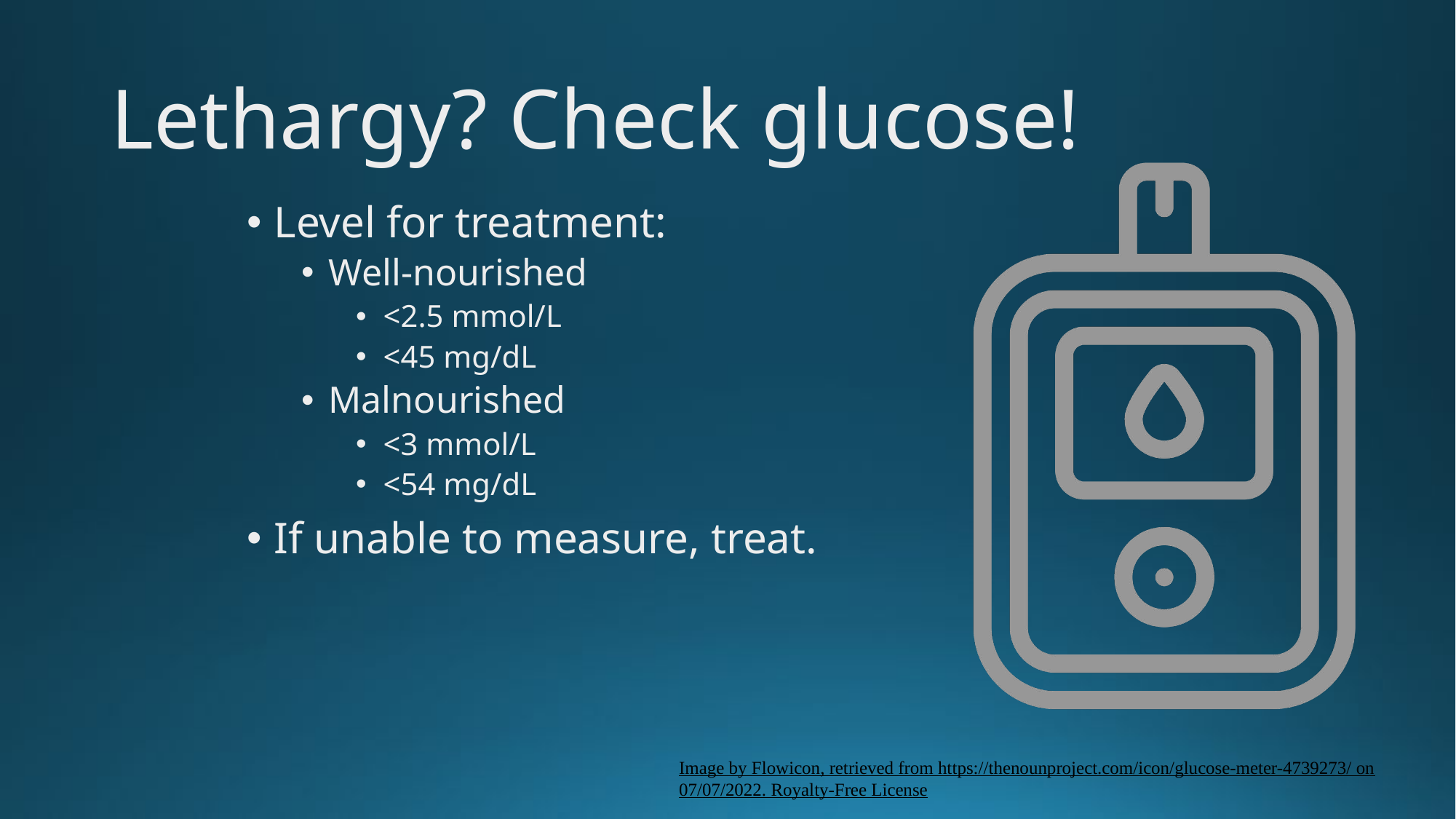

# Lethargy? Check glucose!
Level for treatment:
Well-nourished
<2.5 mmol/L
<45 mg/dL
Malnourished
<3 mmol/L
<54 mg/dL
If unable to measure, treat.
Image by Flowicon, retrieved from https://thenounproject.com/icon/glucose-meter-4739273/ on 07/07/2022. Royalty-Free License

## Slide 19
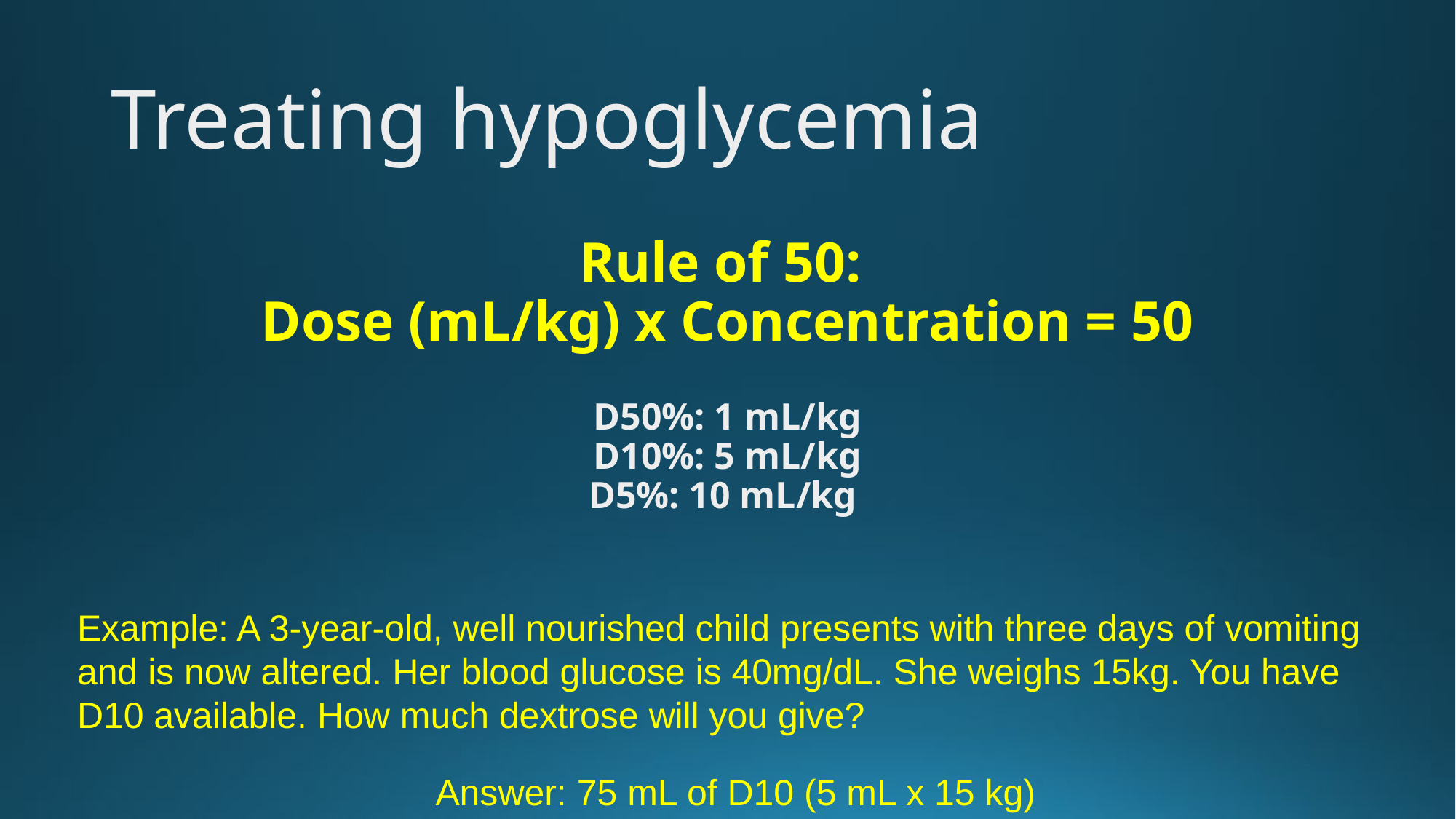

# Treating hypoglycemia
Rule of 50:
Dose (mL/kg) x Concentration = 50
D50%: 1 mL/kg
D10%: 5 mL/kg
D5%: 10 mL/kg
Example: A 3-year-old, well nourished child presents with three days of vomiting and is now altered. Her blood glucose is 40mg/dL. She weighs 15kg. You have D10 available. How much dextrose will you give?
Answer: 75 mL of D10 (5 mL x 15 kg)

## Slide 20
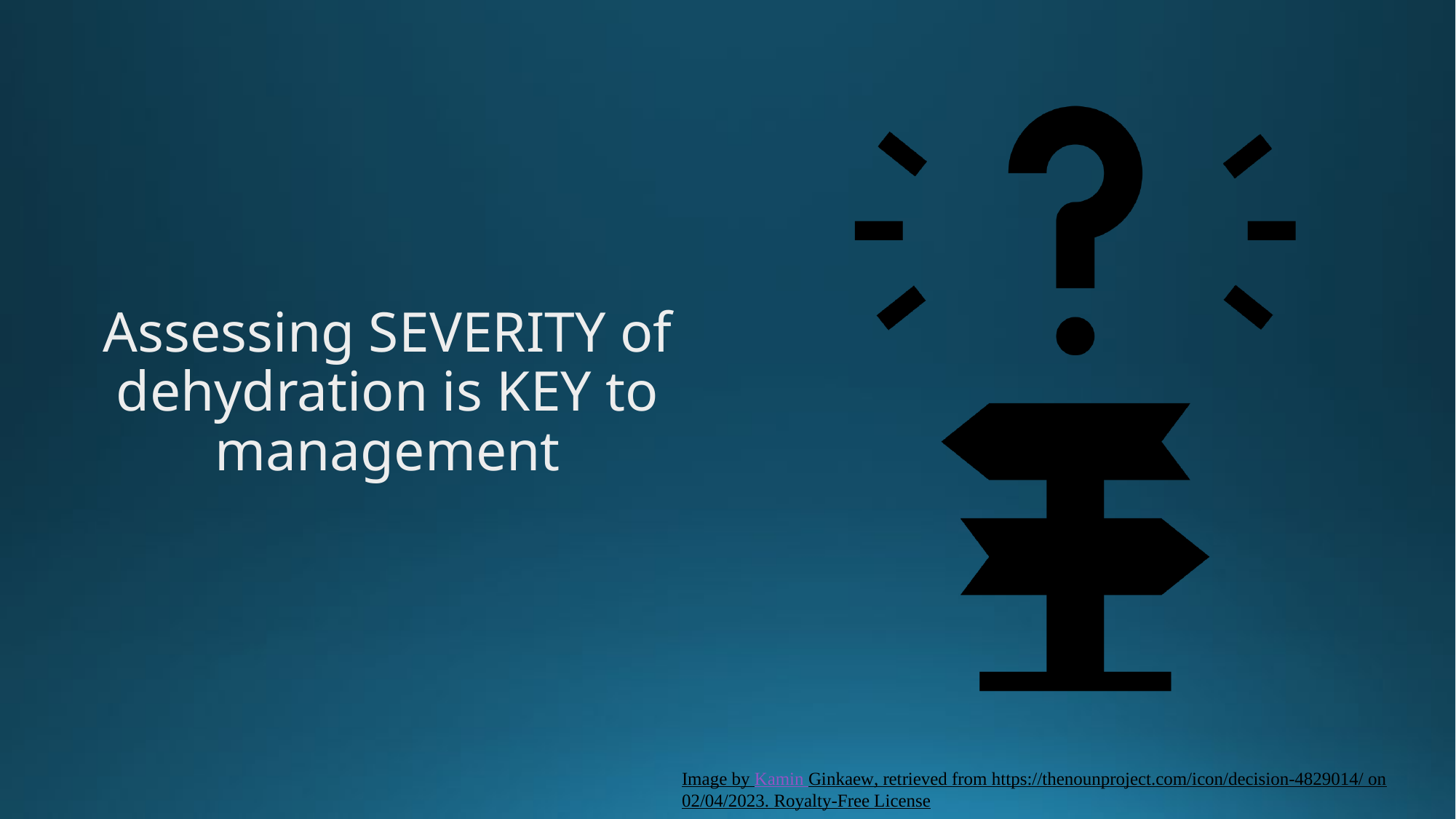

# Assessing SEVERITY of dehydration is KEY to management
Image by Kamin Ginkaew, retrieved from https://thenounproject.com/icon/decision-4829014/ on 02/04/2023. Royalty-Free License

## Slide 21
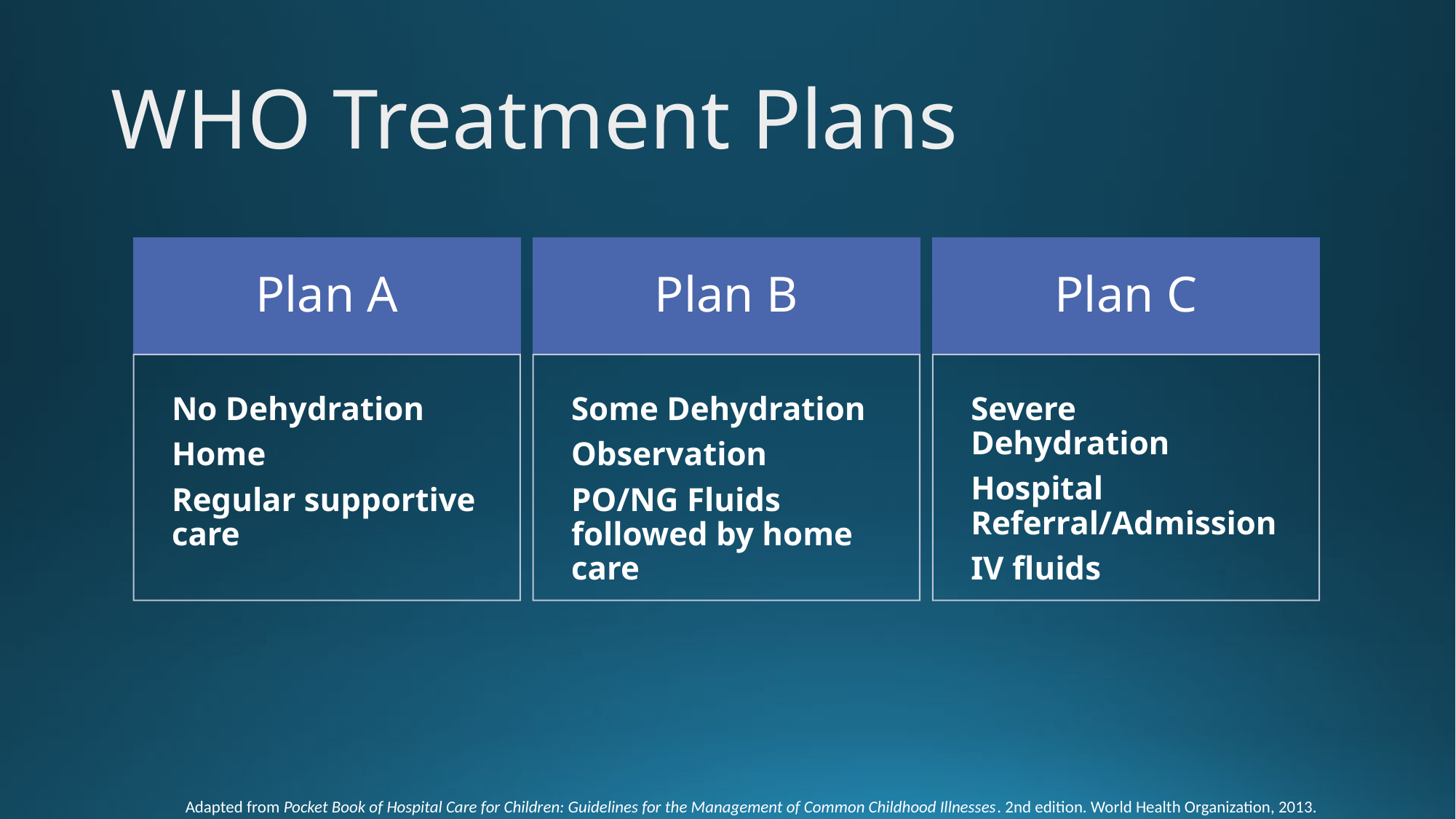

# WHO Treatment Plans
Plan A
Plan B
Plan C
No Dehydration
Home
Regular supportive care
Some Dehydration
Observation
PO/NG Fluids followed by home care
Severe Dehydration
Hospital Referral/Admission
IV fluids
Adapted from Pocket Book of Hospital Care for Children: Guidelines for the Management of Common Childhood Illnesses. 2nd edition. World Health Organization, 2013.

## Slide 22
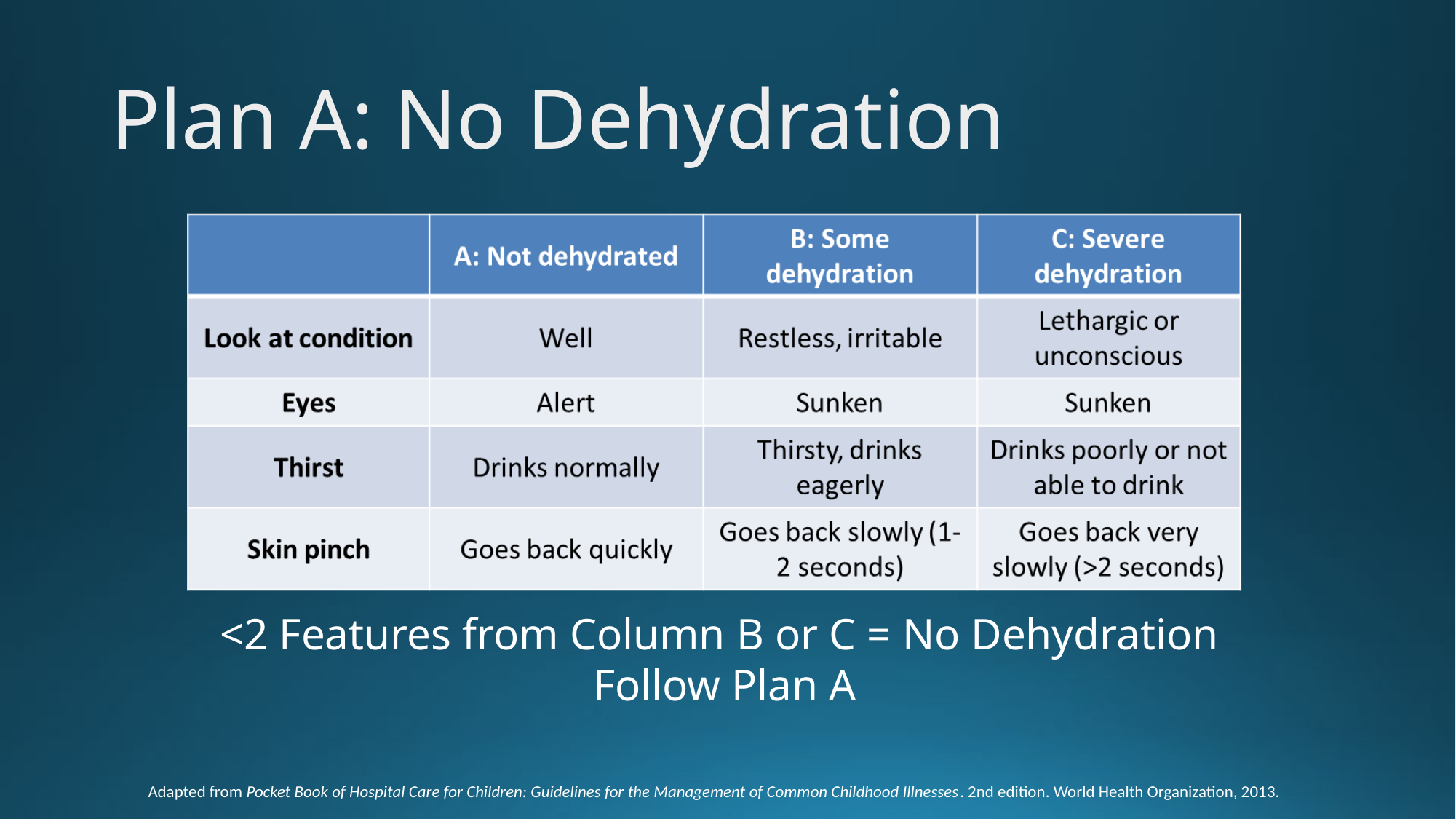

# Plan A: No Dehydration
<2 Features from Column B or C = No Dehydration
 Follow Plan A
Adapted from Pocket Book of Hospital Care for Children: Guidelines for the Management of Common Childhood Illnesses. 2nd edition. World Health Organization, 2013.

## Slide 23
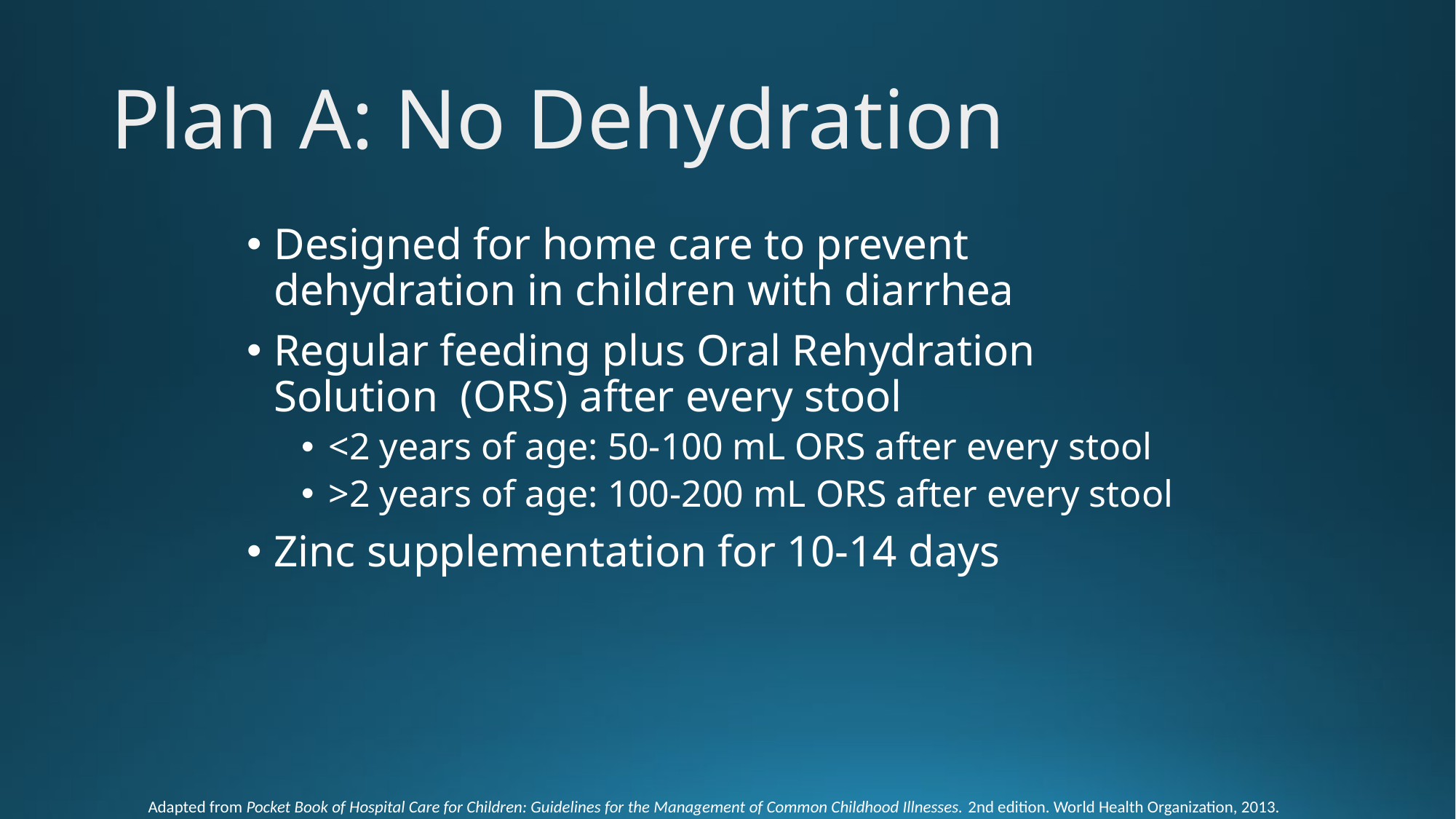

# Plan A: No Dehydration
Designed for home care to prevent dehydration in children with diarrhea
Regular feeding plus Oral Rehydration Solution (ORS) after every stool
<2 years of age: 50-100 mL ORS after every stool
>2 years of age: 100-200 mL ORS after every stool
Zinc supplementation for 10-14 days
Adapted from Pocket Book of Hospital Care for Children: Guidelines for the Management of Common Childhood Illnesses. 2nd edition. World Health Organization, 2013.

## Slide 24
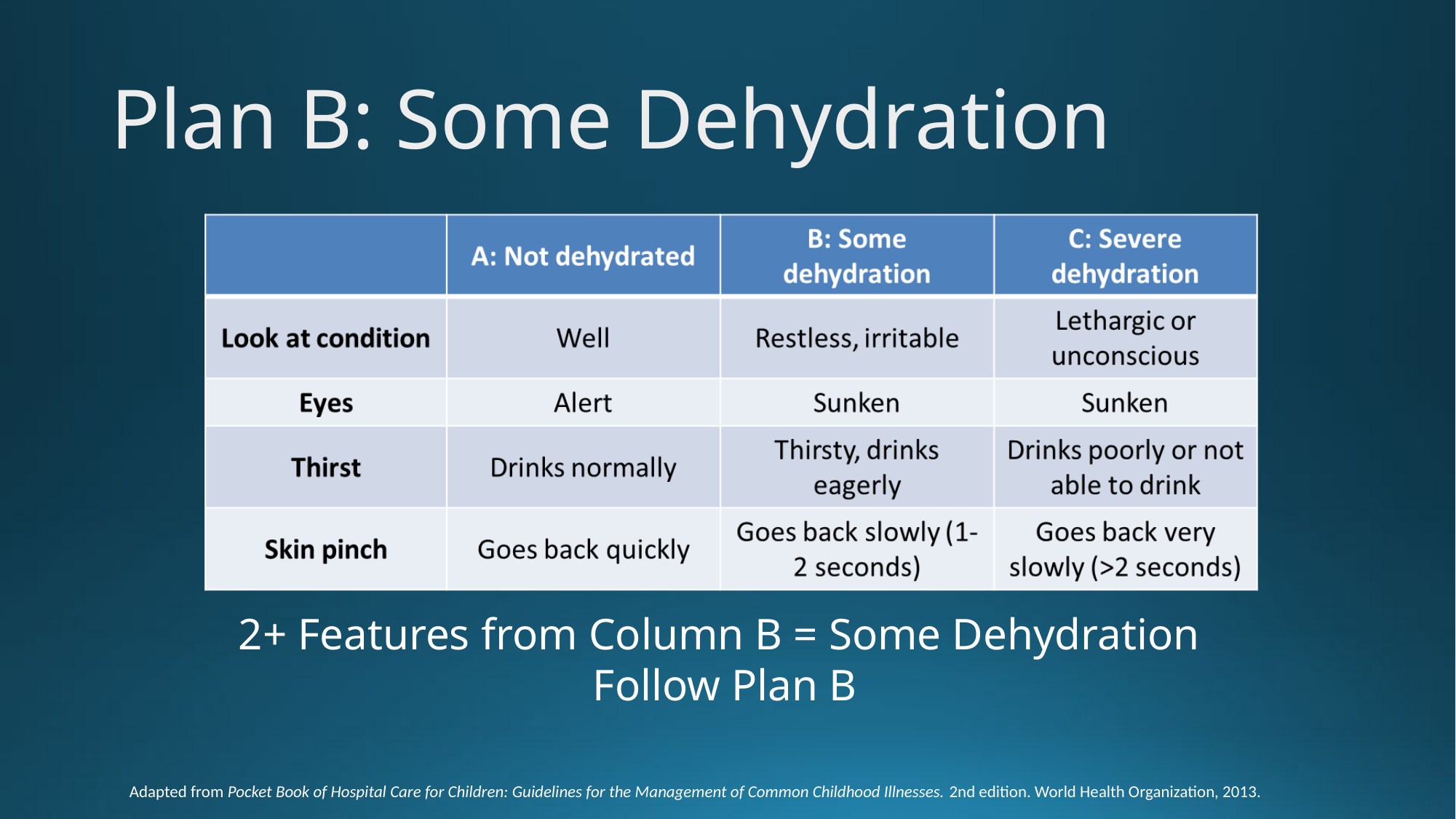

# Plan B: Some Dehydration
2+ Features from Column B = Some Dehydration
 Follow Plan B
Adapted from Pocket Book of Hospital Care for Children: Guidelines for the Management of Common Childhood Illnesses. 2nd edition. World Health Organization, 2013.

## Slide 25
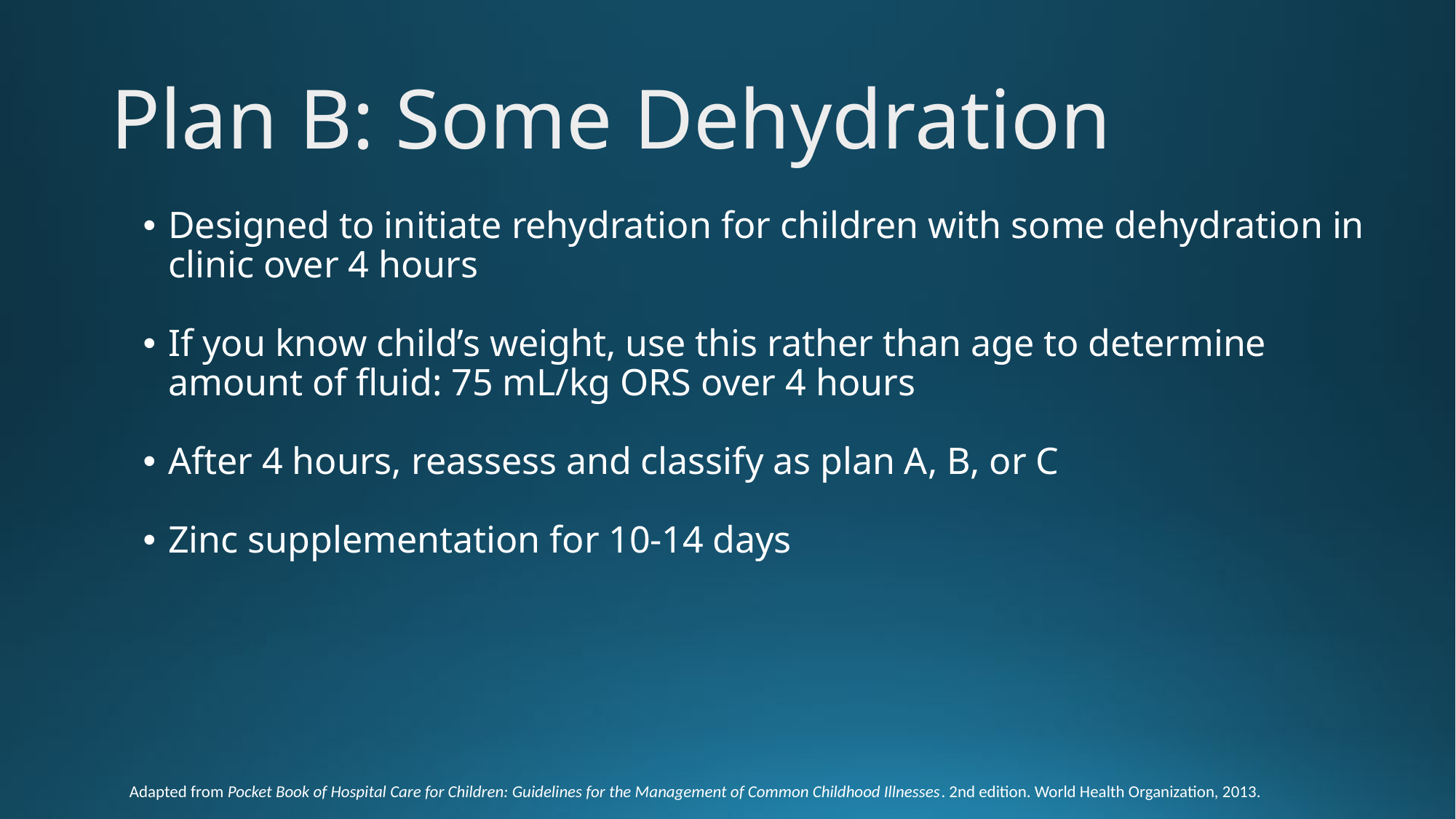

# Plan B: Some Dehydration
Designed to initiate rehydration for children with some dehydration in clinic over 4 hours
If you know child’s weight, use this rather than age to determine amount of fluid: 75 mL/kg ORS over 4 hours
After 4 hours, reassess and classify as plan A, B, or C
Zinc supplementation for 10-14 days
Adapted from Pocket Book of Hospital Care for Children: Guidelines for the Management of Common Childhood Illnesses. 2nd edition. World Health Organization, 2013.

## Slide 26
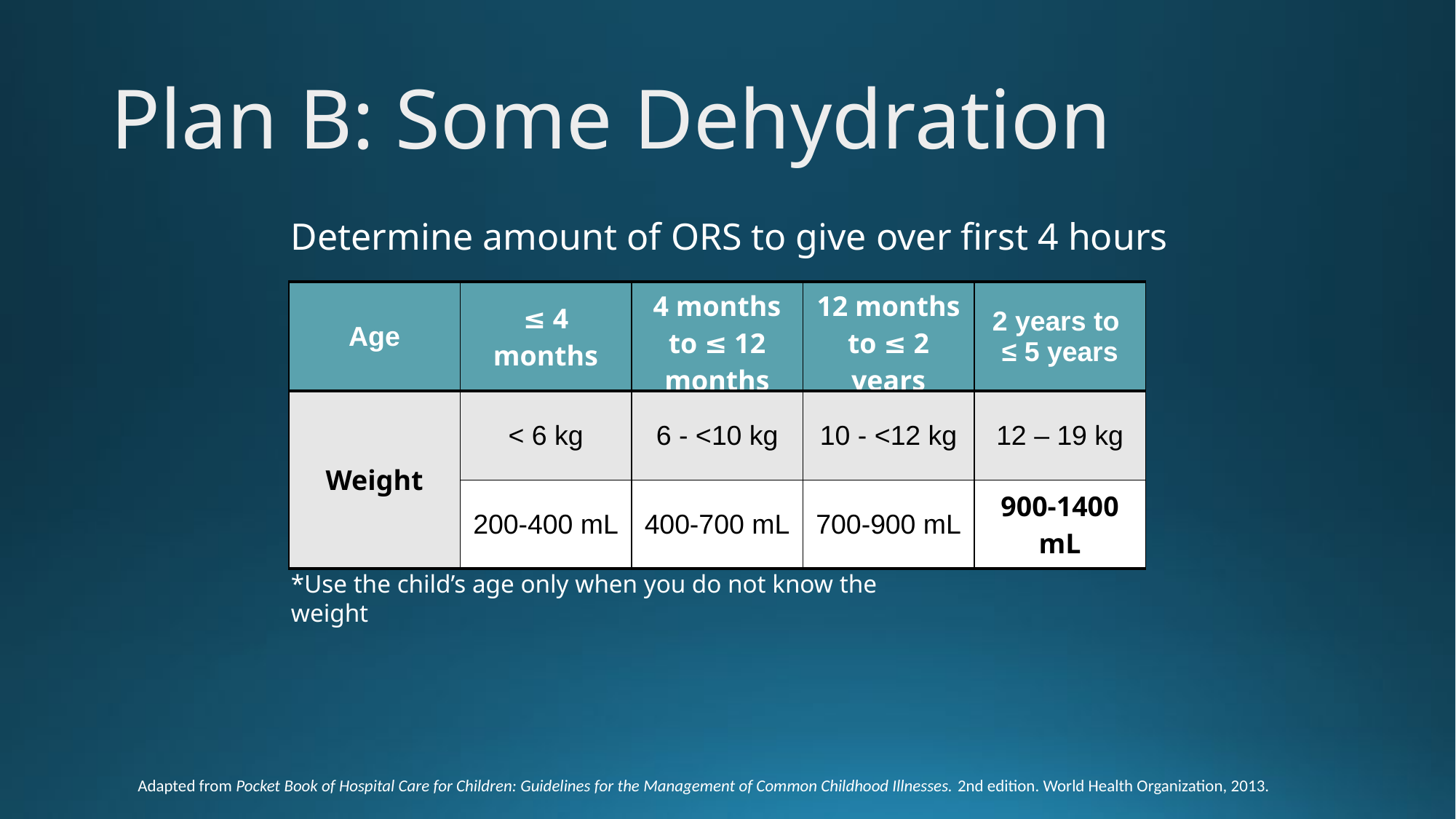

# Plan B: Some Dehydration
Determine amount of ORS to give over first 4 hours
| Age | ≤ 4 months | 4 months to ≤ 12 months | 12 months to ≤ 2 years | 2 years to ≤ 5 years |
| --- | --- | --- | --- | --- |
| Weight | < 6 kg | 6 - <10 kg | 10 - <12 kg | 12 – 19 kg |
| | 200-400 mL | 400-700 mL | 700-900 mL | 900-1400 mL |
*Use the child’s age only when you do not know the weight
Adapted from Pocket Book of Hospital Care for Children: Guidelines for the Management of Common Childhood Illnesses. 2nd edition. World Health Organization, 2013.

## Slide 27
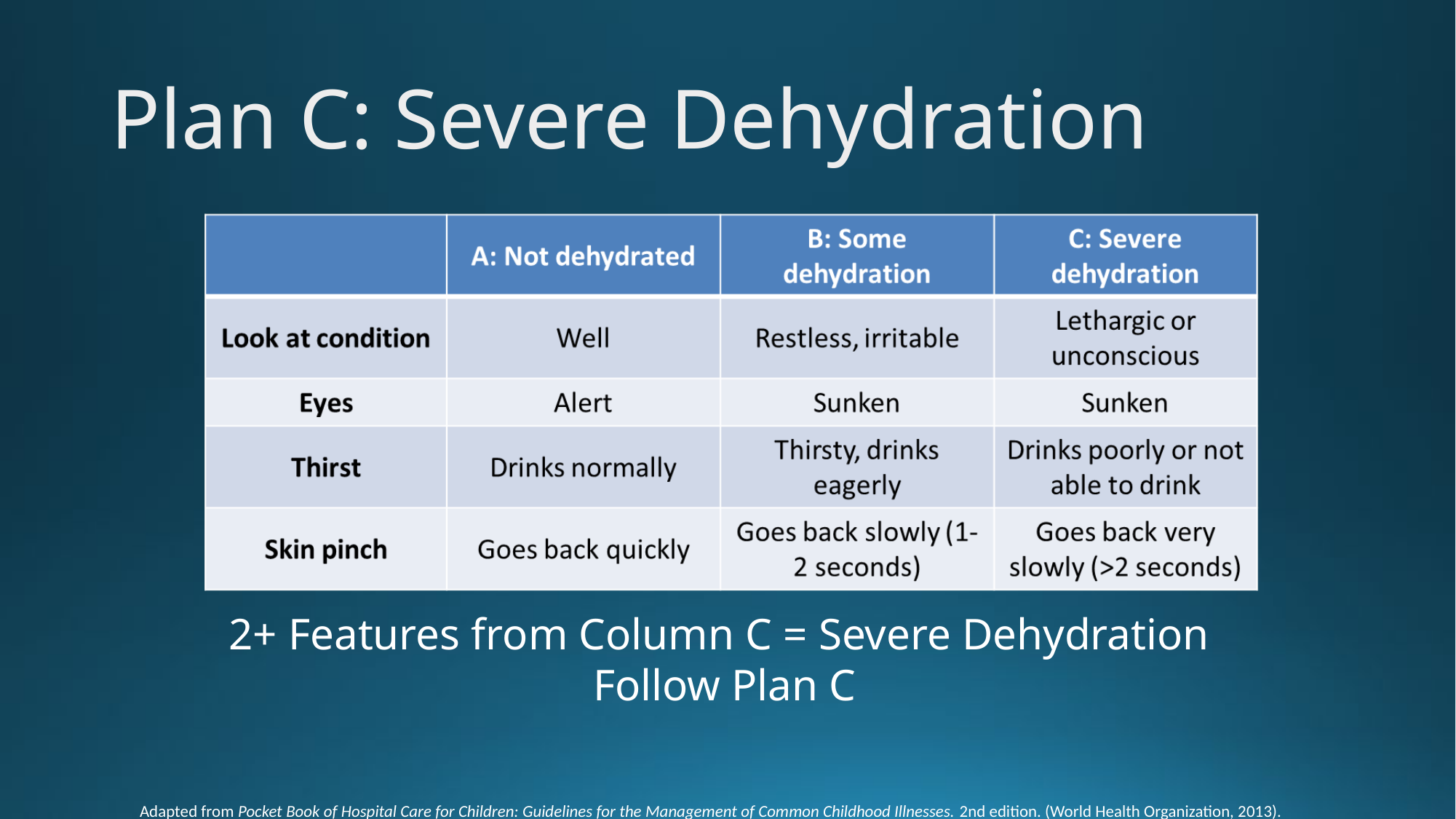

# Plan C: Severe Dehydration
2+ Features from Column C = Severe Dehydration
 Follow Plan C
Adapted from Pocket Book of Hospital Care for Children: Guidelines for the Management of Common Childhood Illnesses. 2nd edition. (World Health Organization, 2013).

## Slide 28
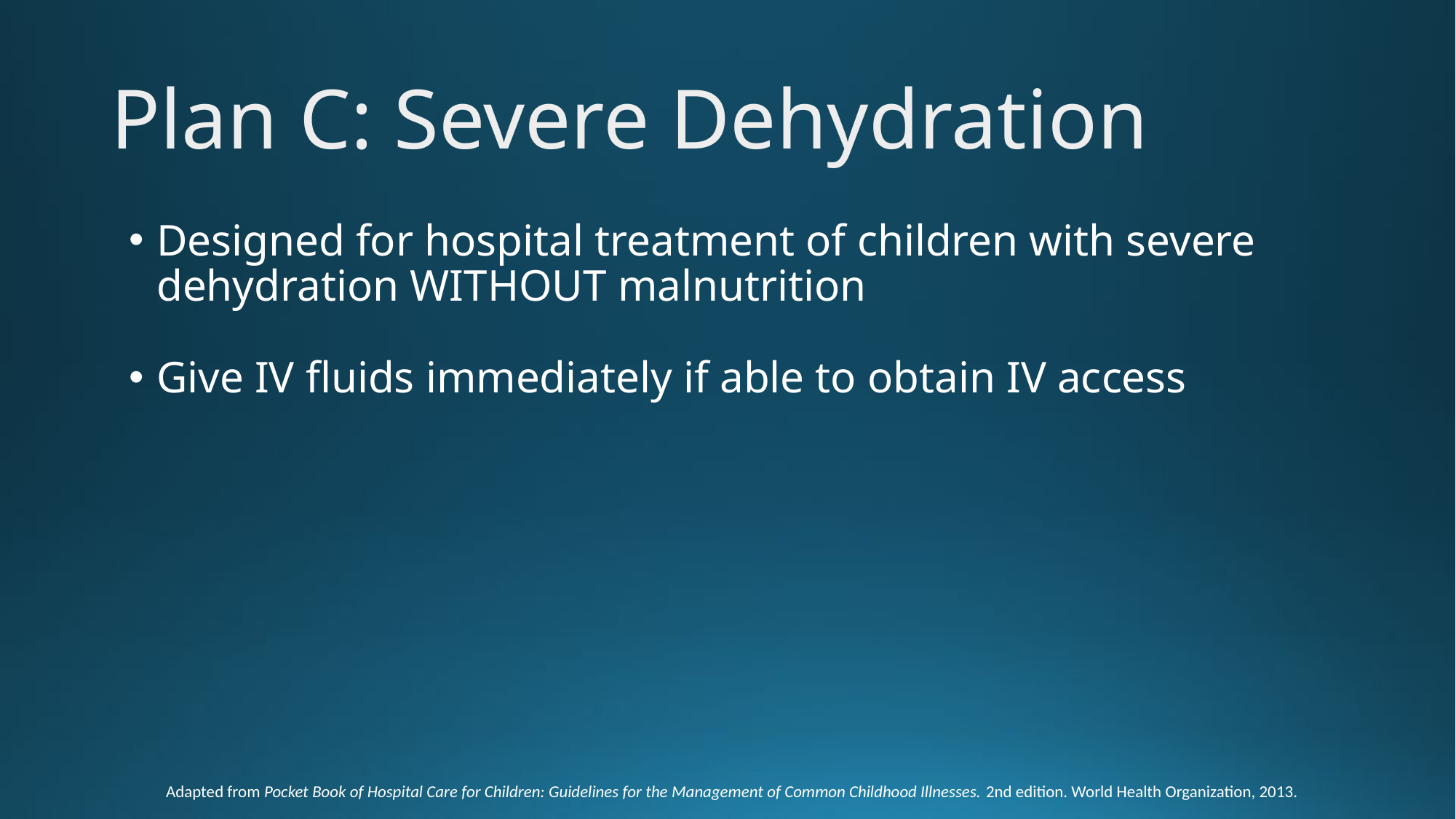

# Plan C: Severe Dehydration
Designed for hospital treatment of children with severe dehydration WITHOUT malnutrition
Give IV fluids immediately if able to obtain IV access
Adapted from Pocket Book of Hospital Care for Children: Guidelines for the Management of Common Childhood Illnesses. 2nd edition. World Health Organization, 2013.

## Slide 29
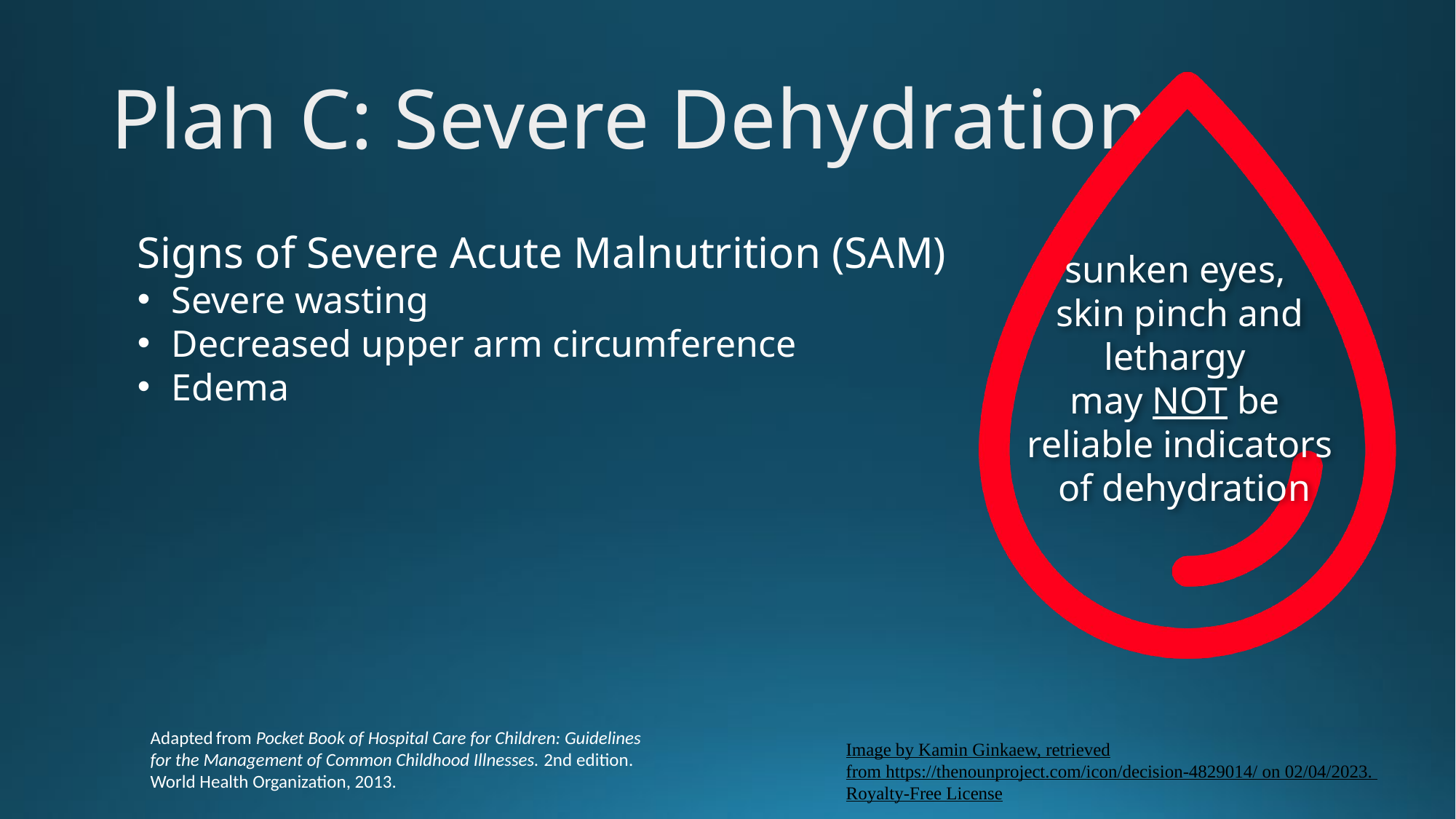

# Plan C: Severe Dehydration
Signs of Severe Acute Malnutrition (SAM)
Severe wasting
Decreased upper arm circumference
Edema
sunken eyes,
skin pinch and lethargy
may NOT be
reliable indicators
 of dehydration
Adapted from Pocket Book of Hospital Care for Children: Guidelines for the Management of Common Childhood Illnesses. 2nd edition. World Health Organization, 2013.
Image by Kamin Ginkaew, retrieved from https://thenounproject.com/icon/decision-4829014/ on 02/04/2023. Royalty-Free License

## Slide 30
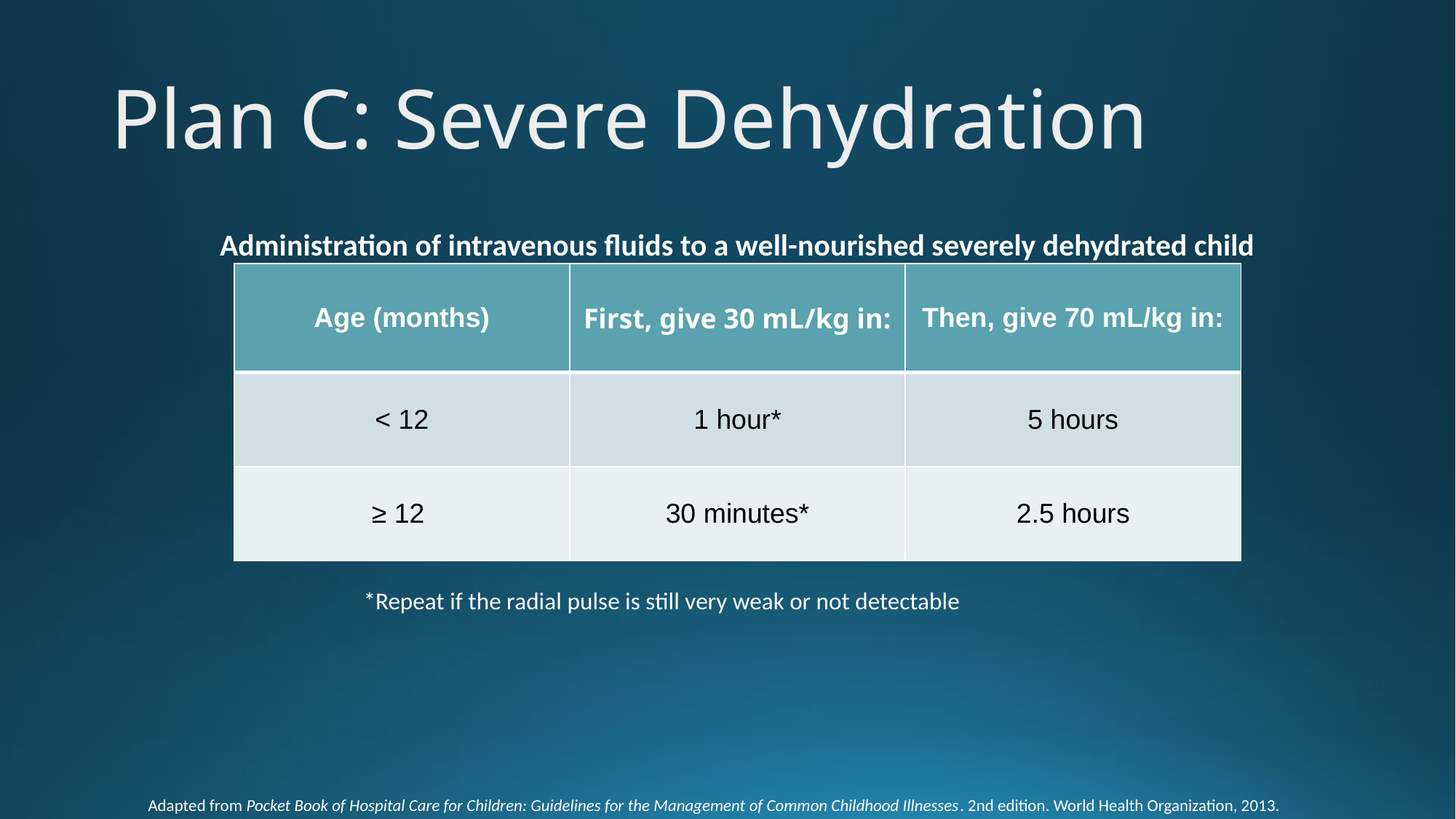

# Plan C: Severe Dehydration
Administration of intravenous fluids to a well-nourished severely dehydrated child
| Age (months) | First, give 30 mL/kg in: | Then, give 70 mL/kg in: |
| --- | --- | --- |
| < 12 | 1 hour\* | 5 hours |
| ≥ 12 | 30 minutes\* | 2.5 hours |
*Repeat if the radial pulse is still very weak or not detectable
Adapted from Pocket Book of Hospital Care for Children: Guidelines for the Management of Common Childhood Illnesses. 2nd edition. World Health Organization, 2013.

## Slide 31
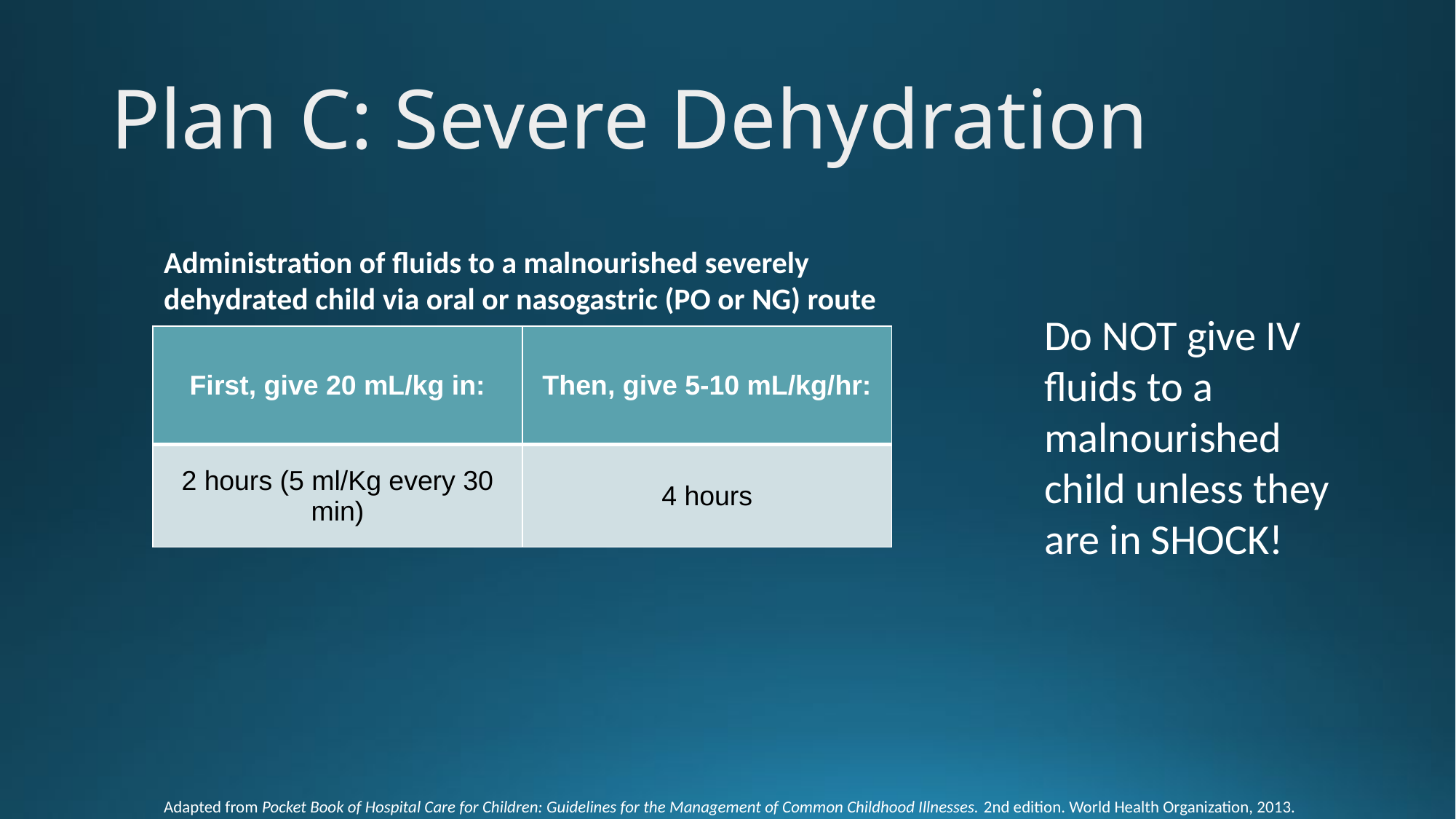

# Plan C: Severe Dehydration
Administration of fluids to a malnourished severely dehydrated child via oral or nasogastric (PO or NG) route
Do NOT give IV fluids to a malnourished child unless they are in SHOCK!
| First, give 20 mL/kg in: | Then, give 5-10 mL/kg/hr: |
| --- | --- |
| 2 hours (5 ml/Kg every 30 min) | 4 hours |
Adapted from Pocket Book of Hospital Care for Children: Guidelines for the Management of Common Childhood Illnesses. 2nd edition. World Health Organization, 2013.

## Slide 32
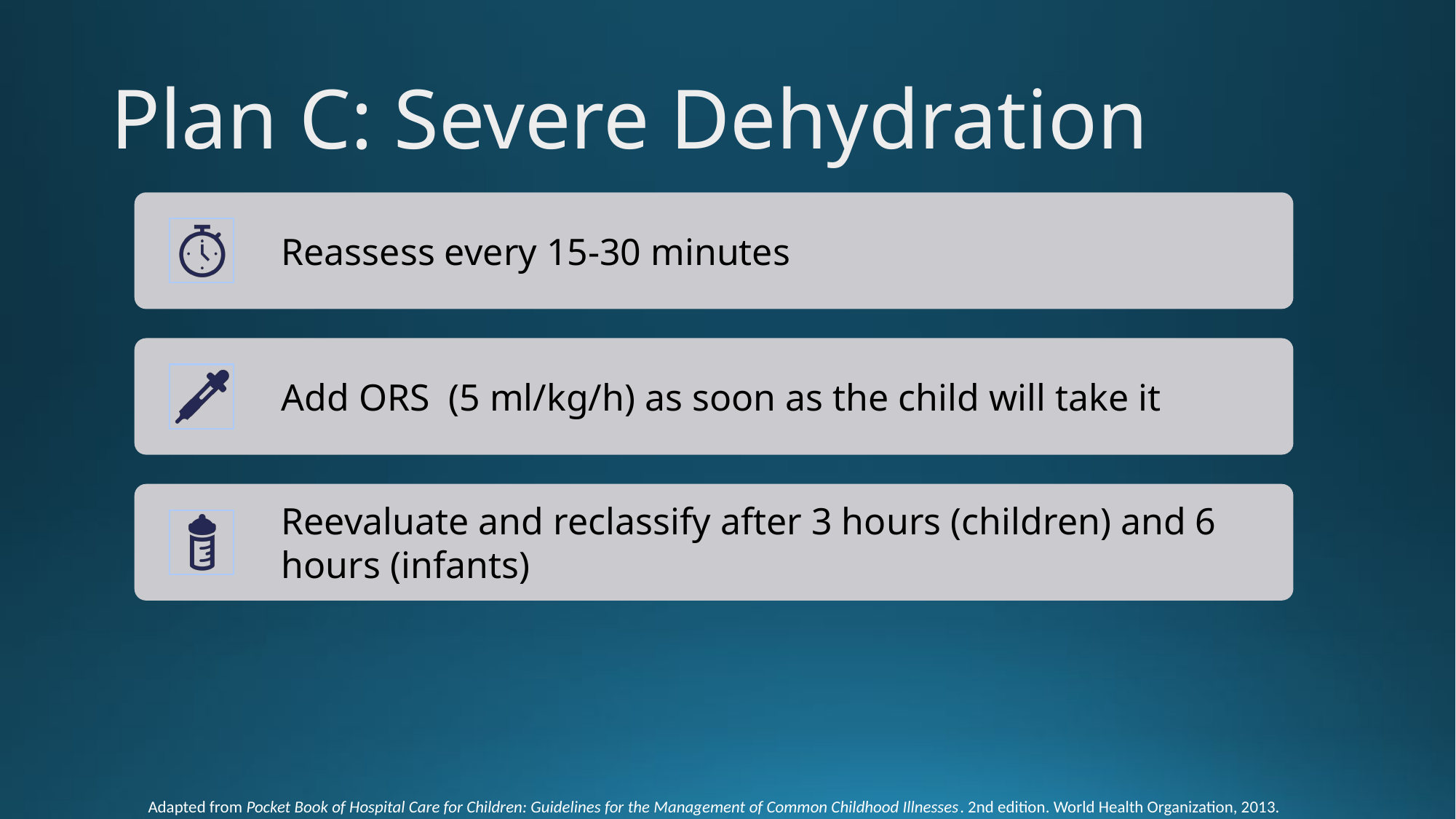

# Plan C: Severe Dehydration
Reassess every 15-30 minutes
Add ORS (5 ml/kg/h) as soon as the child will take it
Reevaluate and reclassify after 3 hours (children) and 6 hours (infants)
Adapted from Pocket Book of Hospital Care for Children: Guidelines for the Management of Common Childhood Illnesses. 2nd edition. World Health Organization, 2013.

## Slide 33
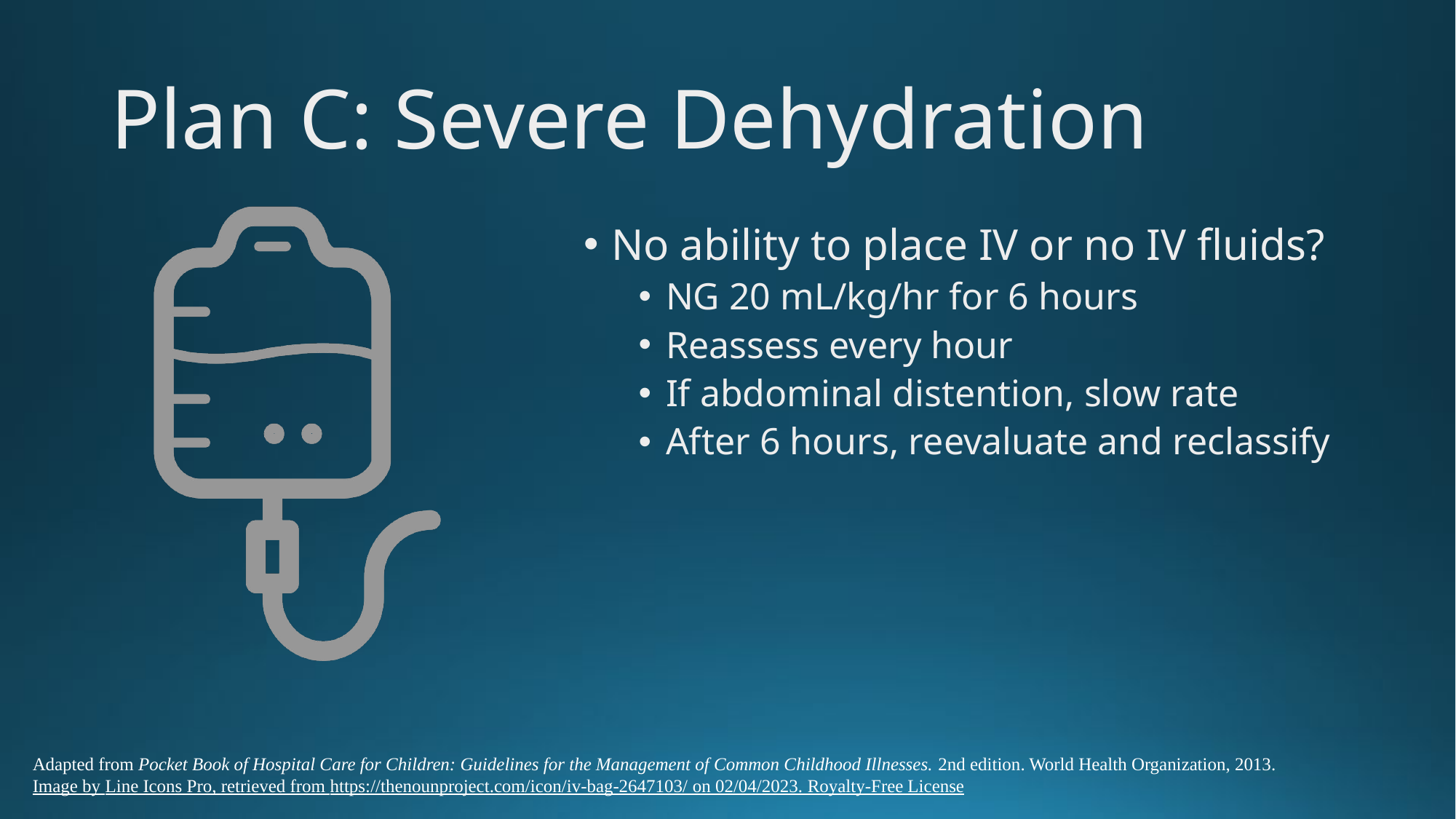

# Plan C: Severe Dehydration
No ability to place IV or no IV fluids?
NG 20 mL/kg/hr for 6 hours
Reassess every hour
If abdominal distention, slow rate
After 6 hours, reevaluate and reclassify
Adapted from Pocket Book of Hospital Care for Children: Guidelines for the Management of Common Childhood Illnesses. 2nd edition. World Health Organization, 2013.
Image by Line Icons Pro, retrieved from https://thenounproject.com/icon/iv-bag-2647103/ on 02/04/2023. Royalty-Free License

## Slide 34
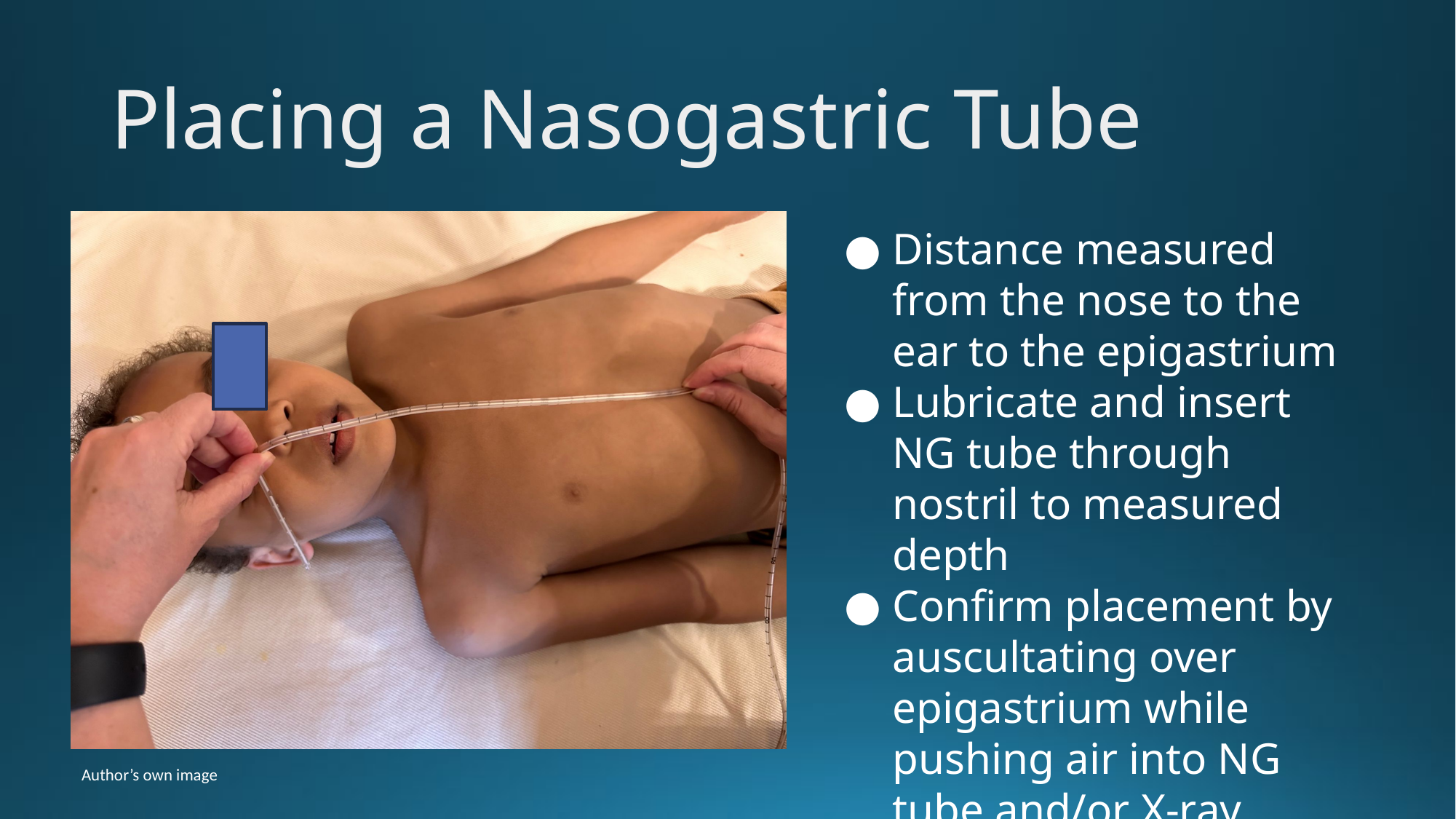

# Placing a Nasogastric Tube
Distance measured from the nose to the ear to the epigastrium
Lubricate and insert NG tube through nostril to measured depth
Confirm placement by auscultating over epigastrium while pushing air into NG tube and/or X-ray
Author’s own image

## Slide 35
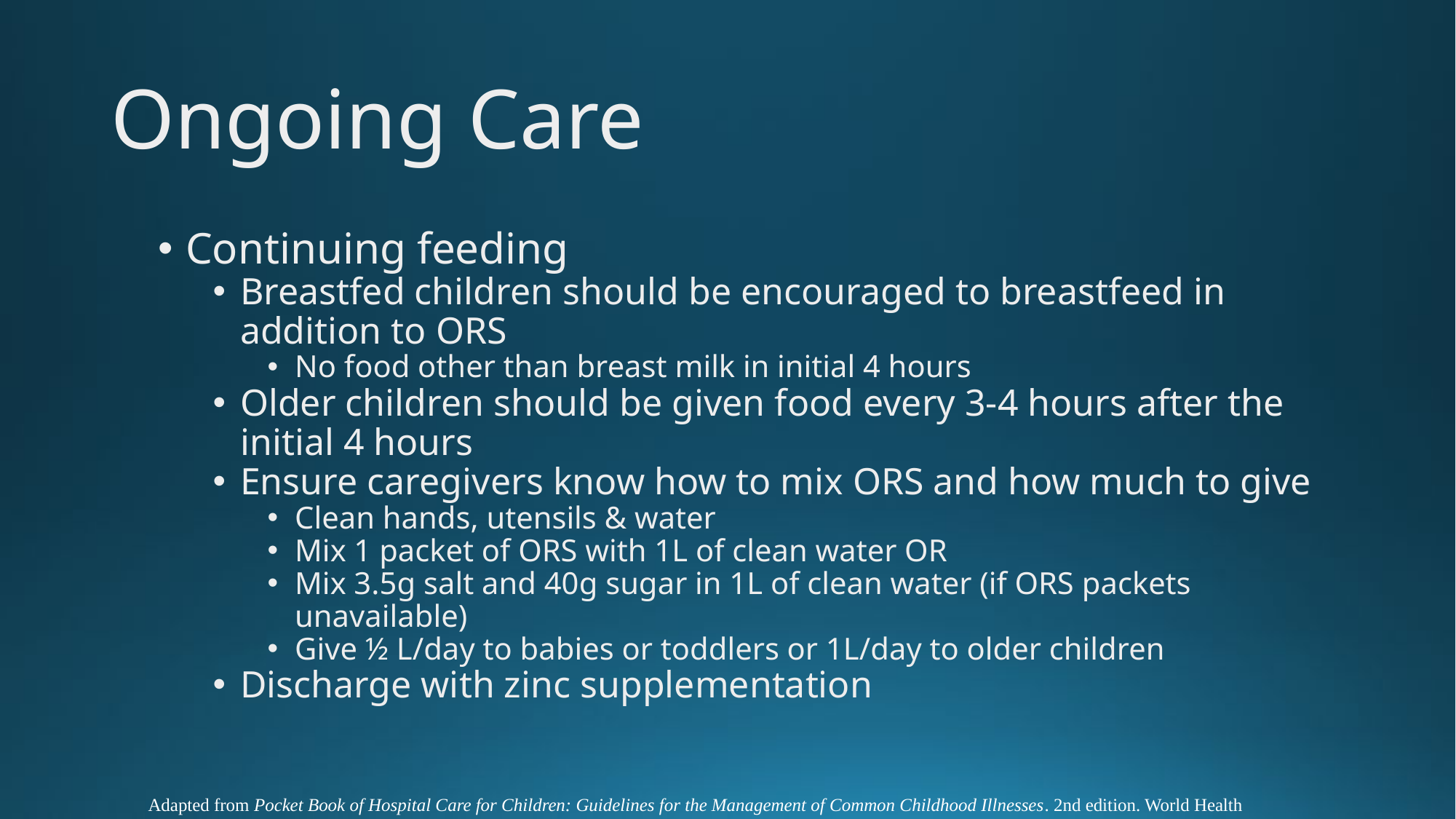

# Ongoing Care
Continuing feeding
Breastfed children should be encouraged to breastfeed in addition to ORS
No food other than breast milk in initial 4 hours
Older children should be given food every 3-4 hours after the initial 4 hours
Ensure caregivers know how to mix ORS and how much to give
Clean hands, utensils & water
Mix 1 packet of ORS with 1L of clean water OR
Mix 3.5g salt and 40g sugar in 1L of clean water (if ORS packets unavailable)
Give ½ L/day to babies or toddlers or 1L/day to older children
Discharge with zinc supplementation
Adapted from Pocket Book of Hospital Care for Children: Guidelines for the Management of Common Childhood Illnesses. 2nd edition. World Health Organization, 2013.

## Slide 36
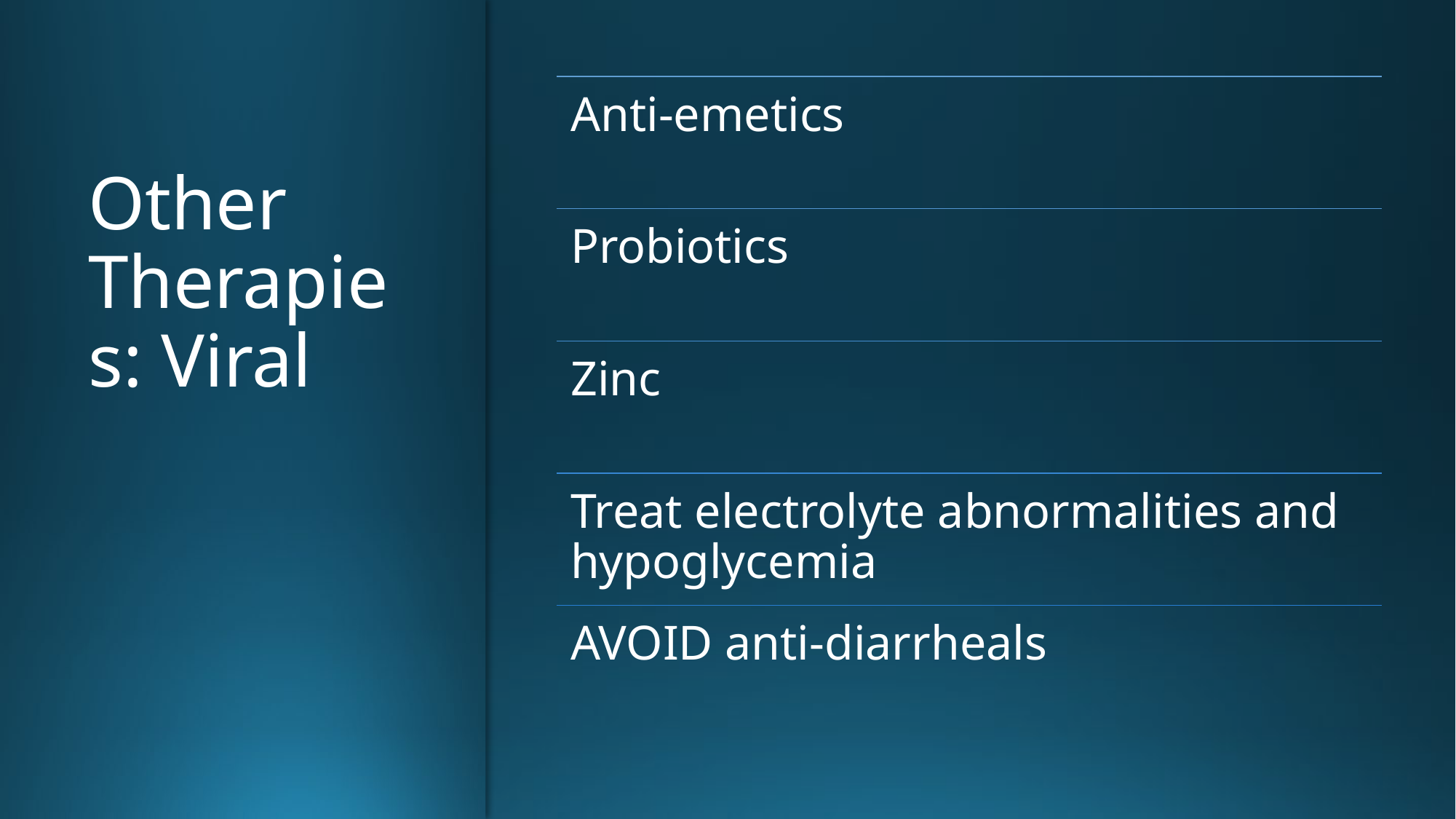

Anti-emetics
Probiotics
Zinc
Treat electrolyte abnormalities and hypoglycemia
AVOID anti-diarrheals
# Other Therapies: Viral

## Slide 37
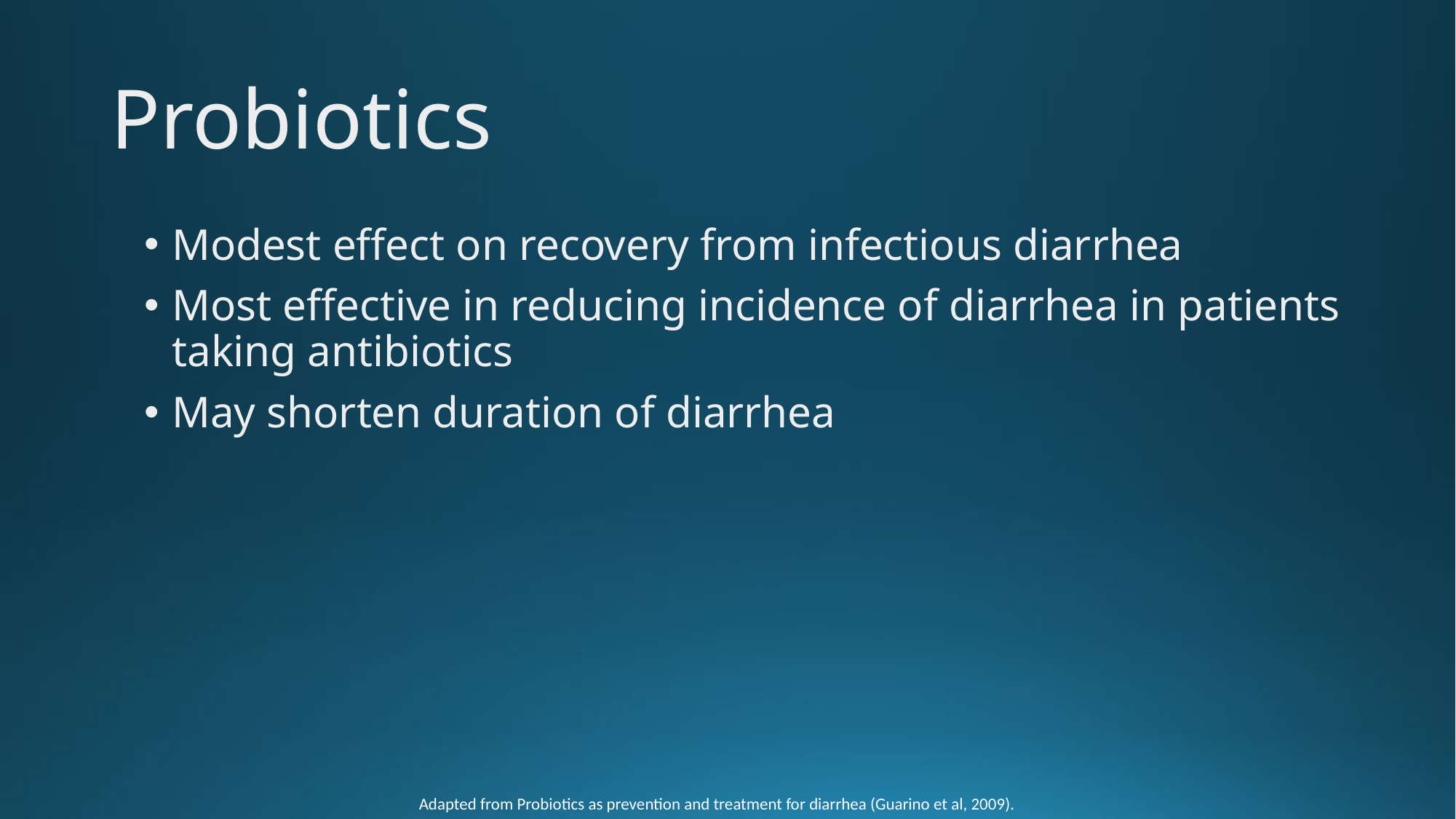

# Probiotics
Modest effect on recovery from infectious diarrhea
Most effective in reducing incidence of diarrhea in patients taking antibiotics
May shorten duration of diarrhea
Adapted from Probiotics as prevention and treatment for diarrhea (Guarino et al, 2009).

## Slide 38
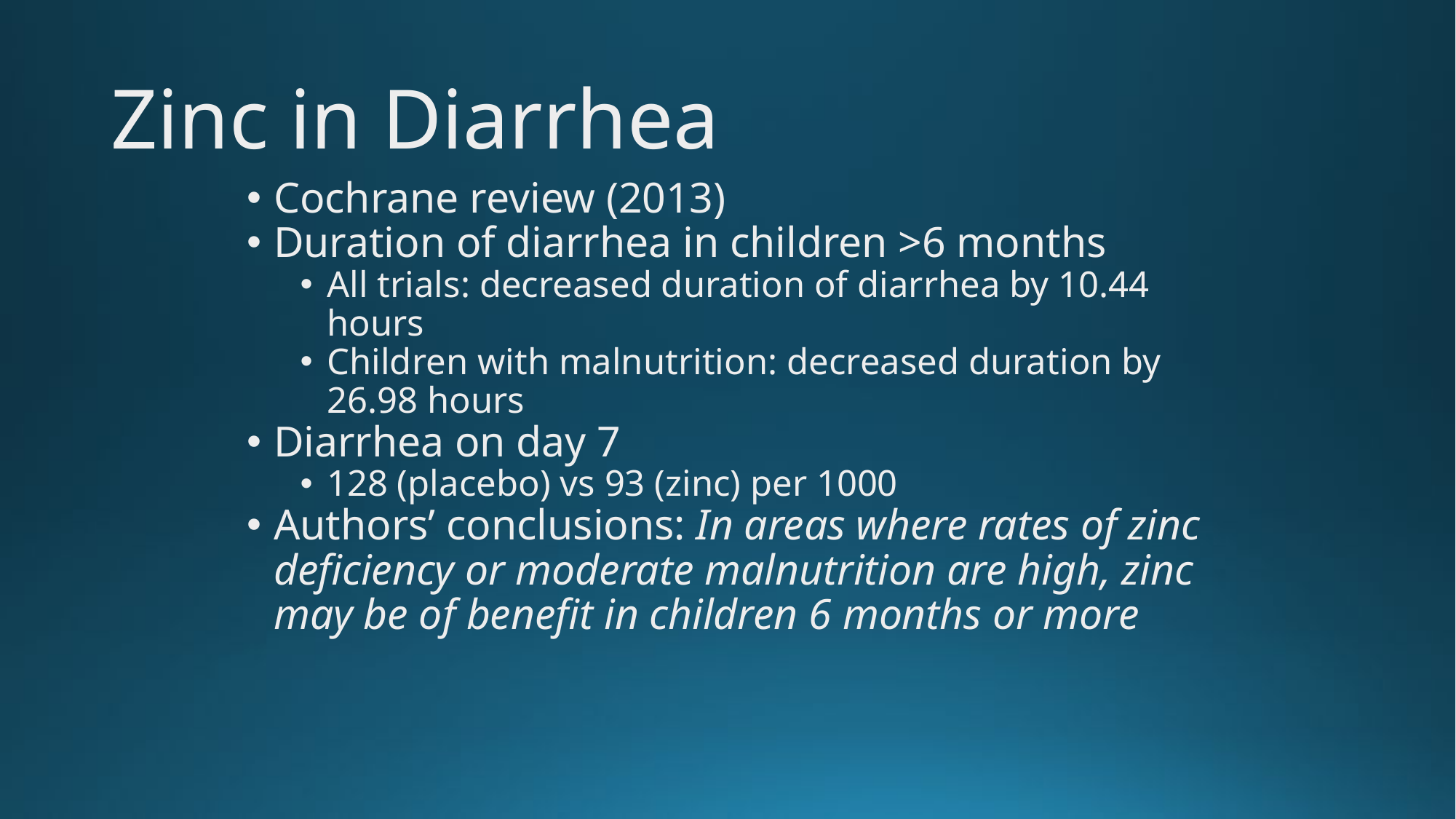

# Zinc in Diarrhea
Cochrane review (2013)
Duration of diarrhea in children >6 months
All trials: decreased duration of diarrhea by 10.44 hours
Children with malnutrition: decreased duration by 26.98 hours
Diarrhea on day 7
128 (placebo) vs 93 (zinc) per 1000
Authors’ conclusions: In areas where rates of zinc deficiency or moderate malnutrition are high, zinc may be of benefit in children 6 months or more

## Slide 39
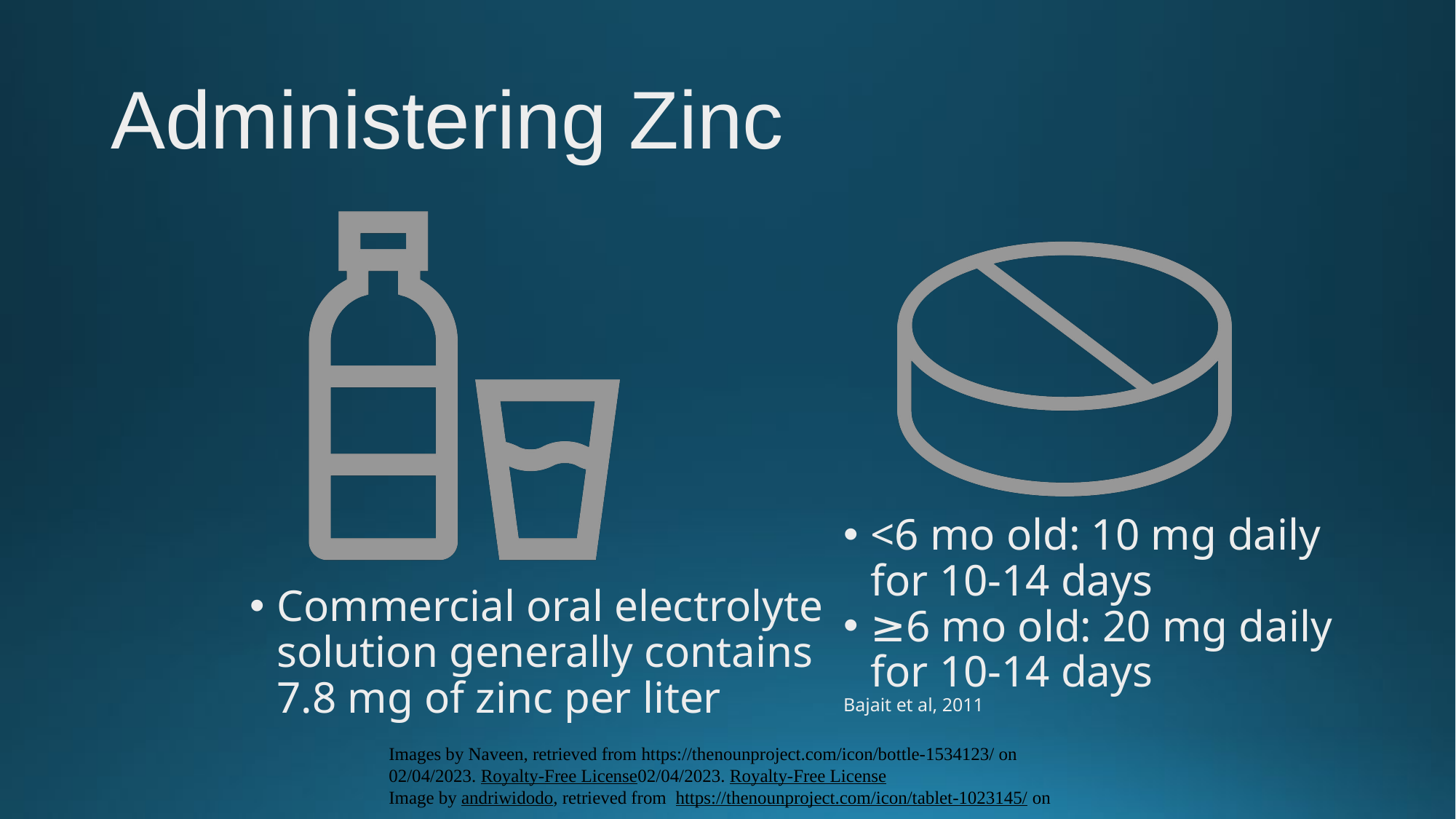

# Administering Zinc
<6 mo old: 10 mg daily for 10-14 days
≥6 mo old: 20 mg daily for 10-14 days
Bajait et al, 2011
Commercial oral electrolyte solution generally contains 7.8 mg of zinc per liter
Images by Naveen, retrieved from https://thenounproject.com/icon/bottle-1534123/ on 02/04/2023. Royalty-Free License02/04/2023. Royalty-Free License
Image by andriwidodo, retrieved from https://thenounproject.com/icon/tablet-1023145/ on

## Slide 40
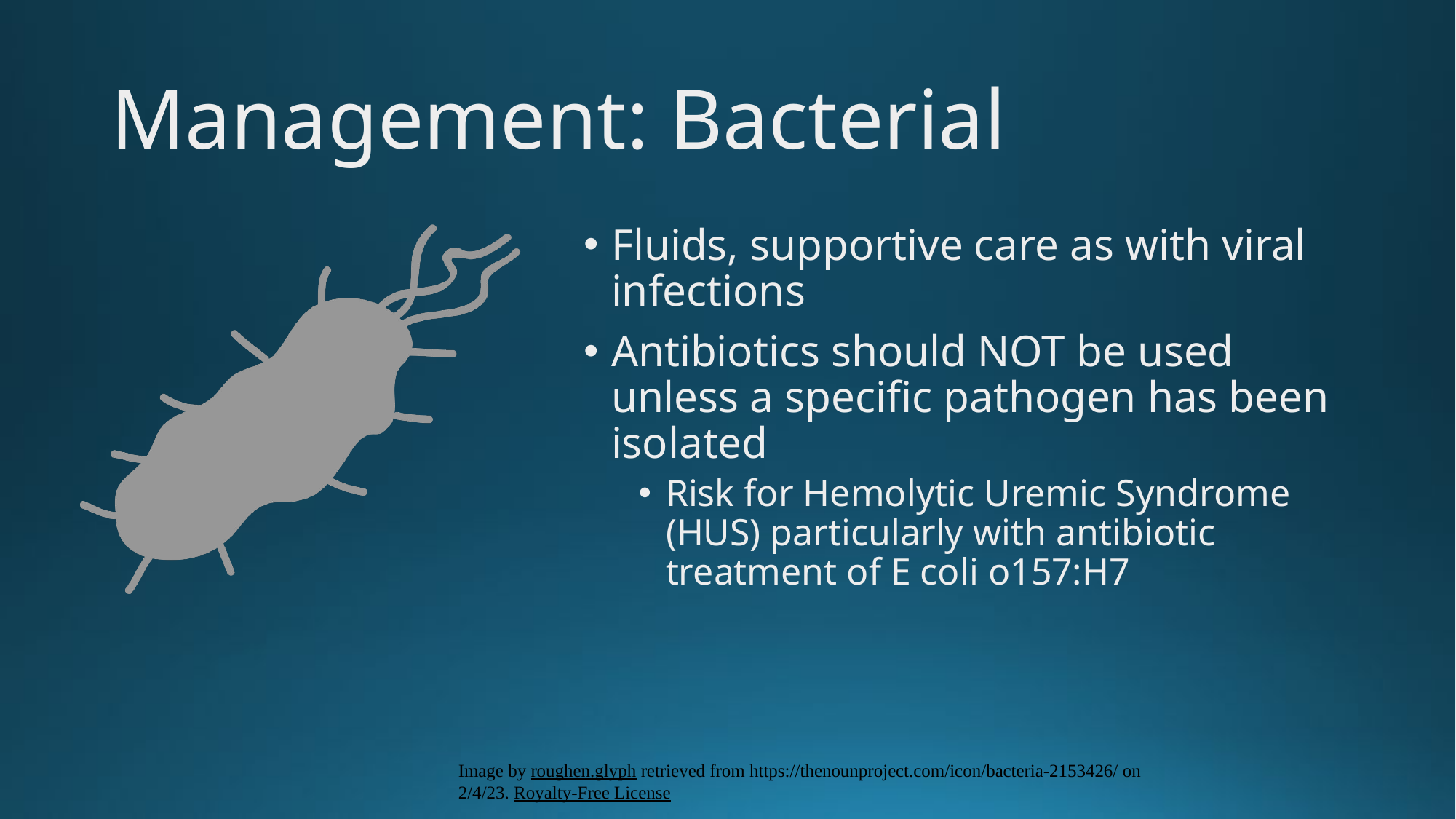

# Management: Bacterial
Fluids, supportive care as with viral infections
Antibiotics should NOT be used unless a specific pathogen has been isolated
Risk for Hemolytic Uremic Syndrome (HUS) particularly with antibiotic treatment of E coli o157:H7
Image by roughen.glyph retrieved from https://thenounproject.com/icon/bacteria-2153426/ on 2/4/23. Royalty-Free License

## Slide 41
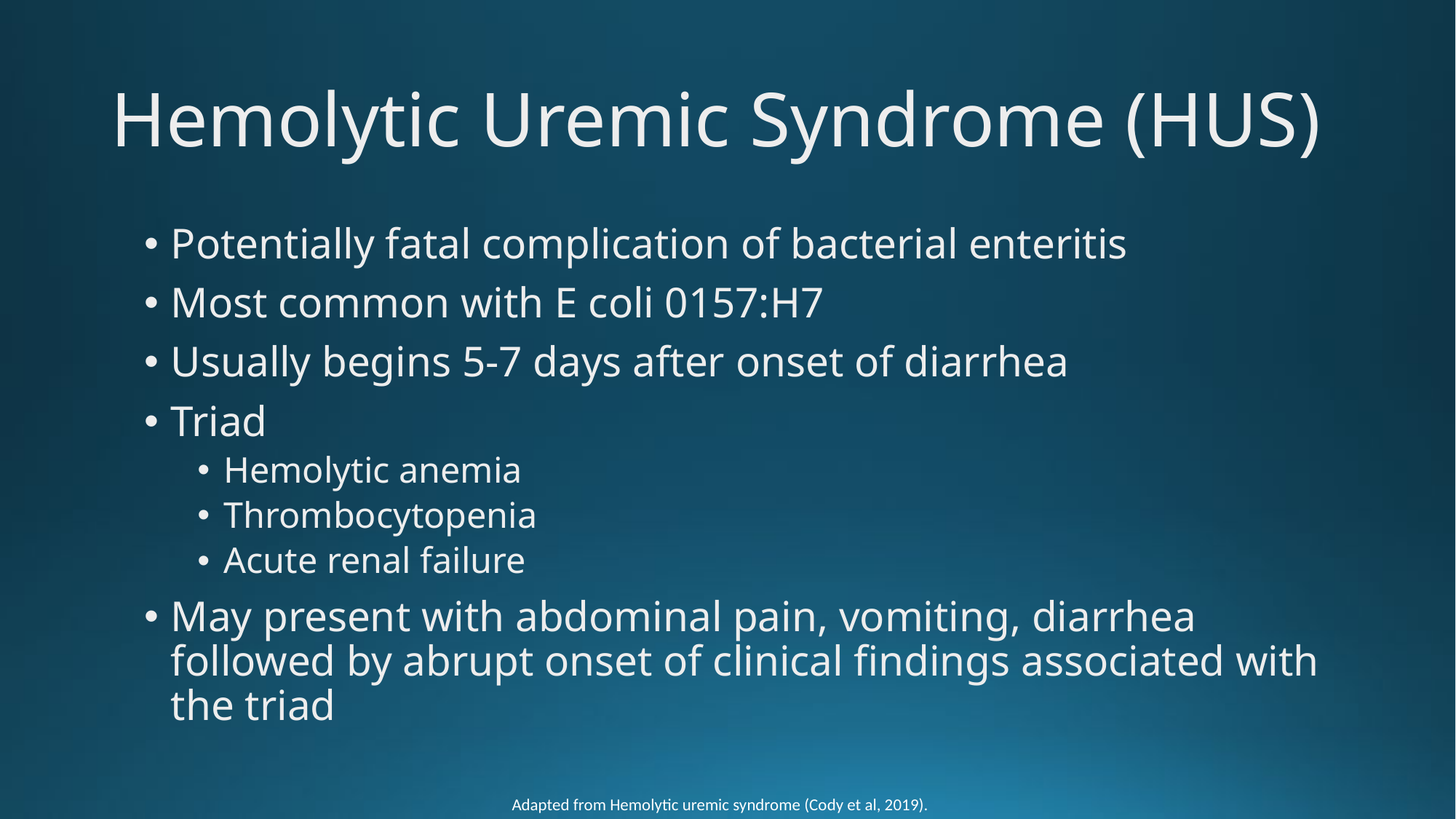

# Hemolytic Uremic Syndrome (HUS)
Potentially fatal complication of bacterial enteritis
Most common with E coli 0157:H7
Usually begins 5-7 days after onset of diarrhea
Triad
Hemolytic anemia
Thrombocytopenia
Acute renal failure
May present with abdominal pain, vomiting, diarrhea followed by abrupt onset of clinical findings associated with the triad
Adapted from Hemolytic uremic syndrome (Cody et al, 2019).

## Slide 42
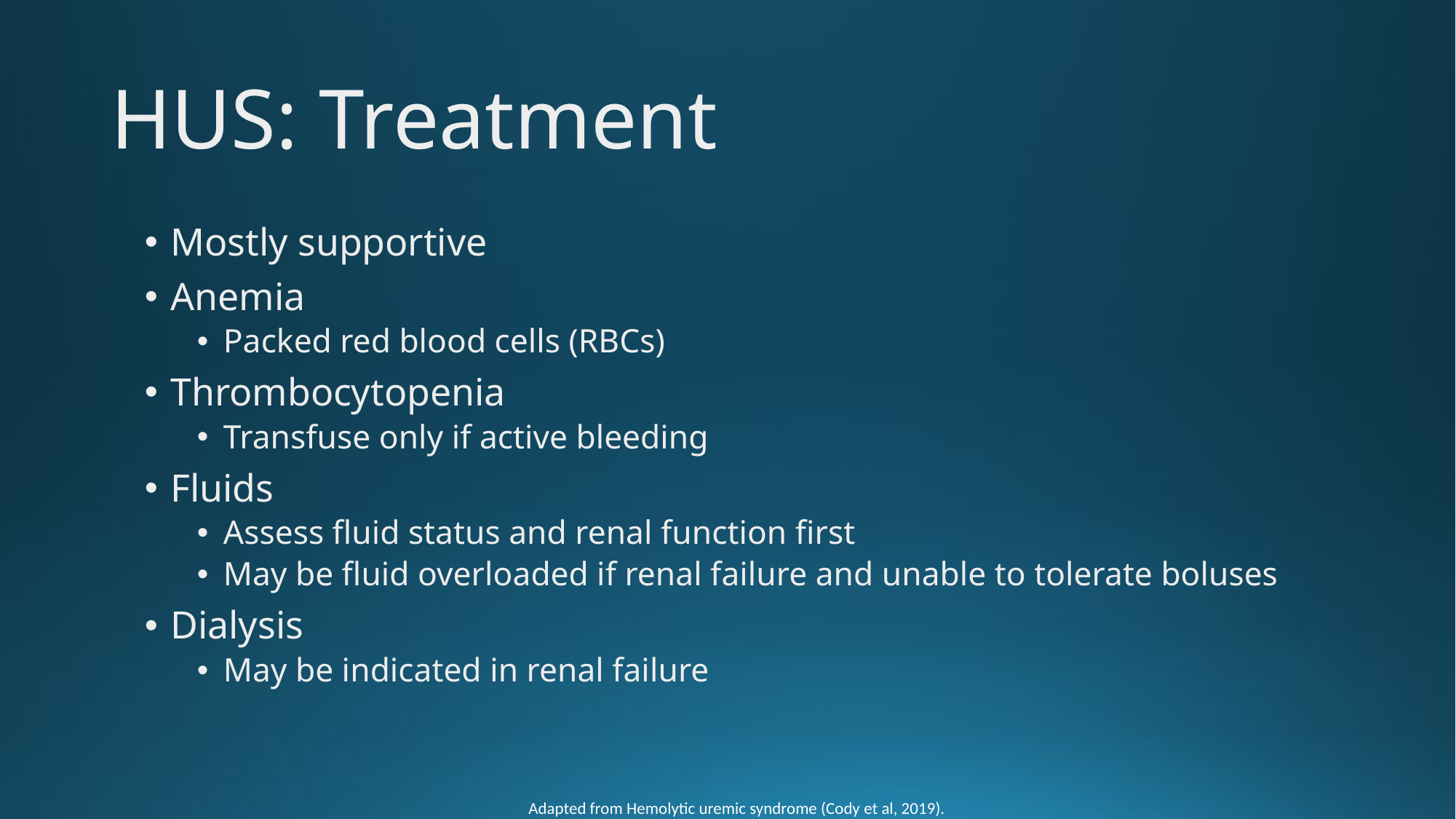

# HUS: Treatment
Mostly supportive
Anemia
Packed red blood cells (RBCs)
Thrombocytopenia
Transfuse only if active bleeding
Fluids
Assess fluid status and renal function first
May be fluid overloaded if renal failure and unable to tolerate boluses
Dialysis
May be indicated in renal failure
Adapted from Hemolytic uremic syndrome (Cody et al, 2019).

## Slide 43
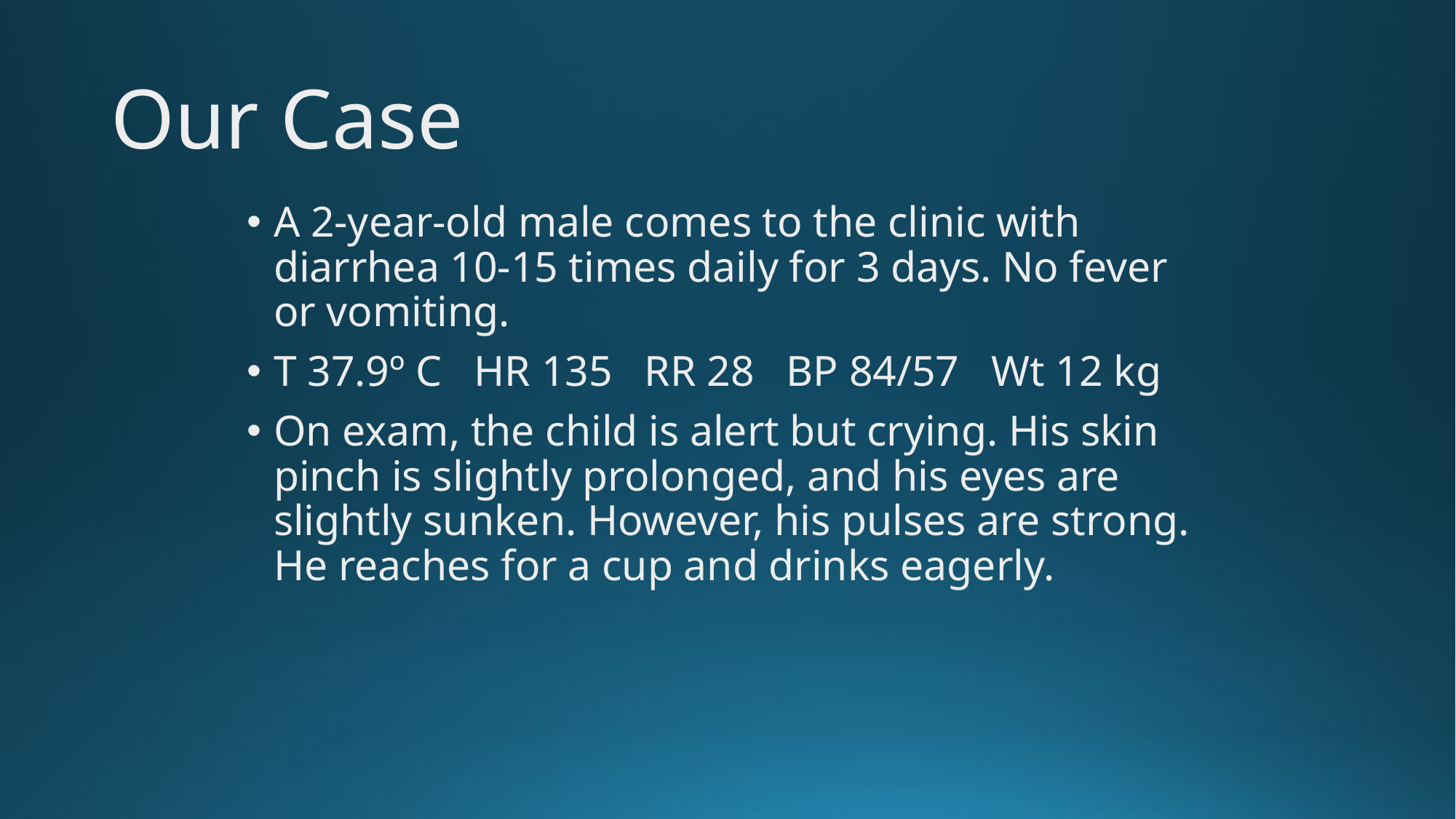

# Our Case
A 2-year-old male comes to the clinic with diarrhea 10-15 times daily for 3 days. No fever or vomiting.
T 37.9º C HR 135 RR 28 BP 84/57 Wt 12 kg
On exam, the child is alert but crying. His skin pinch is slightly prolonged, and his eyes are slightly sunken. However, his pulses are strong. He reaches for a cup and drinks eagerly.

## Slide 44
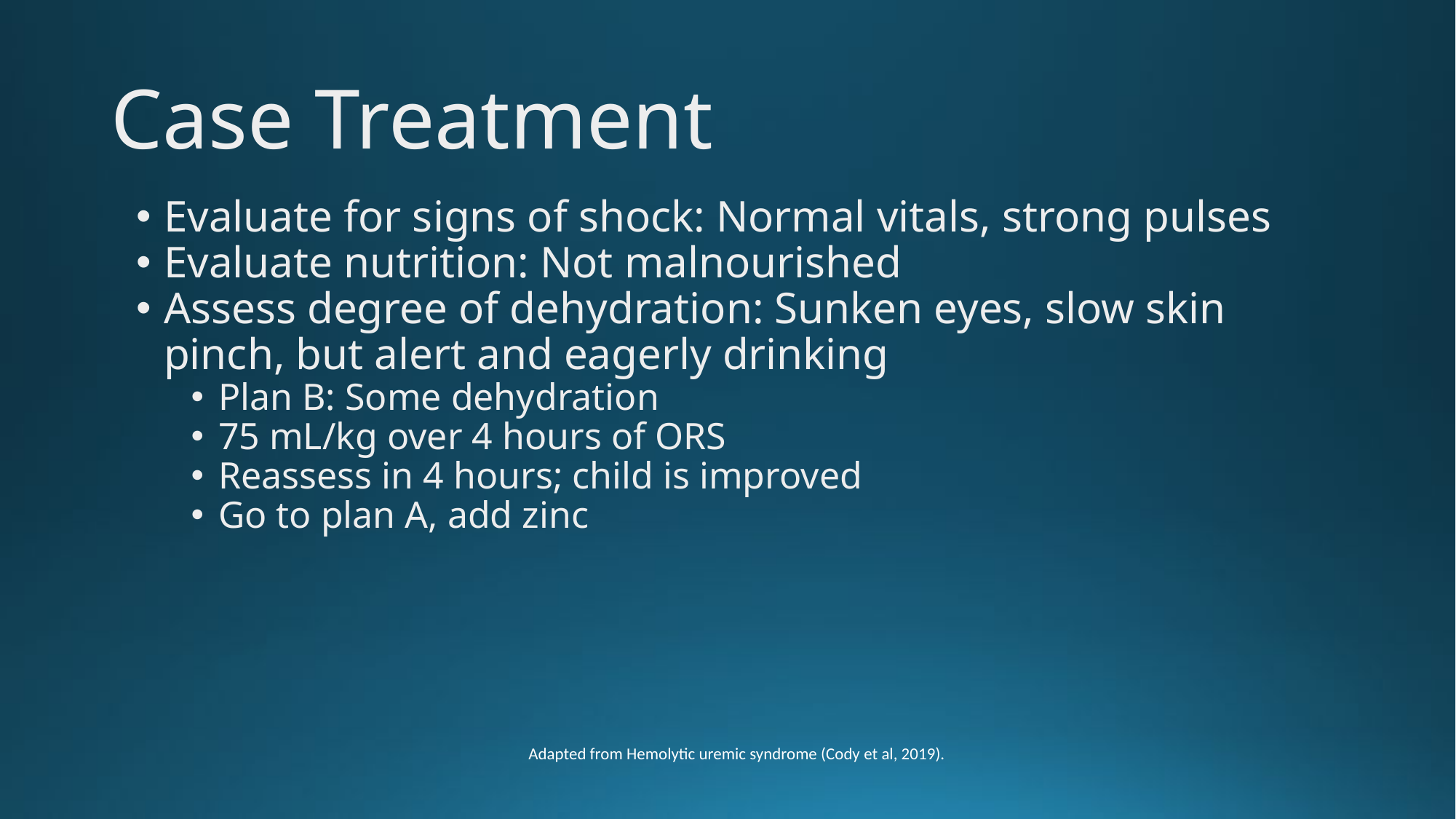

# Case Treatment
Evaluate for signs of shock: Normal vitals, strong pulses
Evaluate nutrition: Not malnourished
Assess degree of dehydration: Sunken eyes, slow skin pinch, but alert and eagerly drinking
Plan B: Some dehydration
75 mL/kg over 4 hours of ORS
Reassess in 4 hours; child is improved
Go to plan A, add zinc
Adapted from Hemolytic uremic syndrome (Cody et al, 2019).

## Slide 45
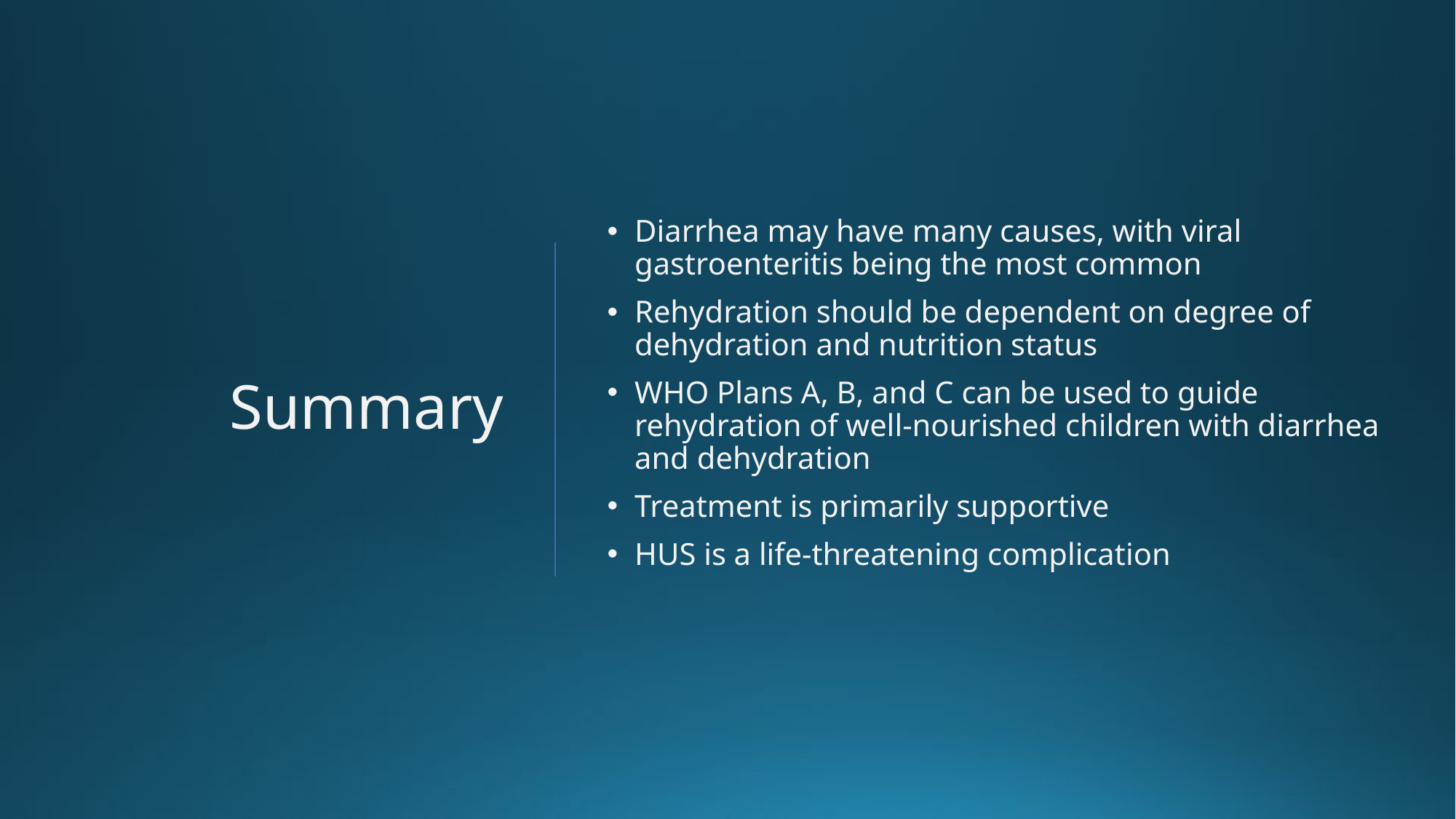

Diarrhea may have many causes, with viral gastroenteritis being the most common
Rehydration should be dependent on degree of dehydration and nutrition status
WHO Plans A, B, and C can be used to guide rehydration of well-nourished children with diarrhea and dehydration
Treatment is primarily supportive
HUS is a life-threatening complication
# Summary

## Slide 46
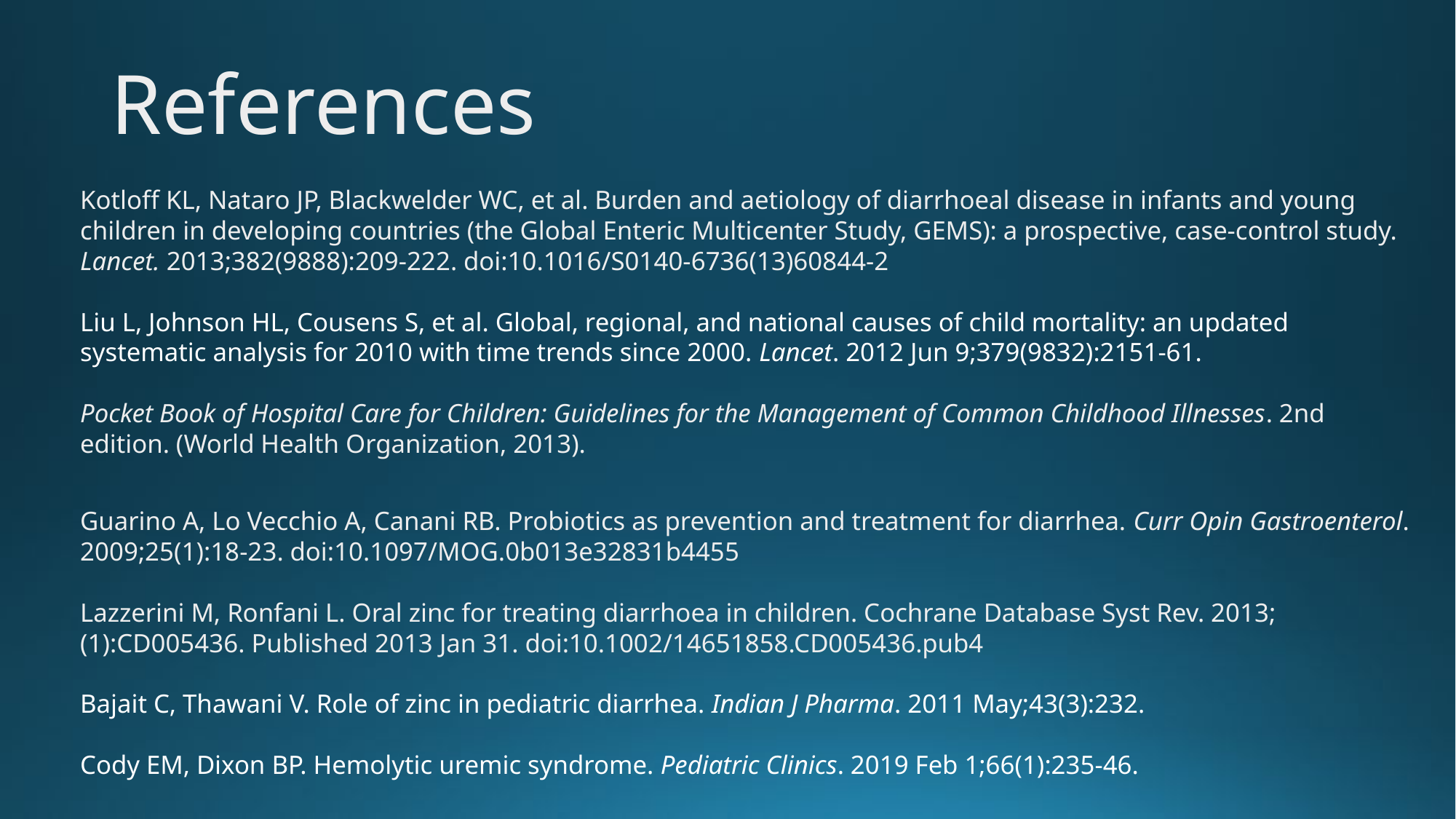

# References
Kotloff KL, Nataro JP, Blackwelder WC, et al. Burden and aetiology of diarrhoeal disease in infants and young children in developing countries (the Global Enteric Multicenter Study, GEMS): a prospective, case-control study. Lancet. 2013;382(9888):209-222. doi:10.1016/S0140-6736(13)60844-2
Liu L, Johnson HL, Cousens S, et al. Global, regional, and national causes of child mortality: an updated systematic analysis for 2010 with time trends since 2000. Lancet. 2012 Jun 9;379(9832):2151-61.
Pocket Book of Hospital Care for Children: Guidelines for the Management of Common Childhood Illnesses. 2nd edition. (World Health Organization, 2013).
Guarino A, Lo Vecchio A, Canani RB. Probiotics as prevention and treatment for diarrhea. Curr Opin Gastroenterol. 2009;25(1):18-23. doi:10.1097/MOG.0b013e32831b4455
Lazzerini M, Ronfani L. Oral zinc for treating diarrhoea in children. Cochrane Database Syst Rev. 2013;(1):CD005436. Published 2013 Jan 31. doi:10.1002/14651858.CD005436.pub4
Bajait C, Thawani V. Role of zinc in pediatric diarrhea. Indian J Pharma. 2011 May;43(3):232.
Cody EM, Dixon BP. Hemolytic uremic syndrome. Pediatric Clinics. 2019 Feb 1;66(1):235-46.
