# Supplementary material for: A Simulation and Small-Group Pediatric Emergency Medicine Course for Generalist Healthcare Providers: Gastrointestinal and Nutrition Emergencies
Source: J Educ Teach Emerg Med. 2024 Oct 31;9(4):C1–C120. doi: 10.21980/J8WH2K (PMC11537732; doi:10.21980/J8WH2K)
Supplement: Supplementary file 2 — Please see associated PowerPoint file [file 9-4-C1-Appendix_I.pptx]

## Slide 1
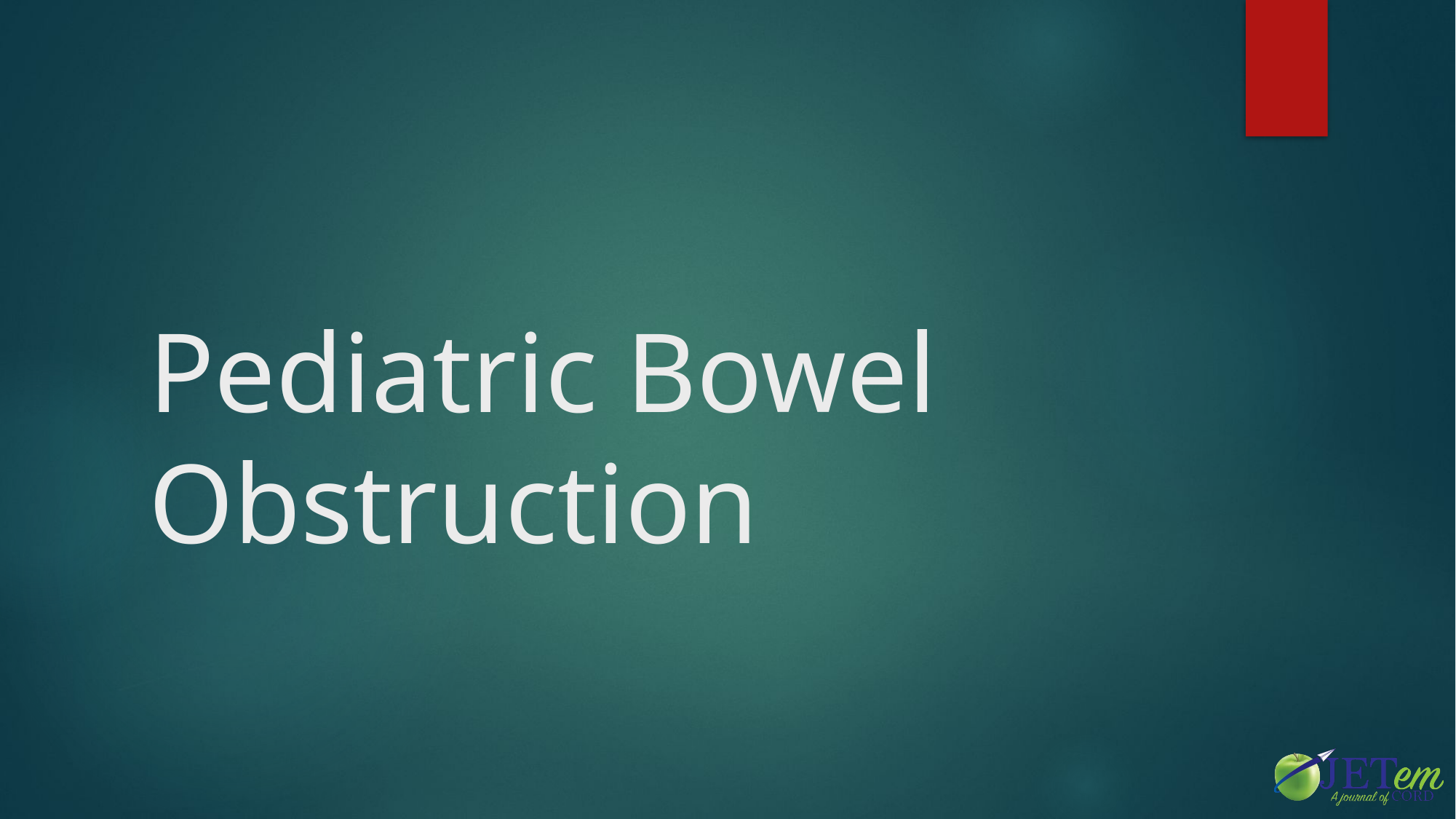

# Pediatric Bowel Obstruction

## Slide 2
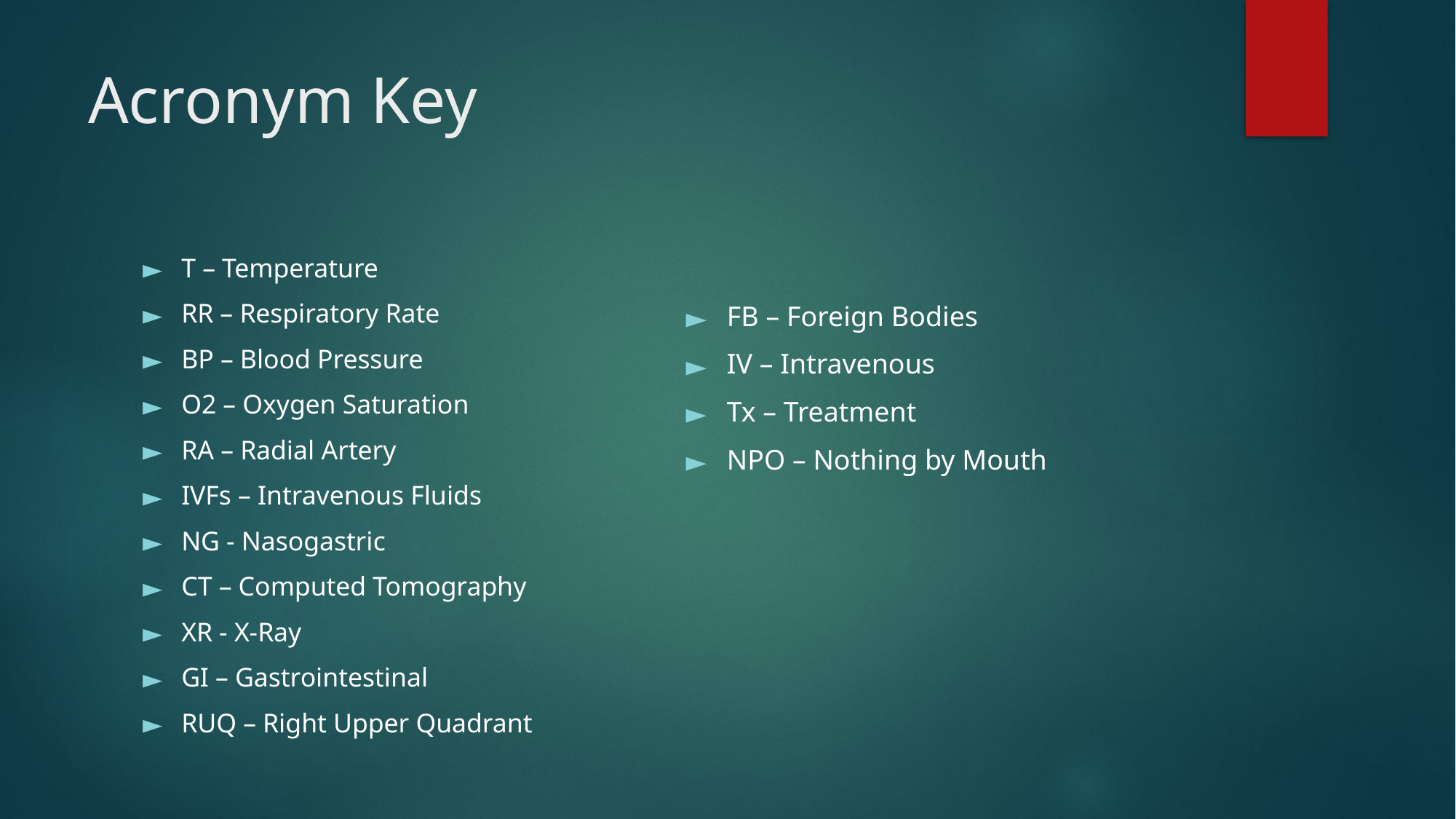

# Acronym Key
FB – Foreign Bodies
IV – Intravenous
Tx – Treatment
NPO – Nothing by Mouth
T – Temperature
RR – Respiratory Rate
BP – Blood Pressure
O2 – Oxygen Saturation
RA – Radial Artery
IVFs – Intravenous Fluids
NG - Nasogastric
CT – Computed Tomography
XR - X-Ray
GI – Gastrointestinal
RUQ – Right Upper Quadrant

## Slide 3
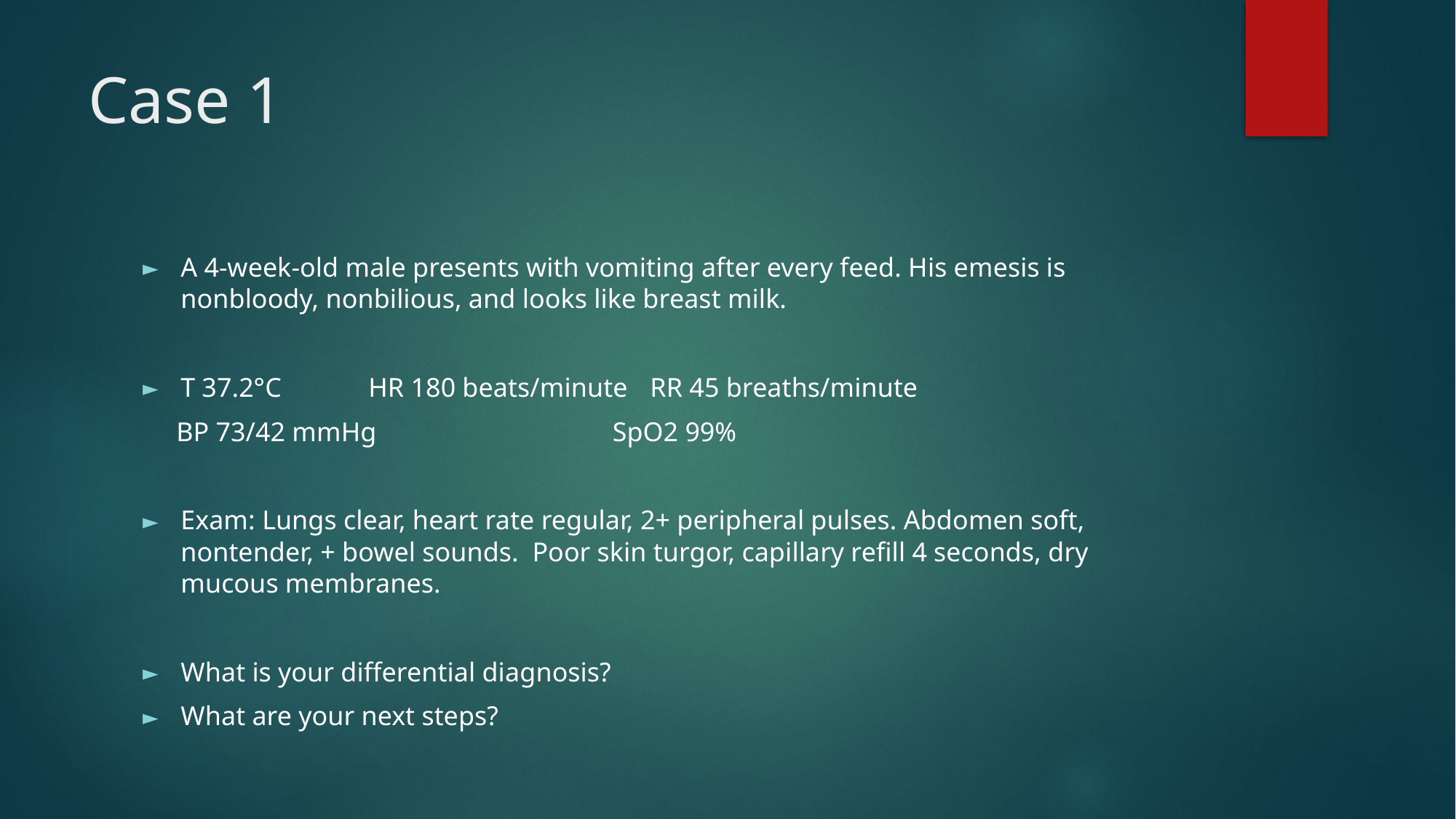

# Case 1
A 4-week-old male presents with vomiting after every feed. His emesis is nonbloody, nonbilious, and looks like breast milk.
T 37.2°C	HR 180 beats/minute 		RR 45 breaths/minute
 BP 73/42 mmHg 		 SpO2 99%
Exam: Lungs clear, heart rate regular, 2+ peripheral pulses. Abdomen soft, nontender, + bowel sounds. Poor skin turgor, capillary refill 4 seconds, dry mucous membranes.
What is your differential diagnosis?
What are your next steps?

## Slide 4
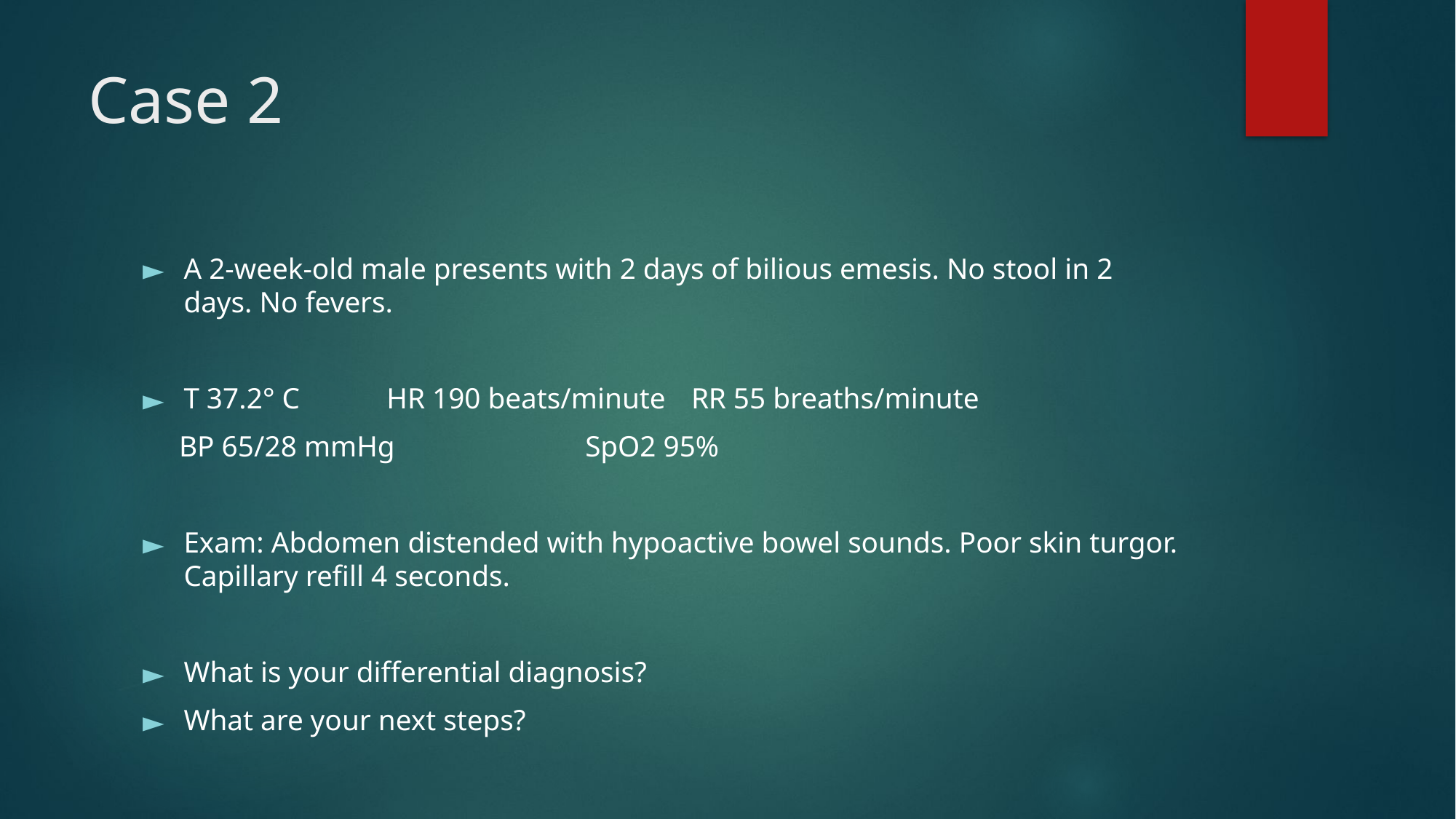

# Case 2
A 2-week-old male presents with 2 days of bilious emesis. No stool in 2 days. No fevers.
T 37.2° C	HR 190 beats/minute 	RR 55 breaths/minute
 BP 65/28 mmHg 		 SpO2 95%
Exam: Abdomen distended with hypoactive bowel sounds. Poor skin turgor. Capillary refill 4 seconds.
What is your differential diagnosis?
What are your next steps?

## Slide 5
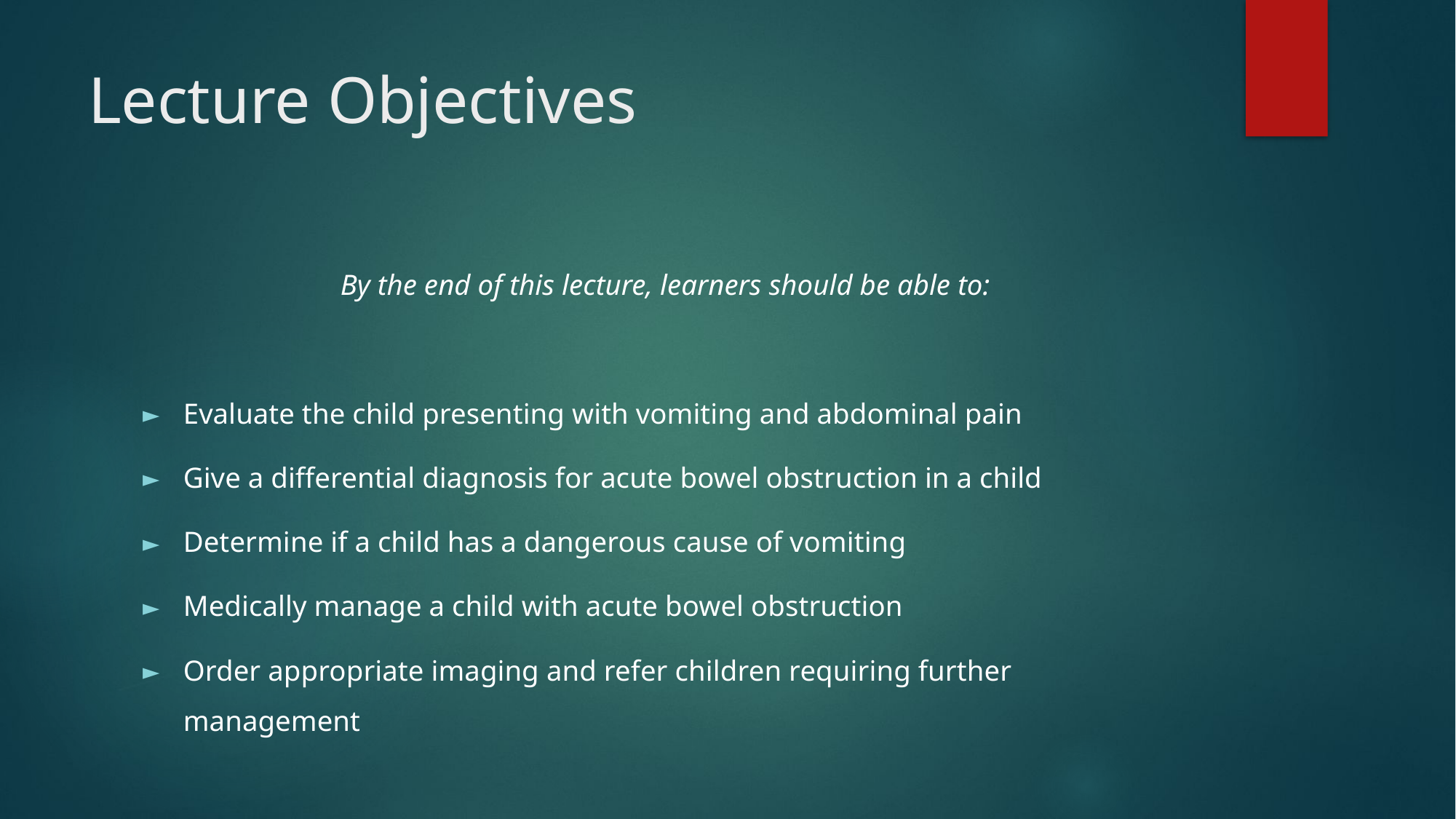

# Lecture Objectives
By the end of this lecture, learners should be able to:
Evaluate the child presenting with vomiting and abdominal pain
Give a differential diagnosis for acute bowel obstruction in a child
Determine if a child has a dangerous cause of vomiting
Medically manage a child with acute bowel obstruction
Order appropriate imaging and refer children requiring further management

## Slide 6
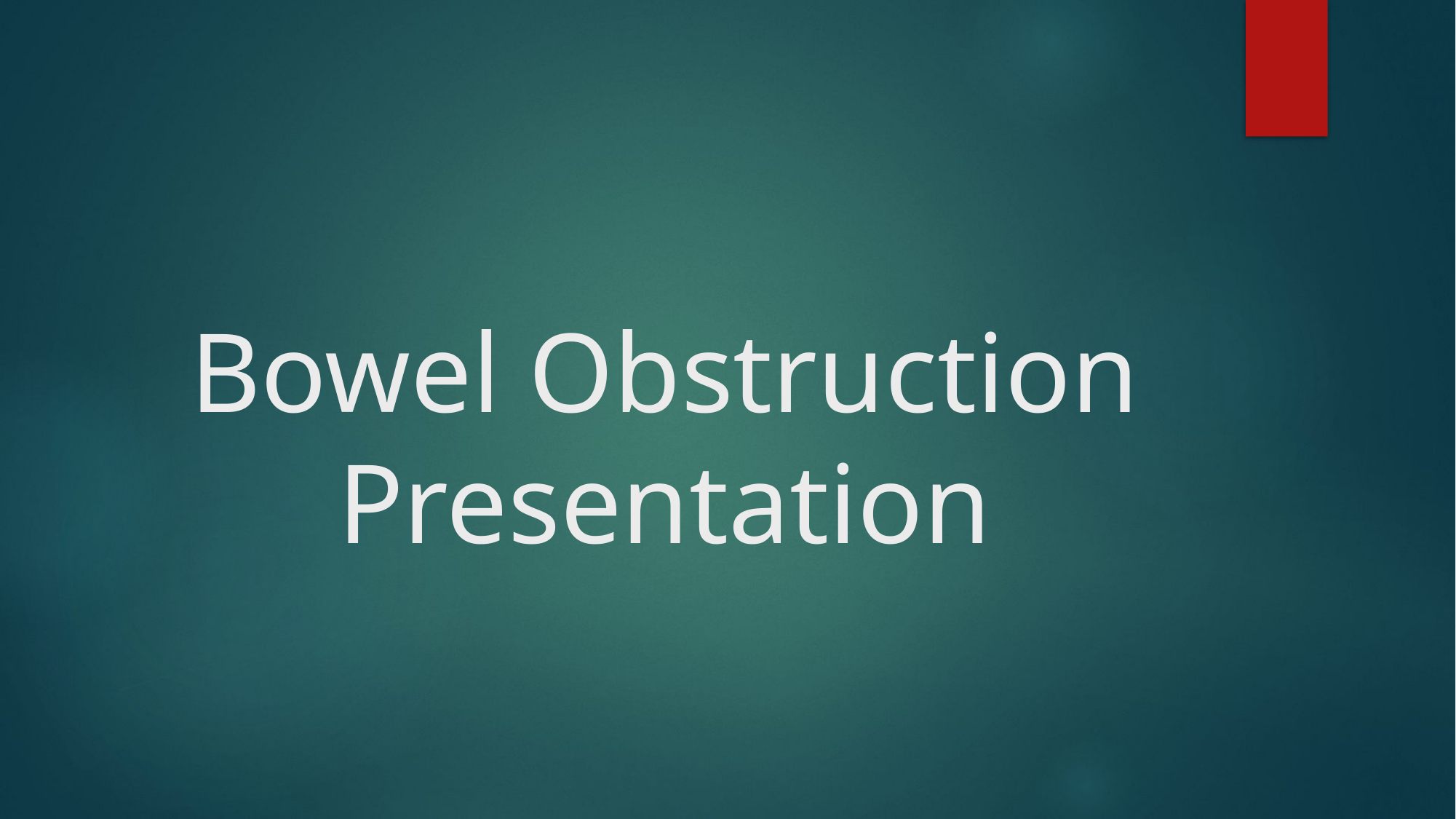

# Bowel Obstruction Presentation

## Slide 7
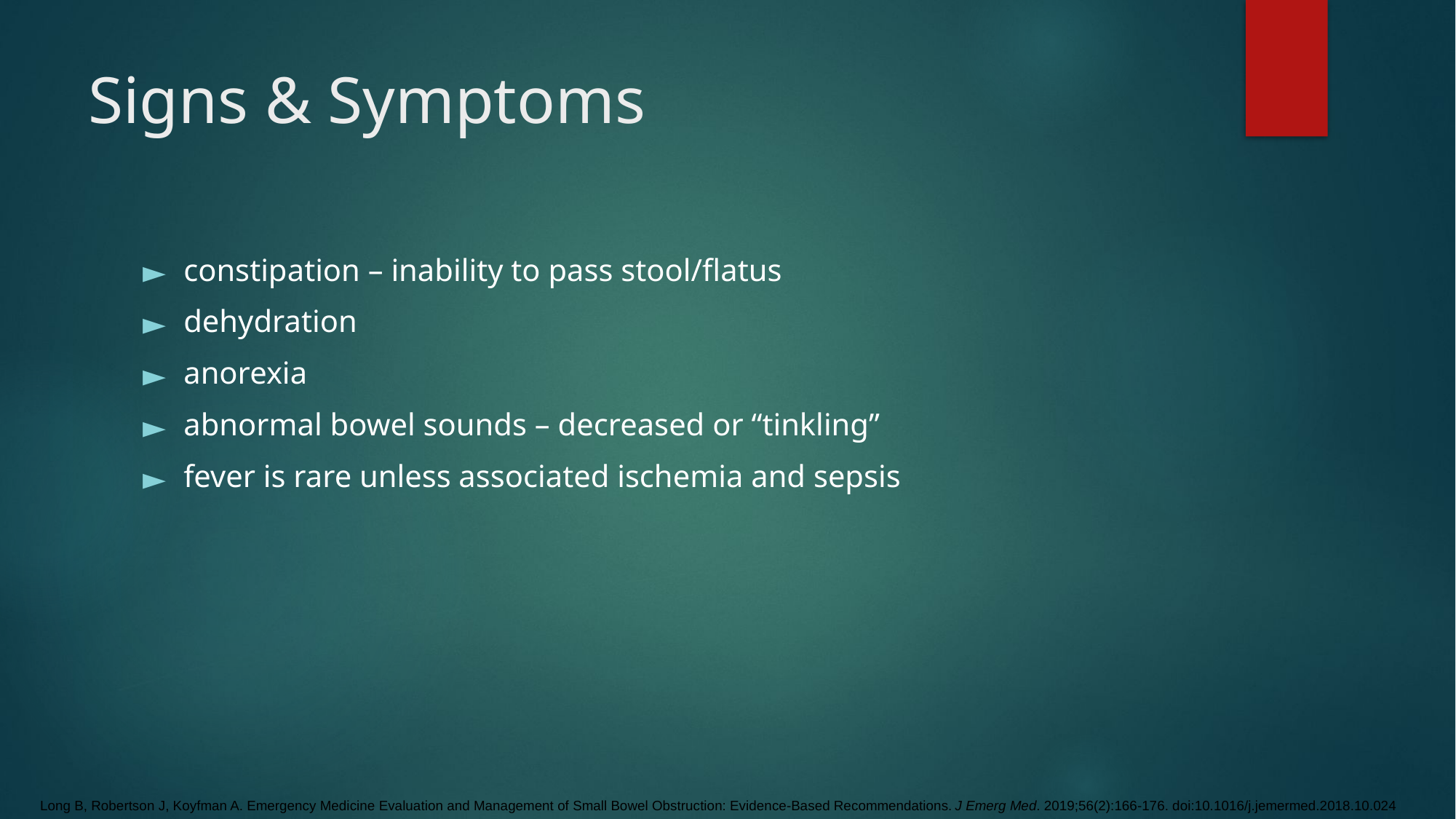

# Signs & Symptoms
constipation – inability to pass stool/flatus
dehydration
anorexia
abnormal bowel sounds – decreased or “tinkling”
fever is rare unless associated ischemia and sepsis
Long B, Robertson J, Koyfman A. Emergency Medicine Evaluation and Management of Small Bowel Obstruction: Evidence-Based Recommendations. J Emerg Med. 2019;56(2):166-176. doi:10.1016/j.jemermed.2018.10.024

## Slide 8
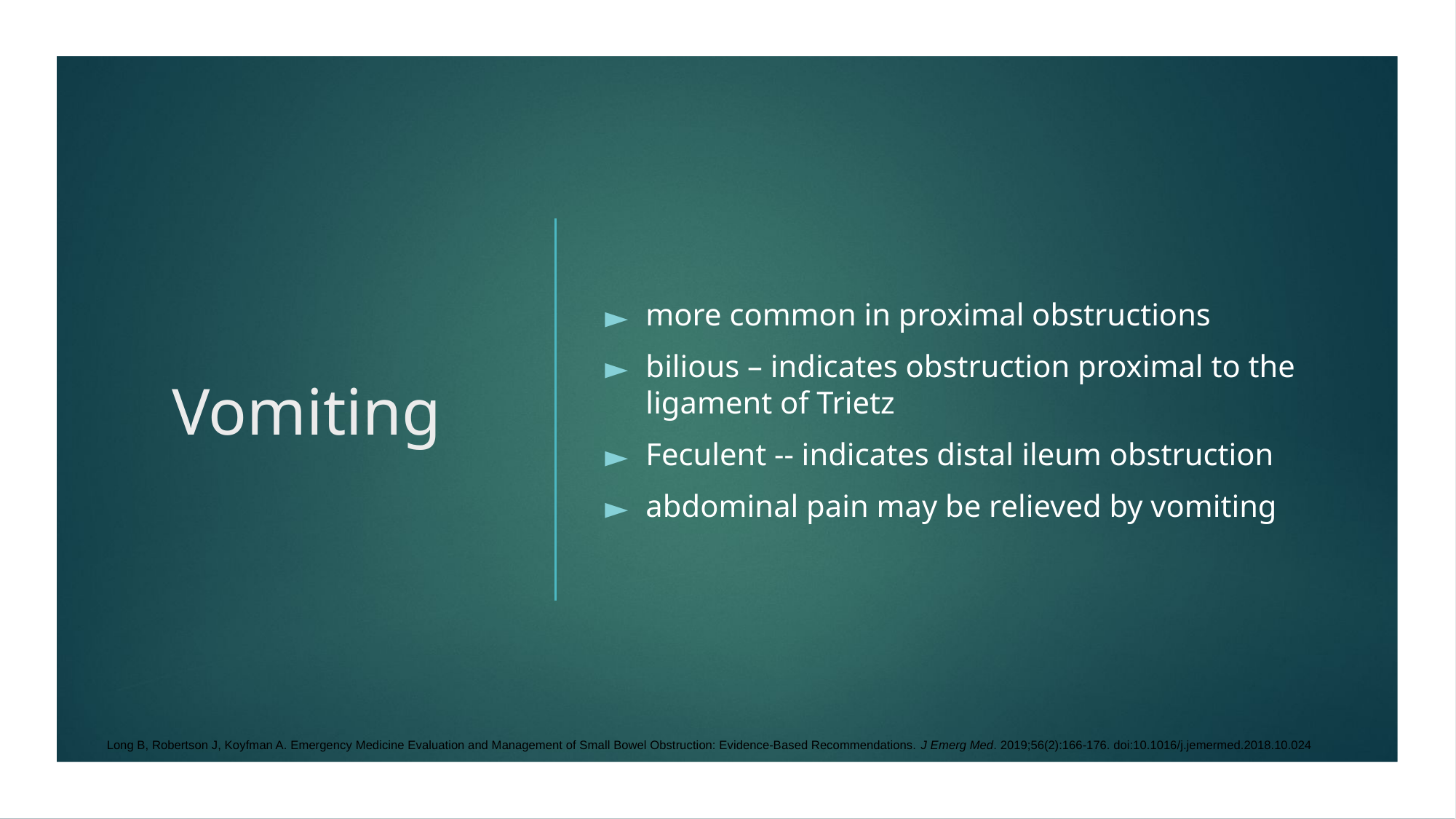

more common in proximal obstructions
bilious – indicates obstruction proximal to the ligament of Trietz
Feculent -- indicates distal ileum obstruction
abdominal pain may be relieved by vomiting
# Vomiting
Long B, Robertson J, Koyfman A. Emergency Medicine Evaluation and Management of Small Bowel Obstruction: Evidence-Based Recommendations. J Emerg Med. 2019;56(2):166-176. doi:10.1016/j.jemermed.2018.10.024

## Slide 9
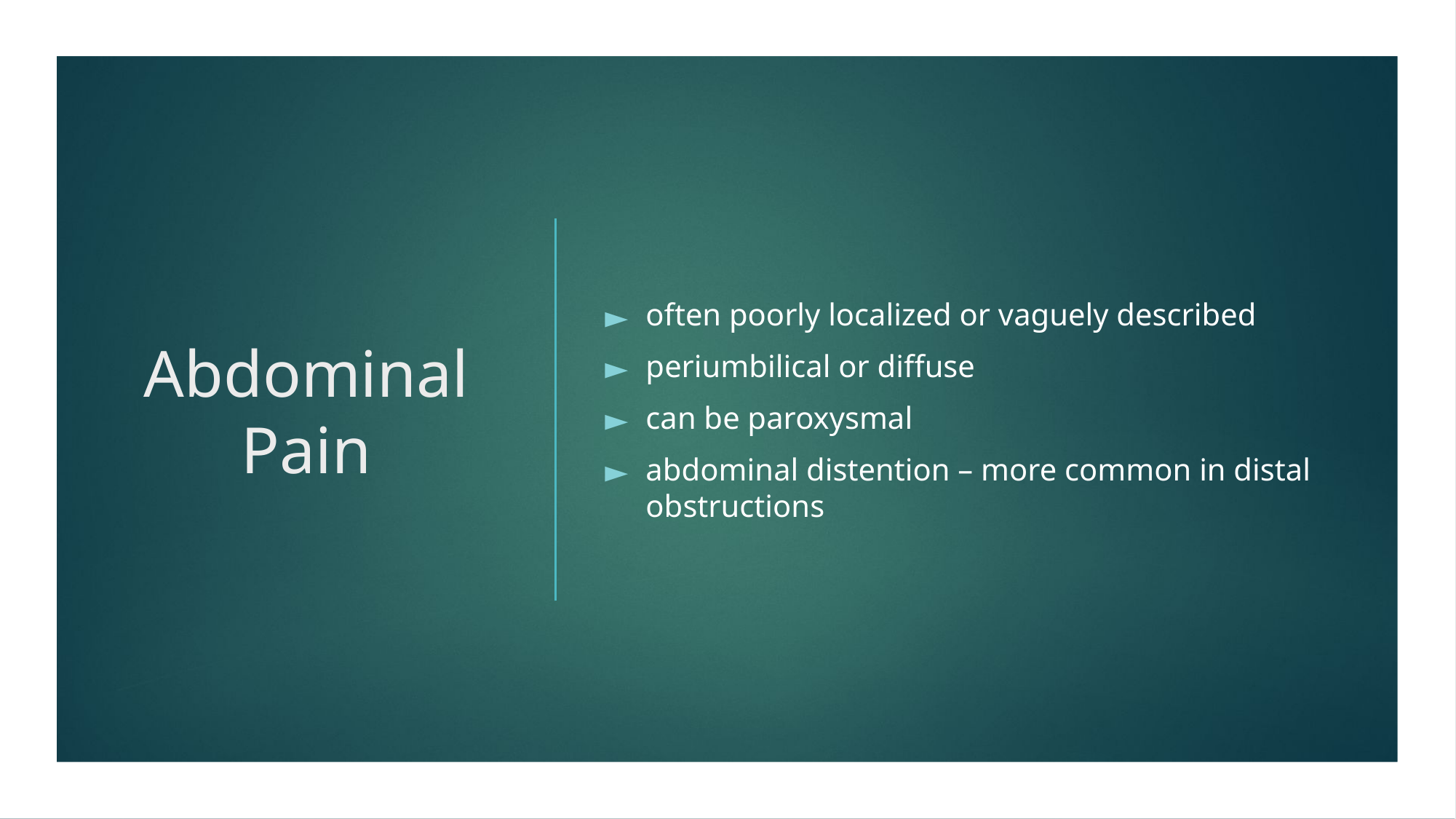

often poorly localized or vaguely described
periumbilical or diffuse
can be paroxysmal
abdominal distention – more common in distal obstructions
# Abdominal Pain

## Slide 10
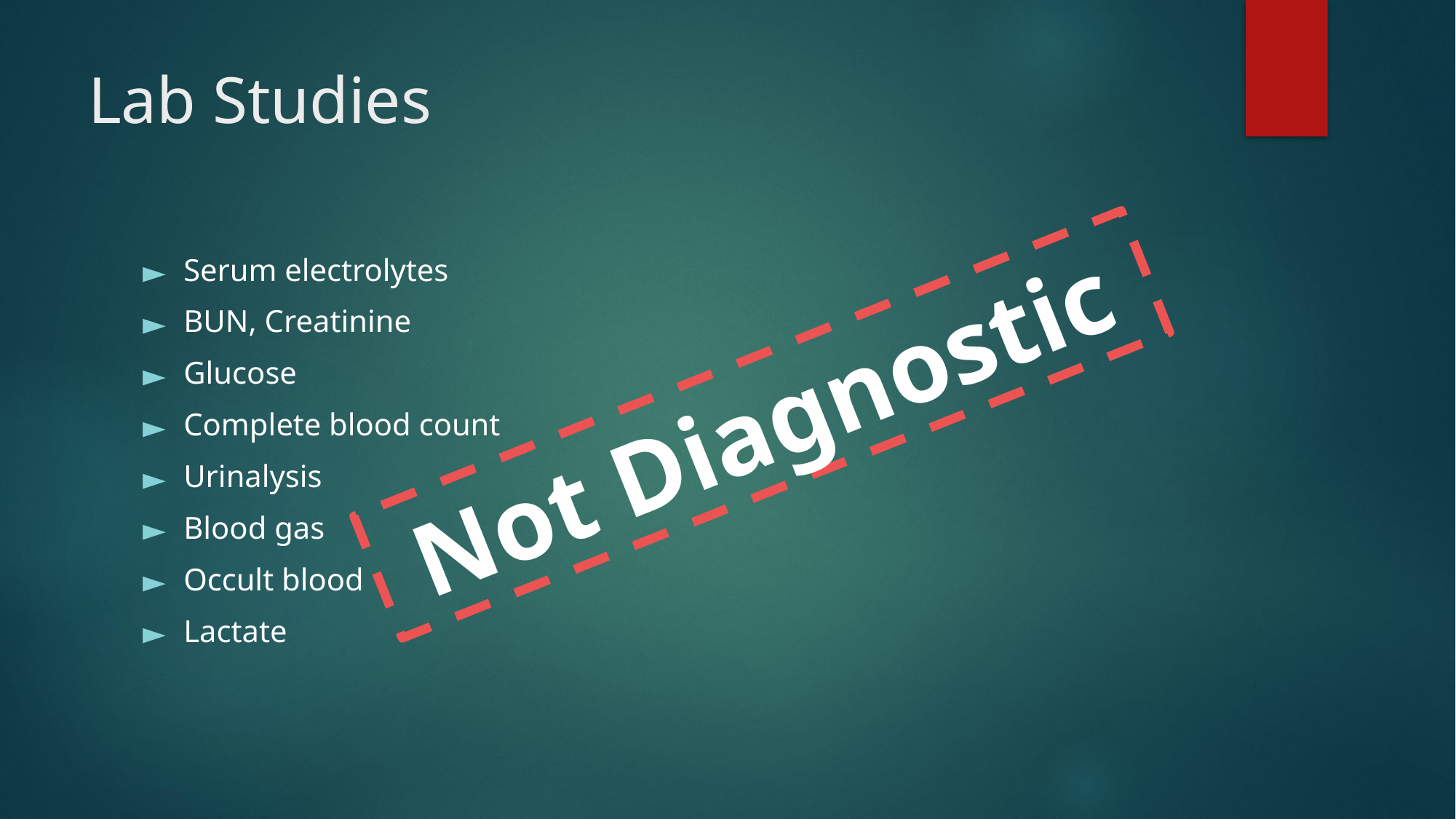

# Lab Studies
Serum electrolytes
BUN, Creatinine
Glucose
Complete blood count
Urinalysis
Blood gas
Occult blood
Lactate
Not Diagnostic

## Slide 11
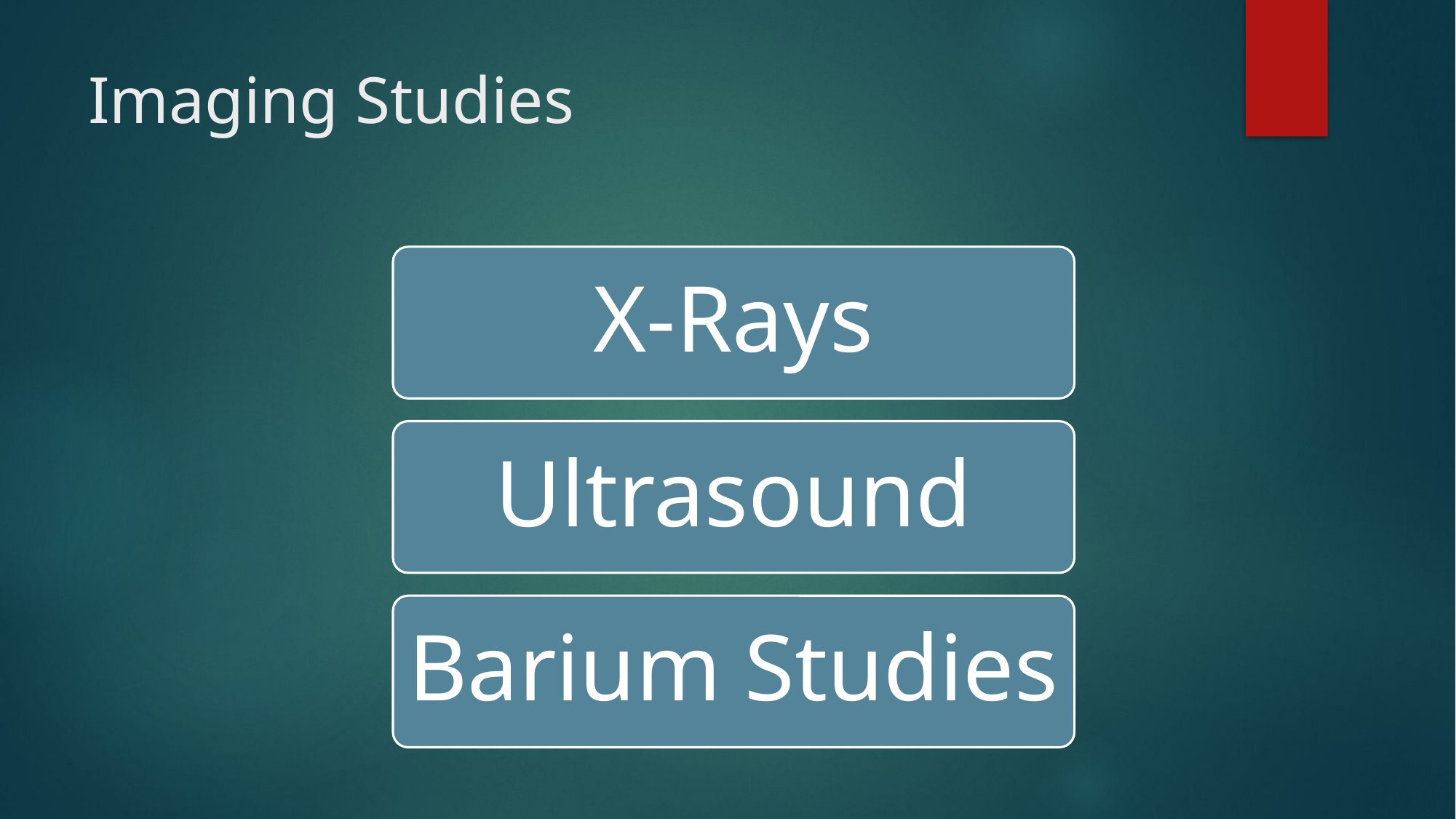

# Imaging Studies
X-Rays
Ultrasound
Barium Studies

## Slide 12
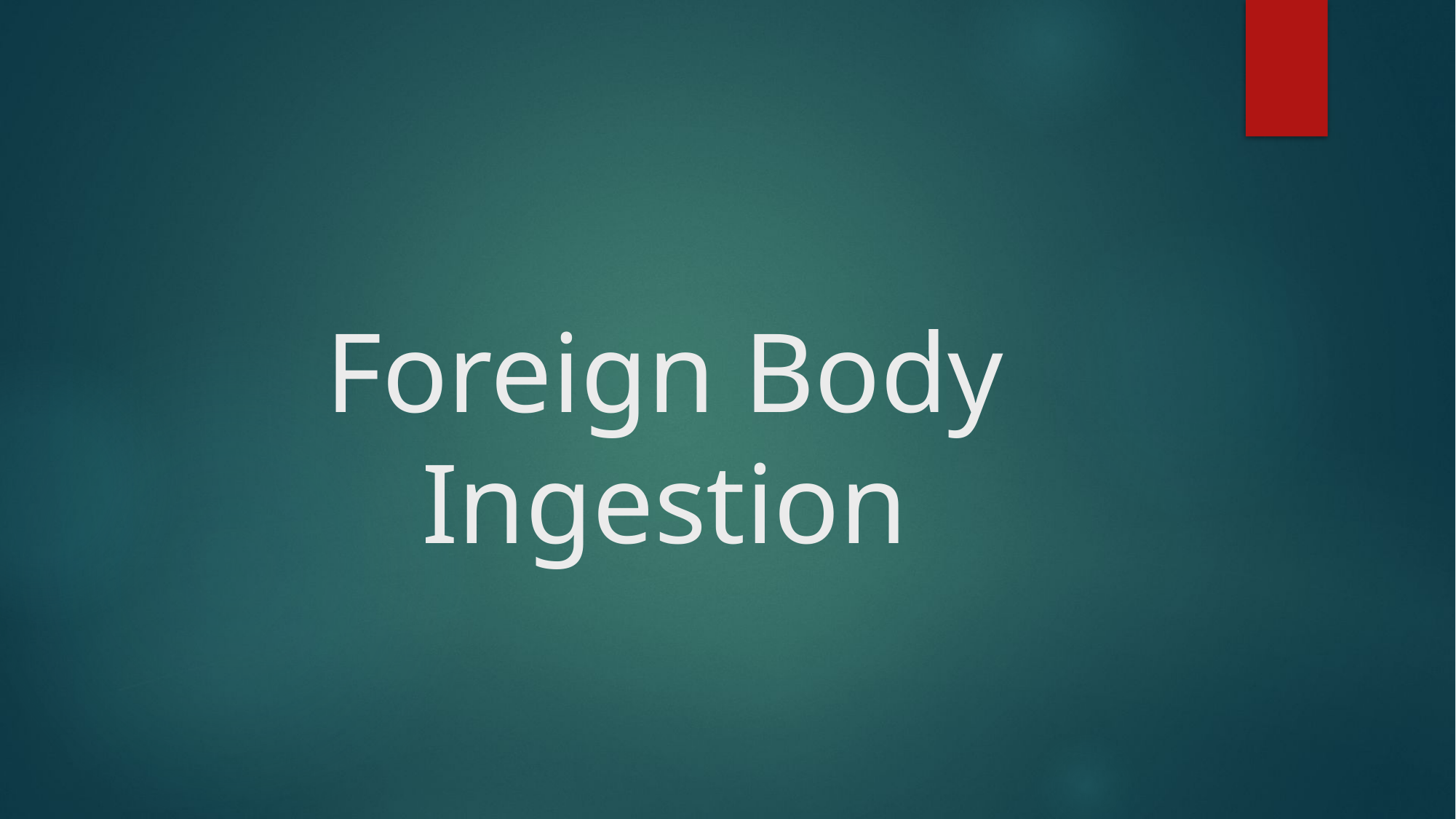

# Foreign Body Ingestion

## Slide 13
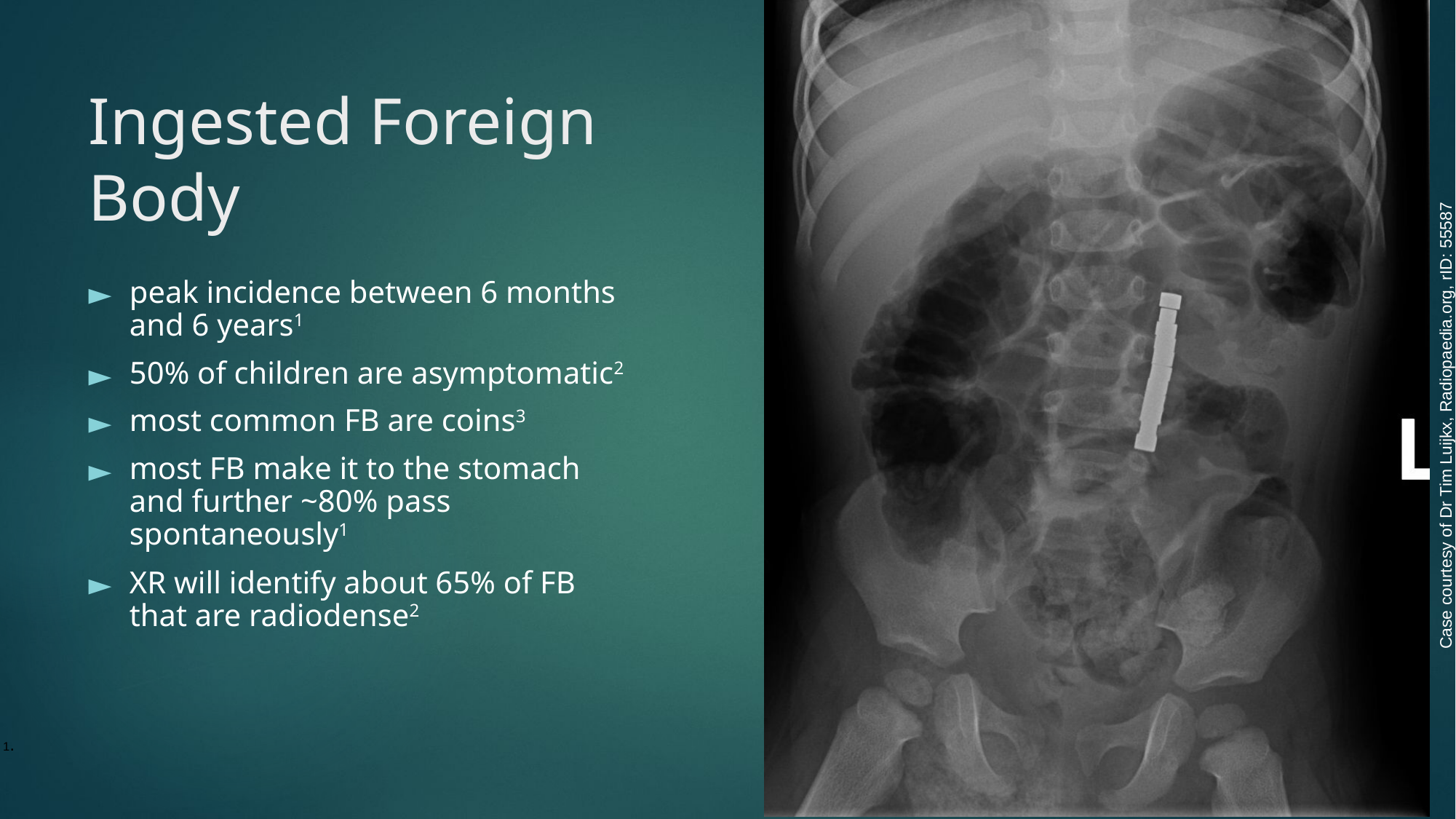

# Ingested Foreign Body
peak incidence between 6 months and 6 years1
50% of children are asymptomatic2
most common FB are coins3
most FB make it to the stomach and further ~80% pass spontaneously1
XR will identify about 65% of FB that are radiodense2
Case courtesy of Dr Tim Luijkx, Radiopaedia.org, rID: 55587
1.

## Slide 14
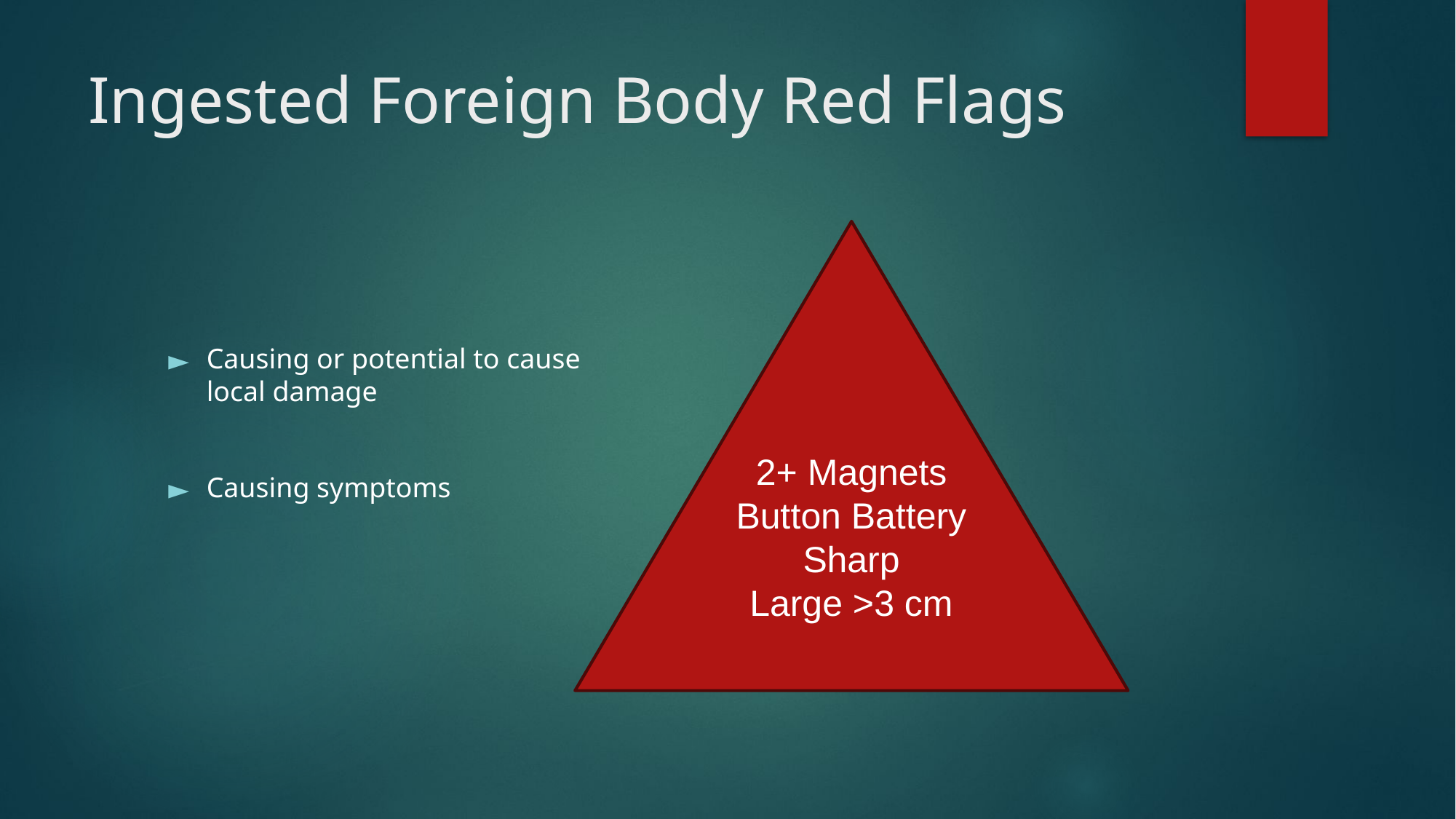

# Ingested Foreign Body Red Flags
Causing or potential to cause local damage
Causing symptoms
2+ Magnets
Button Battery
Sharp
Large >3 cm

## Slide 15
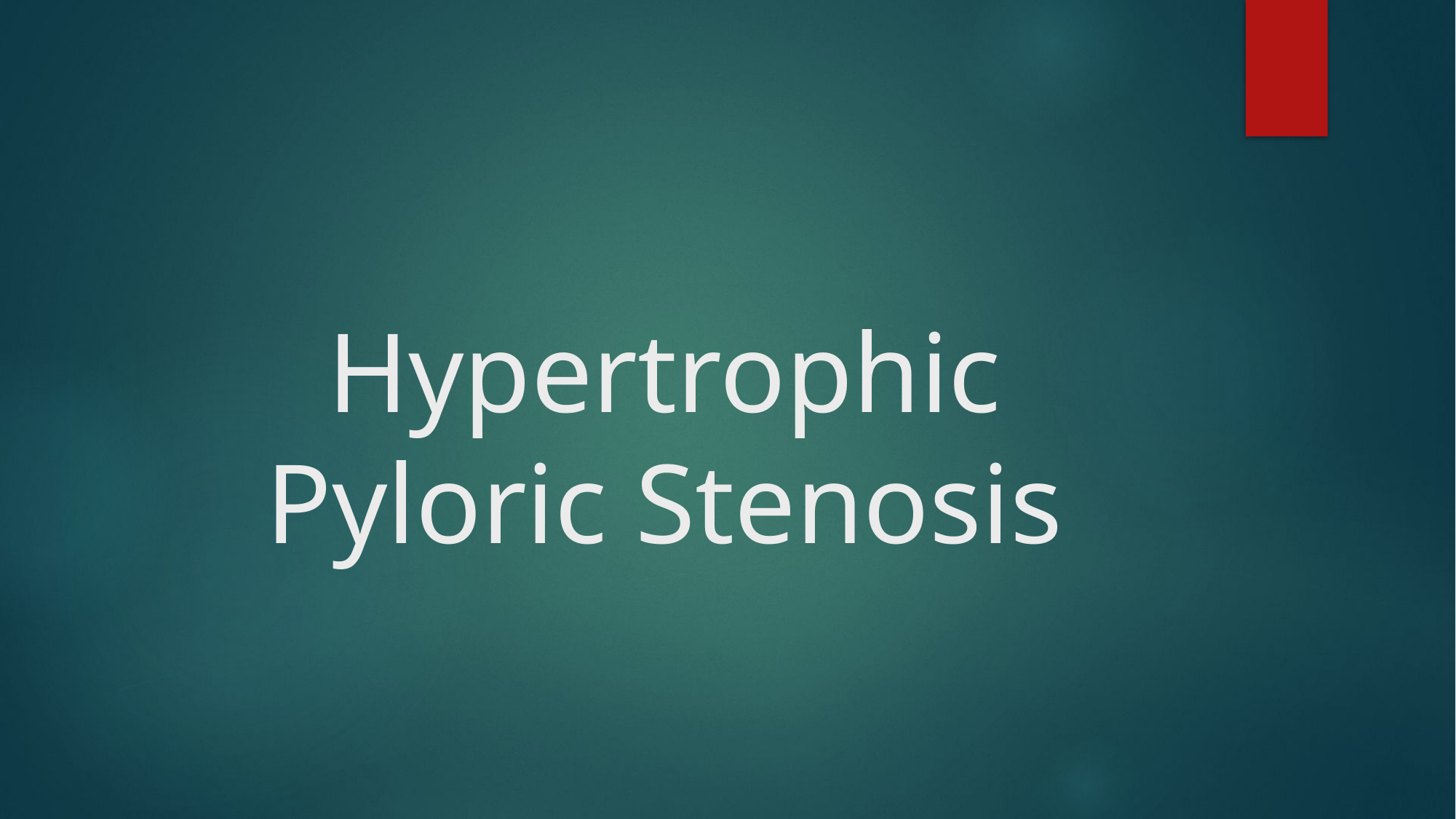

# Hypertrophic Pyloric Stenosis

## Slide 16
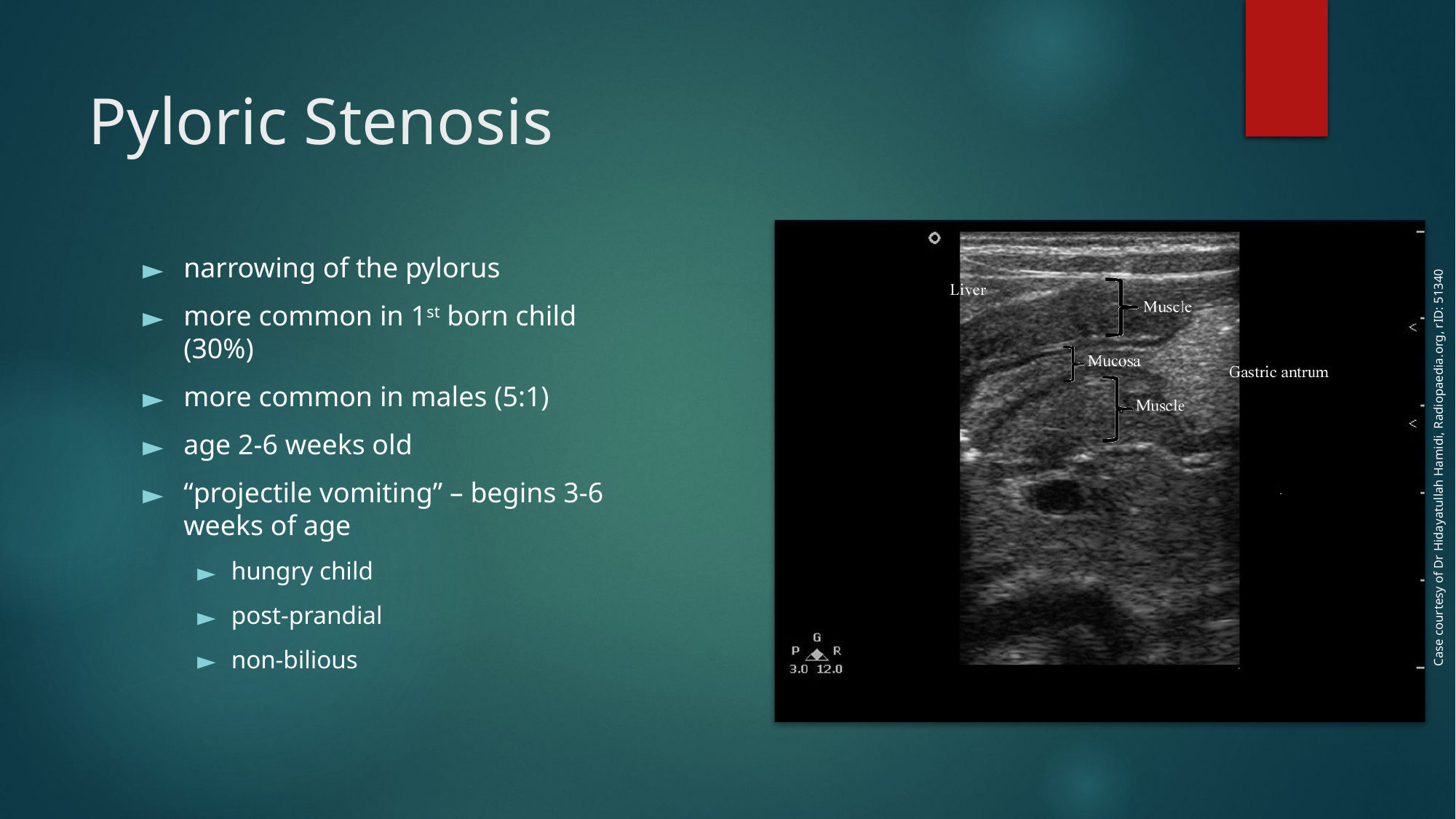

# Pyloric Stenosis
narrowing of the pylorus
more common in 1st born child (30%)
more common in males (5:1)
age 2-6 weeks old
“projectile vomiting” – begins 3-6 weeks of age
hungry child
post-prandial
non-bilious
Case courtesy of Dr Hidayatullah Hamidi, Radiopaedia.org, rID: 51340

## Slide 17
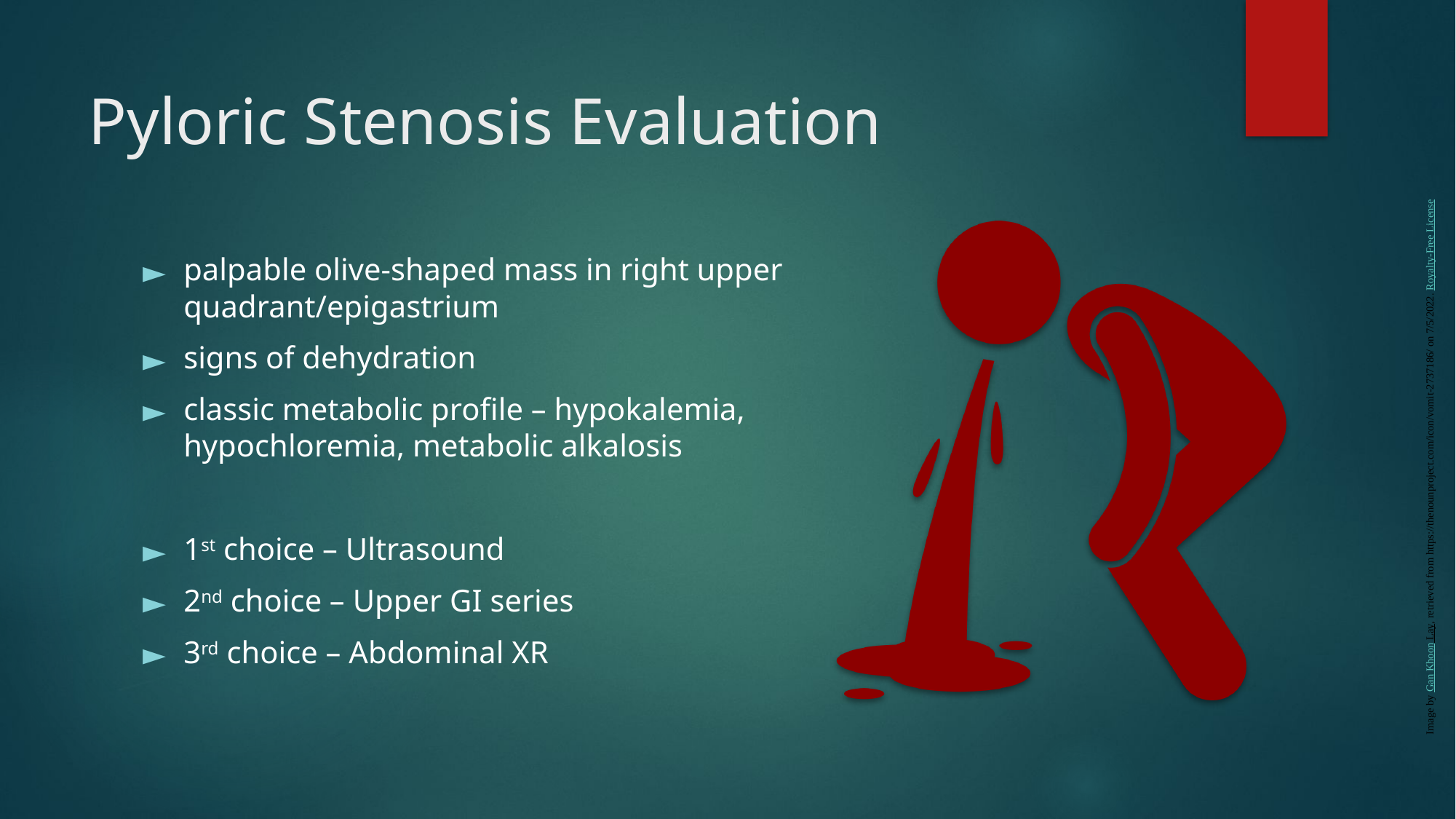

# Pyloric Stenosis Evaluation
palpable olive-shaped mass in right upper quadrant/epigastrium
signs of dehydration
classic metabolic profile – hypokalemia, hypochloremia, metabolic alkalosis
1st choice – Ultrasound
2nd choice – Upper GI series
3rd choice – Abdominal XR
Image by Gan Khoon Lay, retrieved from https://thenounproject.com/icon/vomit-2737186/ on 7/5/2022. Royalty-Free License

## Slide 18
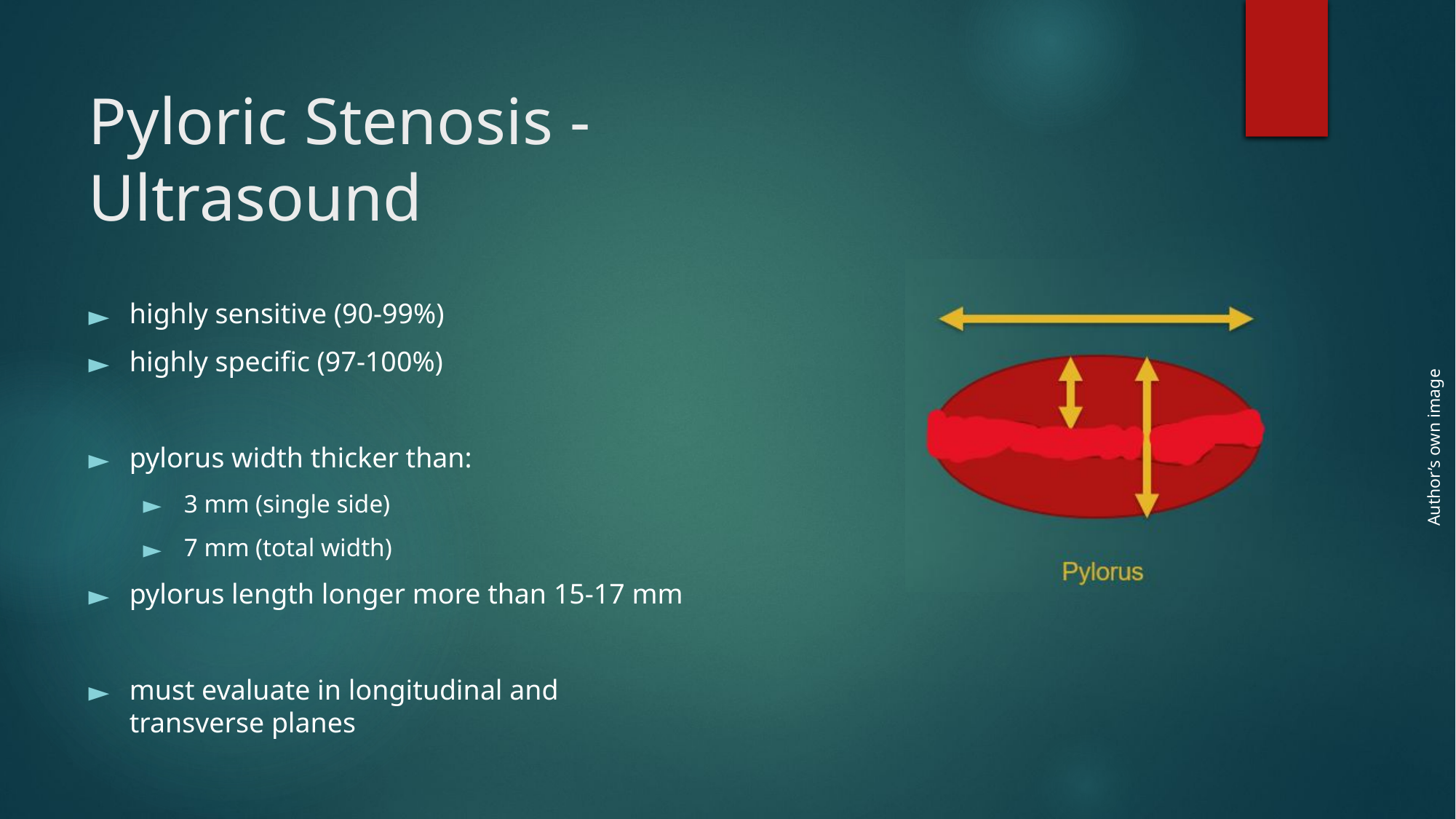

# Pyloric Stenosis - Ultrasound
highly sensitive (90-99%)
highly specific (97-100%)
pylorus width thicker than:
3 mm (single side)
7 mm (total width)
pylorus length longer more than 15-17 mm
must evaluate in longitudinal and transverse planes
Author’s own image

## Slide 19
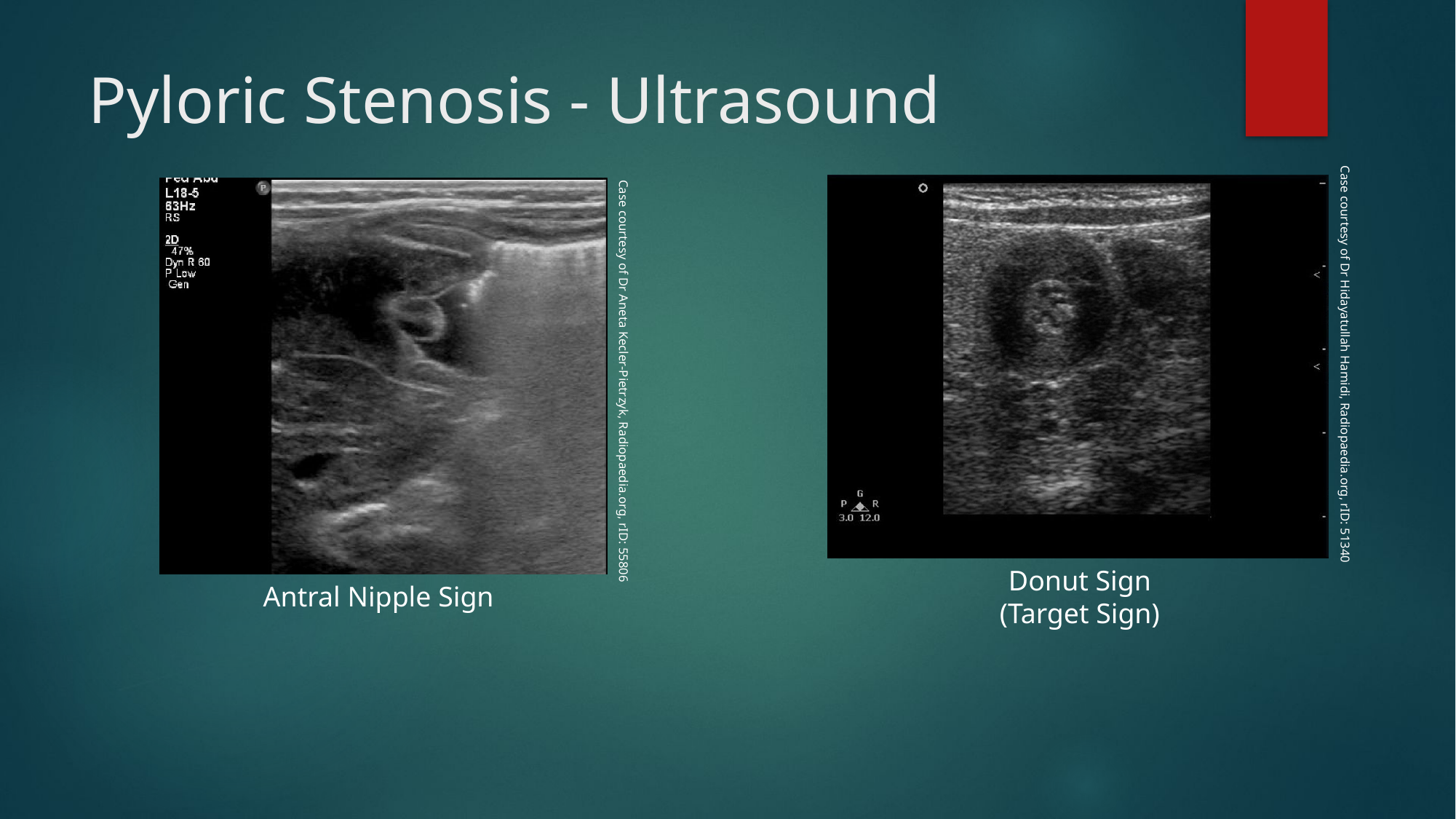

# Pyloric Stenosis - Ultrasound
Case courtesy of Dr Hidayatullah Hamidi, Radiopaedia.org, rID: 51340
Case courtesy of Dr Aneta Kecler-Pietrzyk, Radiopaedia.org, rID: 55806
Donut Sign
(Target Sign)
Antral Nipple Sign

## Slide 20
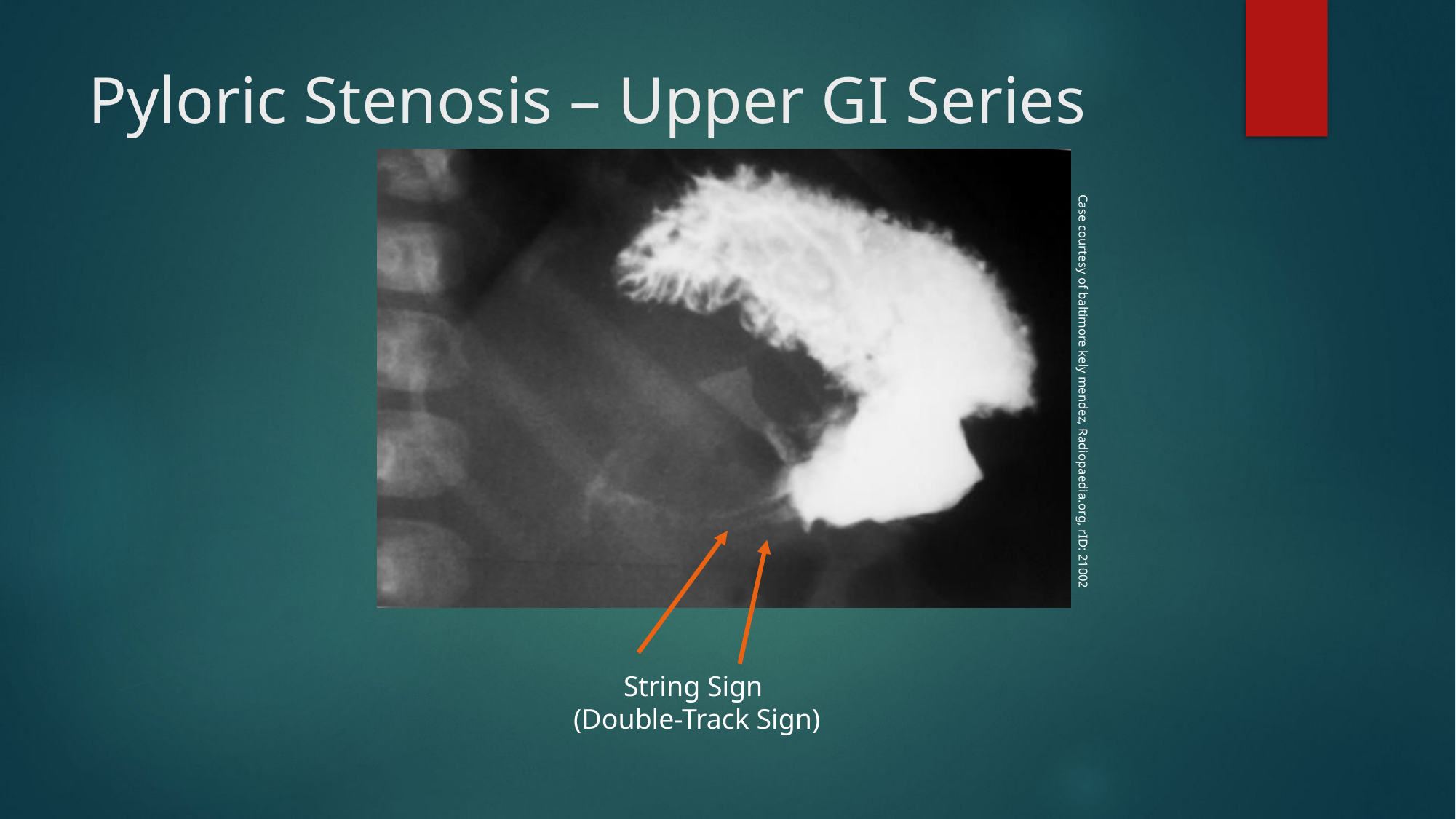

# Pyloric Stenosis – Upper GI Series
Case courtesy of baltimore kely mendez, Radiopaedia.org, rID: 21002
String Sign
(Double-Track Sign)

## Slide 21
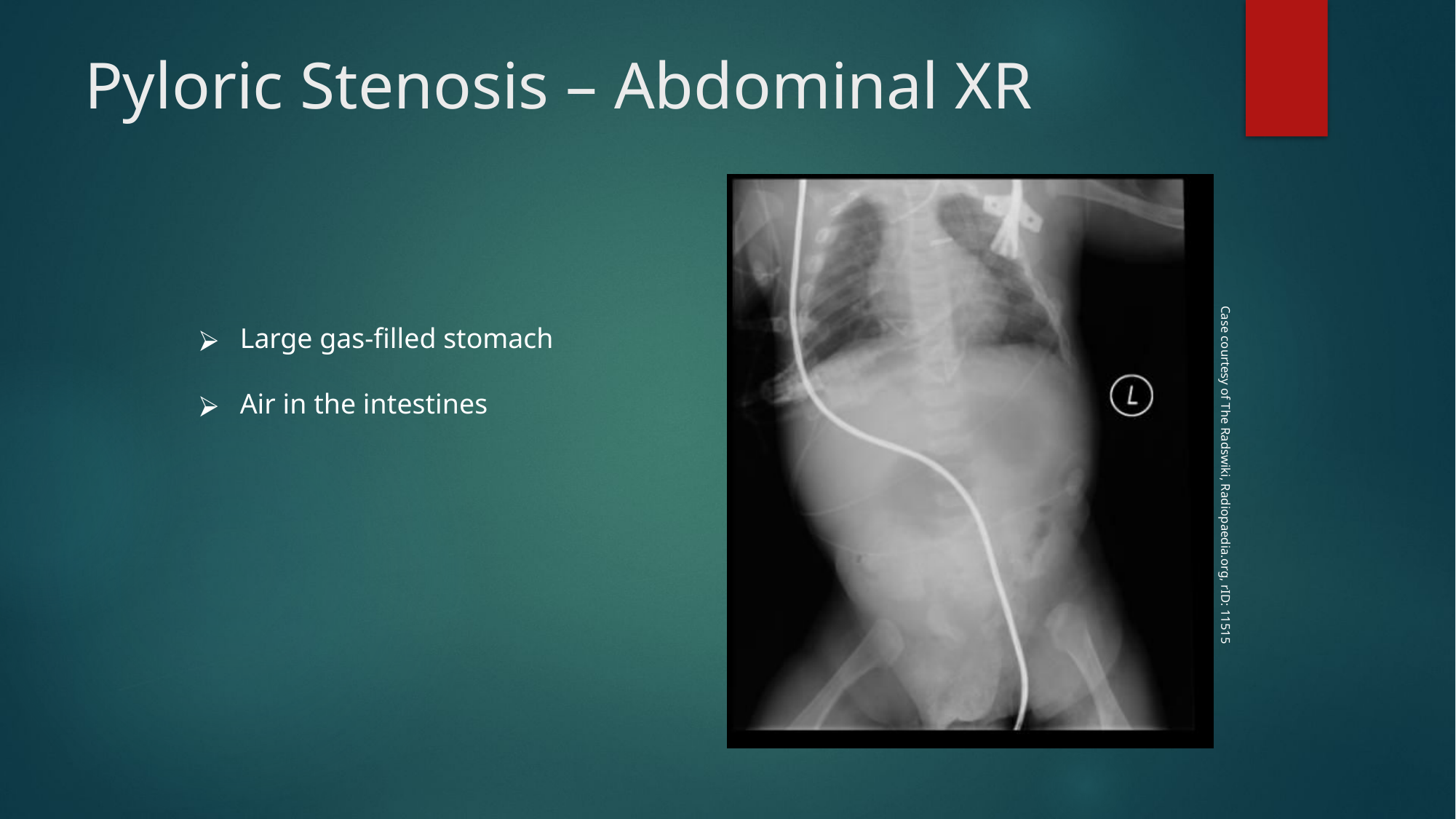

# Pyloric Stenosis – Abdominal XR
Large gas-filled stomach
Air in the intestines
Case courtesy of The Radswiki, Radiopaedia.org, rID: 11515

## Slide 22
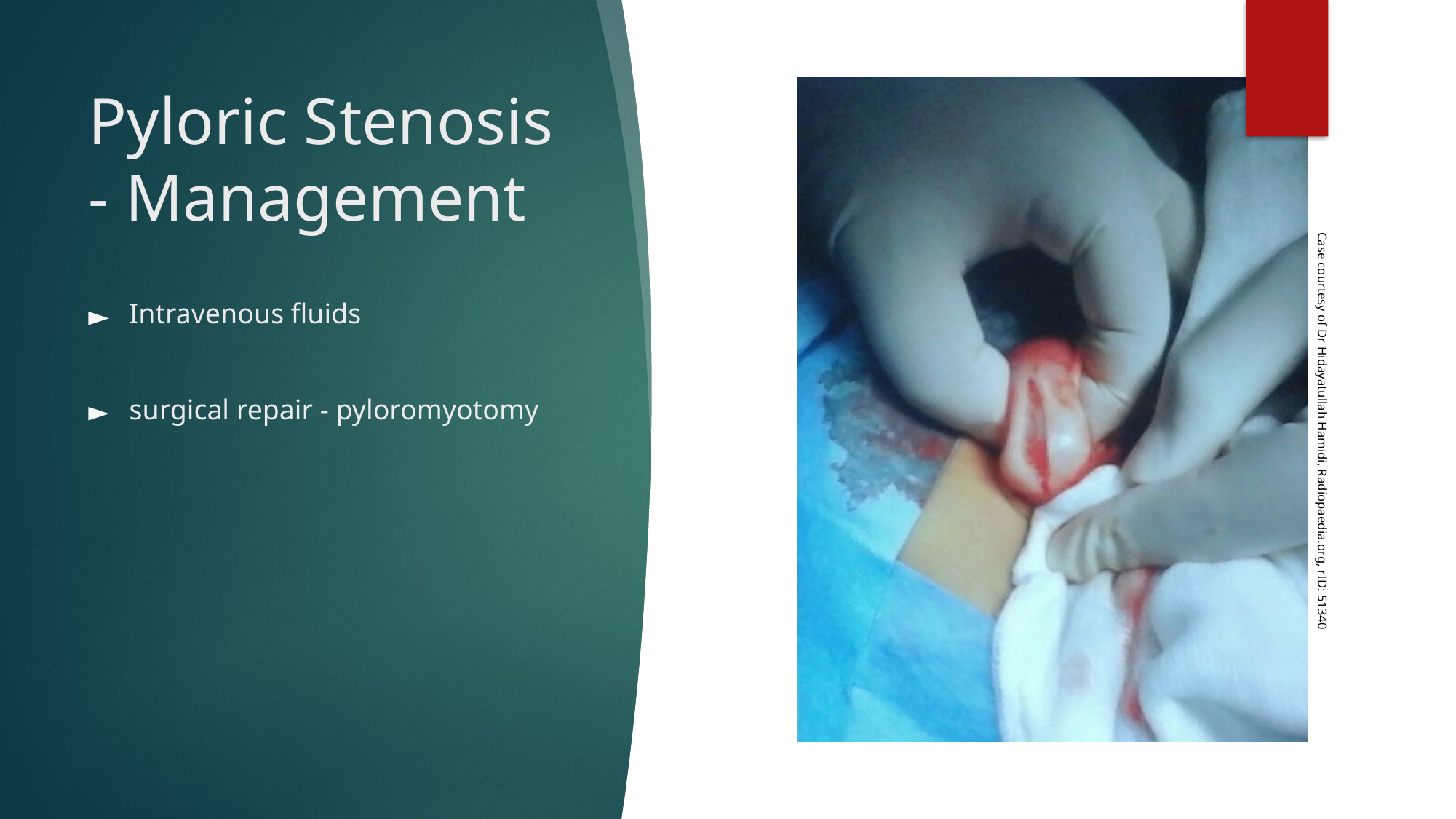

# Pyloric Stenosis - Management
Intravenous fluids
surgical repair - pyloromyotomy
Case courtesy of Dr Hidayatullah Hamidi, Radiopaedia.org, rID: 51340

## Slide 23
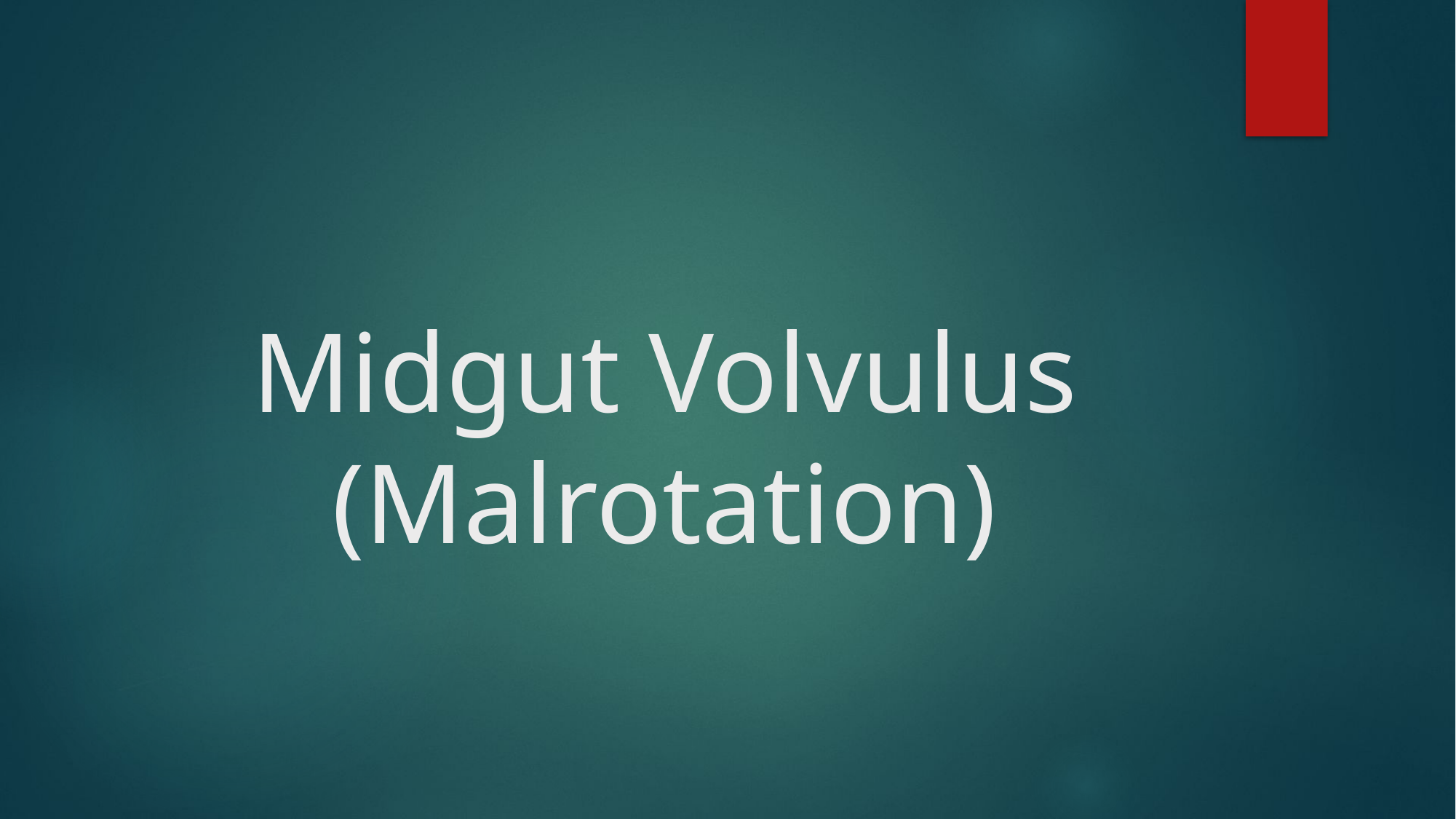

# Midgut Volvulus (Malrotation)

## Slide 24
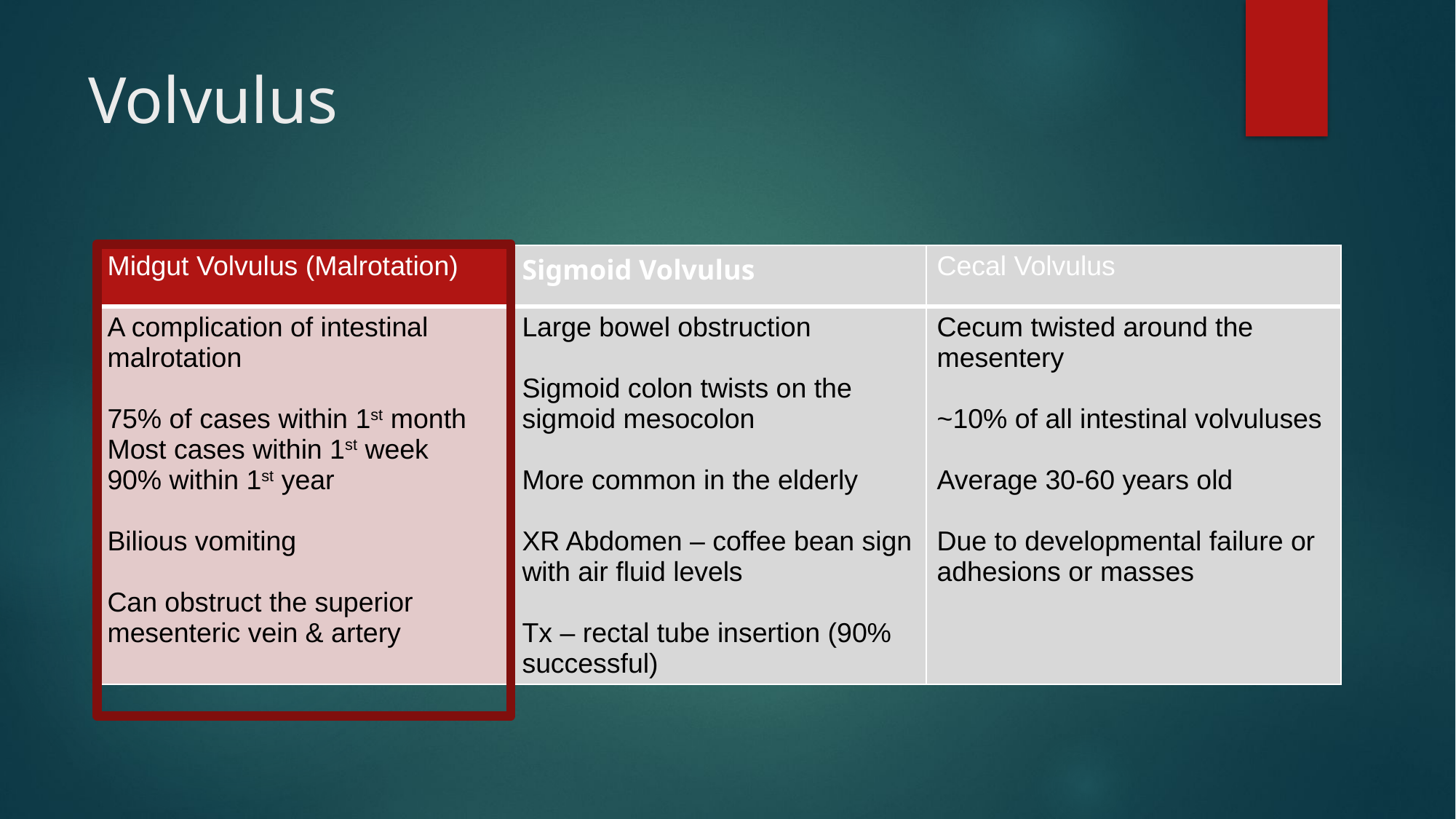

# Volvulus
| Midgut Volvulus (Malrotation) | Sigmoid Volvulus | Cecal Volvulus |
| --- | --- | --- |
| A complication of intestinal malrotation 75% of cases within 1st month Most cases within 1st week 90% within 1st year Bilious vomiting Can obstruct the superior mesenteric vein & artery | Large bowel obstruction Sigmoid colon twists on the sigmoid mesocolon More common in the elderly XR Abdomen – coffee bean sign with air fluid levels Tx – rectal tube insertion (90% successful) | Cecum twisted around the mesentery ~10% of all intestinal volvuluses Average 30-60 years old Due to developmental failure or adhesions or masses |

## Slide 25
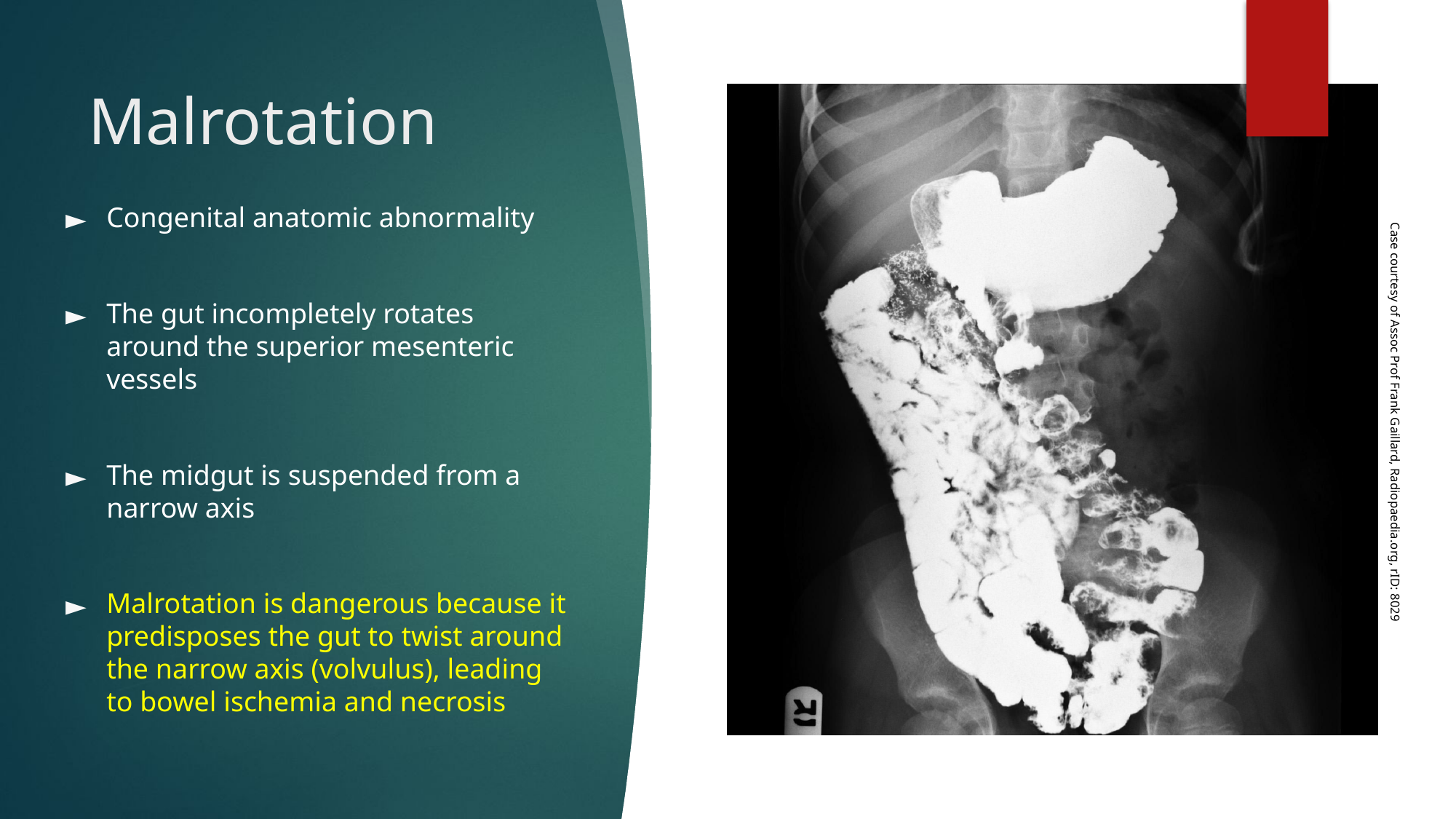

# Malrotation
Congenital anatomic abnormality
The gut incompletely rotates around the superior mesenteric vessels
The midgut is suspended from a narrow axis
Malrotation is dangerous because it predisposes the gut to twist around the narrow axis (volvulus), leading to bowel ischemia and necrosis
Case courtesy of Assoc Prof Frank Gaillard, Radiopaedia.org, rID: 8029

## Slide 26
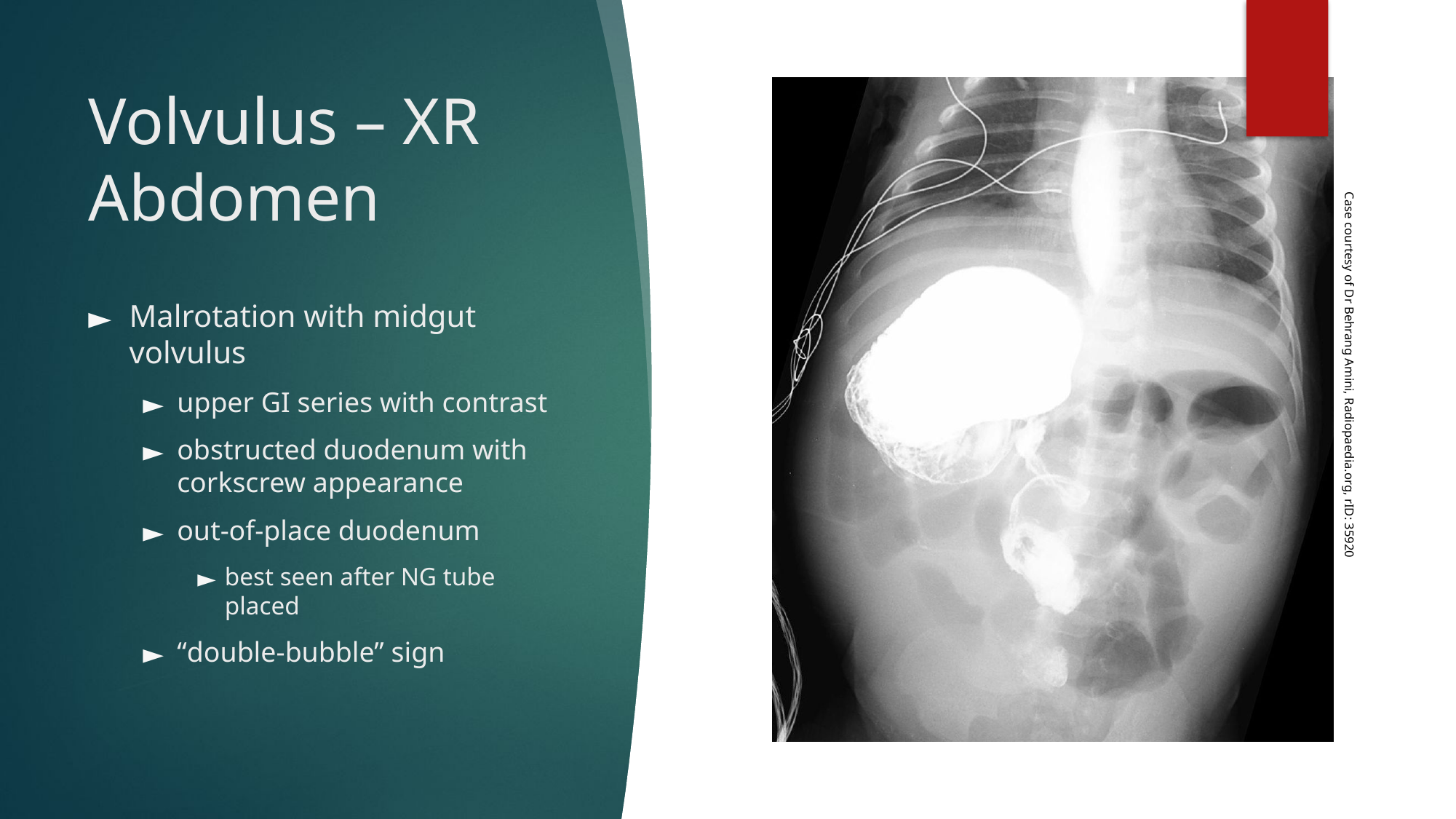

# Volvulus – XR Abdomen
Malrotation with midgut volvulus
upper GI series with contrast
obstructed duodenum with corkscrew appearance
out-of-place duodenum
best seen after NG tube placed
“double-bubble” sign
Case courtesy of Dr Behrang Amini, Radiopaedia.org, rID: 35920

## Slide 27
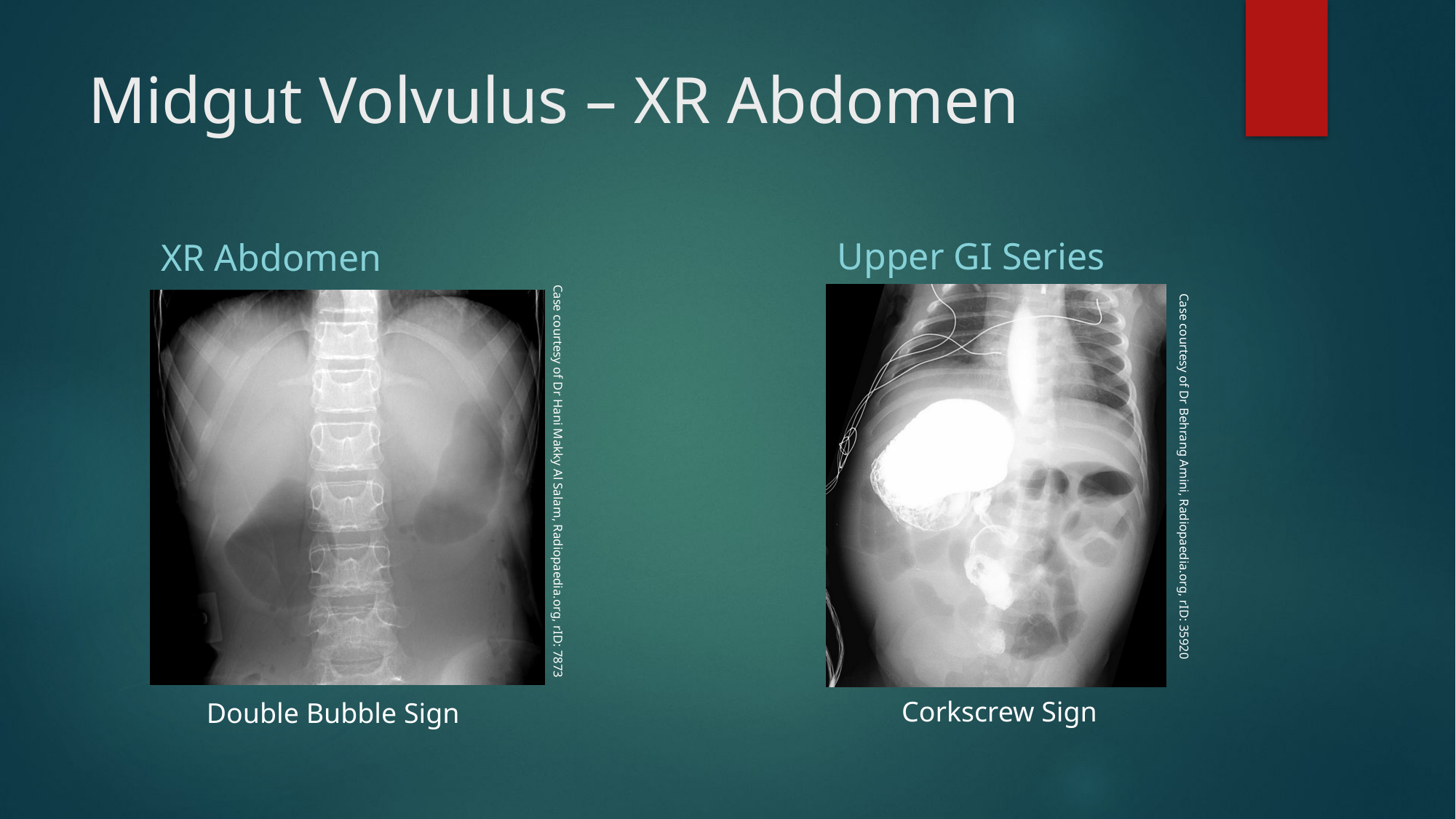

# Midgut Volvulus – XR Abdomen
Upper GI Series
XR Abdomen
Case courtesy of Dr Hani Makky Al Salam, Radiopaedia.org, rID: 7873
Case courtesy of Dr Behrang Amini, Radiopaedia.org, rID: 35920
Corkscrew Sign
Double Bubble Sign

## Slide 28
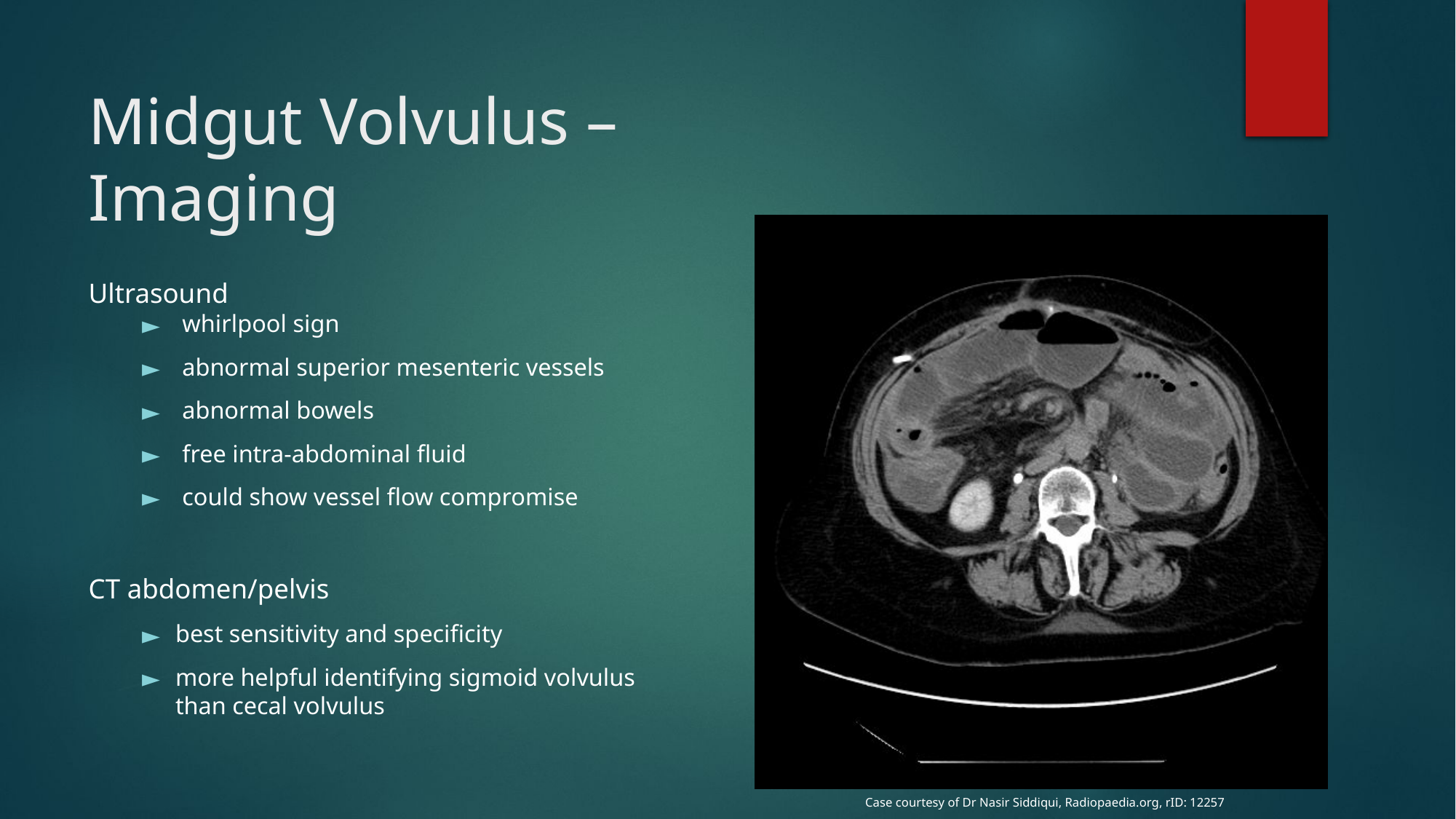

# Midgut Volvulus – Imaging
Ultrasound
whirlpool sign
abnormal superior mesenteric vessels
abnormal bowels
free intra-abdominal fluid
could show vessel flow compromise
CT abdomen/pelvis
best sensitivity and specificity
more helpful identifying sigmoid volvulus than cecal volvulus
Case courtesy of Dr Nasir Siddiqui, Radiopaedia.org, rID: 12257

## Slide 29
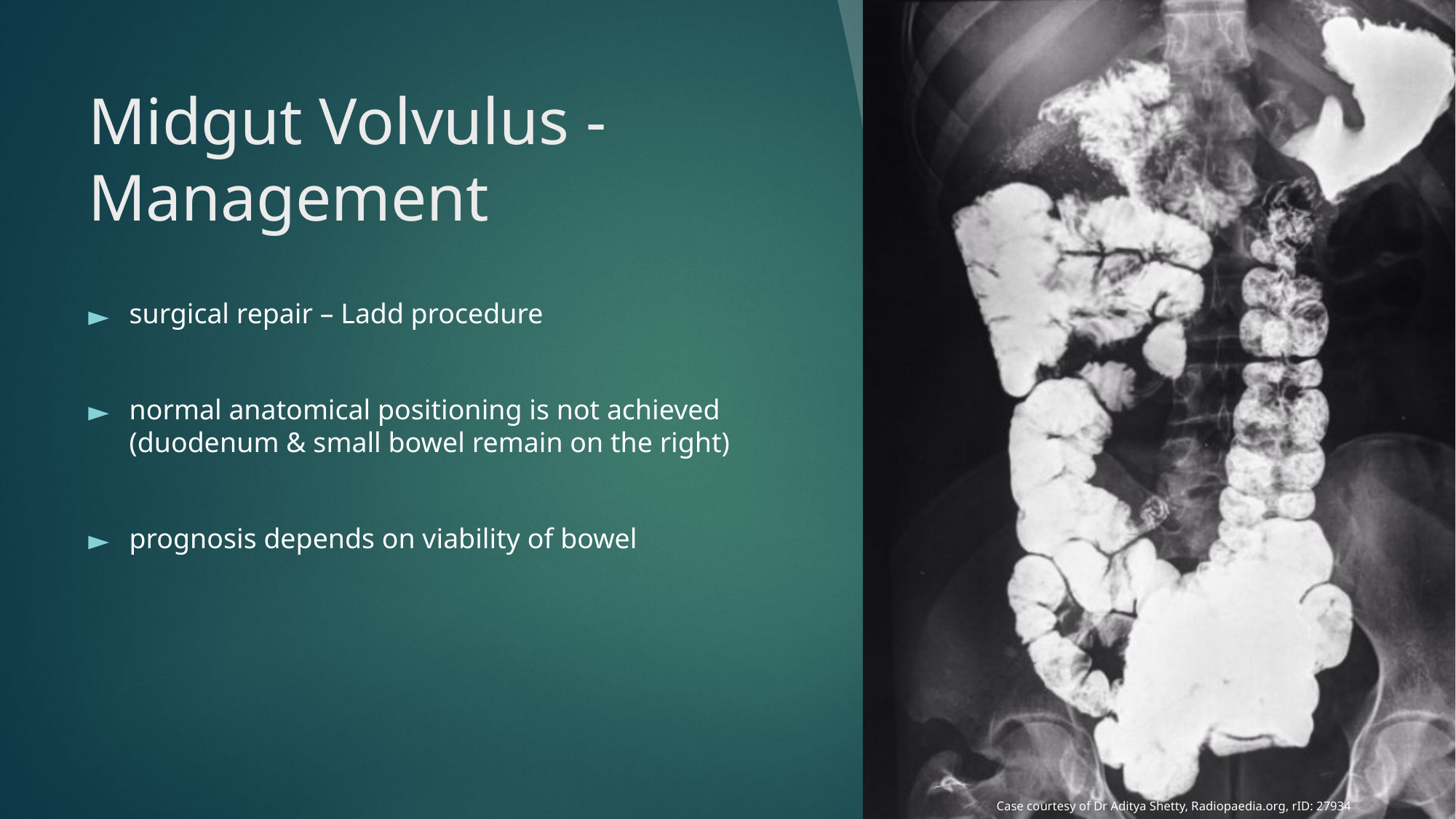

# Midgut Volvulus - Management
surgical repair – Ladd procedure
normal anatomical positioning is not achieved (duodenum & small bowel remain on the right)
prognosis depends on viability of bowel
Case courtesy of Dr Aditya Shetty, Radiopaedia.org, rID: 27934

## Slide 30
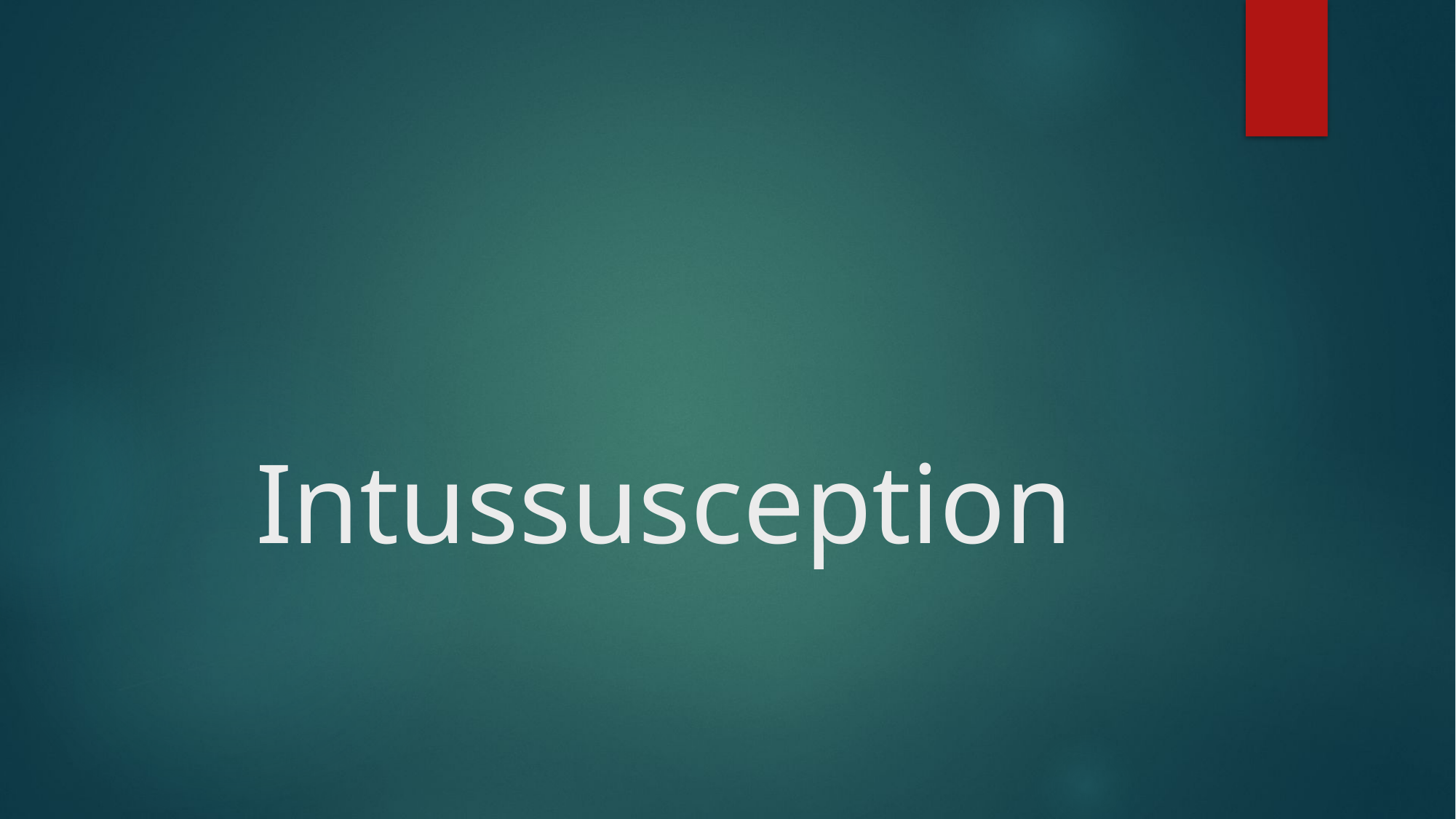

# Intussusception

## Slide 31
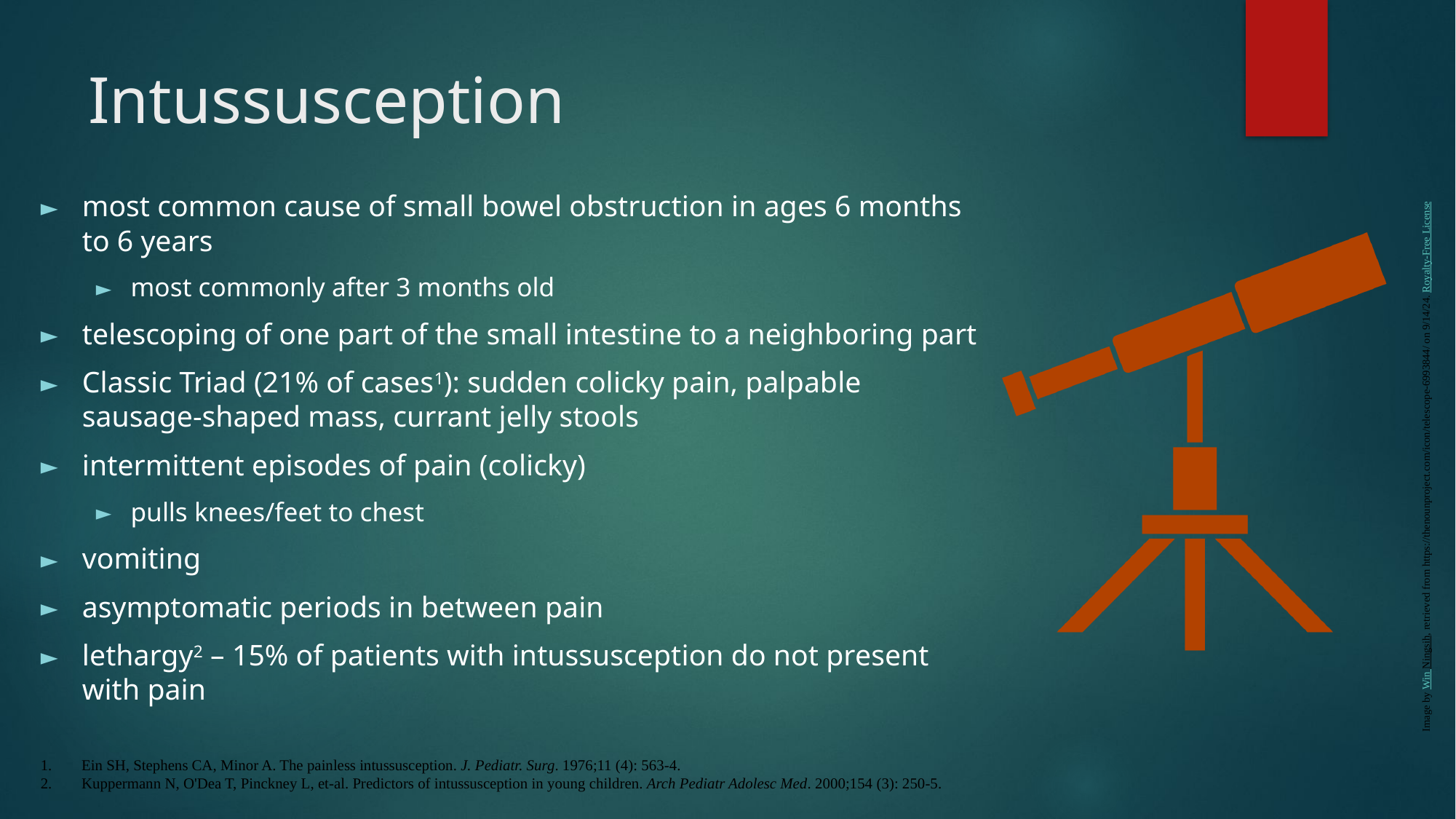

# Intussusception
most common cause of small bowel obstruction in ages 6 months to 6 years
most commonly after 3 months old
telescoping of one part of the small intestine to a neighboring part
Classic Triad (21% of cases1): sudden colicky pain, palpable sausage-shaped mass, currant jelly stools
intermittent episodes of pain (colicky)
pulls knees/feet to chest
vomiting
asymptomatic periods in between pain
lethargy2 – 15% of patients with intussusception do not present with pain
Image by Win Ningsih, retrieved from https://thenounproject.com/icon/telescope-6993844/ on 9/14/24. Royalty-Free License
Ein SH, Stephens CA, Minor A. The painless intussusception. J. Pediatr. Surg. 1976;11 (4): 563-4.
Kuppermann N, O'Dea T, Pinckney L, et-al. Predictors of intussusception in young children. Arch Pediatr Adolesc Med. 2000;154 (3): 250-5.

## Slide 32
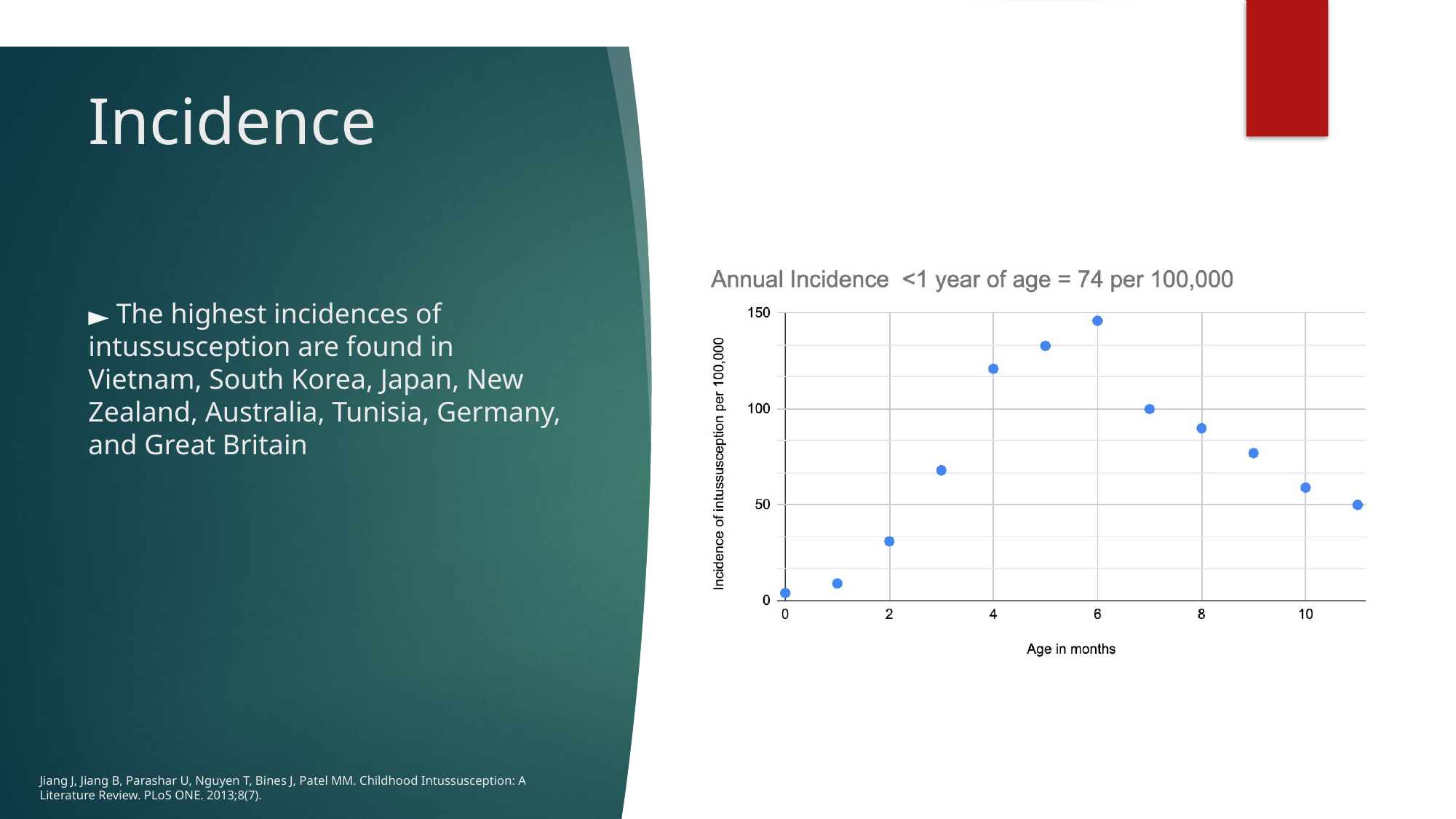

# Incidence
 The highest incidences of intussusception are found in Vietnam, South Korea, Japan, New Zealand, Australia, Tunisia, Germany, and Great Britain
Jiang J, Jiang B, Parashar U, Nguyen T, Bines J, Patel MM. Childhood Intussusception: A Literature Review. PLoS ONE. 2013;8(7).

## Slide 33
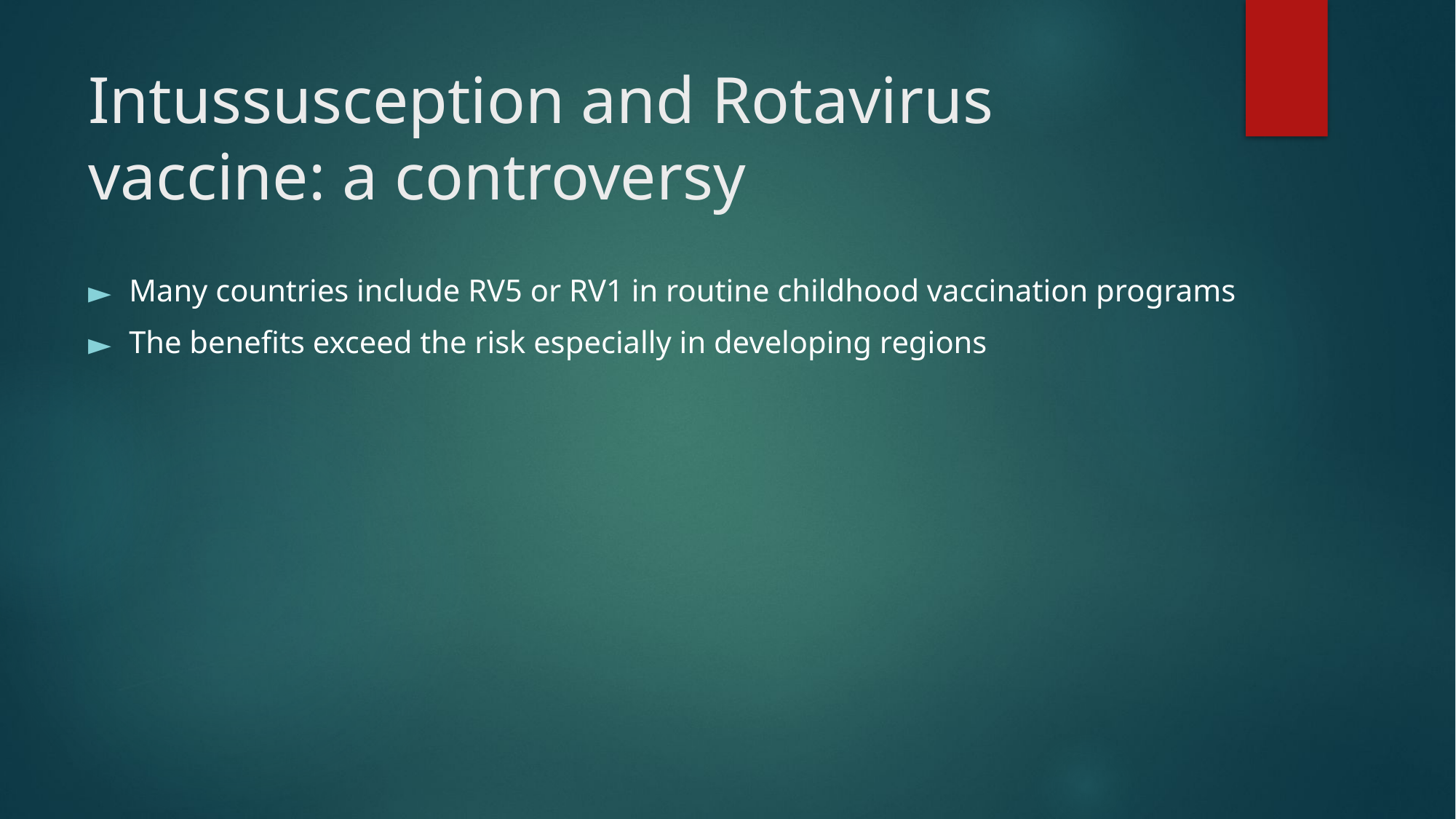

# Intussusception and Rotavirus vaccine: a controversy
Many countries include RV5 or RV1 in routine childhood vaccination programs
The benefits exceed the risk especially in developing regions

## Slide 34
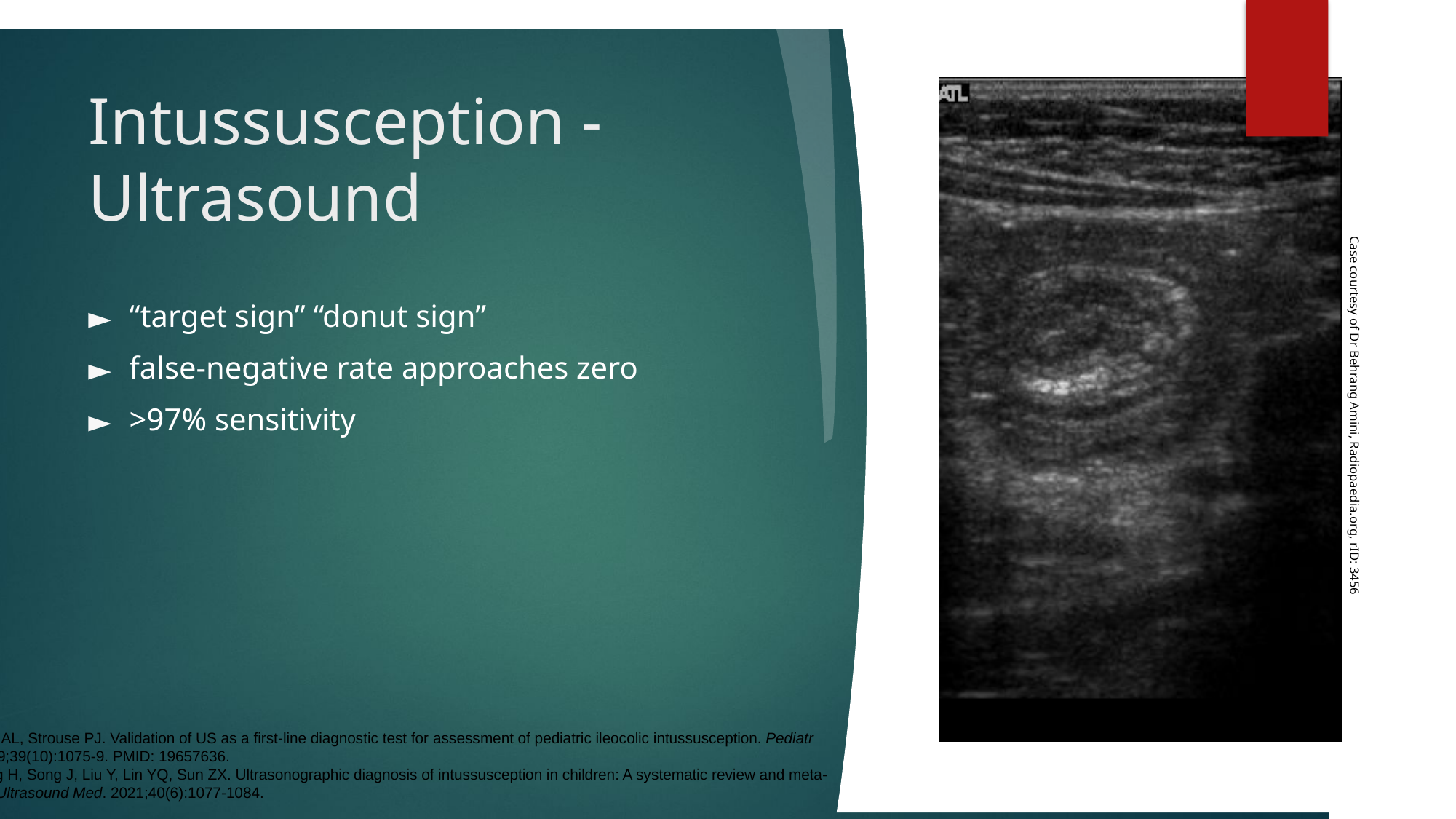

# Intussusception - Ultrasound
“target sign” “donut sign”
false-negative rate approaches zero
>97% sensitivity
Case courtesy of Dr Behrang Amini, Radiopaedia.org, rID: 3456
Hryhorczuk AL, Strouse PJ. Validation of US as a first-line diagnostic test for assessment of pediatric ileocolic intussusception. Pediatr Radiol. 2009;39(10):1075-9. PMID: 19657636.
Li XZ, Wang H, Song J, Liu Y, Lin YQ, Sun ZX. Ultrasonographic diagnosis of intussusception in children: A systematic review and meta-analysis. J Ultrasound Med. 2021;40(6):1077-1084.

## Slide 35
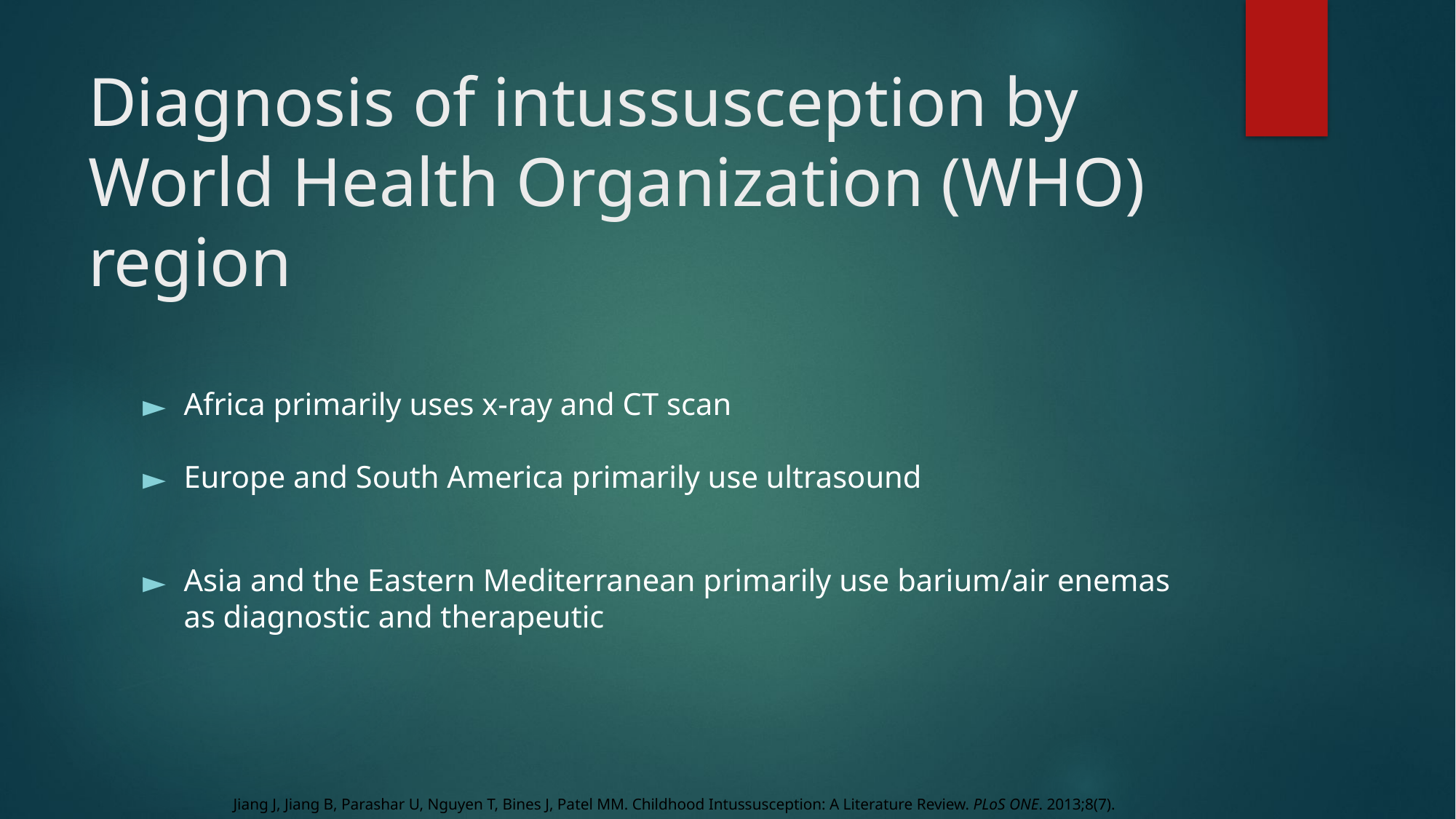

# Diagnosis of intussusception by World Health Organization (WHO) region
Africa primarily uses x-ray and CT scan
Europe and South America primarily use ultrasound
Asia and the Eastern Mediterranean primarily use barium/air enemas as diagnostic and therapeutic
Jiang J, Jiang B, Parashar U, Nguyen T, Bines J, Patel MM. Childhood Intussusception: A Literature Review. PLoS ONE. 2013;8(7).

## Slide 36
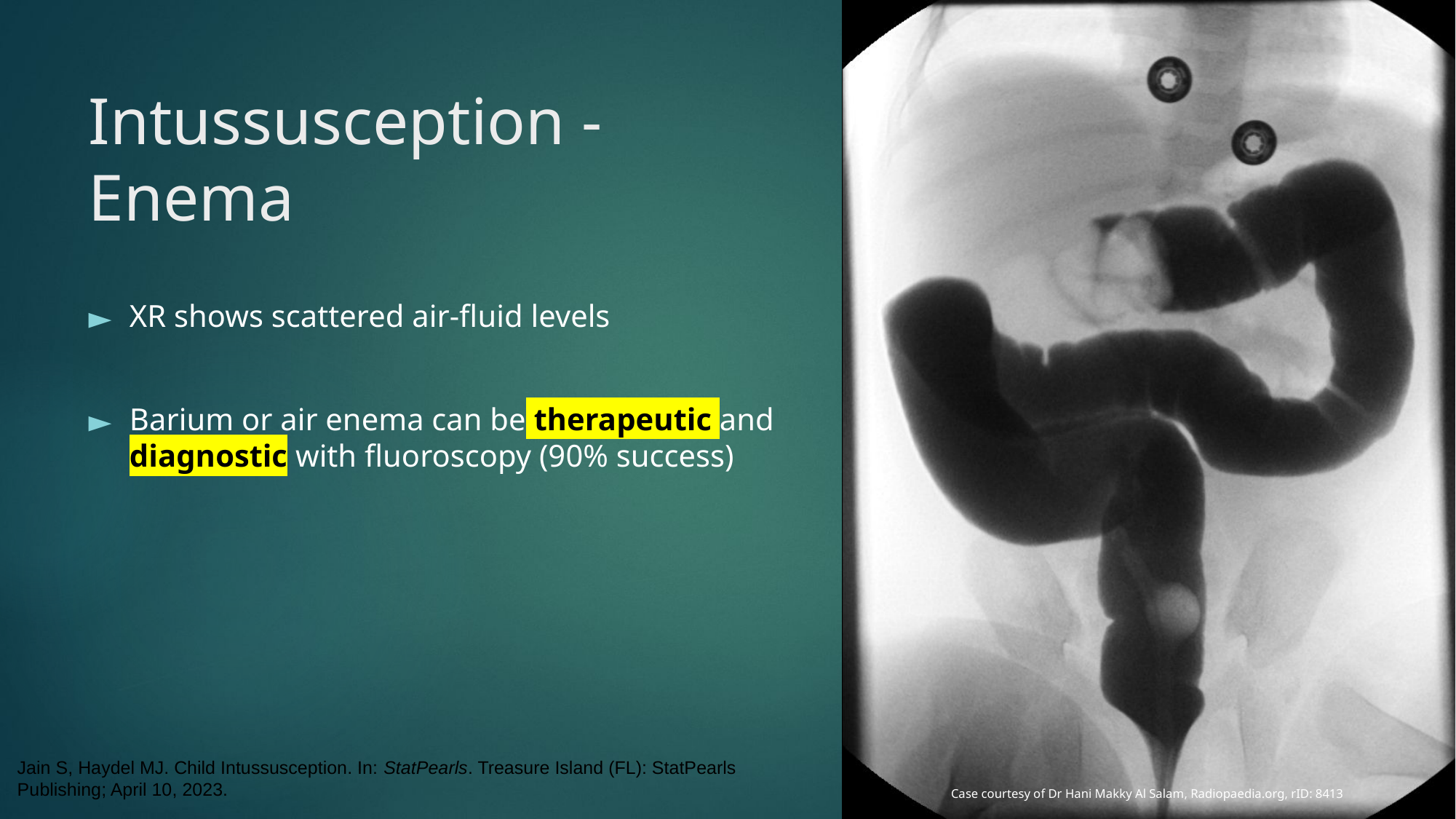

# Intussusception - Enema
XR shows scattered air-fluid levels
Barium or air enema can be therapeutic and diagnostic with fluoroscopy (90% success)
Jain S, Haydel MJ. Child Intussusception. In: StatPearls. Treasure Island (FL): StatPearls Publishing; April 10, 2023.
Case courtesy of Dr Hani Makky Al Salam, Radiopaedia.org, rID: 8413
http://www.klinikaikozpont.u-szeged.hu/radiology/radio/surgos/asurg7c.htm

## Slide 37
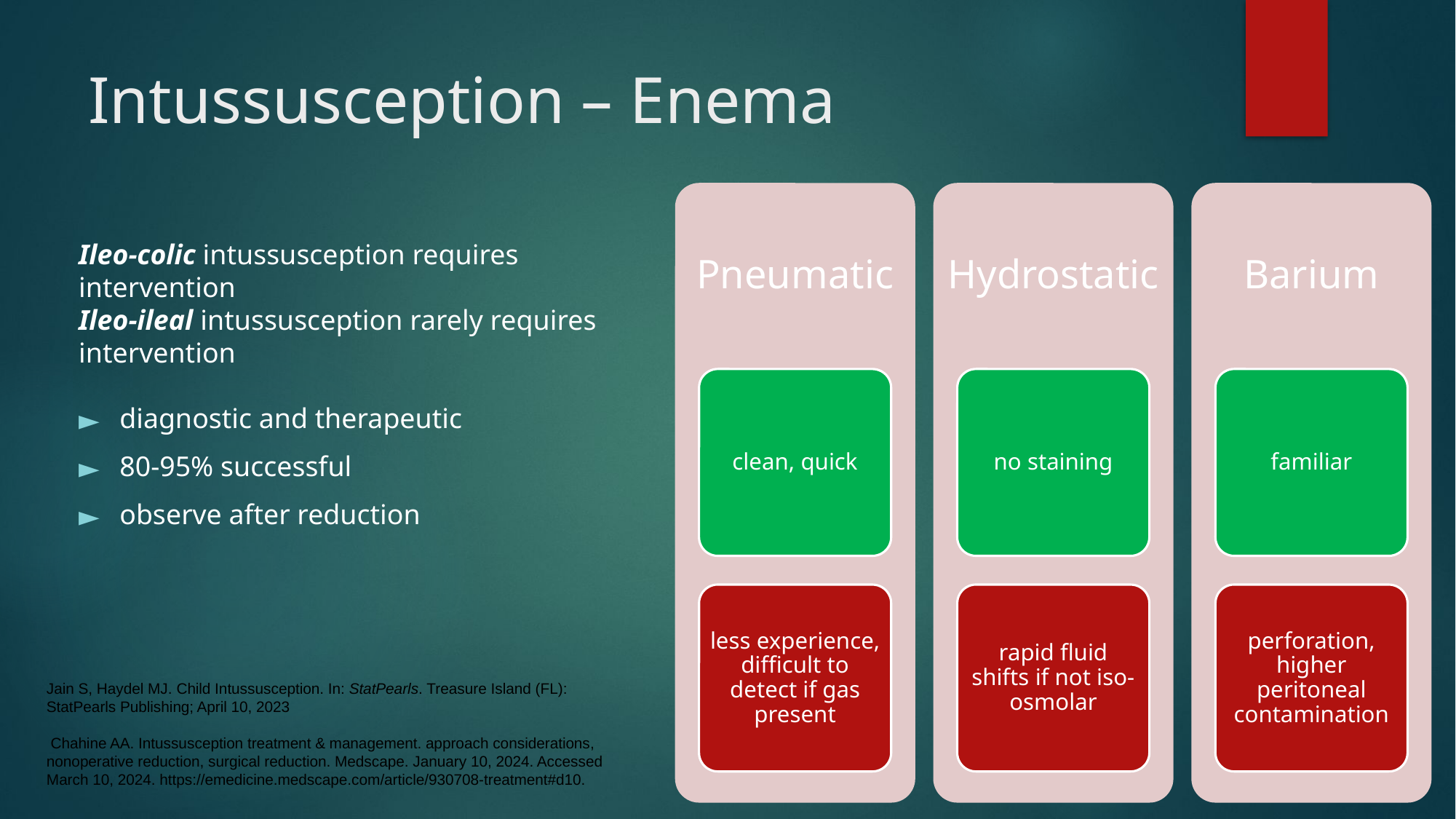

# Intussusception – Enema
Pneumatic
Barium
Hydrostatic
clean, quick
no staining
familiar
less experience, difficult to detect if gas present
rapid fluid shifts if not iso-osmolar
perforation, higher peritoneal contamination
Ileo-colic intussusception requires intervention
Ileo-ileal intussusception rarely requires intervention
diagnostic and therapeutic
80-95% successful
observe after reduction
Jain S, Haydel MJ. Child Intussusception. In: StatPearls. Treasure Island (FL): StatPearls Publishing; April 10, 2023
 Chahine AA. Intussusception treatment & management. approach considerations, nonoperative reduction, surgical reduction. Medscape. January 10, 2024. Accessed March 10, 2024. https://emedicine.medscape.com/article/930708-treatment#d10.

## Slide 38
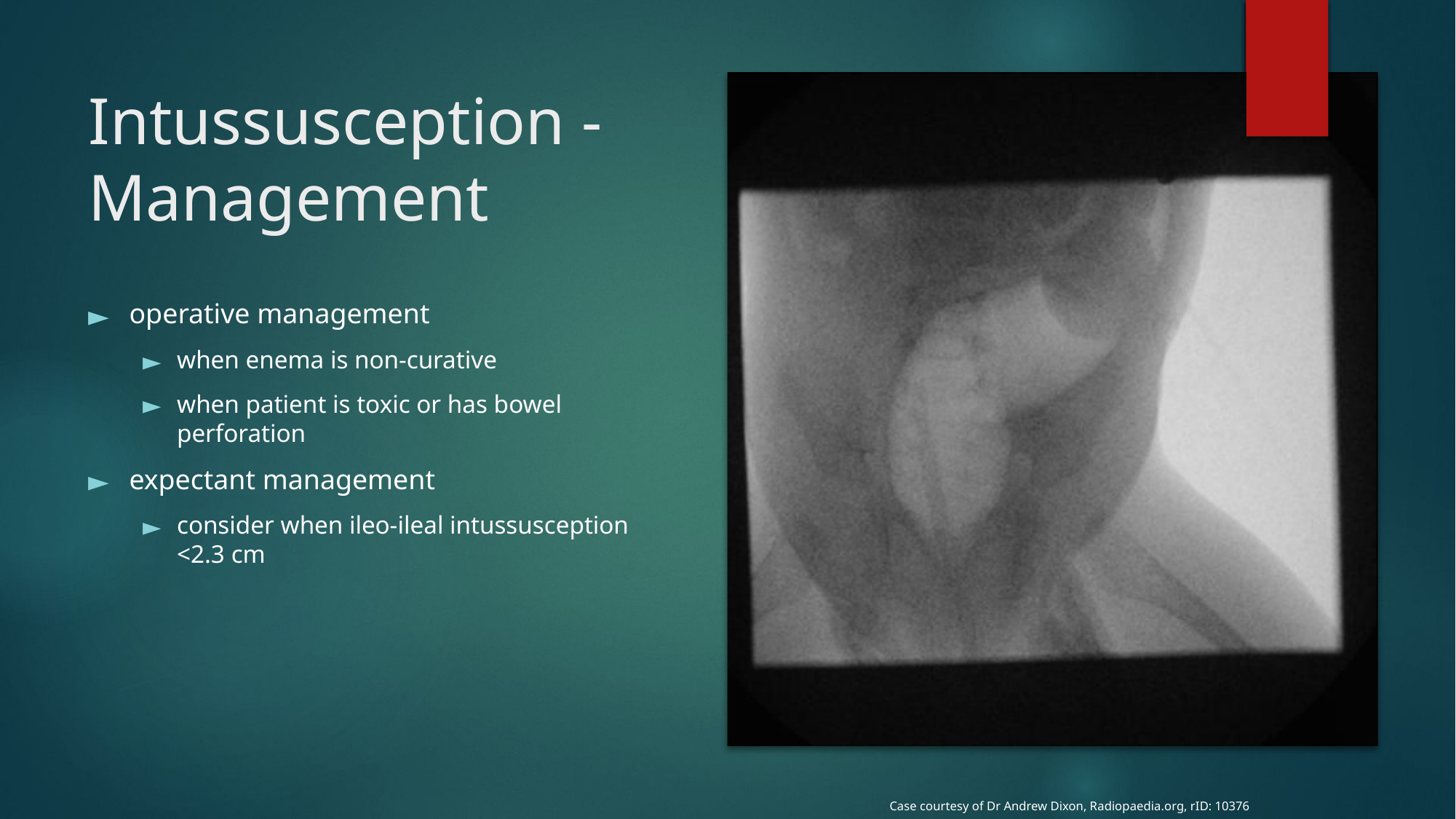

# Intussusception - Management
operative management
when enema is non-curative
when patient is toxic or has bowel perforation
expectant management
consider when ileo-ileal intussusception <2.3 cm
Case courtesy of Dr Andrew Dixon, Radiopaedia.org, rID: 10376

## Slide 39
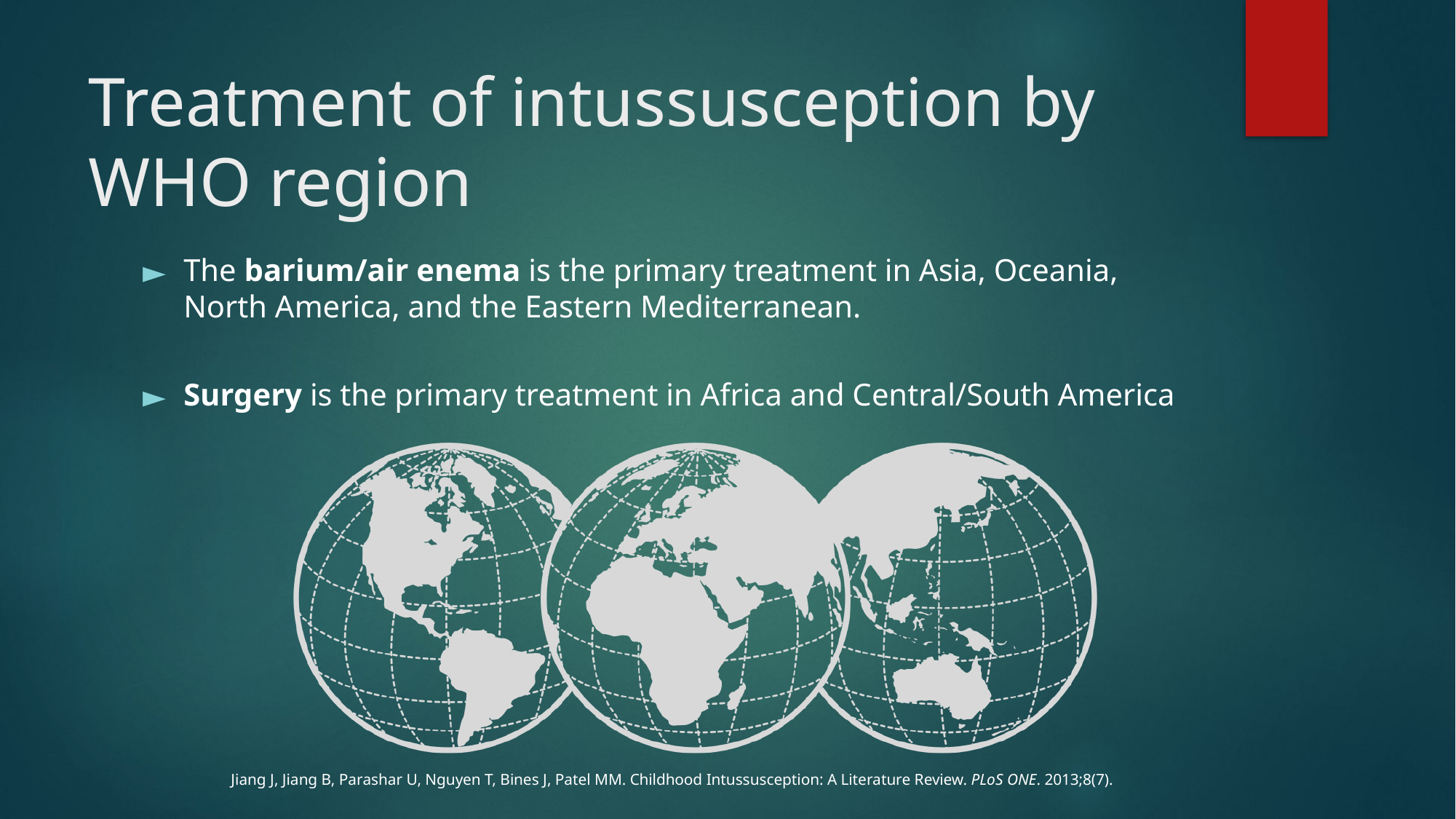

# Treatment of intussusception by WHO region
The barium/air enema is the primary treatment in Asia, Oceania, North America, and the Eastern Mediterranean.
Surgery is the primary treatment in Africa and Central/South America
Jiang J, Jiang B, Parashar U, Nguyen T, Bines J, Patel MM. Childhood Intussusception: A Literature Review. PLoS ONE. 2013;8(7).

## Slide 40
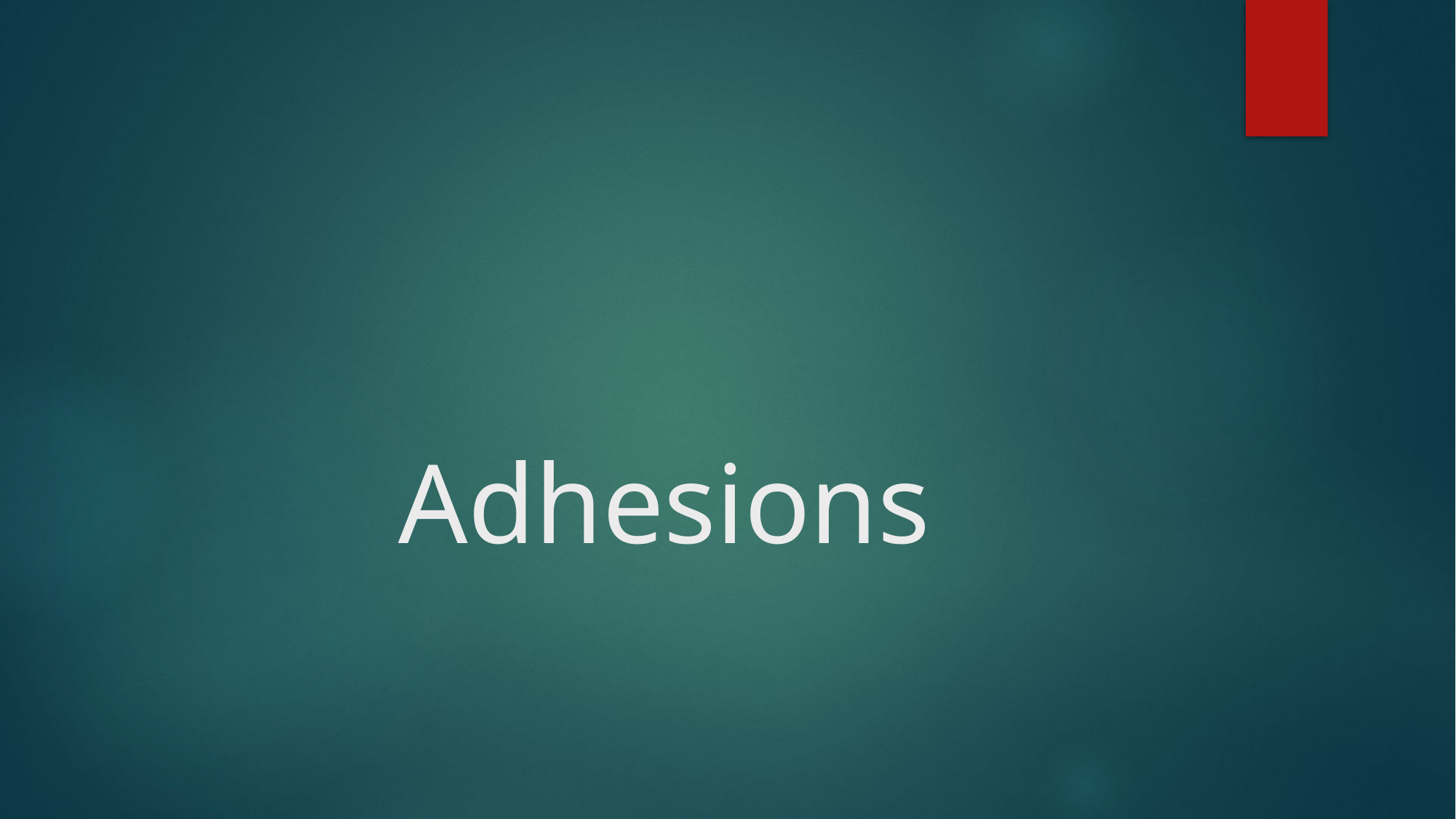

# Adhesions

## Slide 41
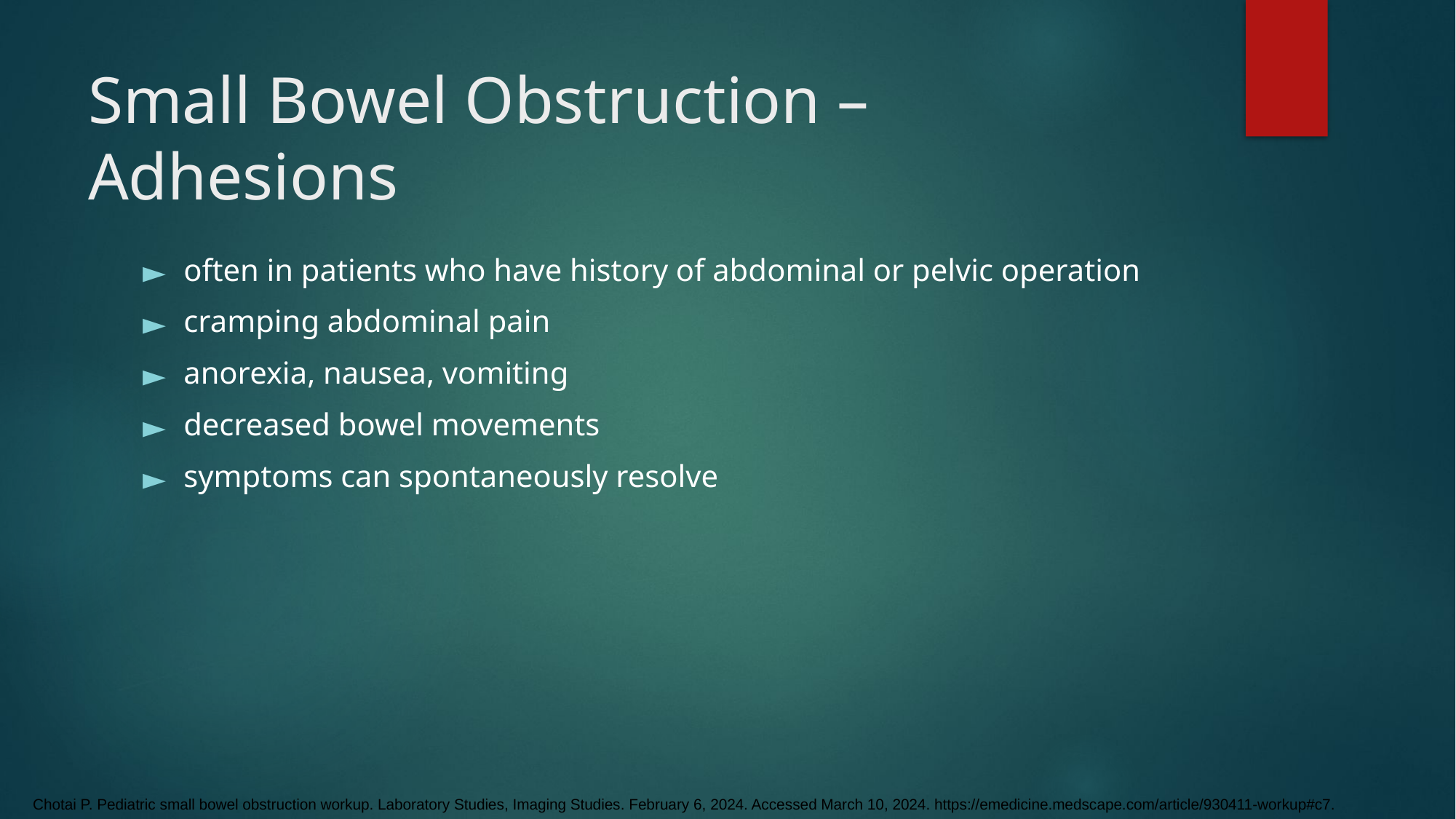

# Small Bowel Obstruction – Adhesions
often in patients who have history of abdominal or pelvic operation
cramping abdominal pain
anorexia, nausea, vomiting
decreased bowel movements
symptoms can spontaneously resolve
Chotai P. Pediatric small bowel obstruction workup. Laboratory Studies, Imaging Studies. February 6, 2024. Accessed March 10, 2024. https://emedicine.medscape.com/article/930411-workup#c7.

## Slide 42
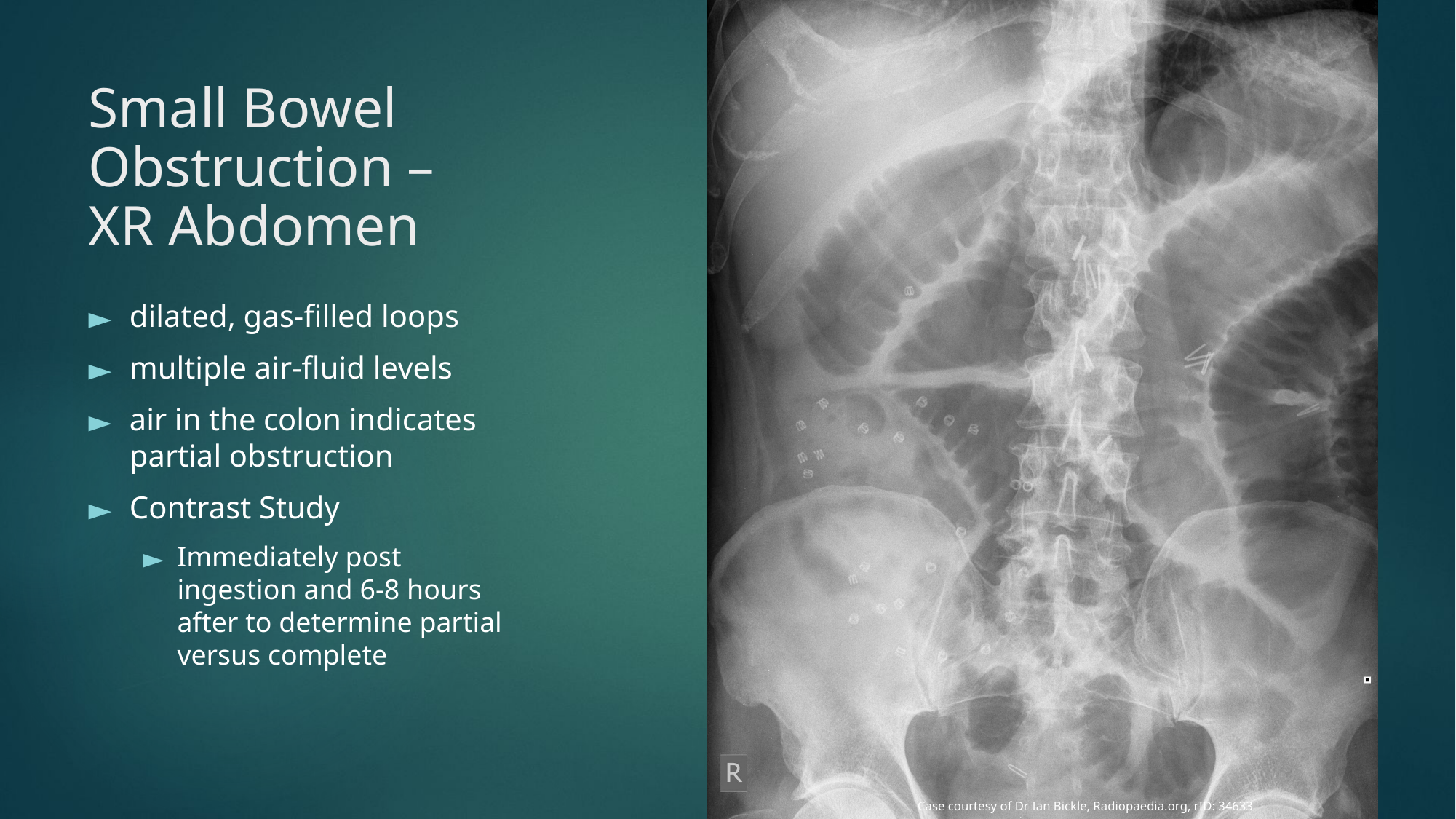

# Small Bowel Obstruction – XR Abdomen
dilated, gas-filled loops
multiple air-fluid levels
air in the colon indicates partial obstruction
Contrast Study
Immediately post ingestion and 6-8 hours after to determine partial versus complete
Case courtesy of Dr Ian Bickle, Radiopaedia.org, rID: 34633

## Slide 43
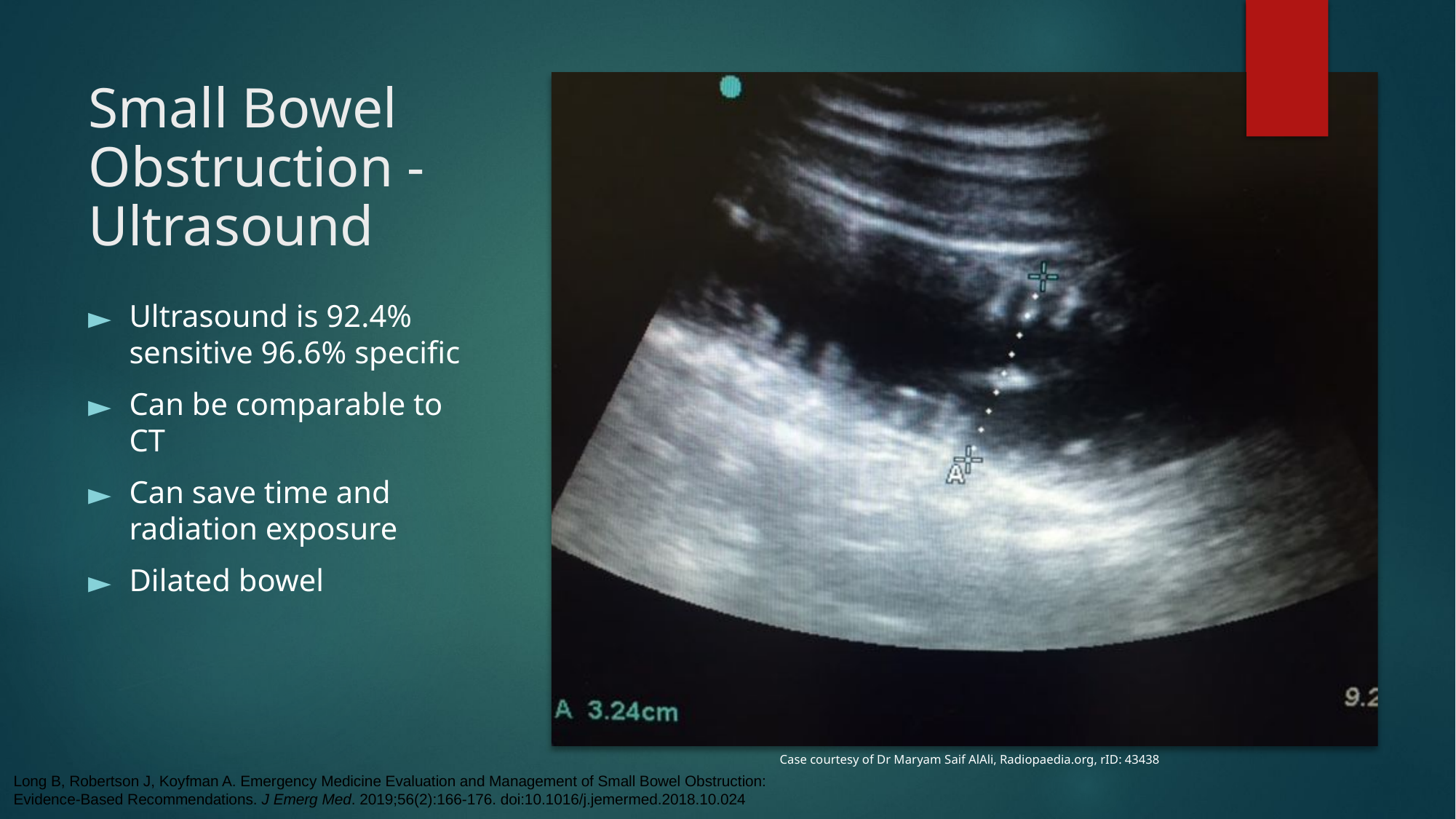

# Small Bowel Obstruction - Ultrasound
Ultrasound is 92.4% sensitive 96.6% specific
Can be comparable to CT
Can save time and radiation exposure
Dilated bowel
Case courtesy of Dr Maryam Saif AlAli, Radiopaedia.org, rID: 43438
Long B, Robertson J, Koyfman A. Emergency Medicine Evaluation and Management of Small Bowel Obstruction: Evidence-Based Recommendations. J Emerg Med. 2019;56(2):166-176. doi:10.1016/j.jemermed.2018.10.024

## Slide 44
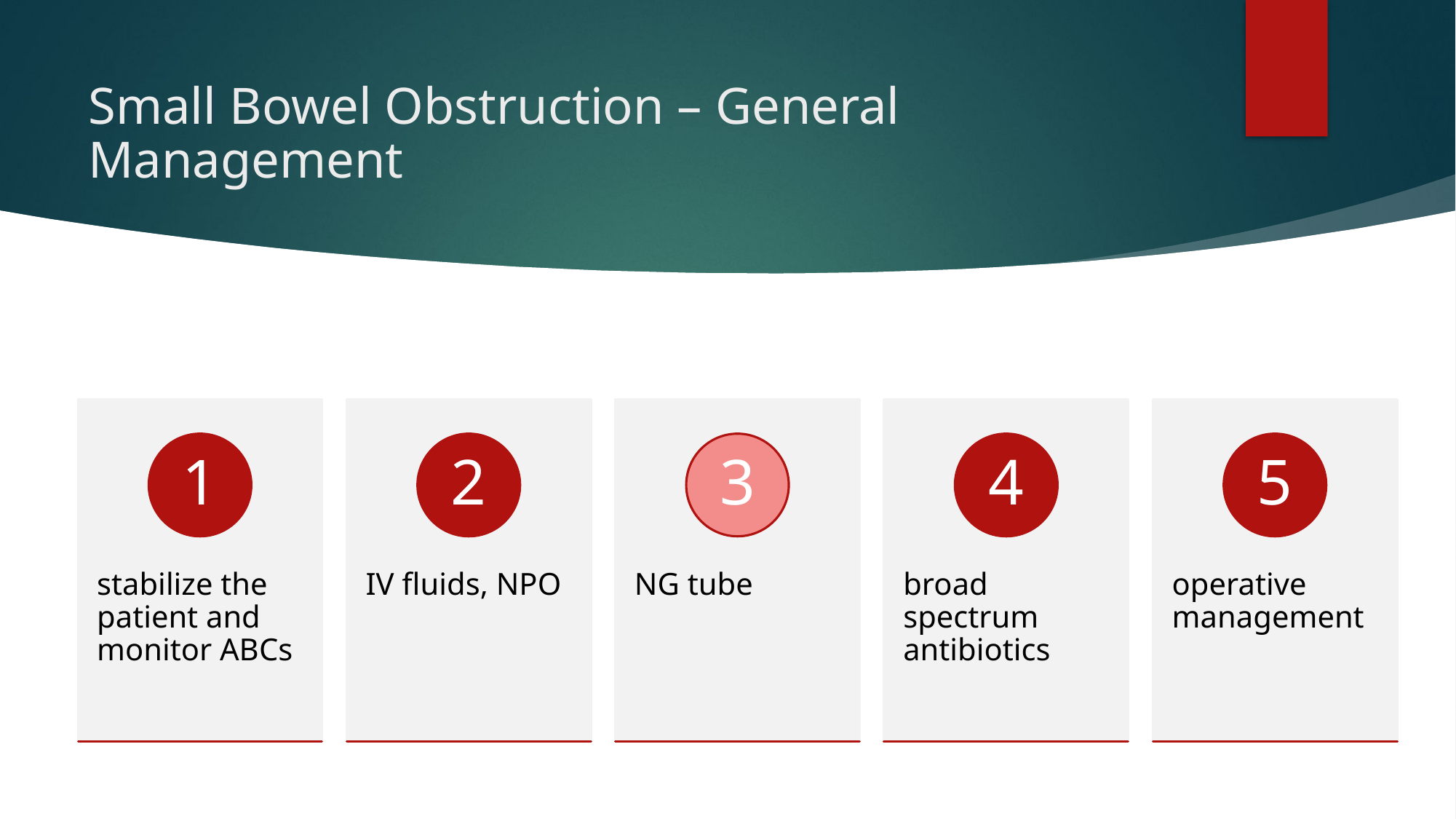

# Small Bowel Obstruction – General Management
1
2
3
4
5
operative management
stabilize the patient and monitor ABCs
IV fluids, NPO
NG tube
broad spectrum antibiotics

## Slide 45
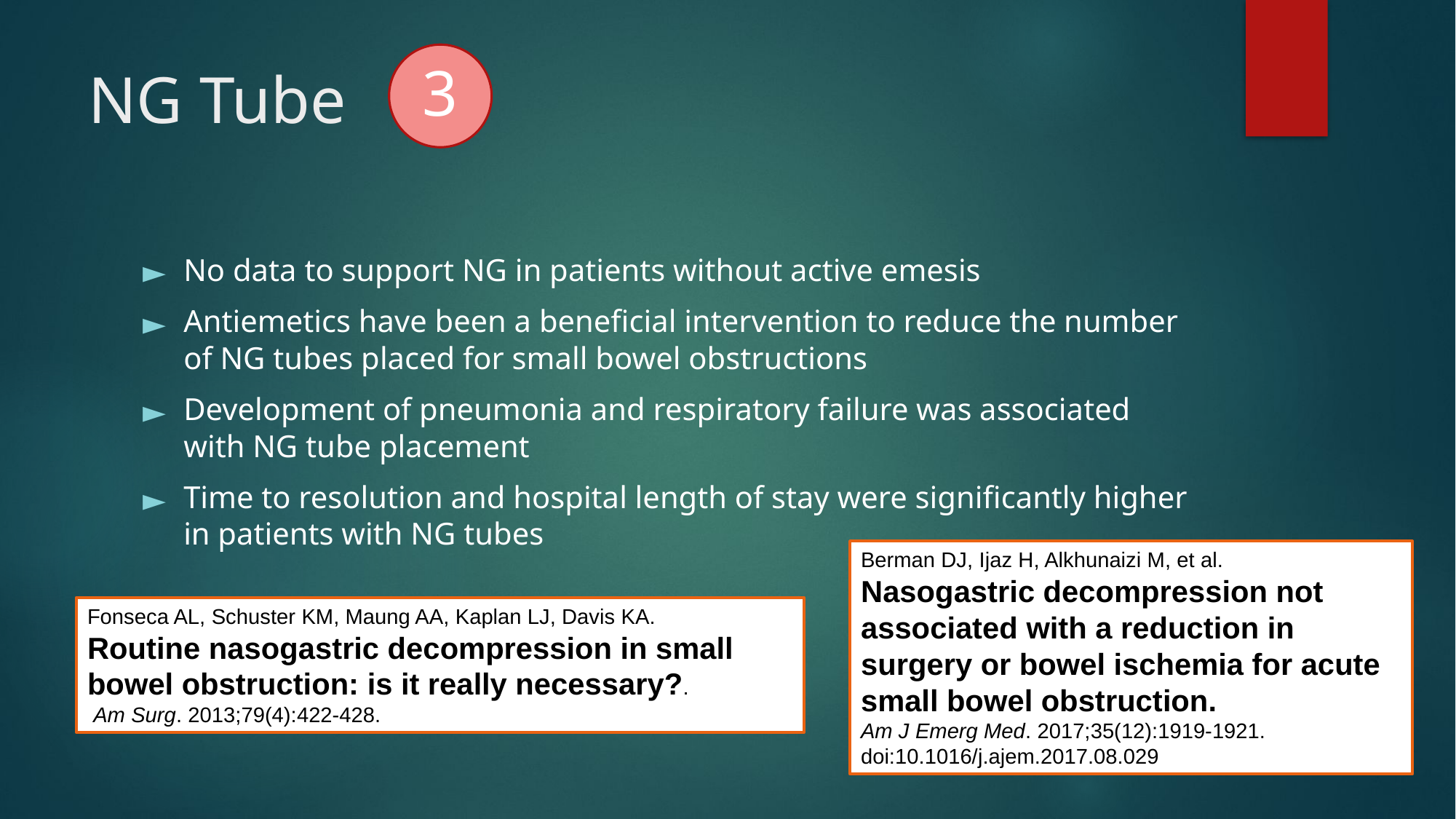

3
# NG Tube
No data to support NG in patients without active emesis
Antiemetics have been a beneficial intervention to reduce the number of NG tubes placed for small bowel obstructions
Development of pneumonia and respiratory failure was associated with NG tube placement
Time to resolution and hospital length of stay were significantly higher in patients with NG tubes
Berman DJ, Ijaz H, Alkhunaizi M, et al.
Nasogastric decompression not associated with a reduction in surgery or bowel ischemia for acute small bowel obstruction.
Am J Emerg Med. 2017;35(12):1919-1921. doi:10.1016/j.ajem.2017.08.029
Fonseca AL, Schuster KM, Maung AA, Kaplan LJ, Davis KA.
Routine nasogastric decompression in small bowel obstruction: is it really necessary?.
 Am Surg. 2013;79(4):422-428.

## Slide 46
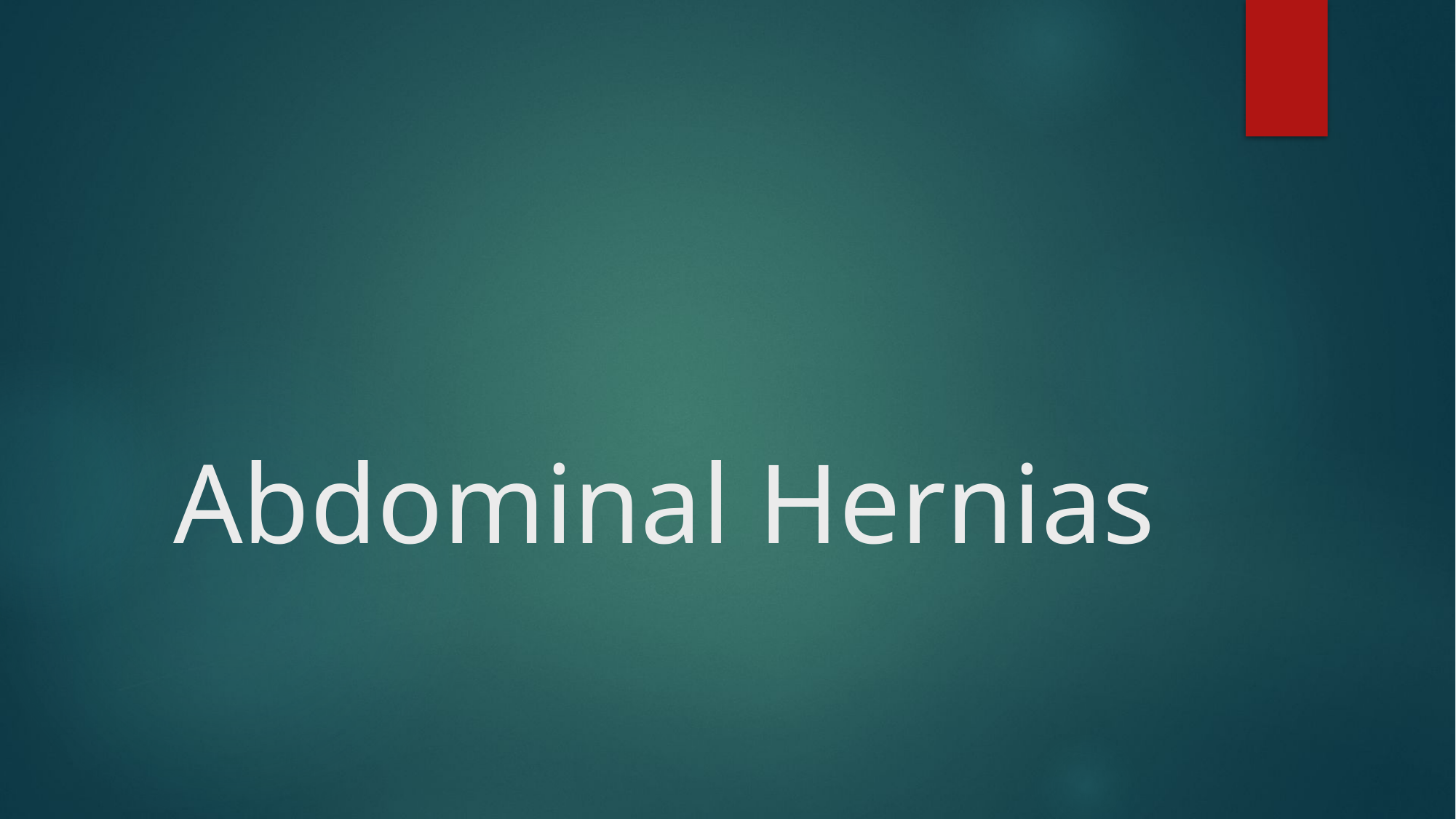

# Abdominal Hernias

## Slide 47
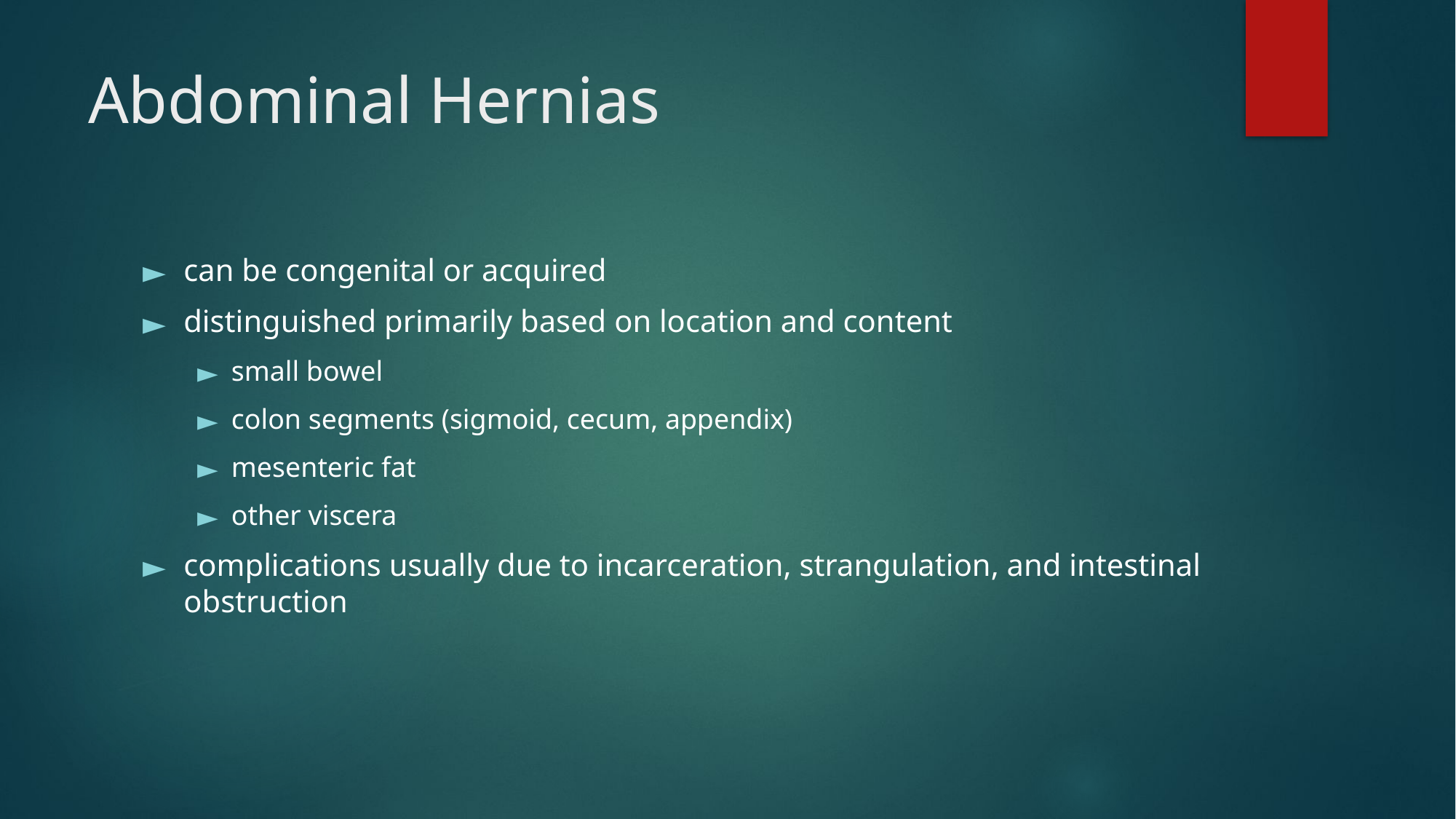

# Abdominal Hernias
can be congenital or acquired
distinguished primarily based on location and content
small bowel
colon segments (sigmoid, cecum, appendix)
mesenteric fat
other viscera
complications usually due to incarceration, strangulation, and intestinal obstruction

## Slide 48
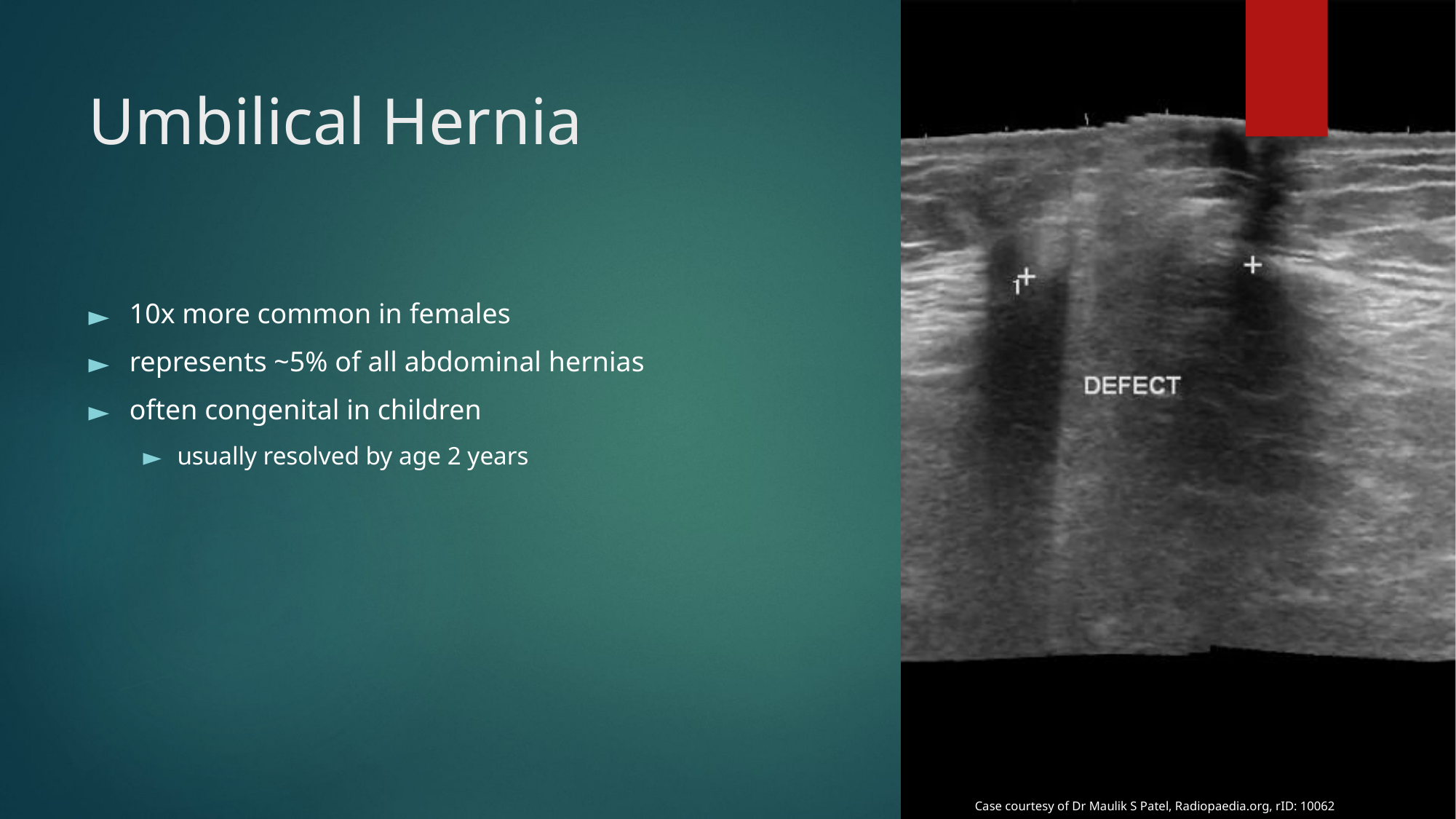

# Umbilical Hernia
10x more common in females
represents ~5% of all abdominal hernias
often congenital in children
usually resolved by age 2 years
Case courtesy of Dr Maulik S Patel, Radiopaedia.org, rID: 10062

## Slide 49
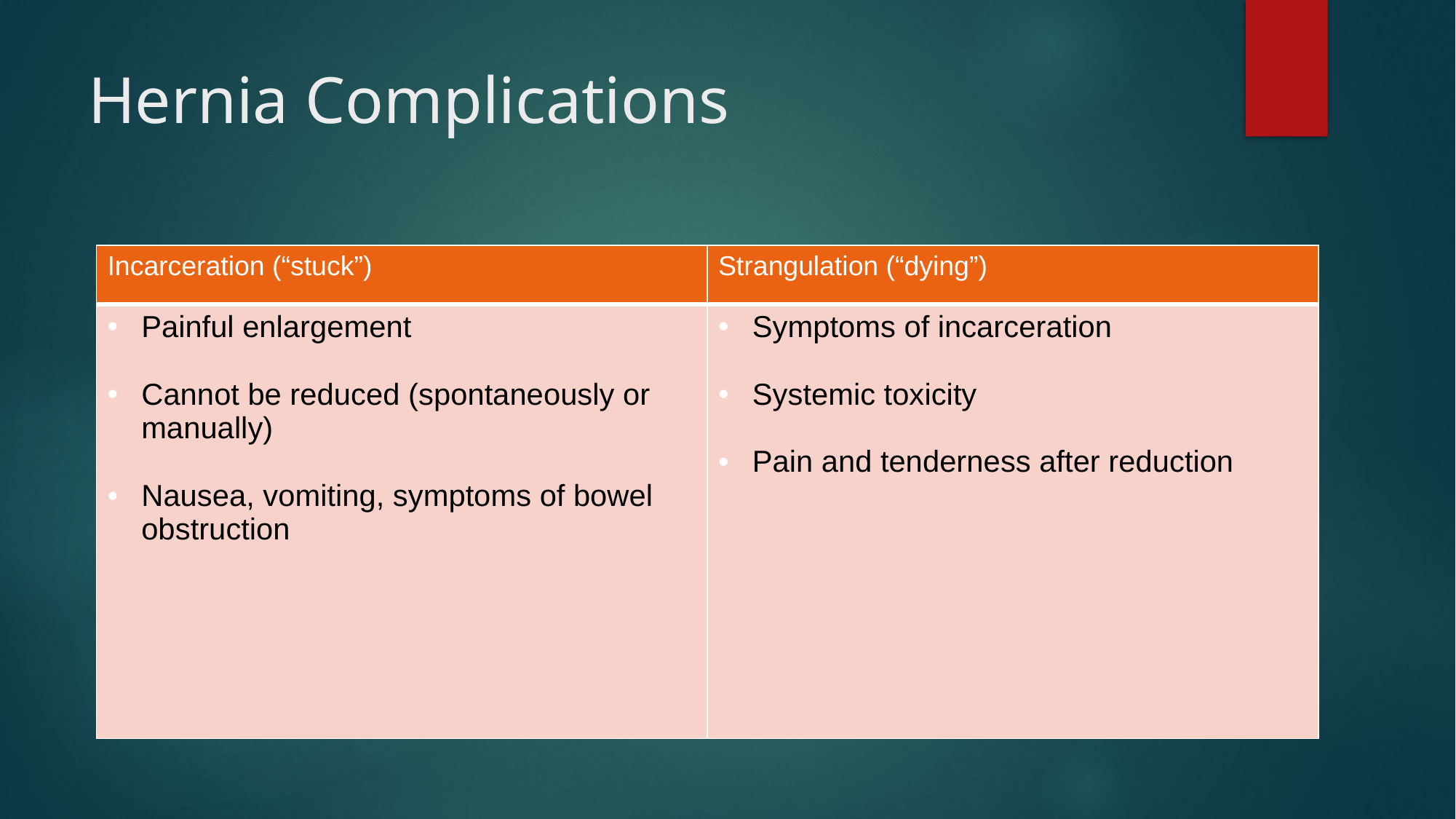

# Hernia Complications
| Incarceration (“stuck”) | Strangulation (“dying”) |
| --- | --- |
| Painful enlargement Cannot be reduced (spontaneously or manually) Nausea, vomiting, symptoms of bowel obstruction | Symptoms of incarceration Systemic toxicity Pain and tenderness after reduction |

## Slide 50
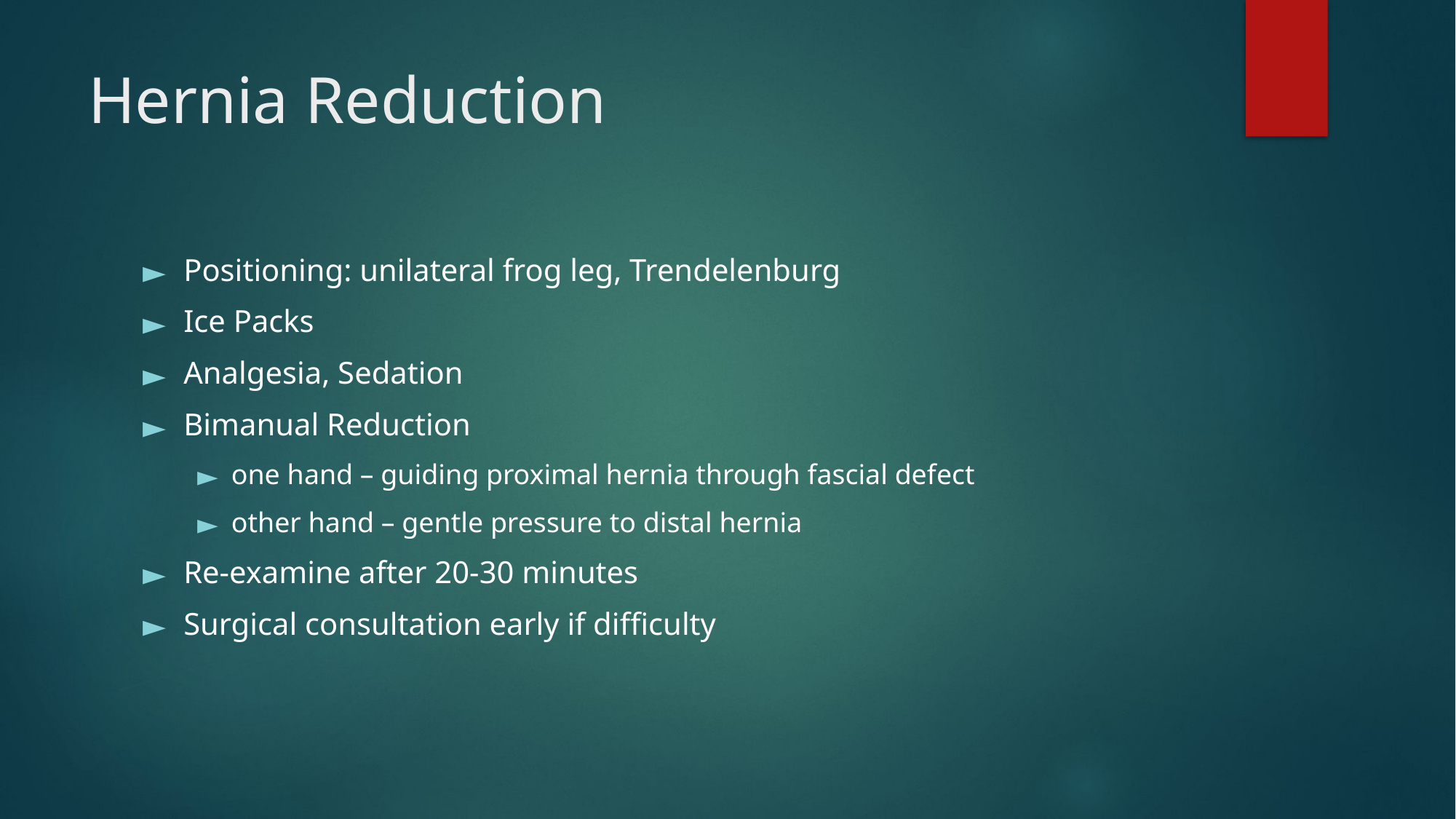

# Hernia Reduction
Positioning: unilateral frog leg, Trendelenburg
Ice Packs
Analgesia, Sedation
Bimanual Reduction
one hand – guiding proximal hernia through fascial defect
other hand – gentle pressure to distal hernia
Re-examine after 20-30 minutes
Surgical consultation early if difficulty

## Slide 51
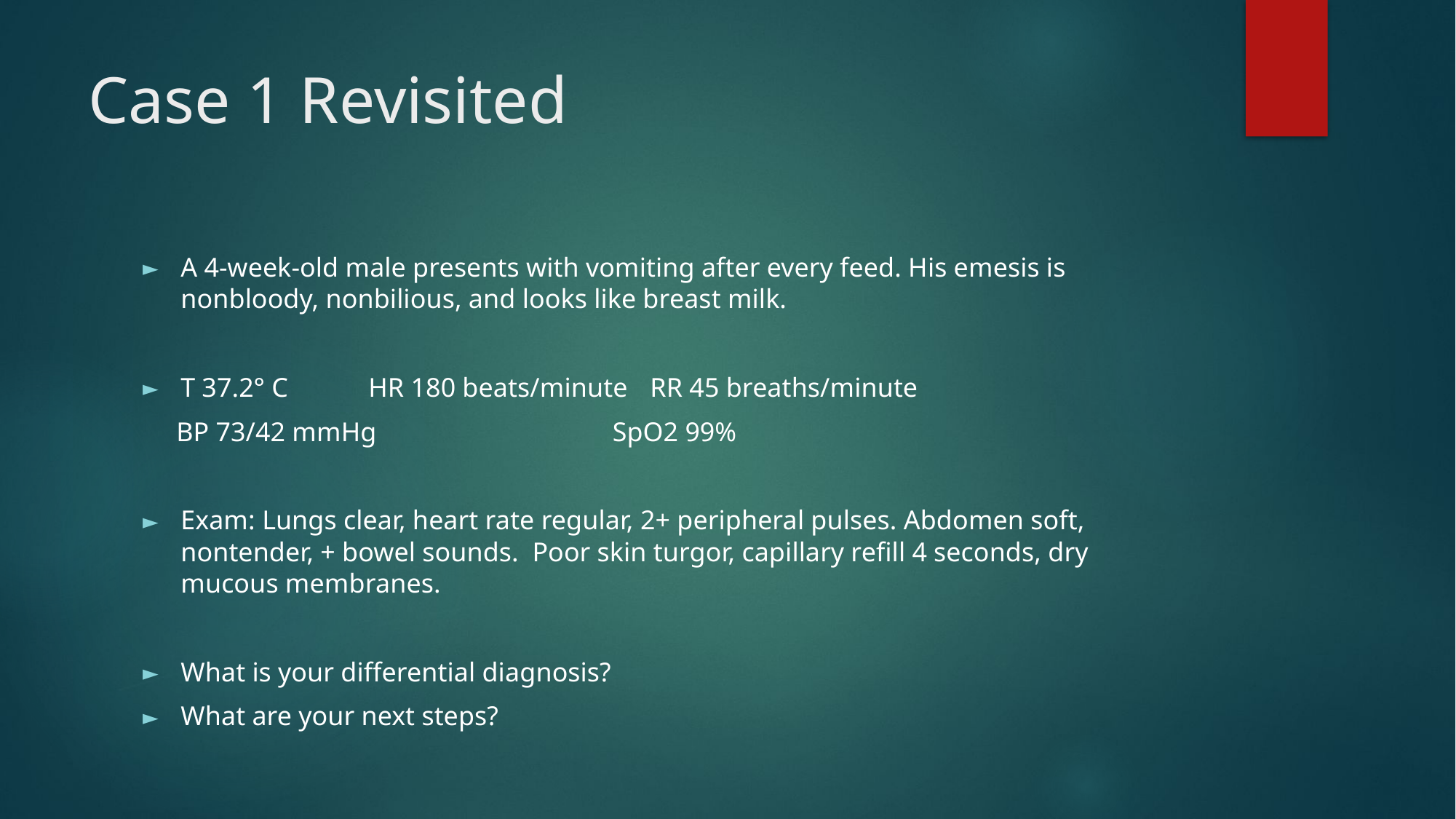

# Case 1 Revisited
A 4-week-old male presents with vomiting after every feed. His emesis is nonbloody, nonbilious, and looks like breast milk.
T 37.2° C	HR 180 beats/minute 		RR 45 breaths/minute
 BP 73/42 mmHg 		 SpO2 99%
Exam: Lungs clear, heart rate regular, 2+ peripheral pulses. Abdomen soft, nontender, + bowel sounds. Poor skin turgor, capillary refill 4 seconds, dry mucous membranes.
What is your differential diagnosis?
What are your next steps?

## Slide 52
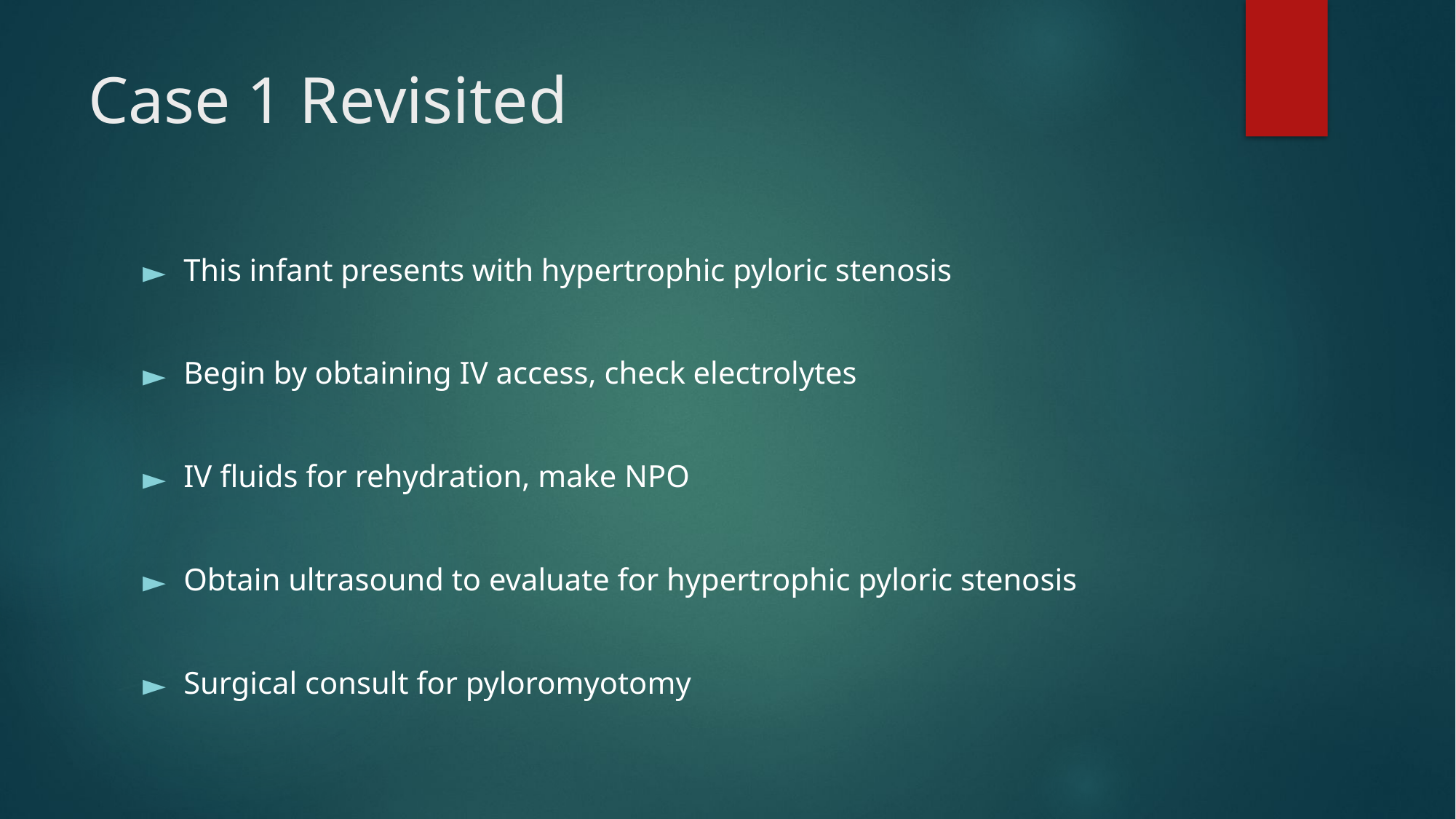

# Case 1 Revisited
This infant presents with hypertrophic pyloric stenosis
Begin by obtaining IV access, check electrolytes
IV fluids for rehydration, make NPO
Obtain ultrasound to evaluate for hypertrophic pyloric stenosis
Surgical consult for pyloromyotomy

## Slide 53
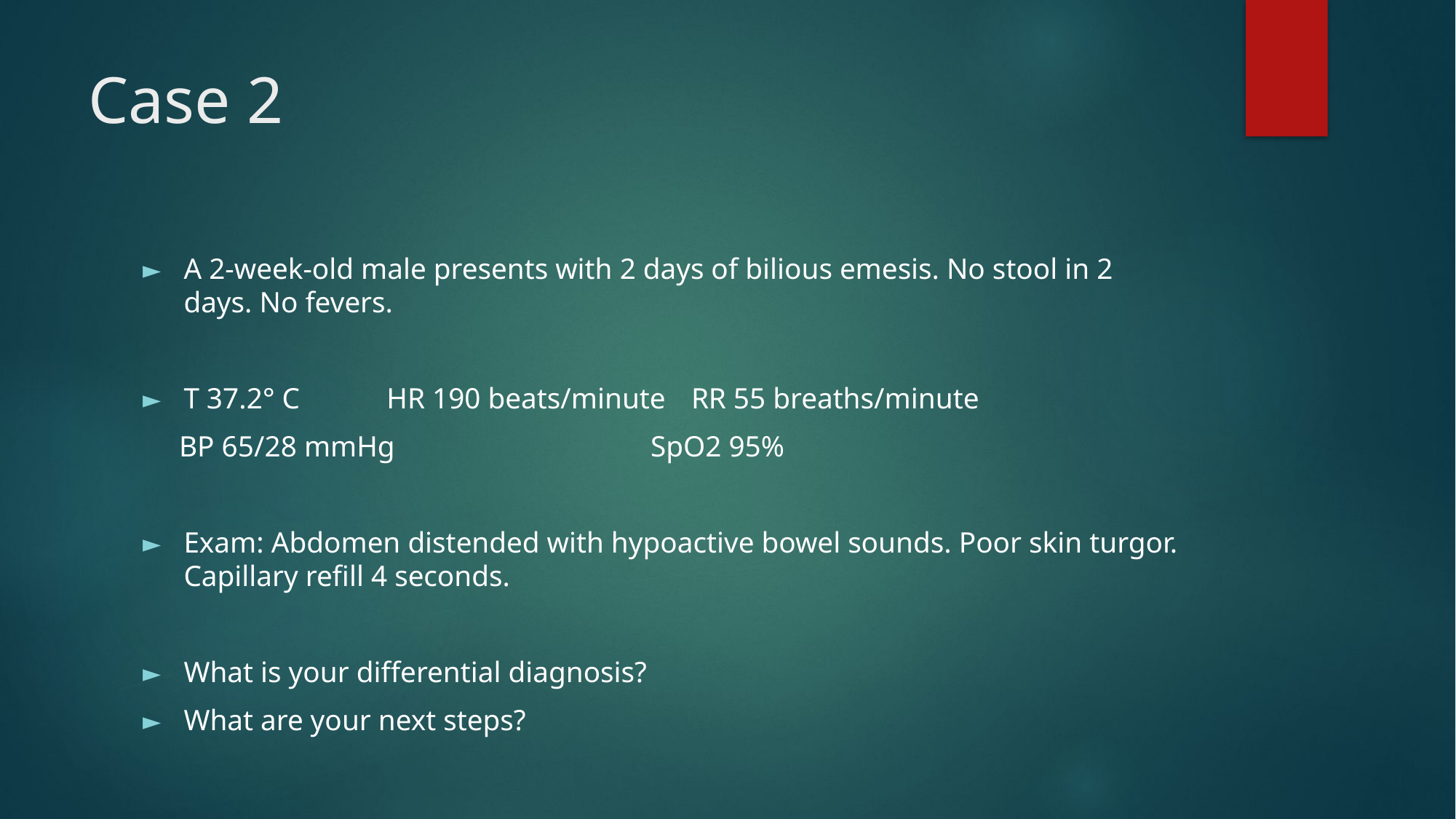

# Case 2
A 2-week-old male presents with 2 days of bilious emesis. No stool in 2 days. No fevers.
T 37.2° C	HR 190 beats/minute 		RR 55 breaths/minute
 BP 65/28 mmHg 		 SpO2 95%
Exam: Abdomen distended with hypoactive bowel sounds. Poor skin turgor. Capillary refill 4 seconds.
What is your differential diagnosis?
What are your next steps?

## Slide 54
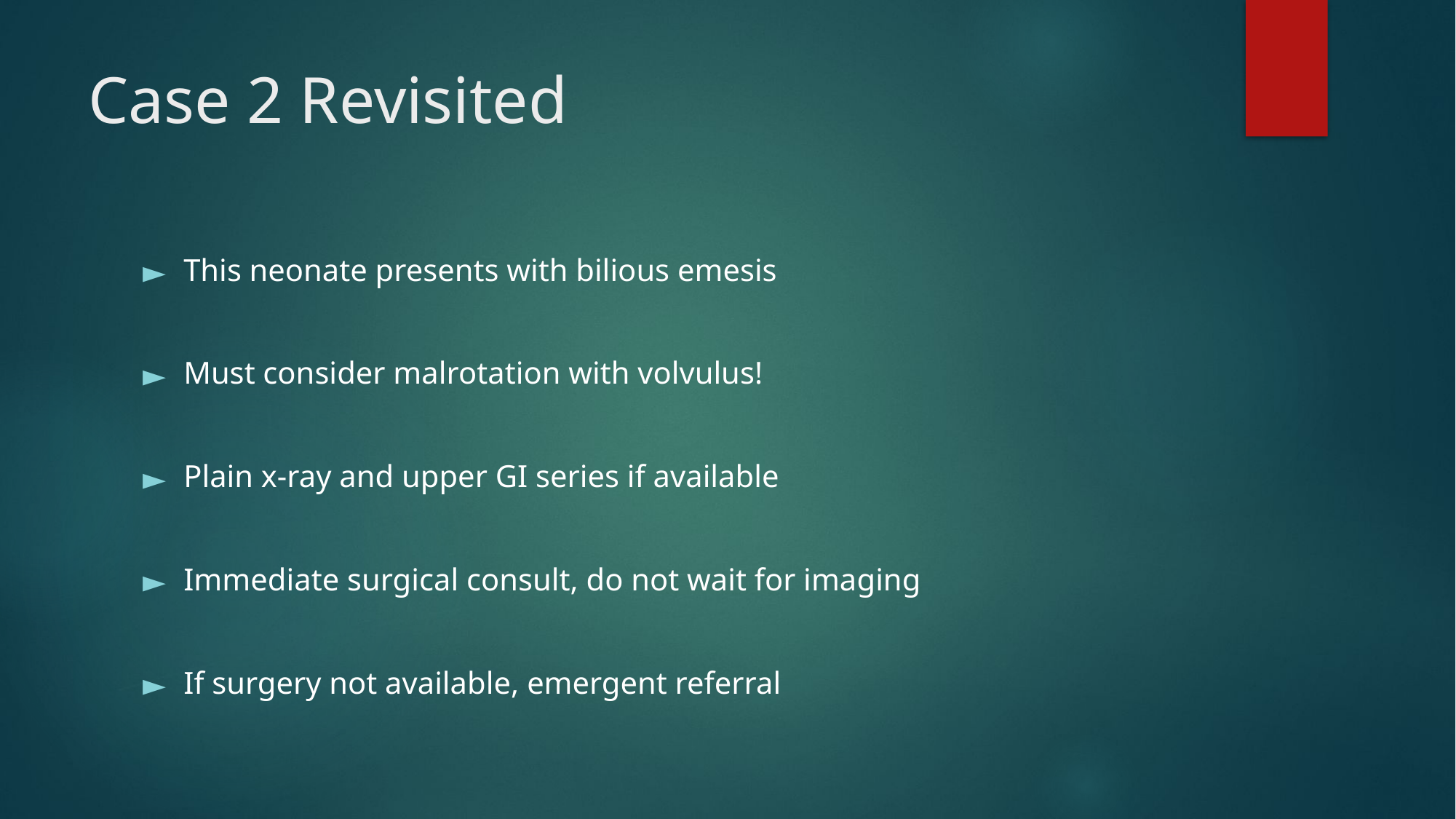

# Case 2 Revisited
This neonate presents with bilious emesis
Must consider malrotation with volvulus!
Plain x-ray and upper GI series if available
Immediate surgical consult, do not wait for imaging
If surgery not available, emergent referral
