# Supplementary material for: A Simulation and Small-Group Pediatric Emergency Medicine Course for Generalist Healthcare Providers: Gastrointestinal and Nutrition Emergencies
Source: J Educ Teach Emerg Med. 2024 Oct 31;9(4):C1–C120. doi: 10.21980/J8WH2K (PMC11537732; doi:10.21980/J8WH2K)
Supplement: Supplementary file 3 — Please see associated PowerPoint file [file 9-4-C1-Appendix_L.pptx]

## Slide 1
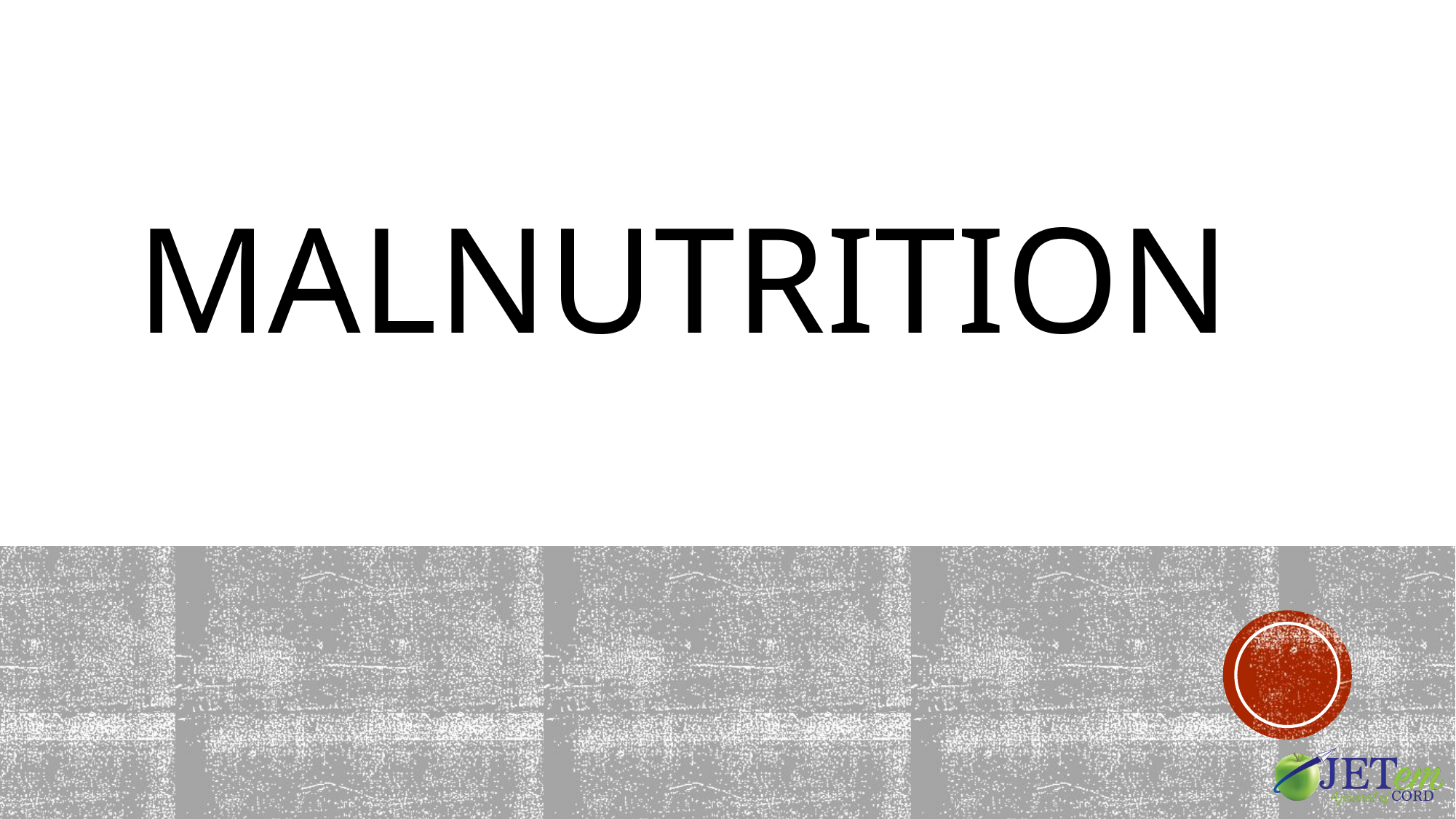

# MALNUTRITION

## Slide 2
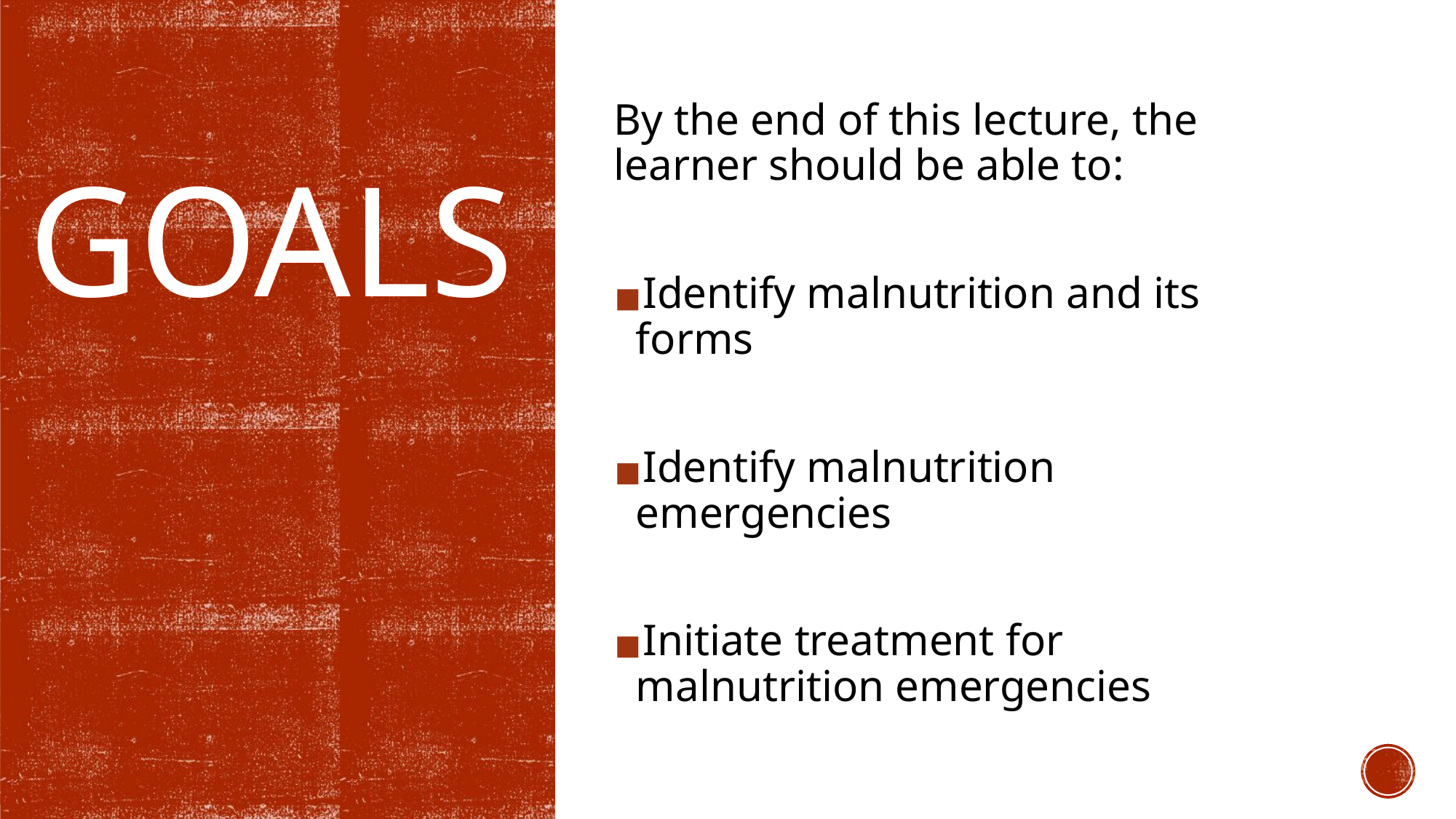

# GOALS
By the end of this lecture, the learner should be able to:
Identify malnutrition and its forms
Identify malnutrition emergencies
Initiate treatment for malnutrition emergencies

## Slide 3
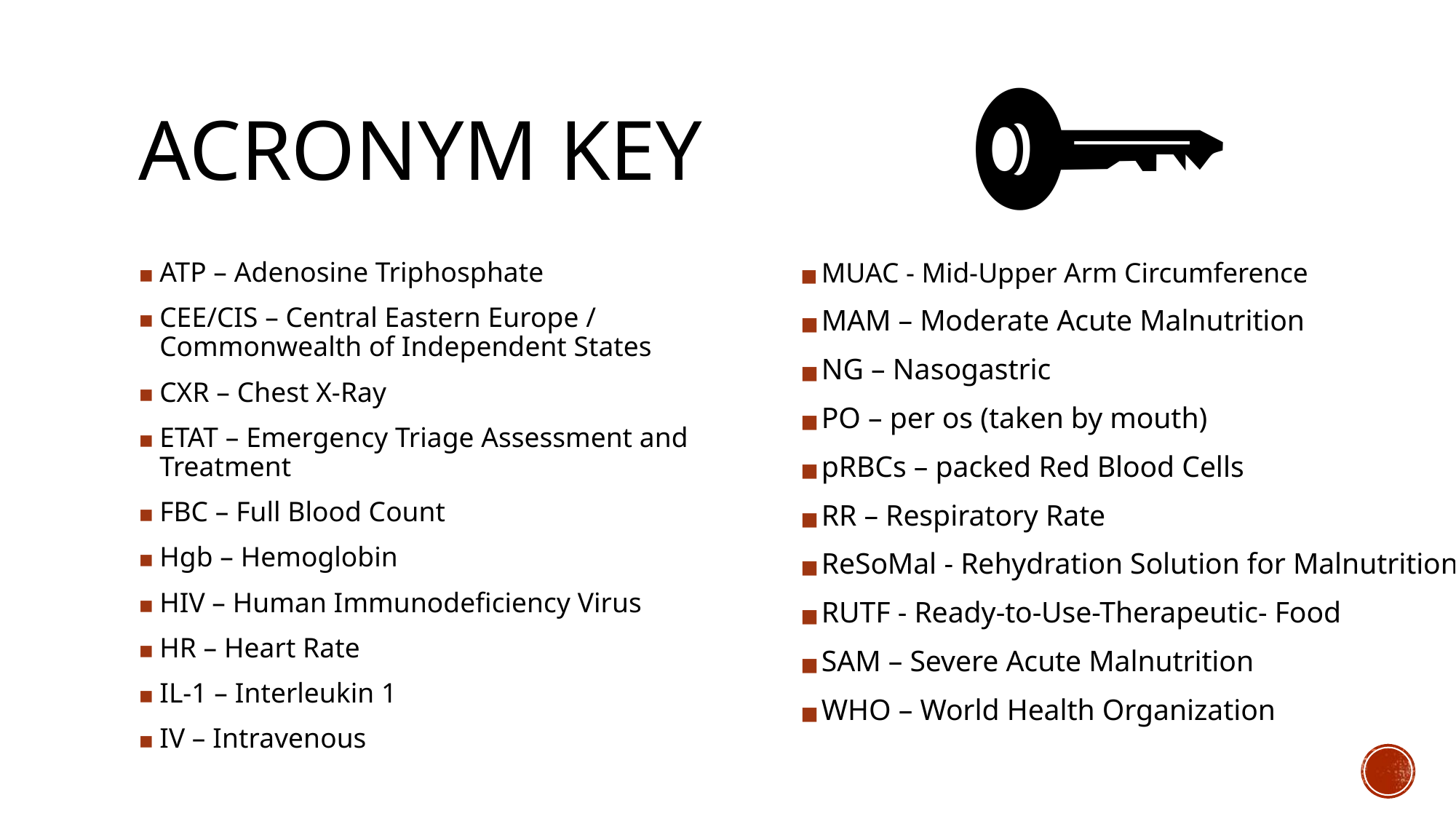

# ACRONYM KEY
ATP – Adenosine Triphosphate
CEE/CIS – Central Eastern Europe / Commonwealth of Independent States
CXR – Chest X-Ray
ETAT – Emergency Triage Assessment and Treatment
FBC – Full Blood Count
Hgb – Hemoglobin
HIV – Human Immunodeficiency Virus
HR – Heart Rate
IL-1 – Interleukin 1
IV – Intravenous
MUAC - Mid-Upper Arm Circumference
MAM – Moderate Acute Malnutrition
NG – Nasogastric
PO – per os (taken by mouth)
pRBCs – packed Red Blood Cells
RR – Respiratory Rate
ReSoMal - Rehydration Solution for Malnutrition
RUTF - Ready-to-Use-Therapeutic- Food
SAM – Severe Acute Malnutrition
WHO – World Health Organization

## Slide 4
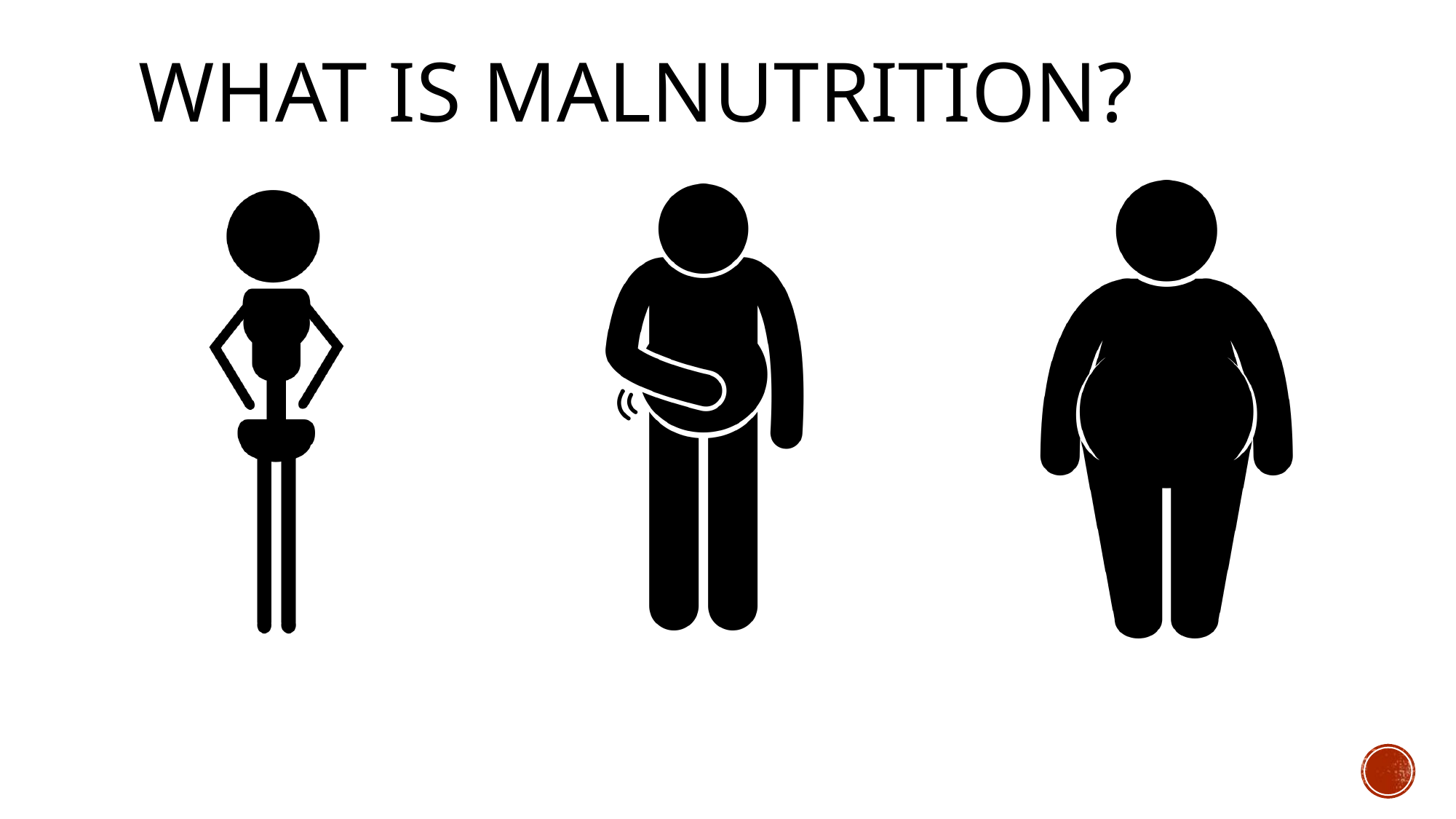

# WHAT IS MALNUTRITION?

## Slide 5
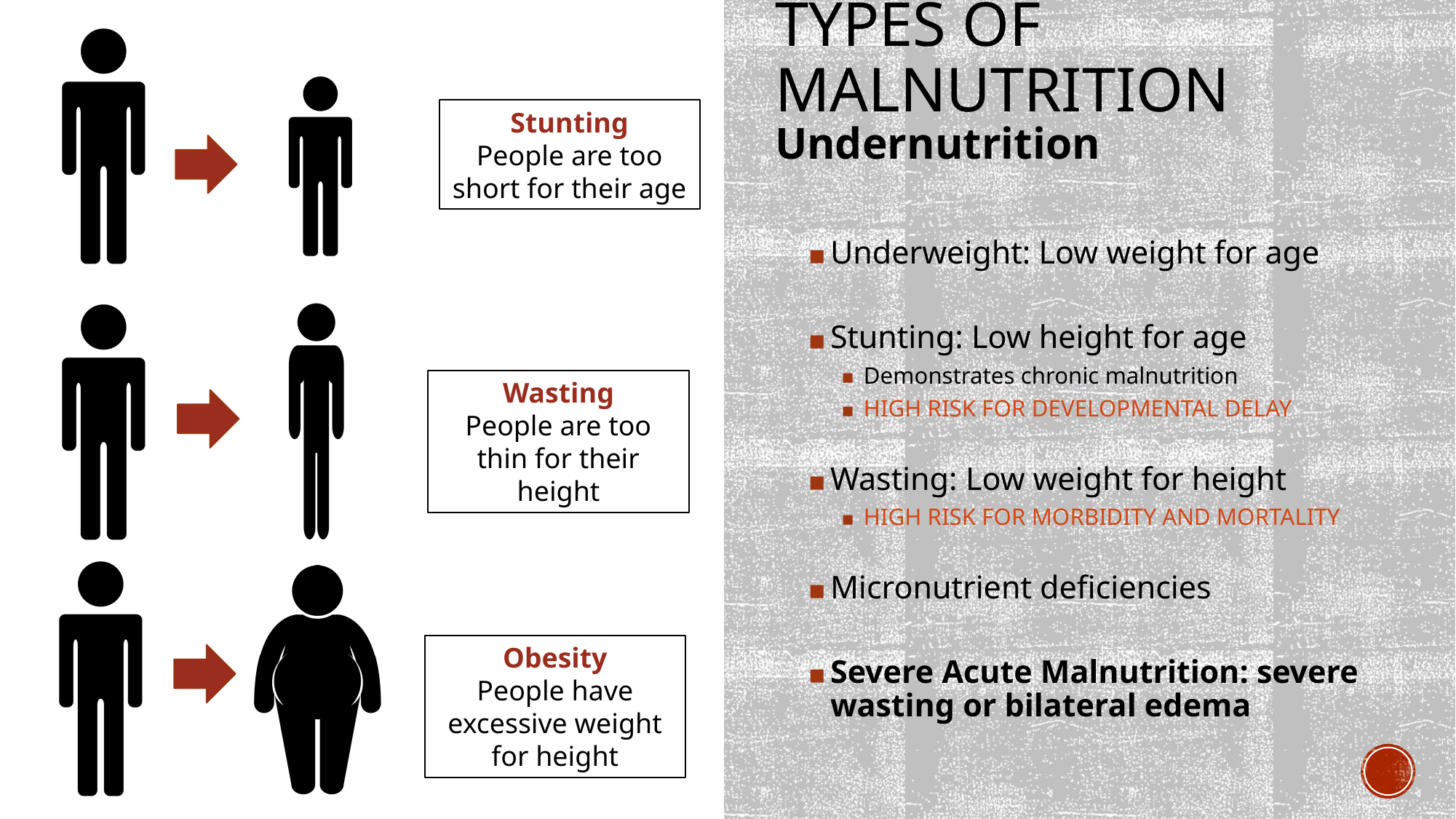

# TYPES OF MALNUTRITION
Stunting
People are too short for their age
Undernutrition
Underweight: Low weight for age
Stunting: Low height for age
Demonstrates chronic malnutrition
HIGH RISK FOR DEVELOPMENTAL DELAY
Wasting: Low weight for height
HIGH RISK FOR MORBIDITY AND MORTALITY
Micronutrient deficiencies
Severe Acute Malnutrition: severe wasting or bilateral edema
Wasting
People are too thin for their height
Obesity
People have excessive weight for height

## Slide 6
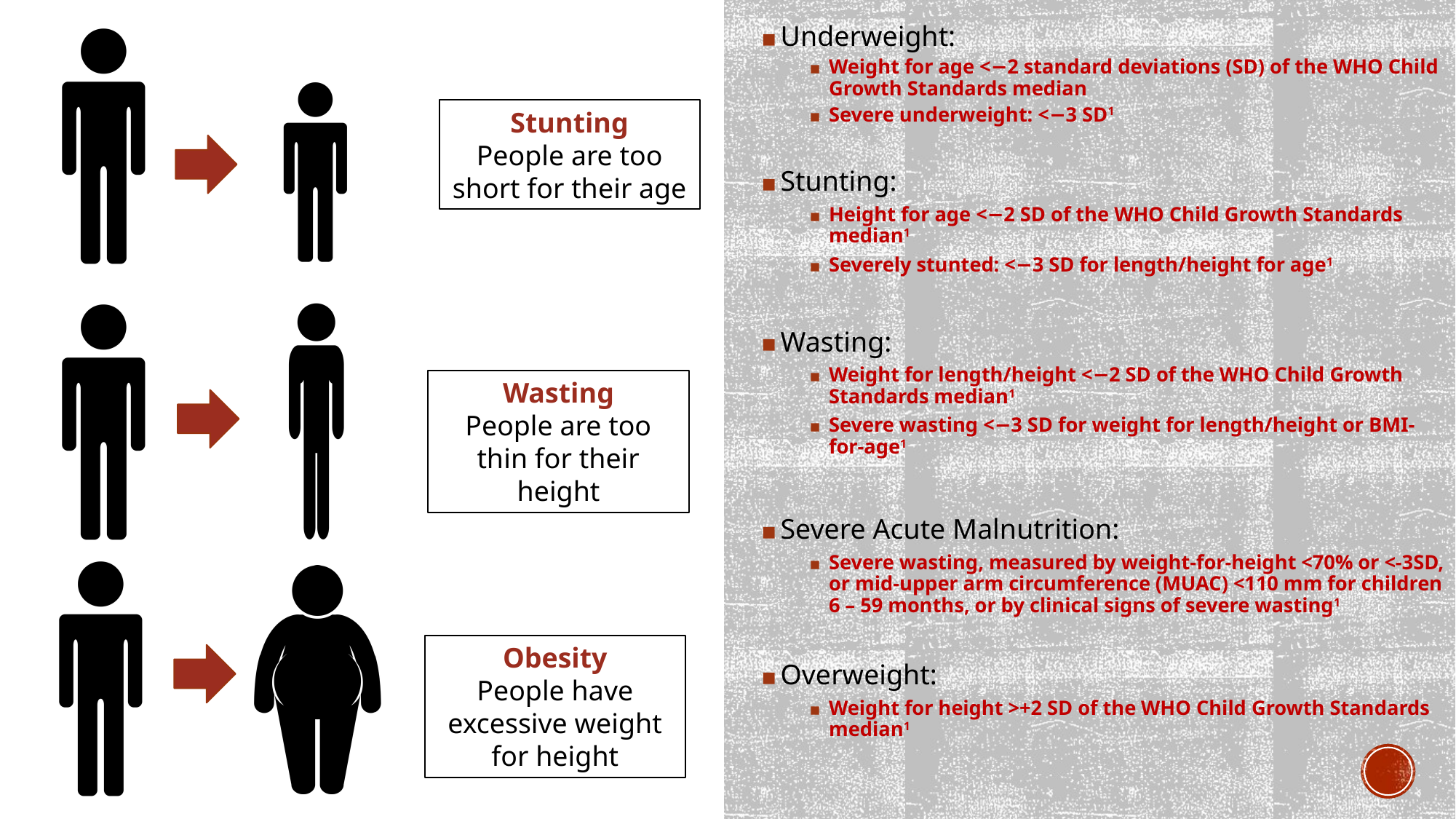

Underweight:
Weight for age <−2 standard deviations (SD) of the WHO Child Growth Standards median
Severe underweight: <−3 SD1
Stunting:
Height for age <−2 SD of the WHO Child Growth Standards median1
Severely stunted: <−3 SD for length/height for age1
Wasting:
Weight for length/height <−2 SD of the WHO Child Growth Standards median1
Severe wasting <−3 SD for weight for length/height or BMI-for-age1
Severe Acute Malnutrition:
Severe wasting, measured by weight-for-height <70% or <-3SD, or mid-upper arm circumference (MUAC) <110 mm for children 6 – 59 months, or by clinical signs of severe wasting1
Overweight:
Weight for height >+2 SD of the WHO Child Growth Standards median1
Stunting
People are too short for their age
Wasting
People are too thin for their height
Obesity
People have excessive weight for height

## Slide 7
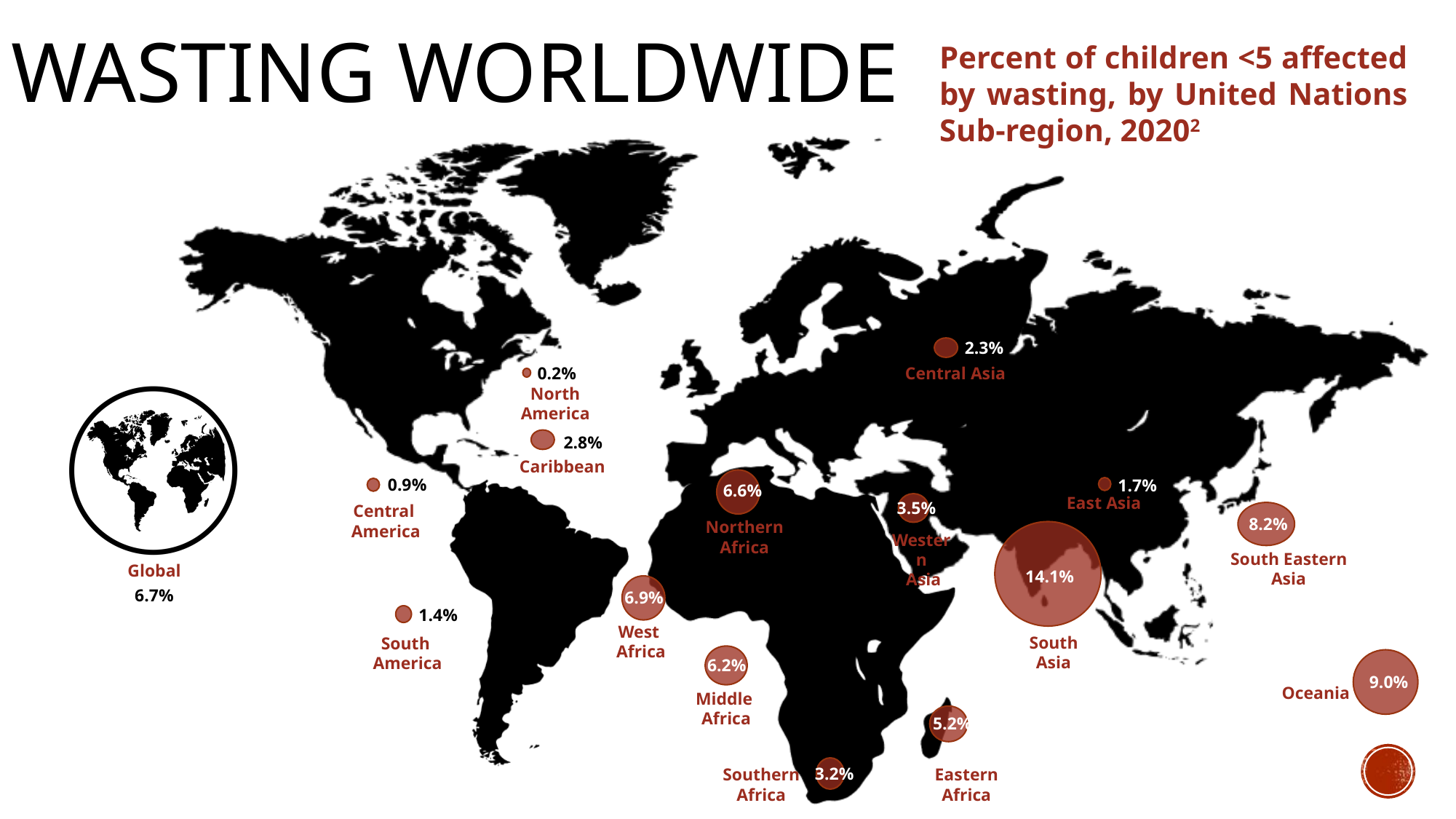

# WASTING WORLDWIDE
Percent of children <5 affected by wasting, by United Nations Sub-region, 20202
2.3%
Central Asia
0.2%
North America
3.4%
2.8%
Caribbean
0.9%
1.7%
6.6%
East Asia
3.5%
Central
America
8.2%
Northern
Africa
Western
 Asia
South Eastern
Asia
Global
14.1%
8.4%
6.7%
6.9%
1.4%
West
Africa
South
Asia
South
America
6.2%
9.0%
Oceania
Middle
Africa
5.2%
3.2%
Southern
Africa
Eastern
Africa

## Slide 8
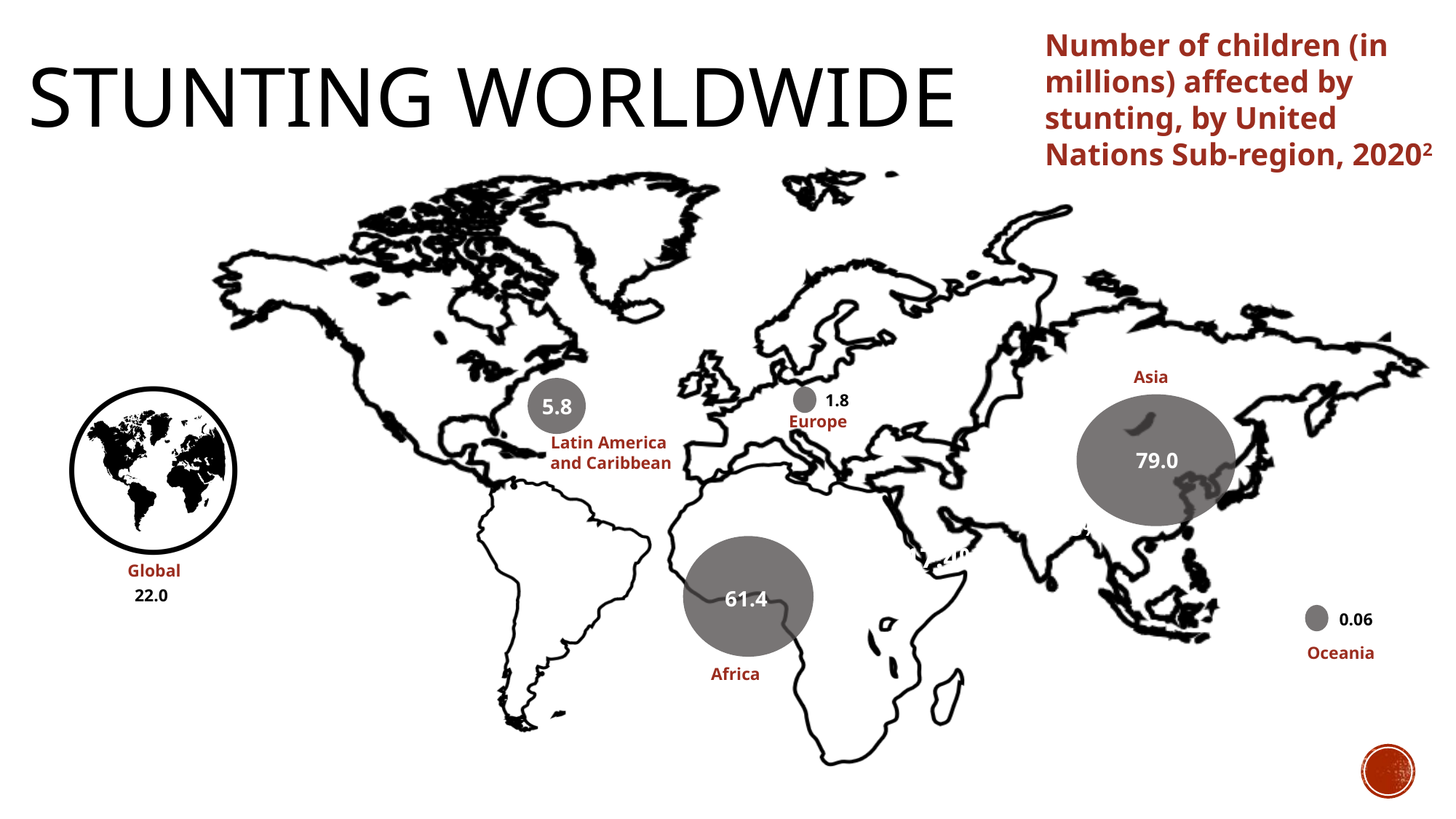

# STUNTING WORLDWIDE
Number of children (in millions) affected by stunting, by United Nations Sub-region, 20202
Asia
1.8
5.8
Europe
Latin America
and Caribbean
79.0
9.8%
35.8%
17.4%
Global
22.0
61.4
0.06
Oceania
Africa

## Slide 9
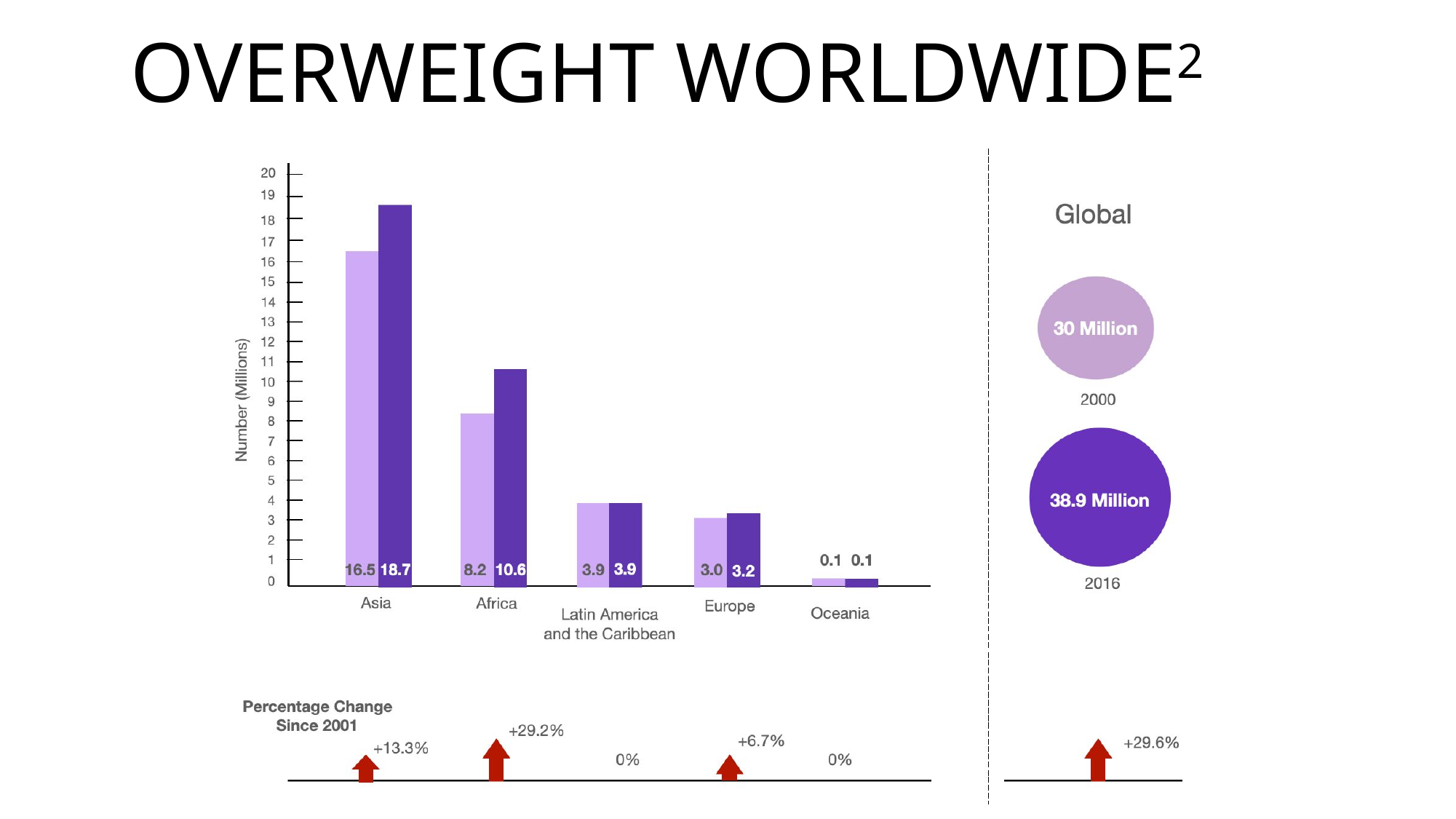

# OVERWEIGHT WORLDWIDE2

## Slide 10
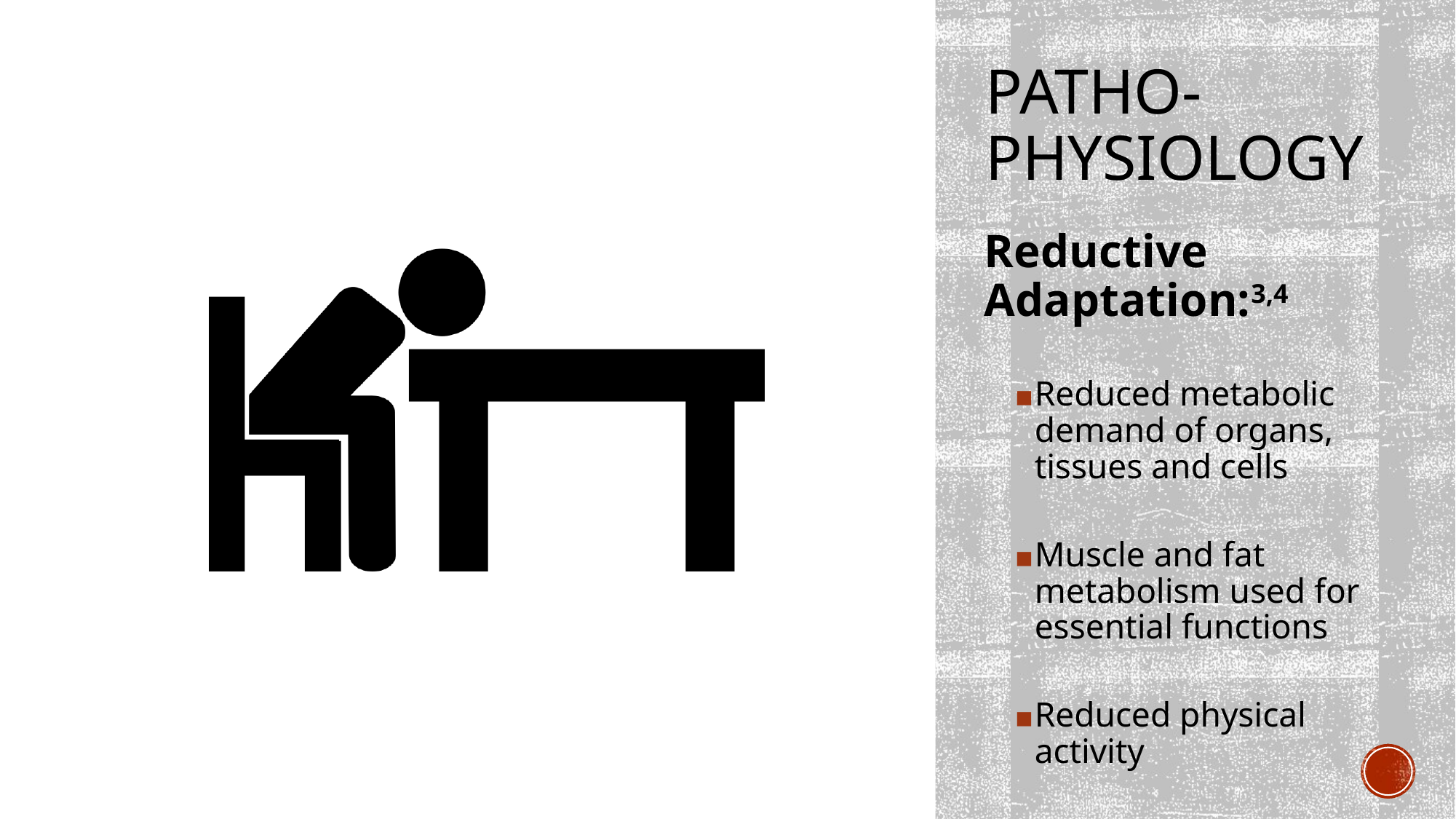

# PATHO-
PHYSIOLOGY
Reductive Adaptation:3,4
Reduced metabolic demand of organs, tissues and cells
Muscle and fat metabolism used for essential functions
Reduced physical activity

## Slide 11
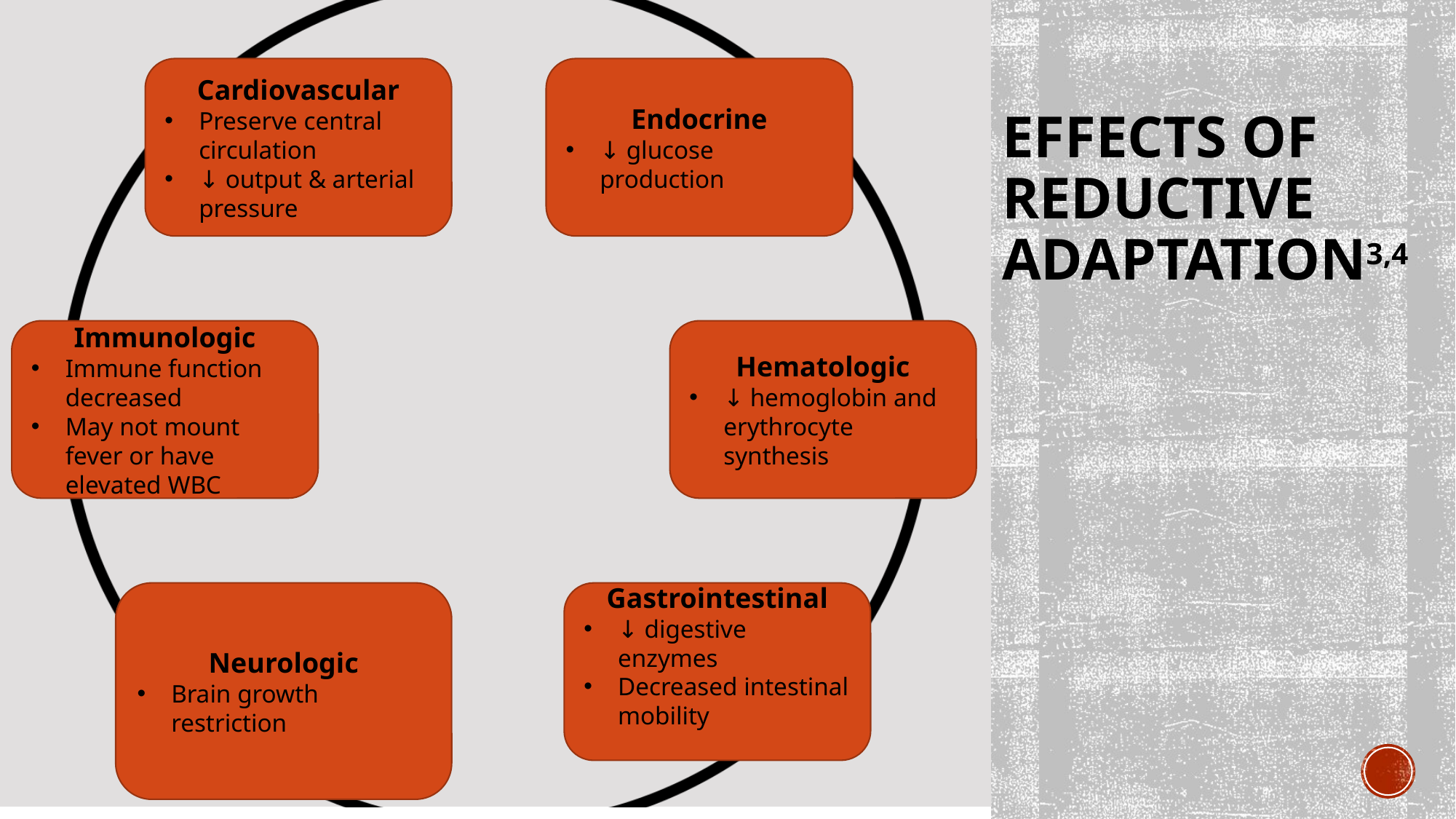

Cardiovascular
Preserve central circulation
↓ output & arterial pressure
Endocrine
↓ glucose production
# EFFECTS OF REDUCTIVE ADAPTATION3,4
Immunologic
Immune function decreased
May not mount fever or have elevated WBC
Hematologic
↓ hemoglobin and erythrocyte synthesis
Neurologic
Brain growth restriction
Gastrointestinal
↓ digestive enzymes
Decreased intestinal mobility

## Slide 12
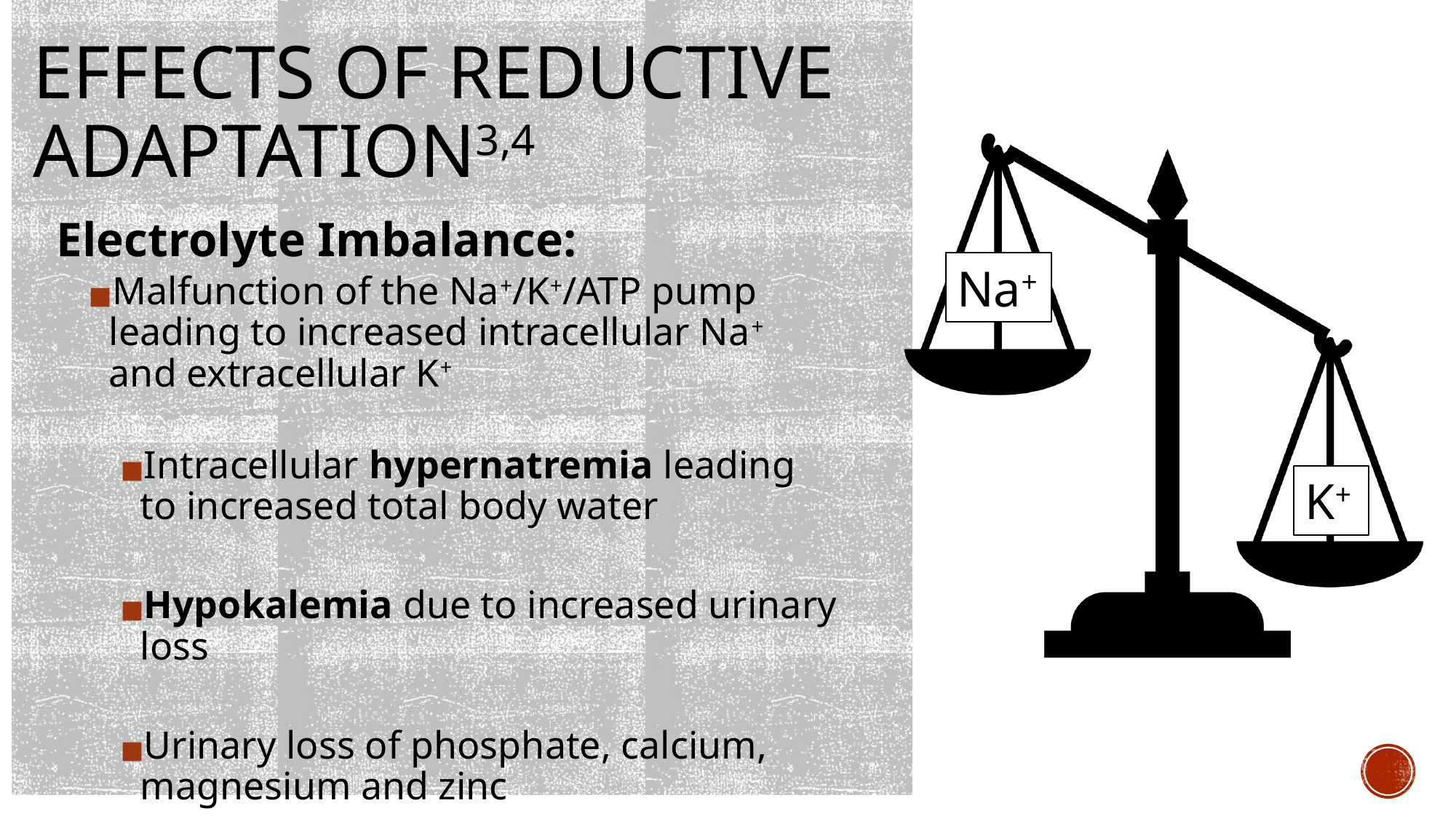

# EFFECTS OF REDUCTIVE ADAPTATION3,4
Electrolyte Imbalance:
Malfunction of the Na+/K+/ATP pump leading to increased intracellular Na+ and extracellular K+
Intracellular hypernatremia leading to increased total body water
Hypokalemia due to increased urinary loss
Urinary loss of phosphate, calcium, magnesium and zinc
Na+
K+

## Slide 13
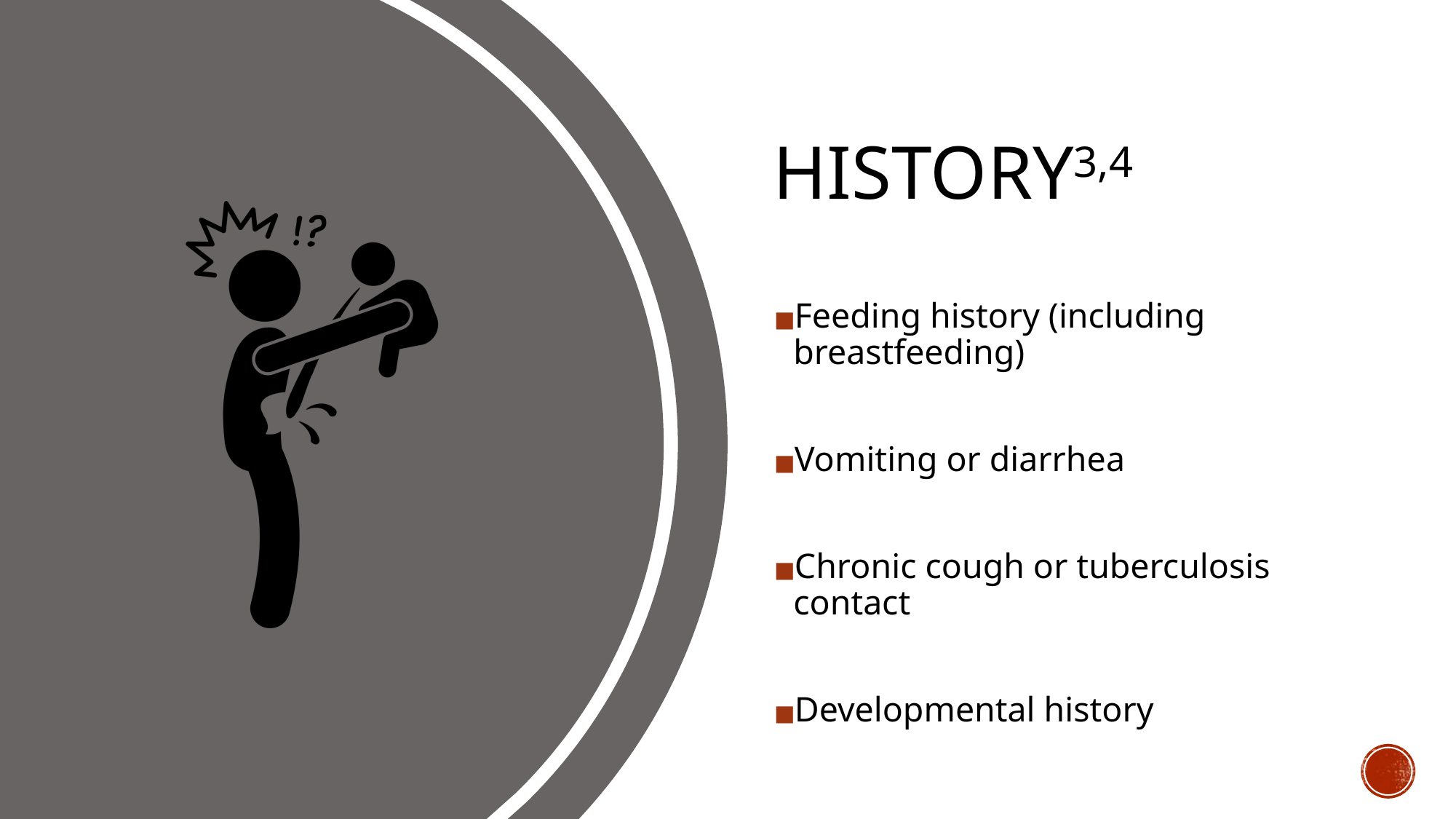

# HISTORY3,4
Feeding history (including breastfeeding)
Vomiting or diarrhea
Chronic cough or tuberculosis contact
Developmental history

## Slide 14
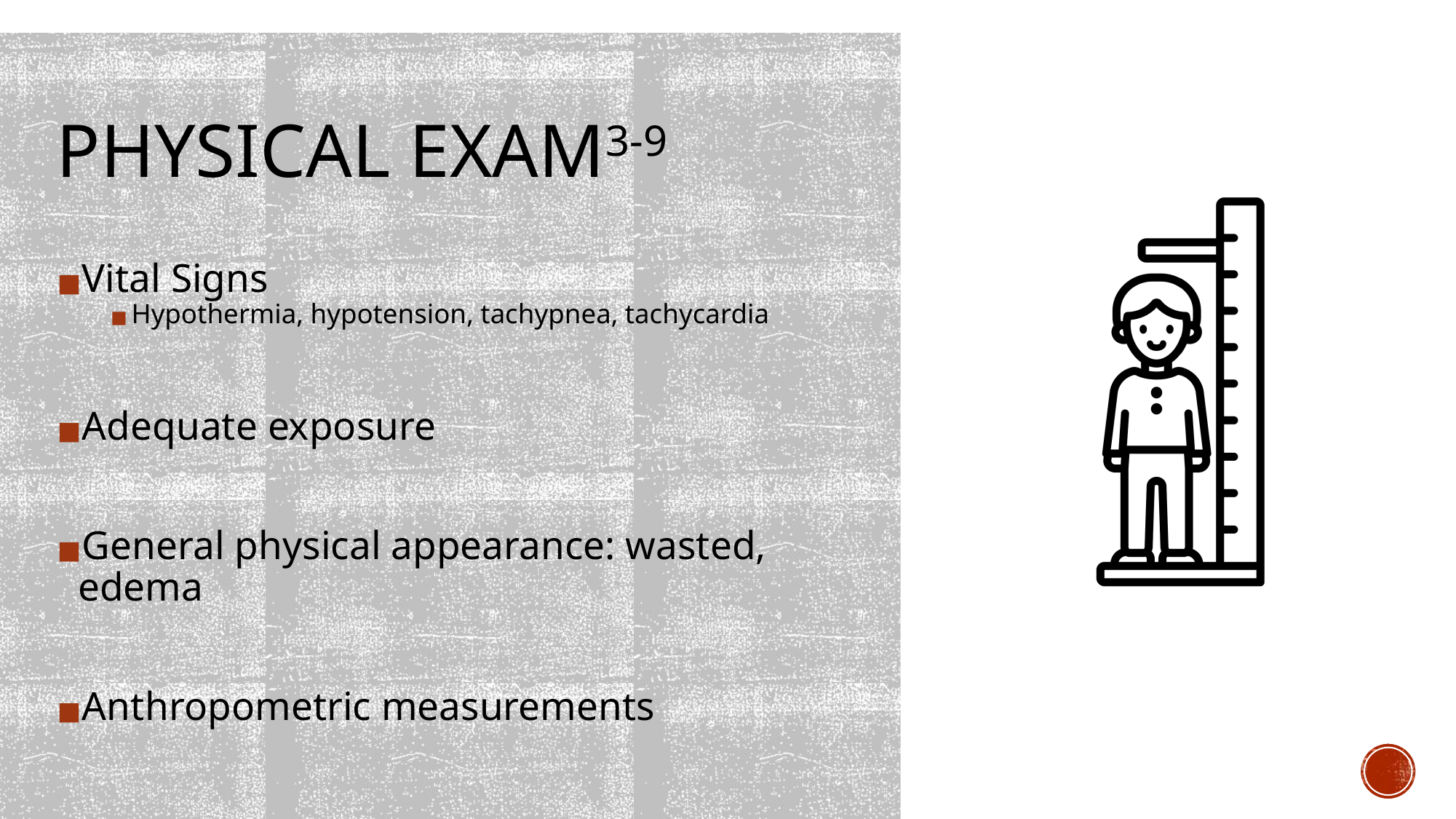

# PHYSICAL EXAM3-9
Vital Signs
Hypothermia, hypotension, tachypnea, tachycardia
Adequate exposure
General physical appearance: wasted, edema
Anthropometric measurements

## Slide 15
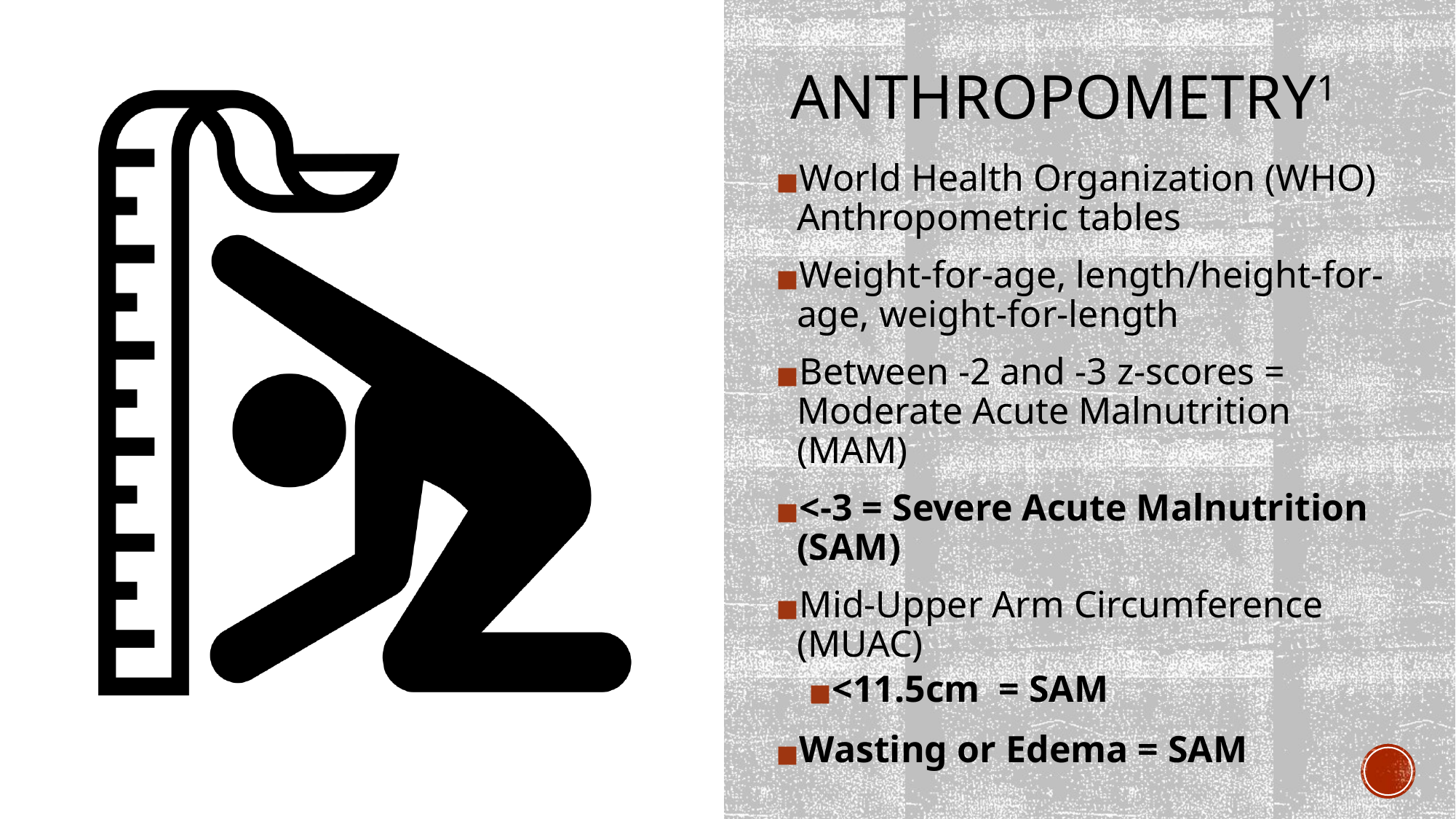

# ANTHROPOMETRY1
World Health Organization (WHO) Anthropometric tables
Weight-for-age, length/height-for-age, weight-for-length
Between -2 and -3 z-scores = Moderate Acute Malnutrition (MAM)
<-3 = Severe Acute Malnutrition (SAM)
Mid-Upper Arm Circumference (MUAC)
<11.5cm = SAM
Wasting or Edema = SAM

## Slide 16
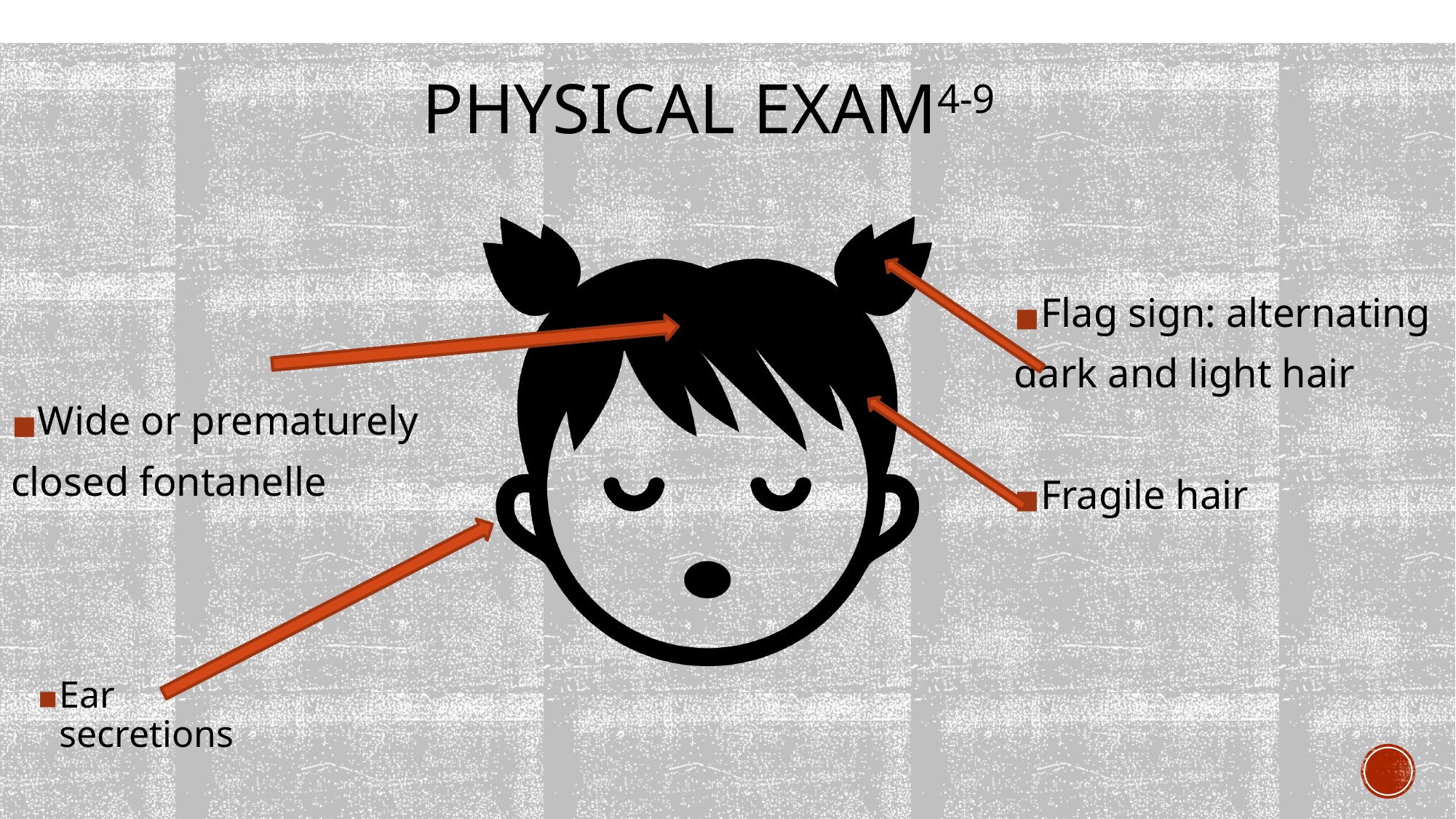

# PHYSICAL EXAM4-9
Wide or prematurely
closed fontanelle
Flag sign: alternating
dark and light hair
Fragile hair
Ear secretions

## Slide 17
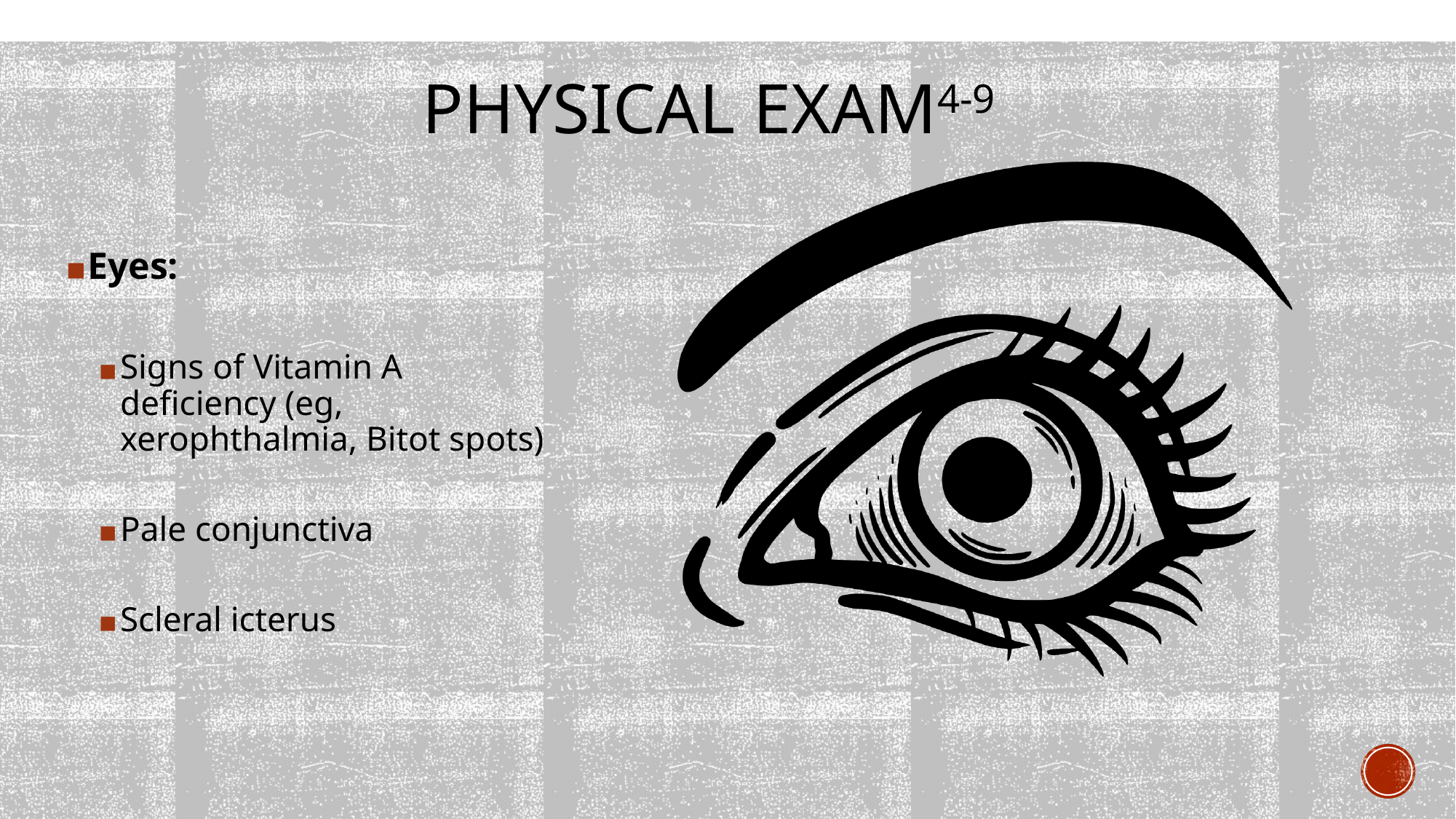

# PHYSICAL EXAM4-9
Eyes:
Signs of Vitamin A deficiency (eg, xerophthalmia, Bitot spots)
Pale conjunctiva
Scleral icterus

## Slide 18
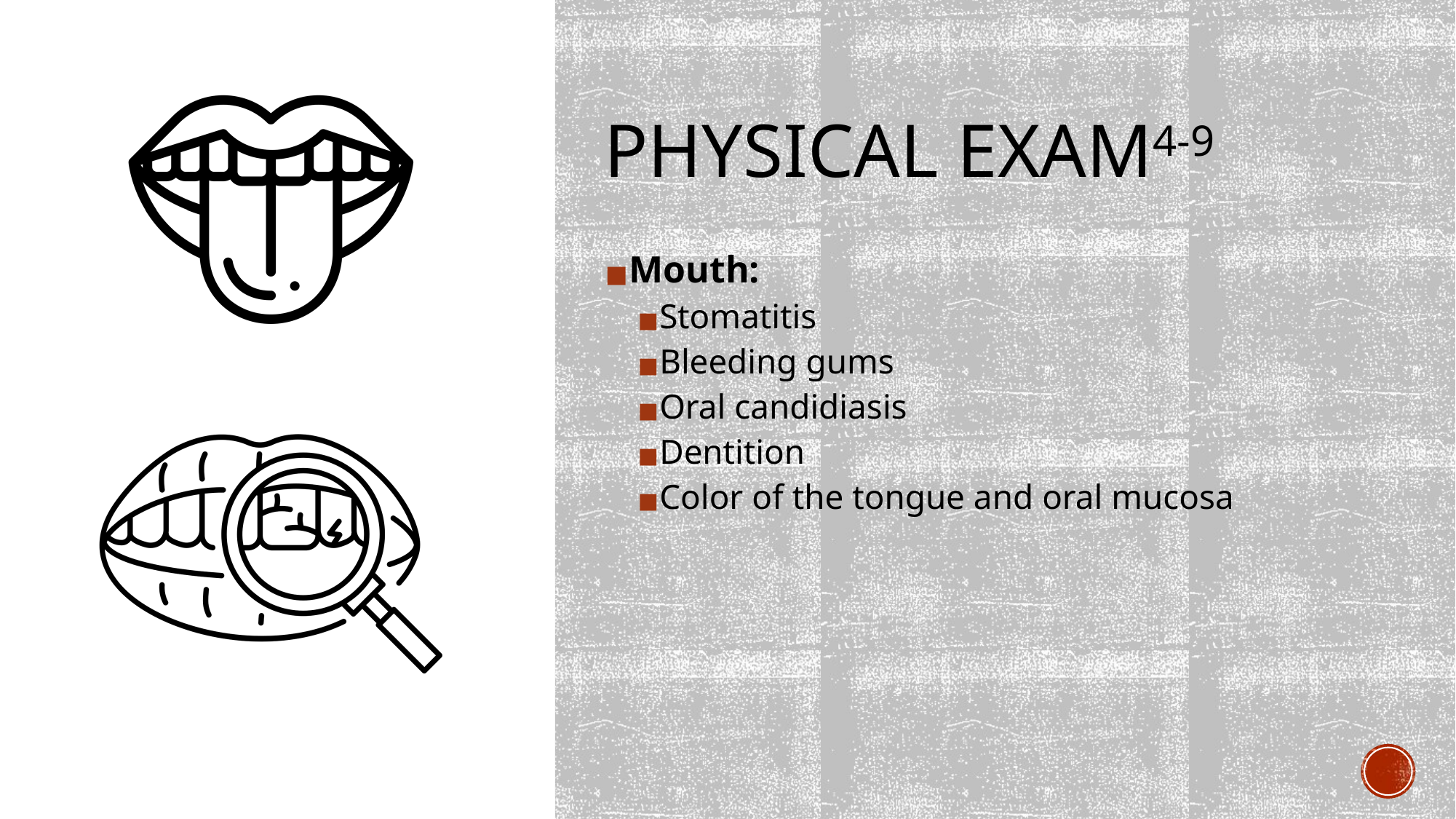

# PHYSICAL EXAM4-9
Mouth:
Stomatitis
Bleeding gums
Oral candidiasis
Dentition
Color of the tongue and oral mucosa

## Slide 19
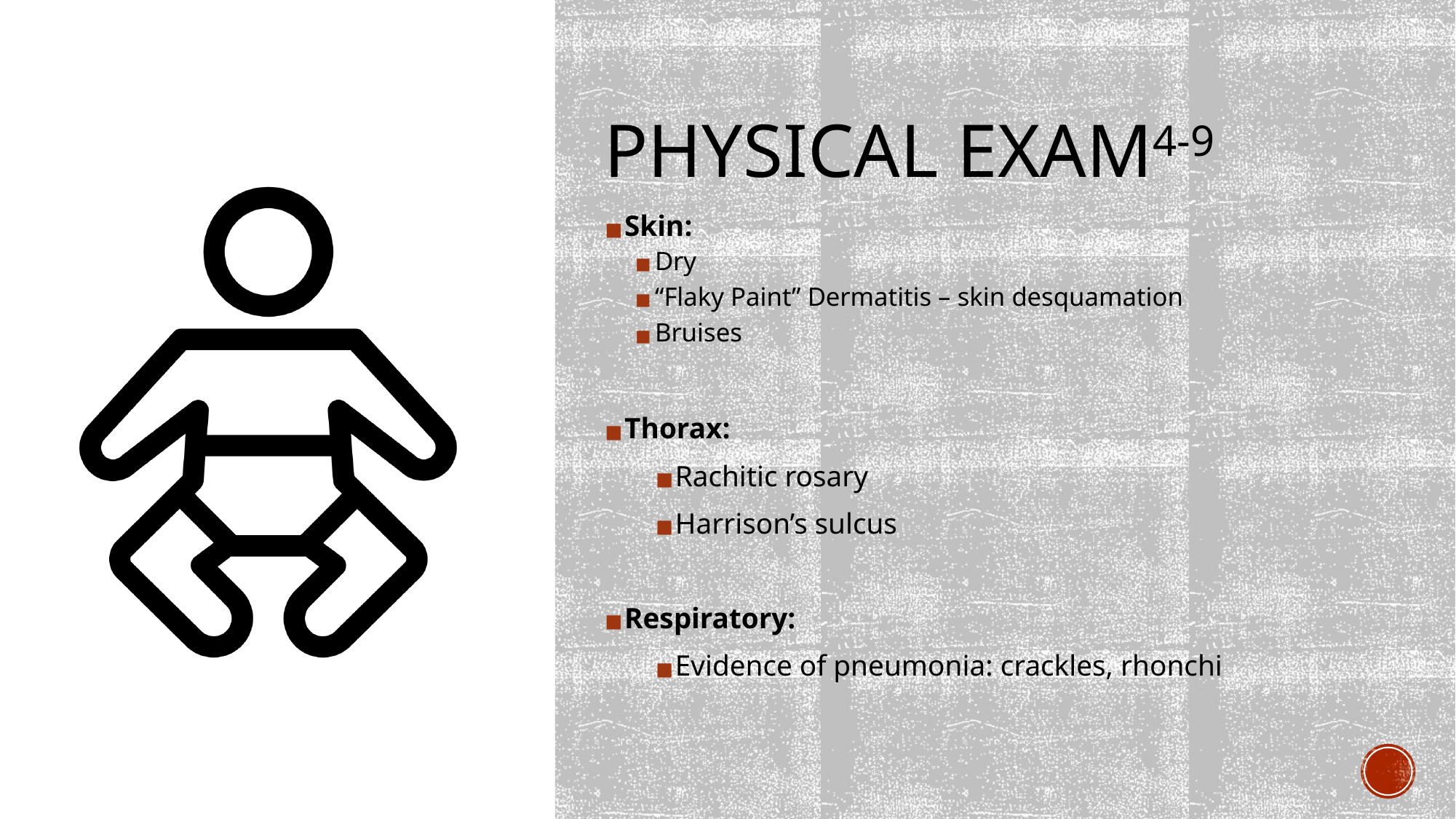

# PHYSICAL EXAM4-9
Skin:
Dry
“Flaky Paint” Dermatitis – skin desquamation
Bruises
Thorax:
Rachitic rosary
Harrison’s sulcus
Respiratory:
Evidence of pneumonia: crackles, rhonchi

## Slide 20
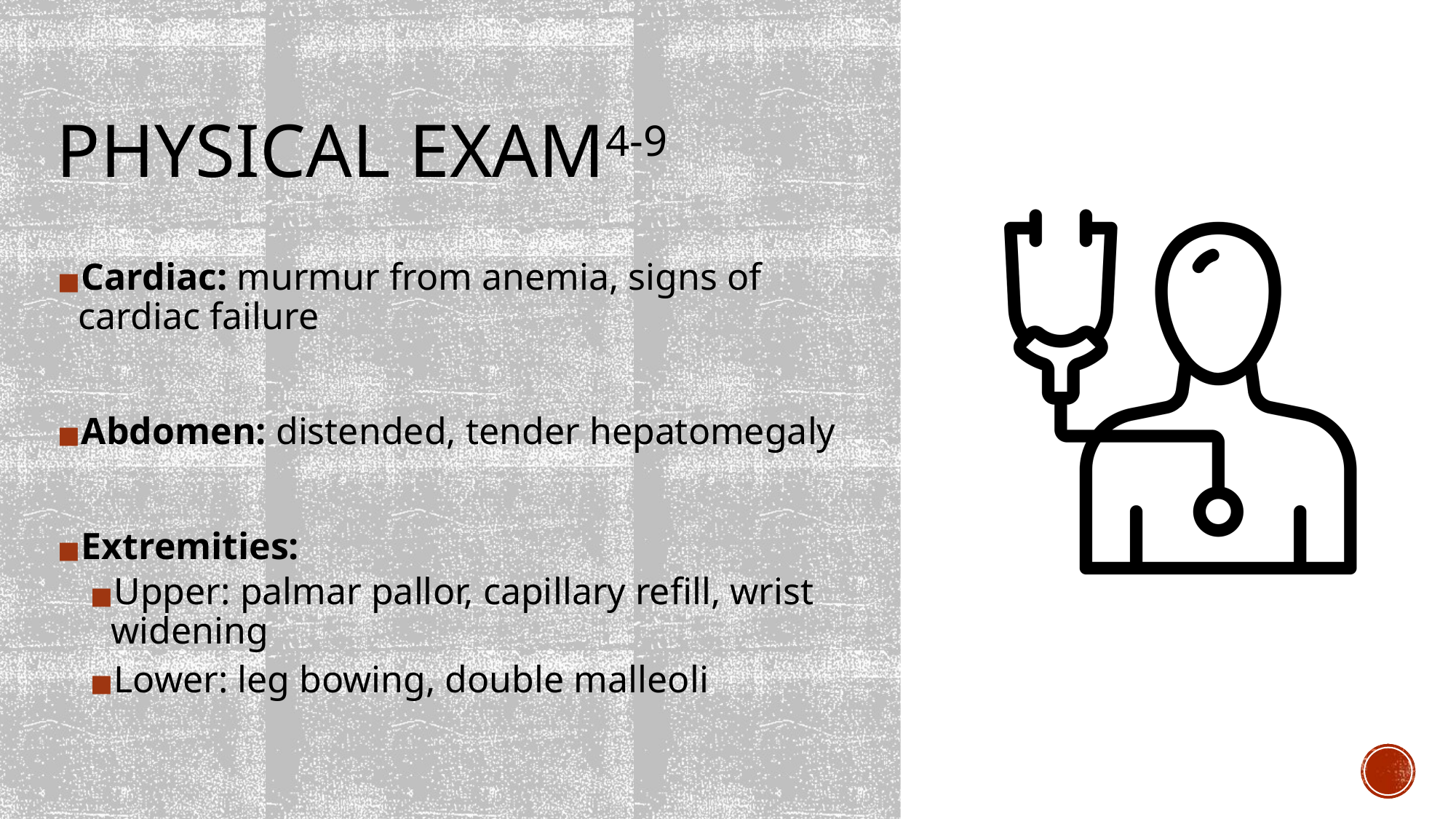

# PHYSICAL EXAM4-9
Cardiac: murmur from anemia, signs of cardiac failure
Abdomen: distended, tender hepatomegaly
Extremities:
Upper: palmar pallor, capillary refill, wrist widening
Lower: leg bowing, double malleoli

## Slide 21
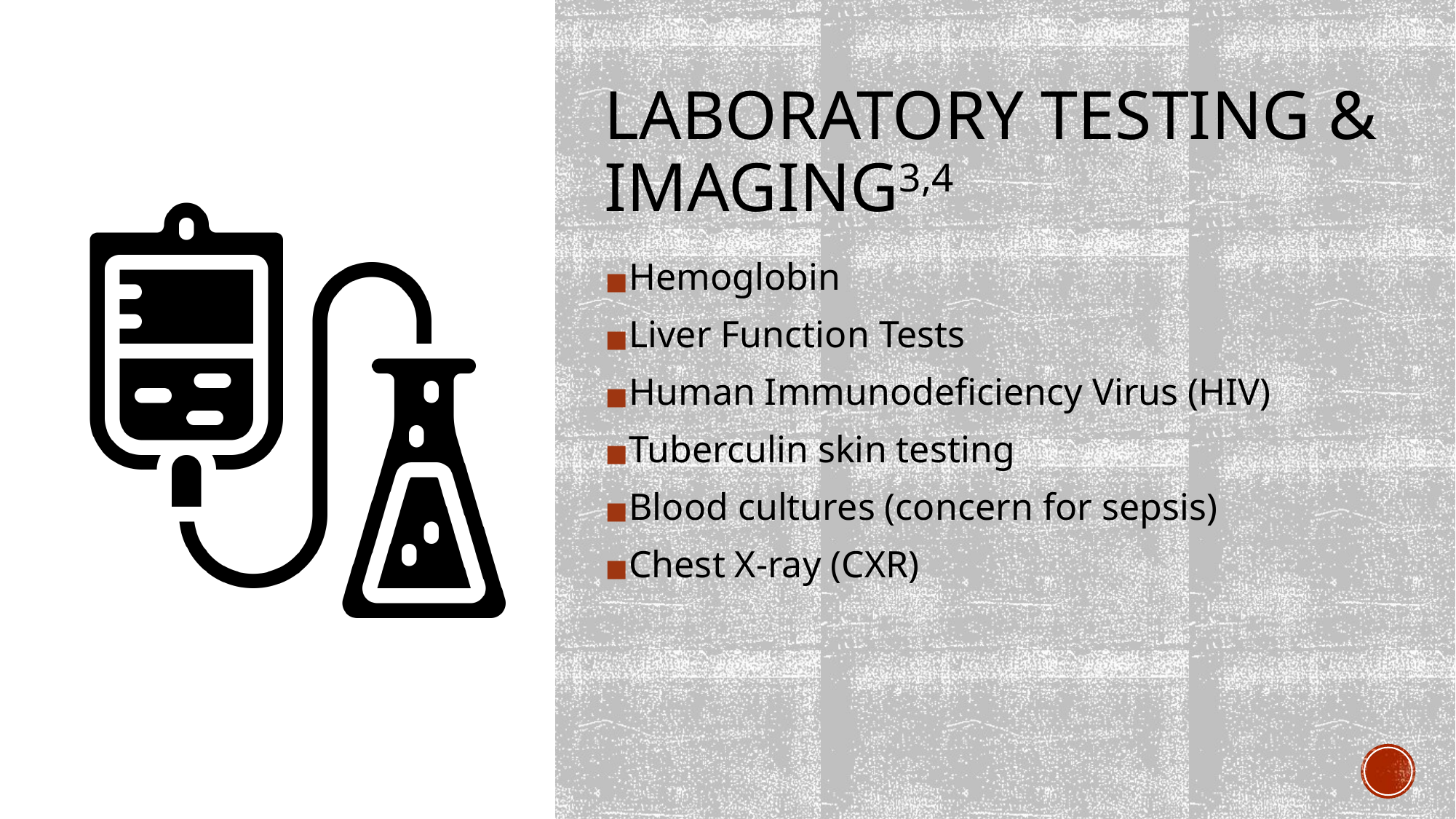

# LABORATORY TESTING & IMAGING3,4
Hemoglobin
Liver Function Tests
Human Immunodeficiency Virus (HIV)
Tuberculin skin testing
Blood cultures (concern for sepsis)
Chest X-ray (CXR)

## Slide 22
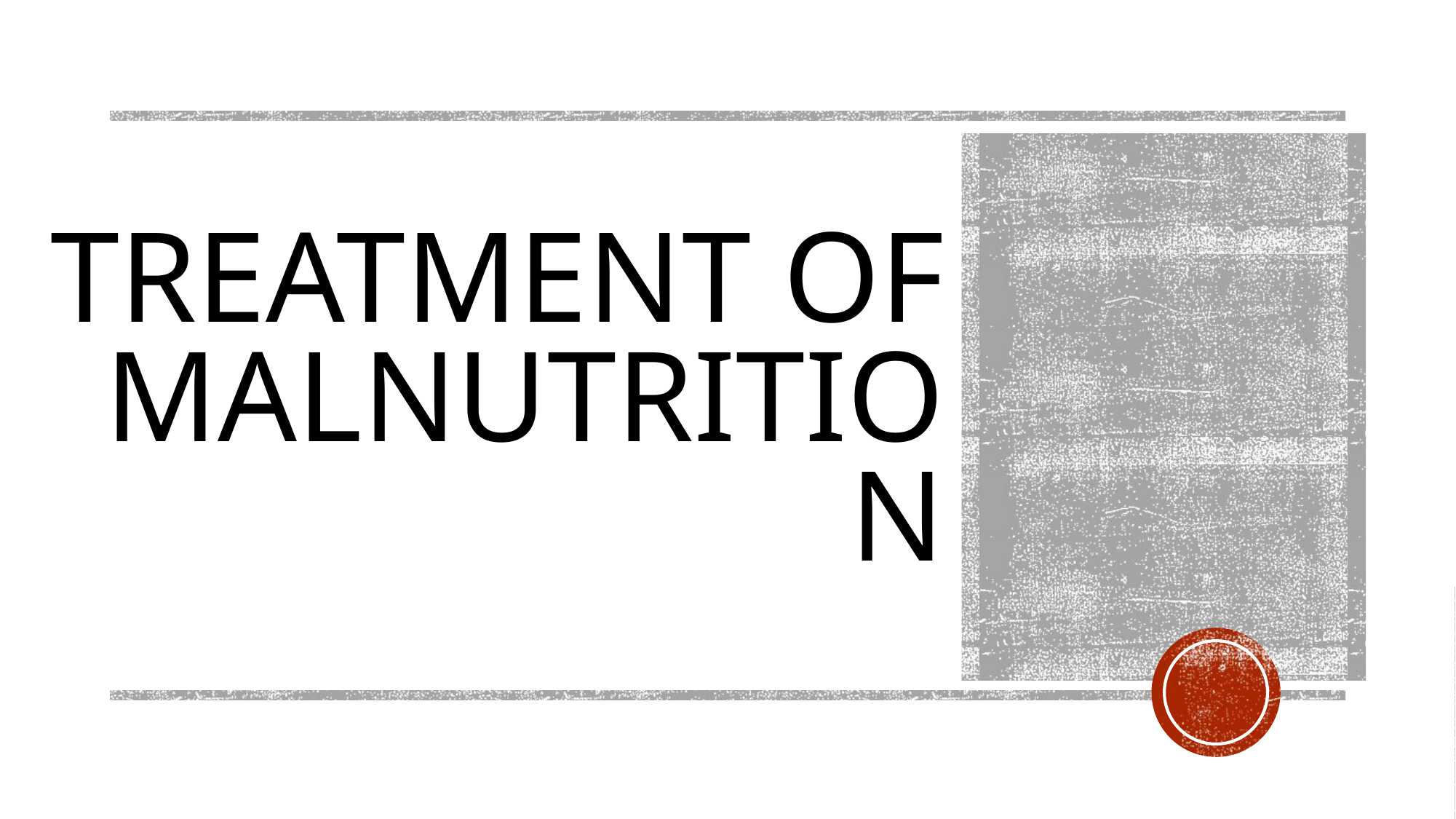

# TREATMENT OF MALNUTRITION

## Slide 23
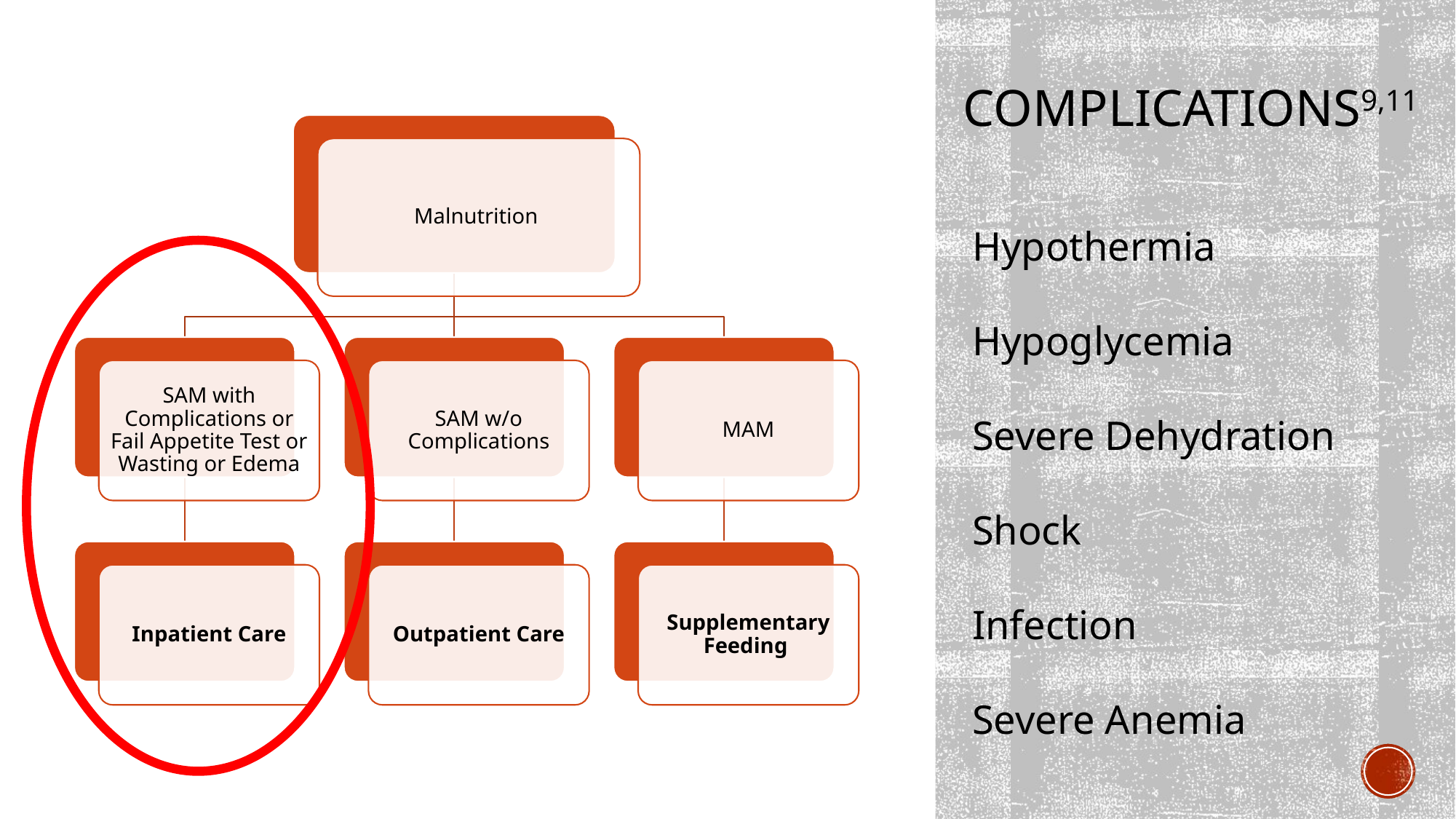

COMPLICATIONS9,11
Malnutrition
SAM with Complications or Fail Appetite Test or Wasting or Edema
SAM w/o Complications
MAM
Inpatient Care
Outpatient Care
Supplementary Feeding
Hypothermia
Hypoglycemia
Severe Dehydration
Shock
Infection
Severe Anemia

## Slide 24
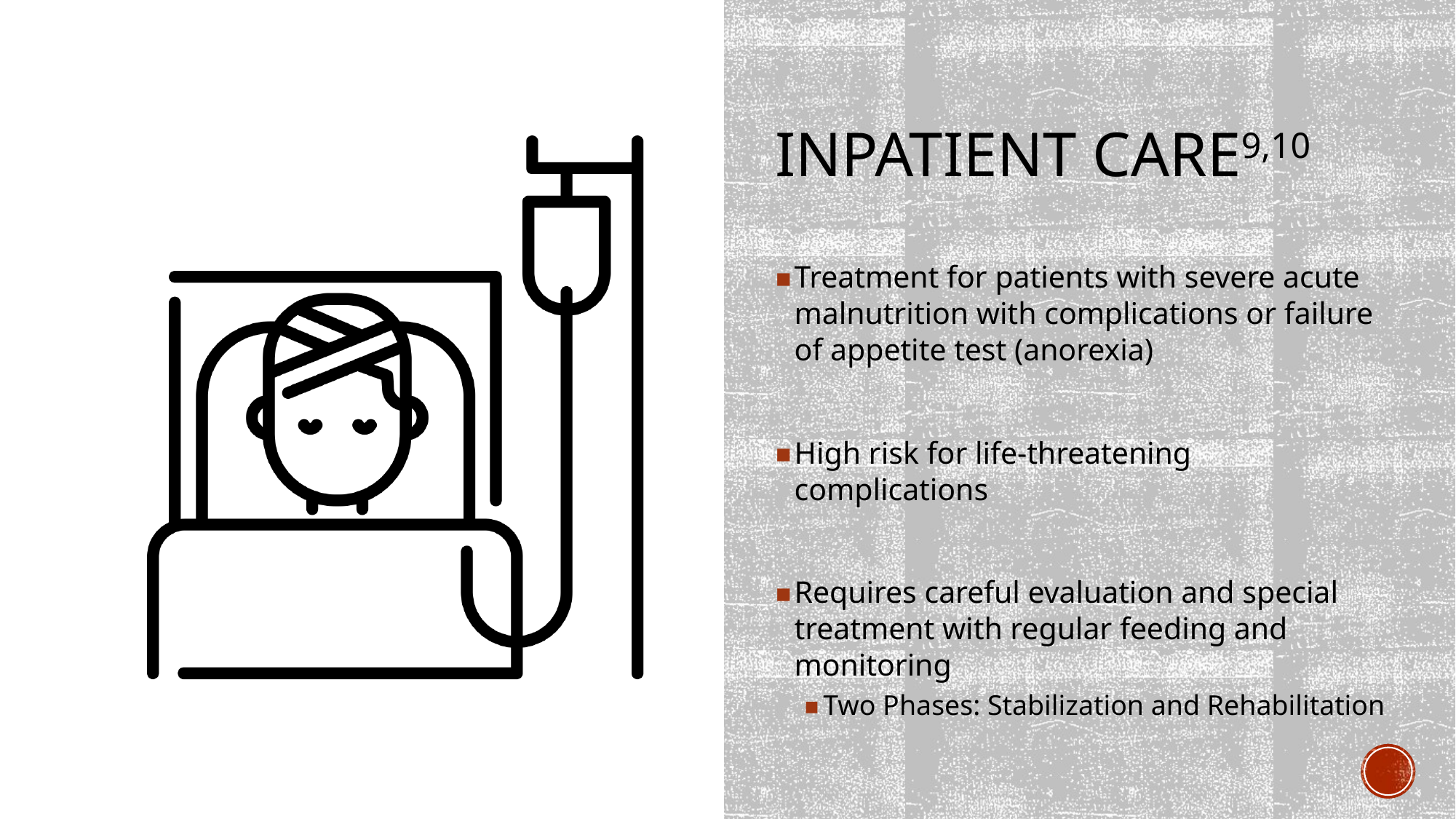

# INPATIENT CARE9,10
Treatment for patients with severe acute malnutrition with complications or failure of appetite test (anorexia)
High risk for life-threatening complications
Requires careful evaluation and special treatment with regular feeding and monitoring
Two Phases: Stabilization and Rehabilitation

## Slide 25
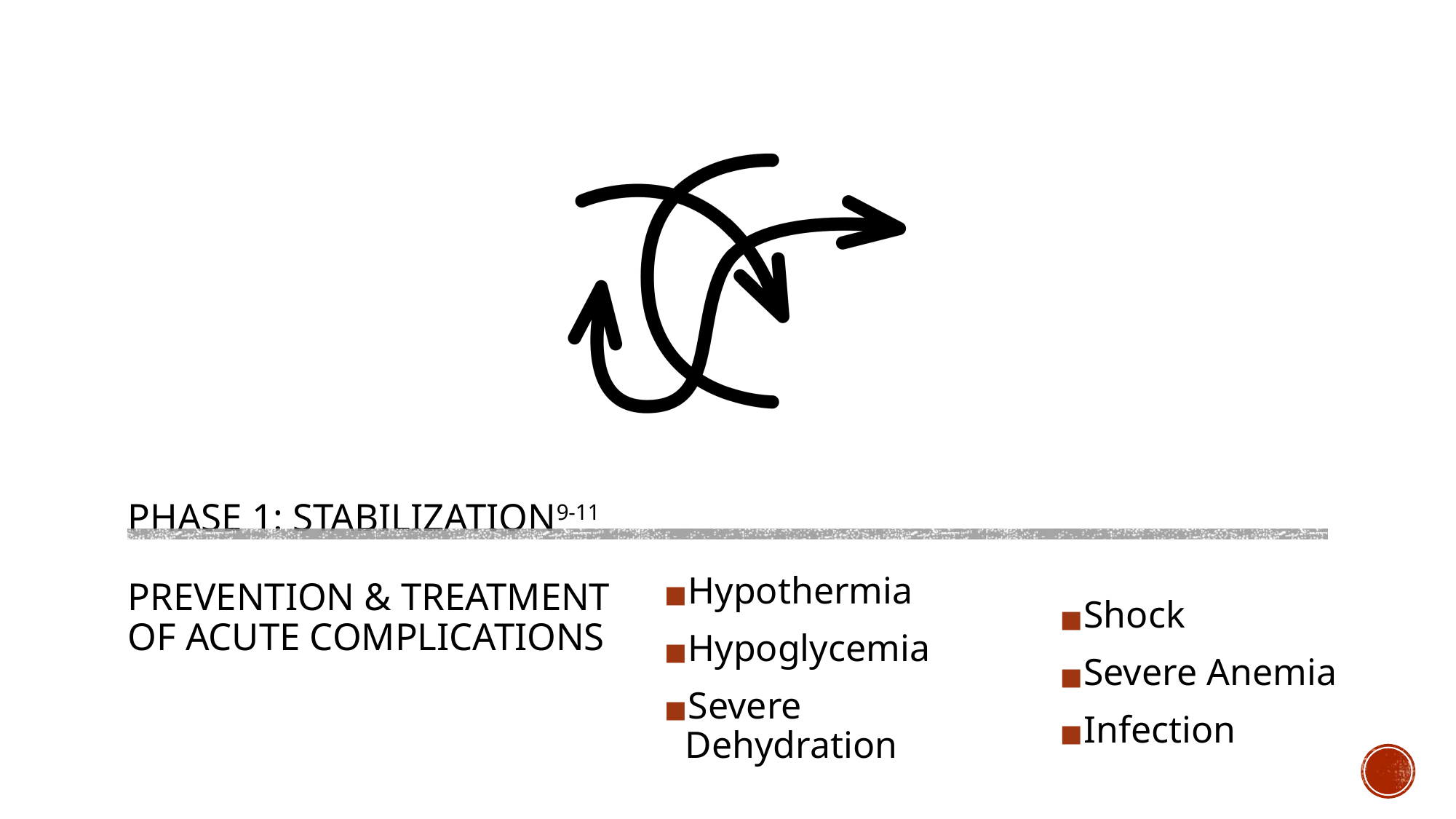

# PHASE 1: STABILIZATION9-11 PREVENTION & TREATMENT OF ACUTE COMPLICATIONS
Hypothermia
Hypoglycemia
Severe Dehydration
Shock
Severe Anemia
Infection

## Slide 26
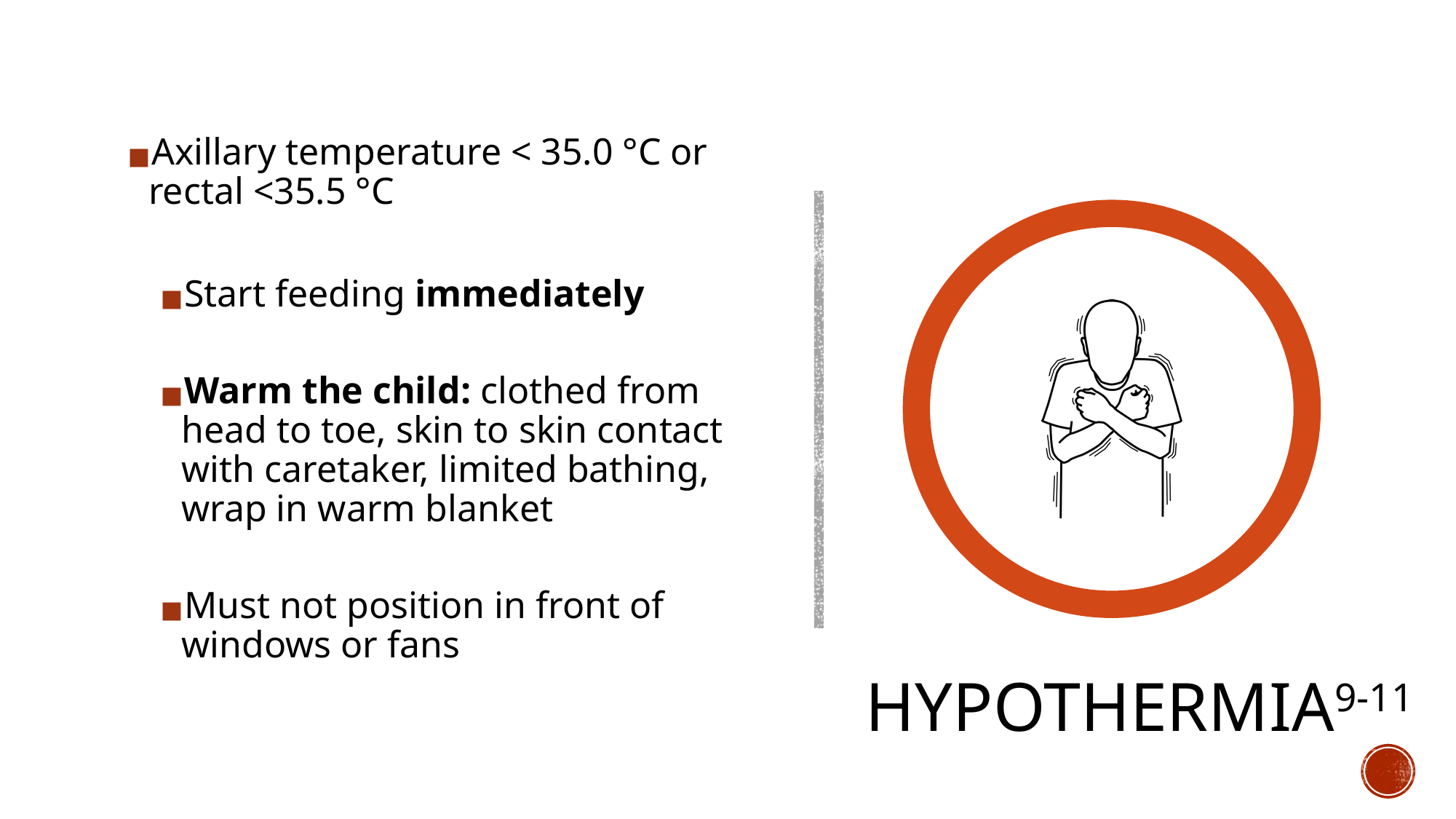

Axillary temperature < 35.0 °C or rectal <35.5 °C
Start feeding immediately
Warm the child: clothed from head to toe, skin to skin contact with caretaker, limited bathing, wrap in warm blanket
Must not position in front of windows or fans
# HYPOTHERMIA9-11

## Slide 27
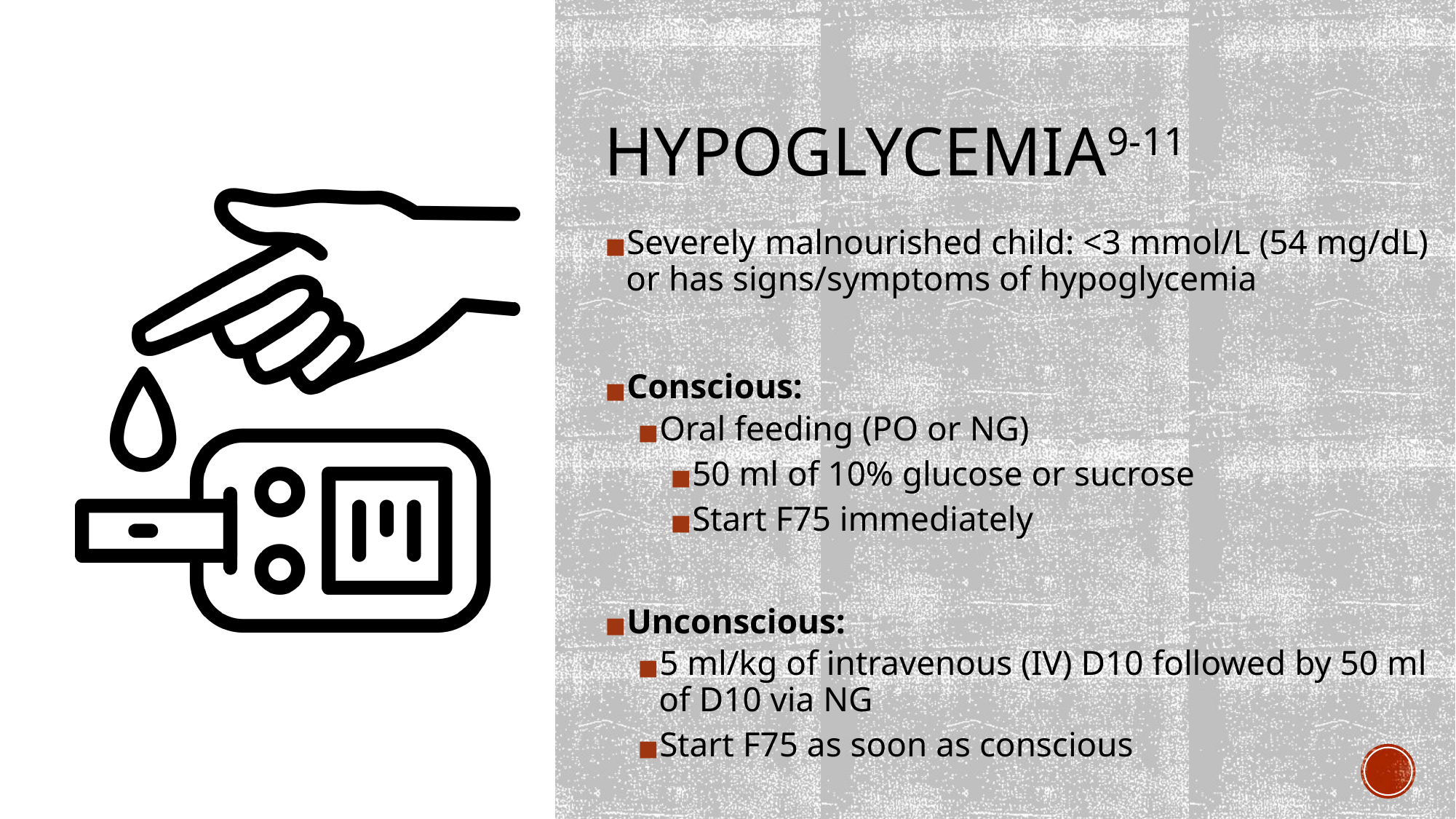

# HYPOGLYCEMIA9-11
Severely malnourished child: <3 mmol/L (54 mg/dL) or has signs/symptoms of hypoglycemia
Conscious:
Oral feeding (PO or NG)
50 ml of 10% glucose or sucrose
Start F75 immediately
Unconscious:
5 ml/kg of intravenous (IV) D10 followed by 50 ml of D10 via NG
Start F75 as soon as conscious

## Slide 28
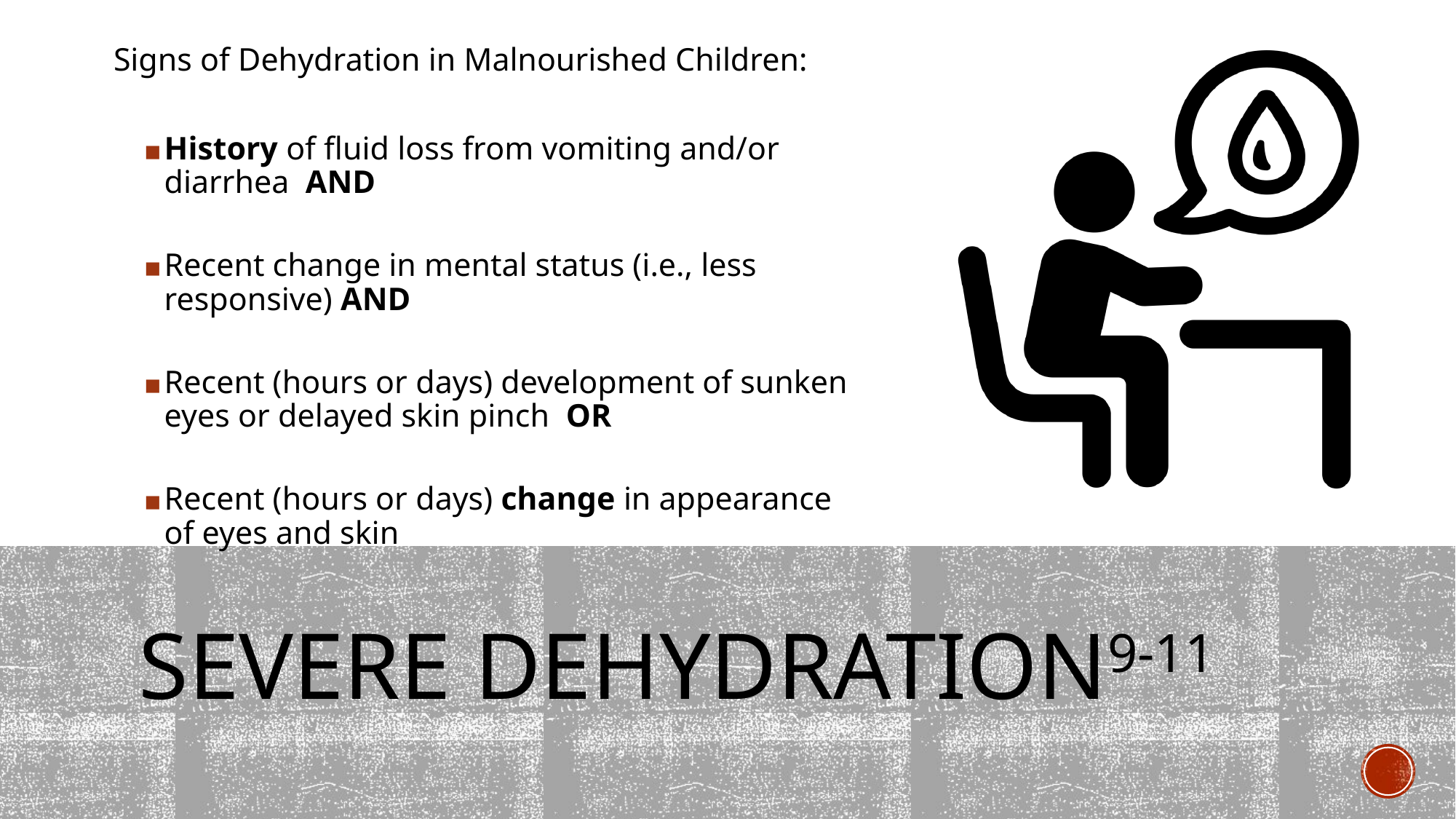

Signs of Dehydration in Malnourished Children:
History of fluid loss from vomiting and/or diarrhea AND
Recent change in mental status (i.e., less responsive) AND
Recent (hours or days) development of sunken eyes or delayed skin pinch OR
Recent (hours or days) change in appearance of eyes and skin
# SEVERE DEHYDRATION9-11

## Slide 29
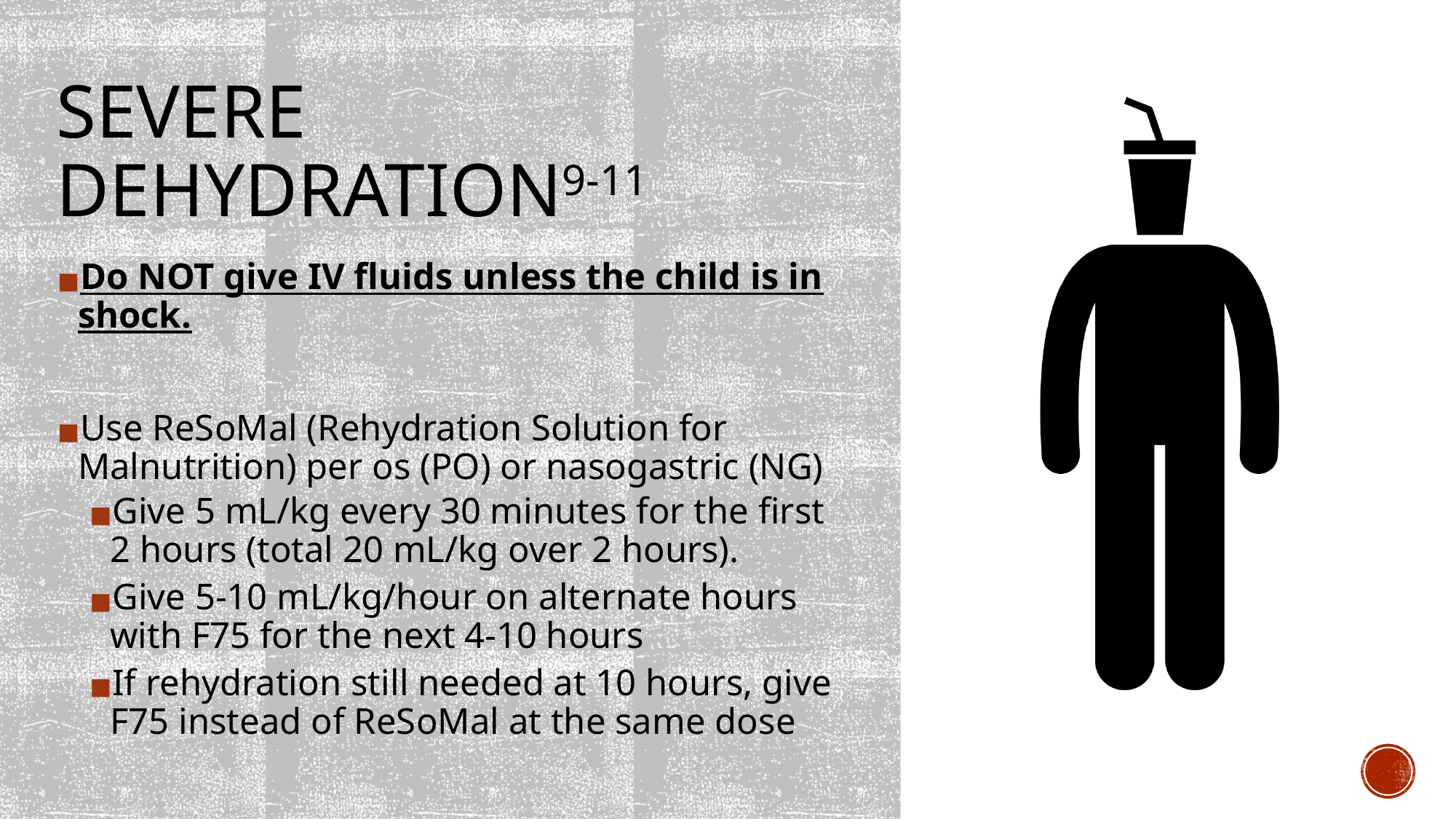

# SEVERE DEHYDRATION9-11
Do NOT give IV fluids unless the child is in shock.
Use ReSoMal (Rehydration Solution for Malnutrition) per os (PO) or nasogastric (NG)
Give 5 mL/kg every 30 minutes for the first 2 hours (total 20 mL/kg over 2 hours).
Give 5-10 mL/kg/hour on alternate hours with F75 for the next 4-10 hours
If rehydration still needed at 10 hours, give F75 instead of ReSoMal at the same dose

## Slide 30
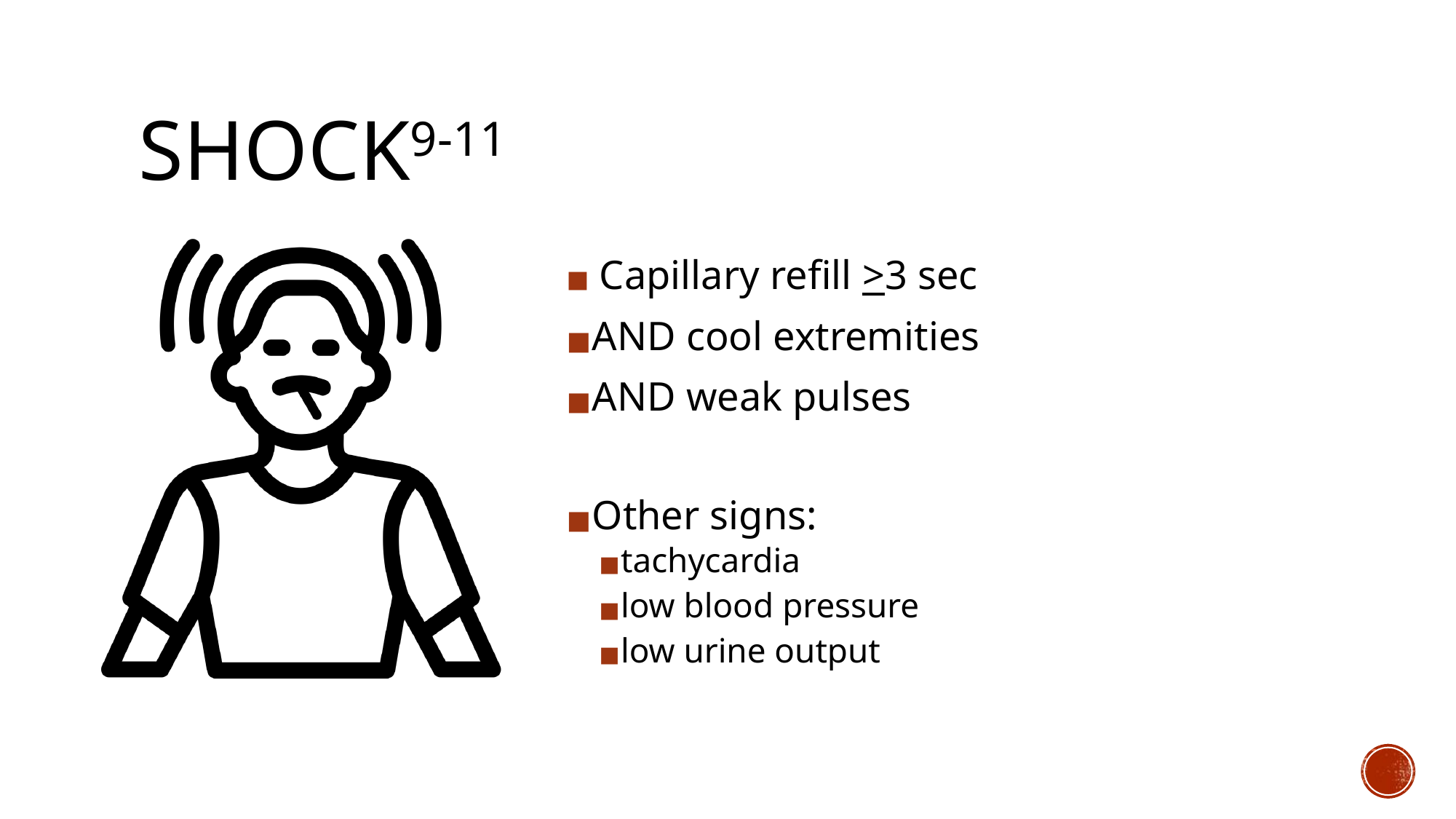

# SHOCK9-11
 Capillary refill >3 sec
AND cool extremities
AND weak pulses
Other signs:
tachycardia
low blood pressure
low urine output

## Slide 31
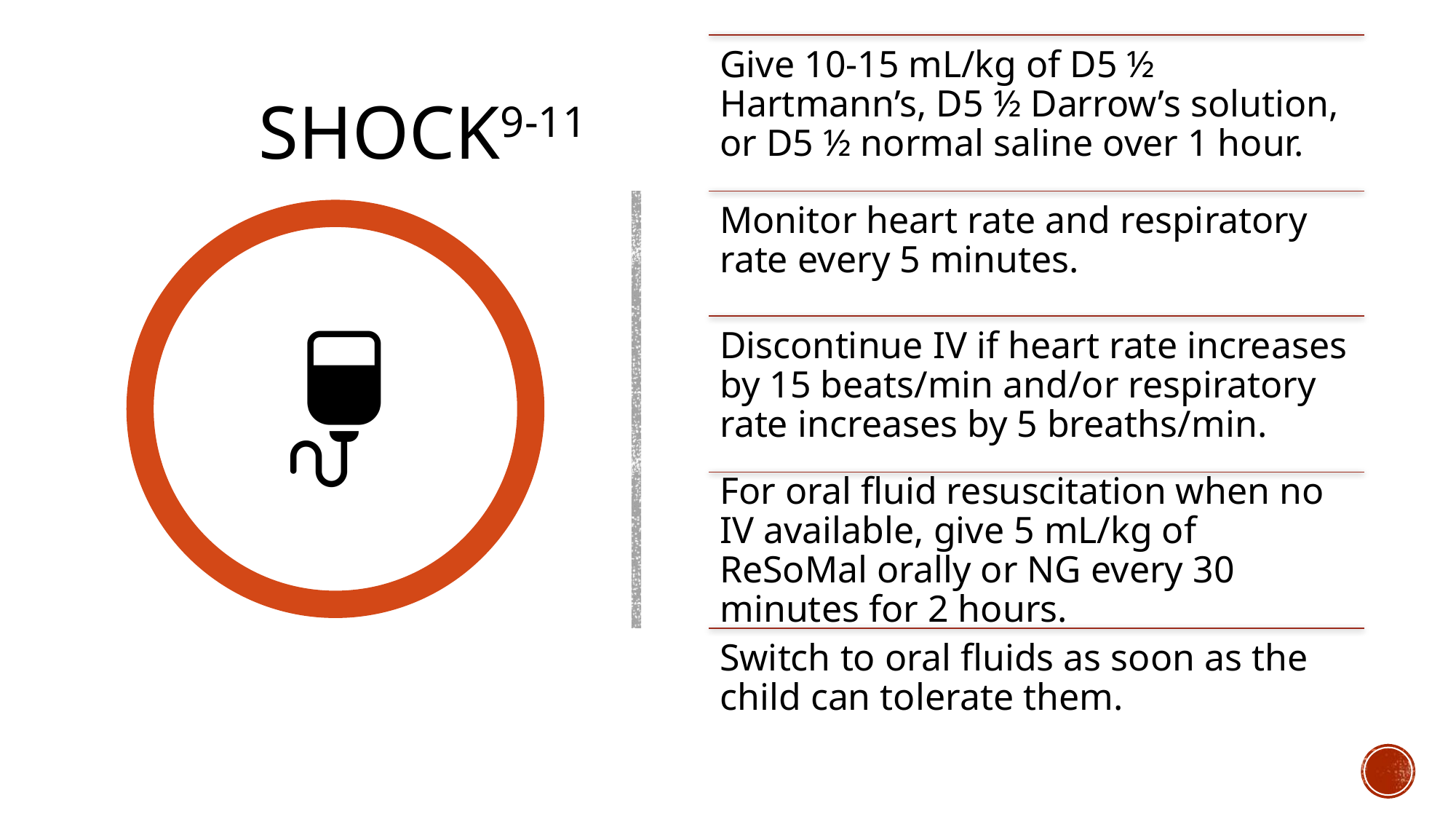

# SHOCK9-11
Give 10-15 mL/kg of D5 ½ Hartmann’s, D5 ½ Darrow’s solution, or D5 ½ normal saline over 1 hour.
Monitor heart rate and respiratory rate every 5 minutes.
Discontinue IV if heart rate increases by 15 beats/min and/or respiratory rate increases by 5 breaths/min.
For oral fluid resuscitation when no IV available, give 5 mL/kg of ReSoMal orally or NG every 30 minutes for 2 hours.
Switch to oral fluids as soon as the child can tolerate them.

## Slide 32
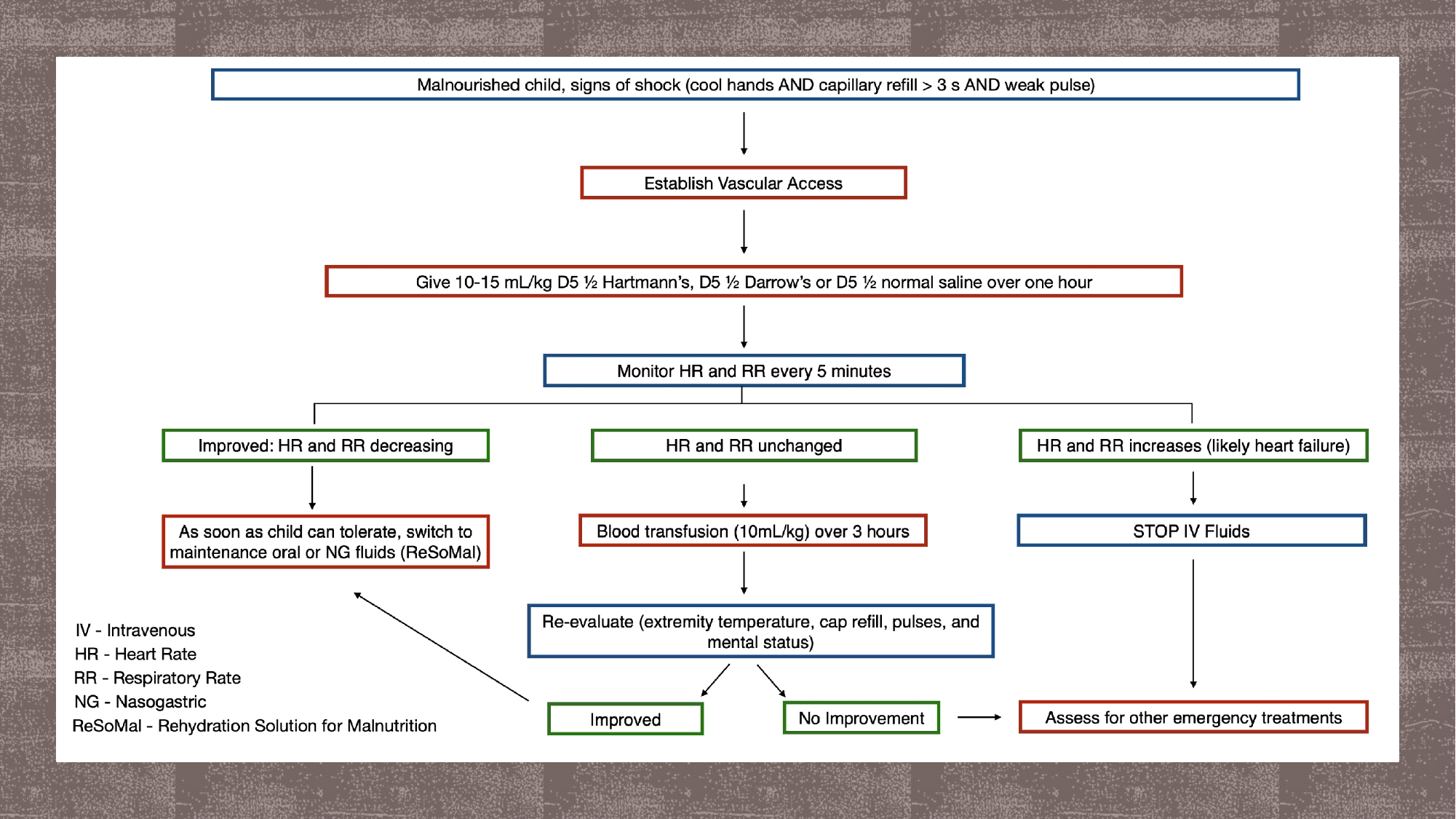

## Slide 33
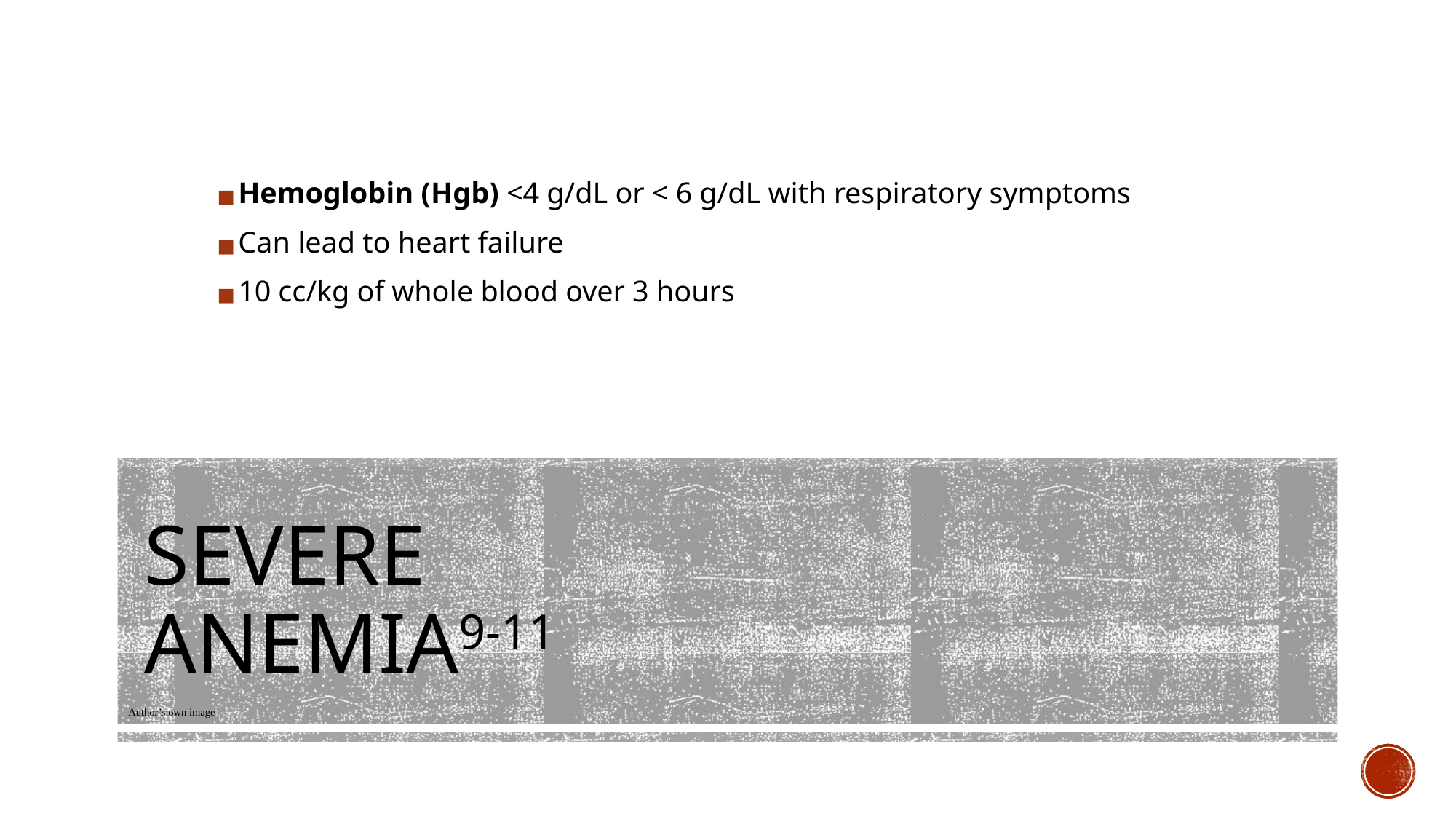

Hemoglobin (Hgb) <4 g/dL or < 6 g/dL with respiratory symptoms
Can lead to heart failure
10 cc/kg of whole blood over 3 hours
# SEVERE ANEMIA9-11
Author’s own image

## Slide 34
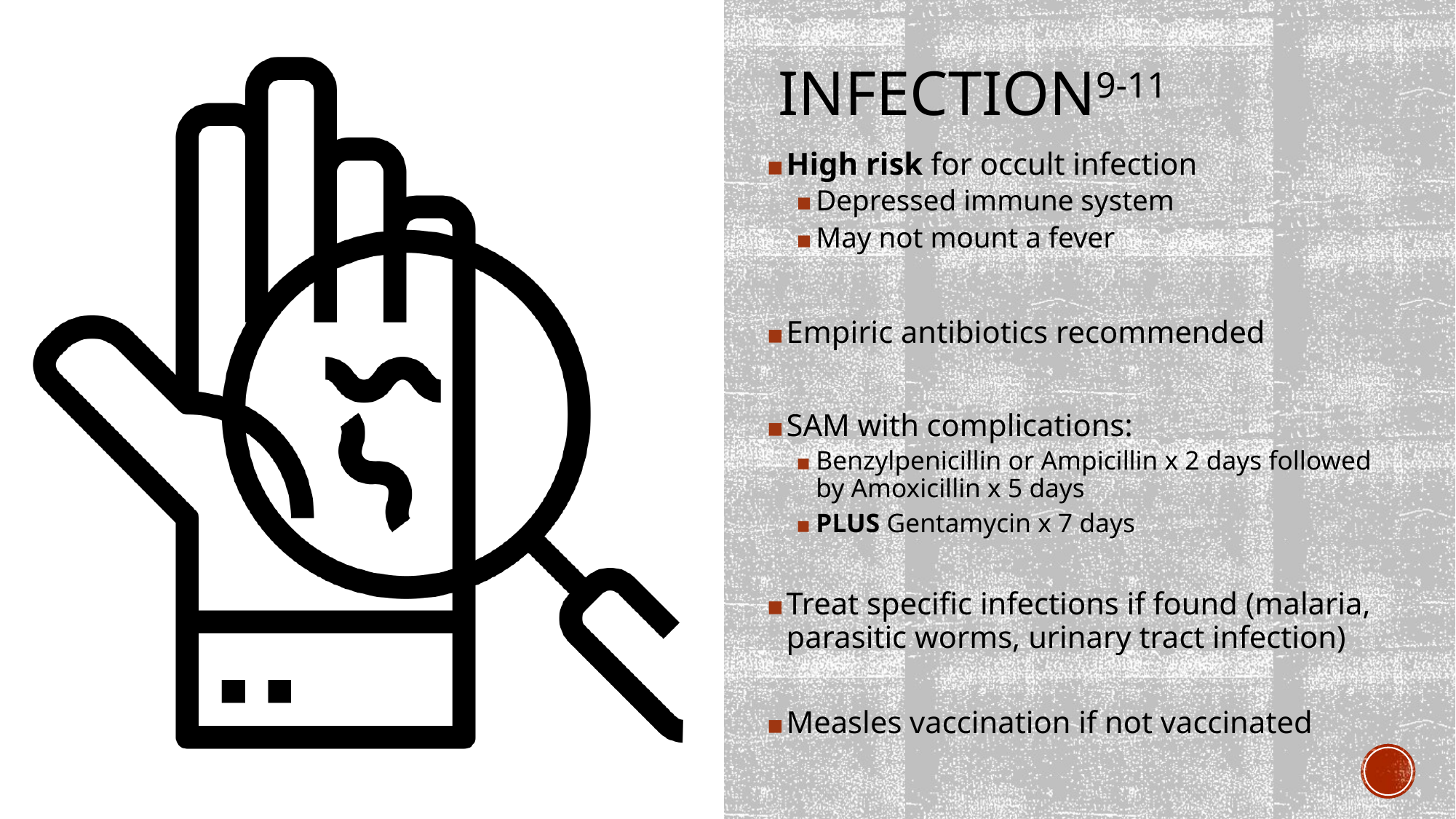

# INFECTION9-11
High risk for occult infection
Depressed immune system
May not mount a fever
Empiric antibiotics recommended
SAM with complications:
Benzylpenicillin or Ampicillin x 2 days followed by Amoxicillin x 5 days
PLUS Gentamycin x 7 days
Treat specific infections if found (malaria, parasitic worms, urinary tract infection)
Measles vaccination if not vaccinated

## Slide 35
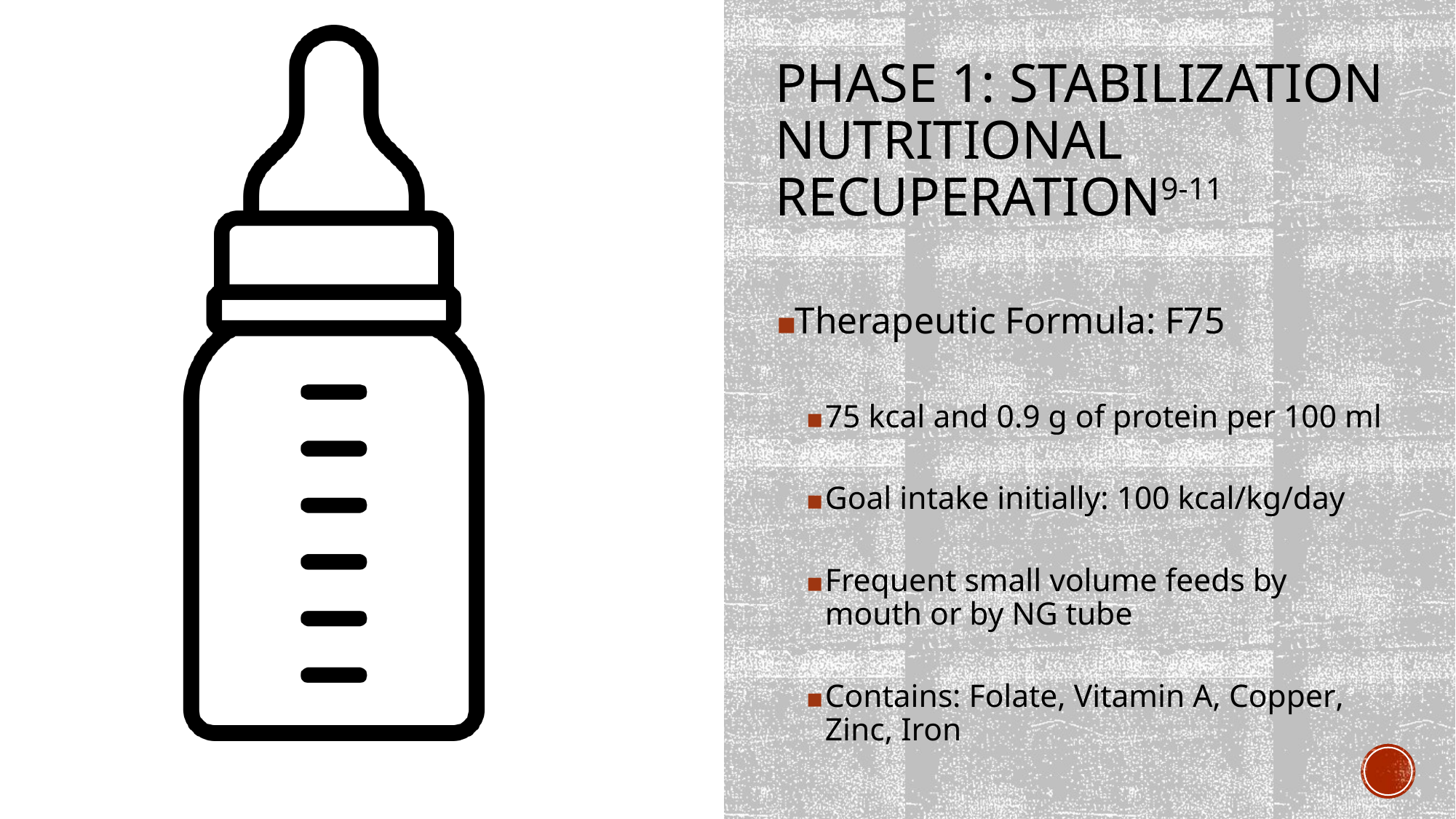

# PHASE 1: STABILIZATION NUTRITIONAL RECUPERATION9-11
Therapeutic Formula: F75
75 kcal and 0.9 g of protein per 100 ml
Goal intake initially: 100 kcal/kg/day
Frequent small volume feeds by mouth or by NG tube
Contains: Folate, Vitamin A, Copper, Zinc, Iron

## Slide 36
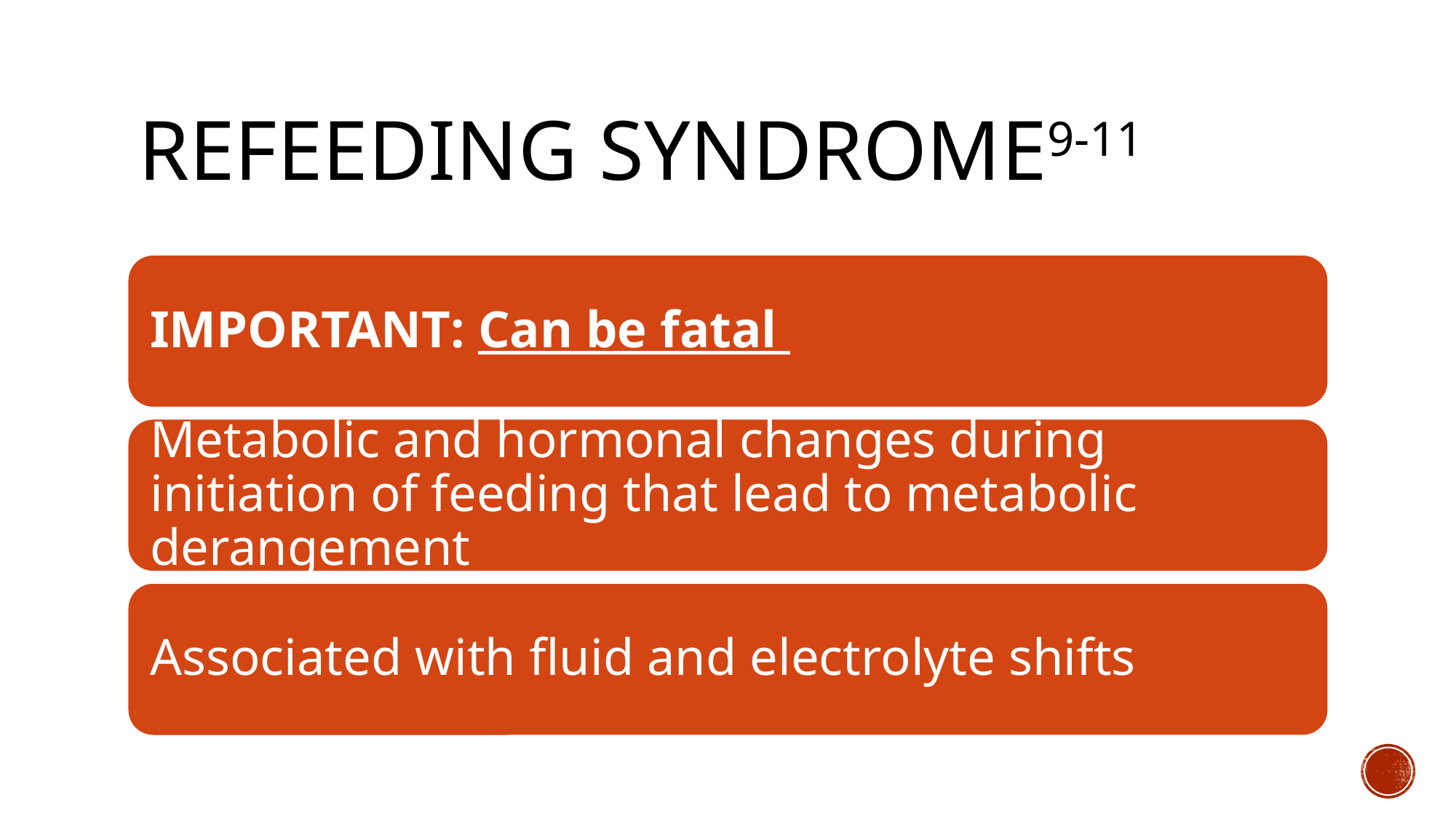

# REFEEDING SYNDROME9-11
IMPORTANT: Can be fatal
Metabolic and hormonal changes during initiation of feeding that lead to metabolic derangement
Associated with fluid and electrolyte shifts

## Slide 37
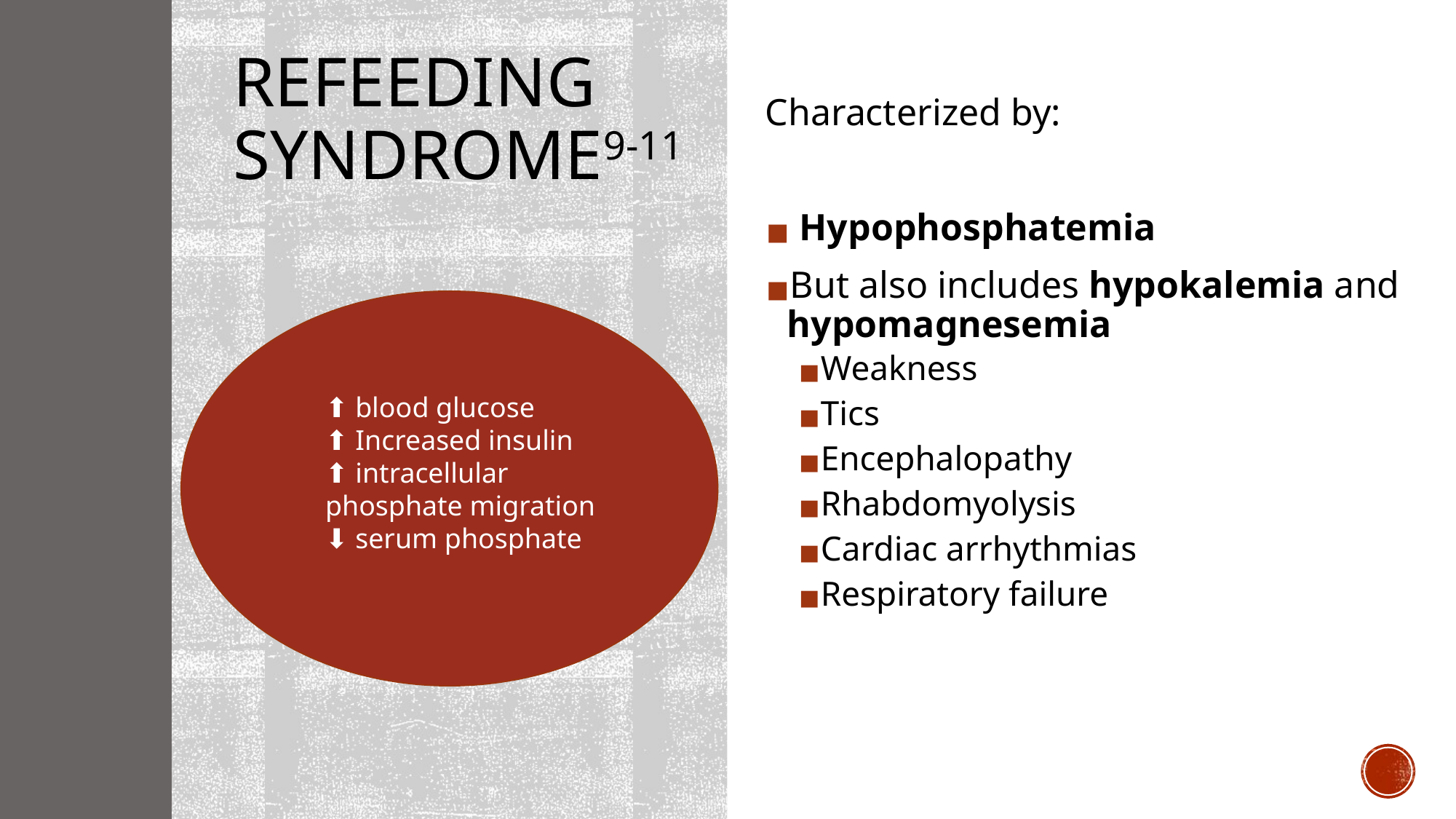

Characterized by:
 Hypophosphatemia
But also includes hypokalemia and hypomagnesemia
Weakness
Tics
Encephalopathy
Rhabdomyolysis
Cardiac arrhythmias
Respiratory failure
# REFEEDING SYNDROME9-11
⬆️ blood glucose
⬆️ Increased insulin
⬆️ intracellular phosphate migration
⬇️ serum phosphate

## Slide 38
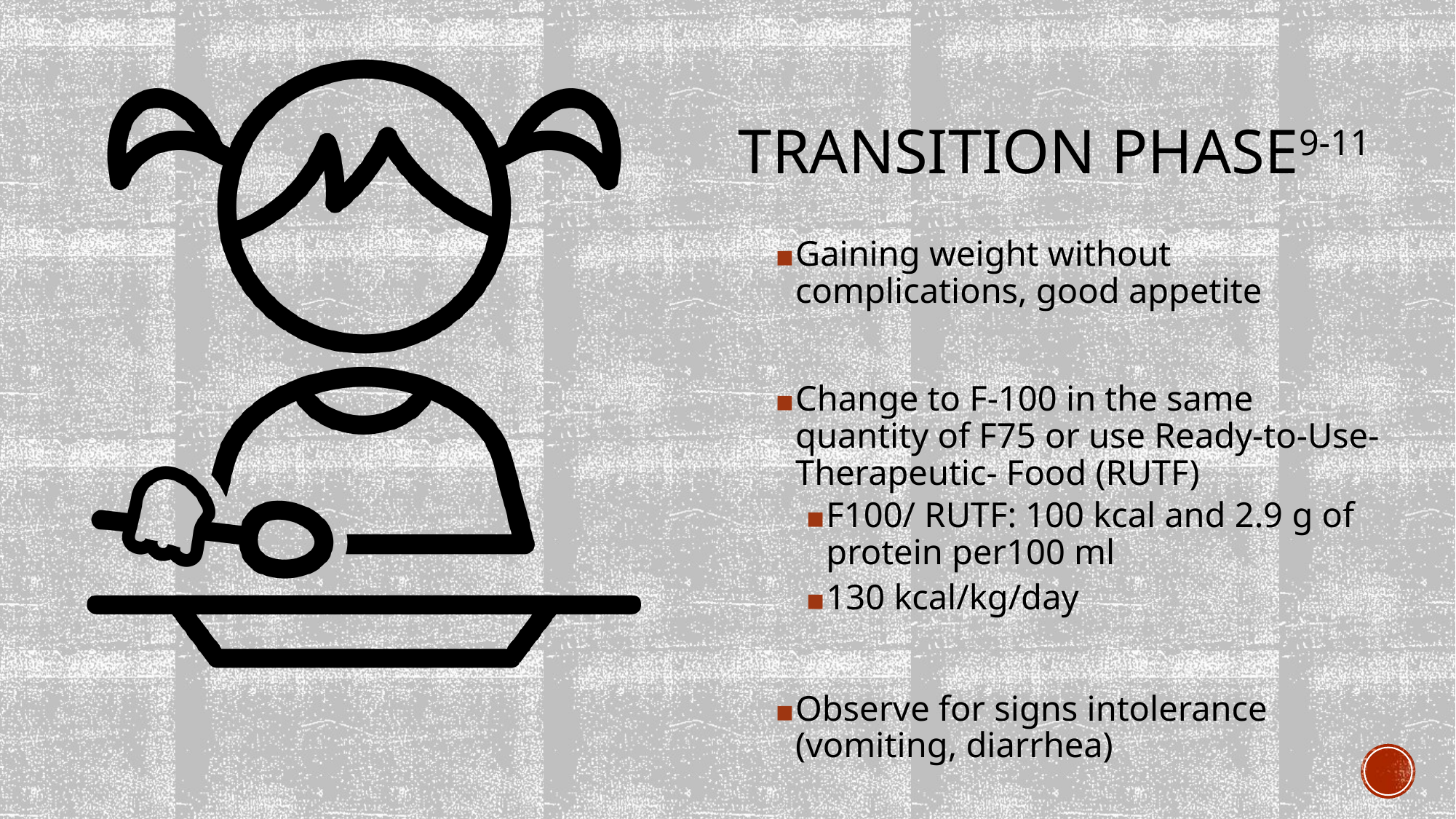

# TRANSITION PHASE9-11
Gaining weight without complications, good appetite
Change to F-100 in the same quantity of F75 or use Ready-to-Use-Therapeutic- Food (RUTF)
F100/ RUTF: 100 kcal and 2.9 g of protein per100 ml
130 kcal/kg/day
Observe for signs intolerance (vomiting, diarrhea)

## Slide 39
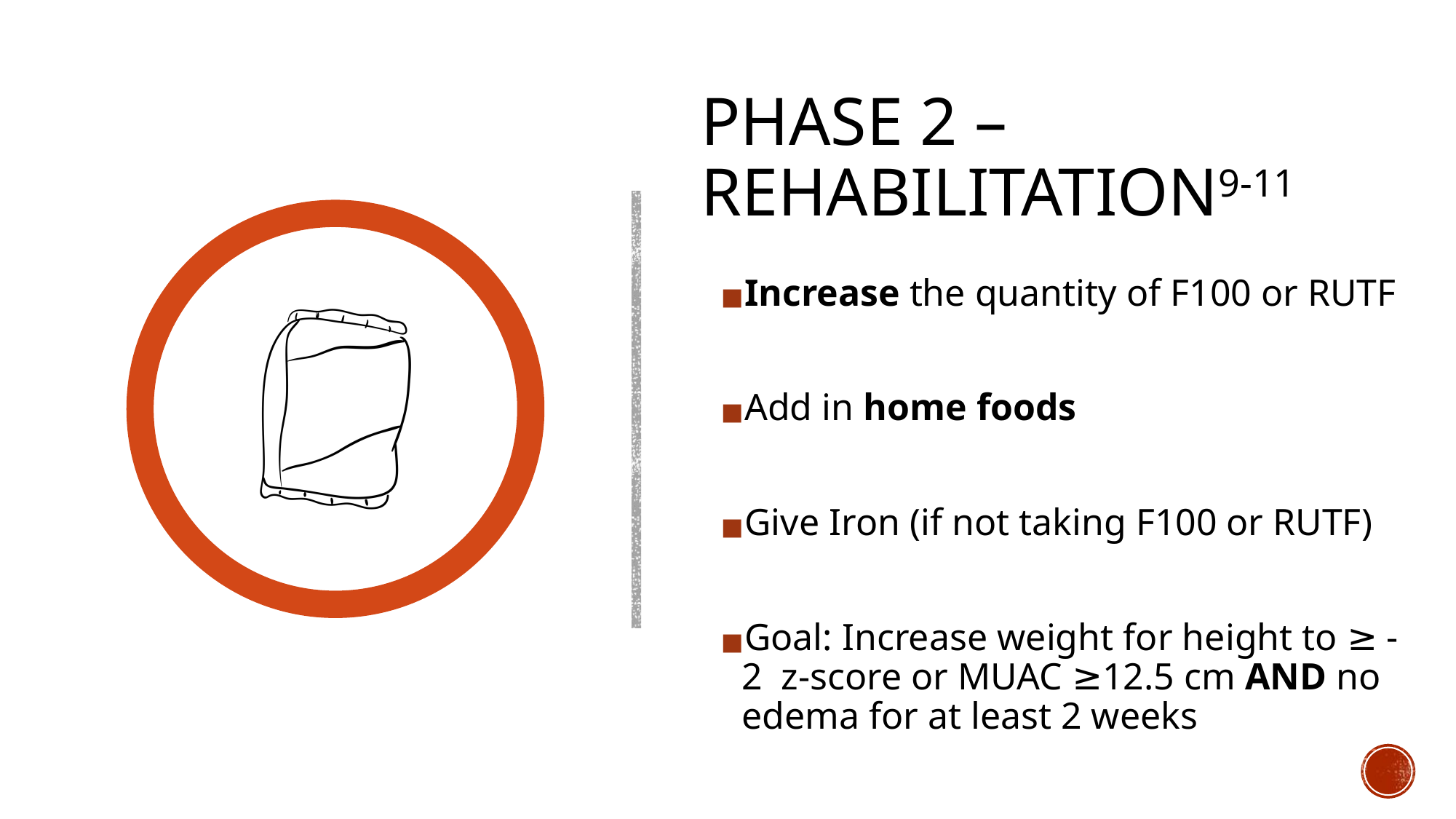

# PHASE 2 – REHABILITATION9-11
Increase the quantity of F100 or RUTF
Add in home foods
Give Iron (if not taking F100 or RUTF)
Goal: Increase weight for height to ≥ -2 z-score or MUAC ≥12.5 cm AND no edema for at least 2 weeks

## Slide 40
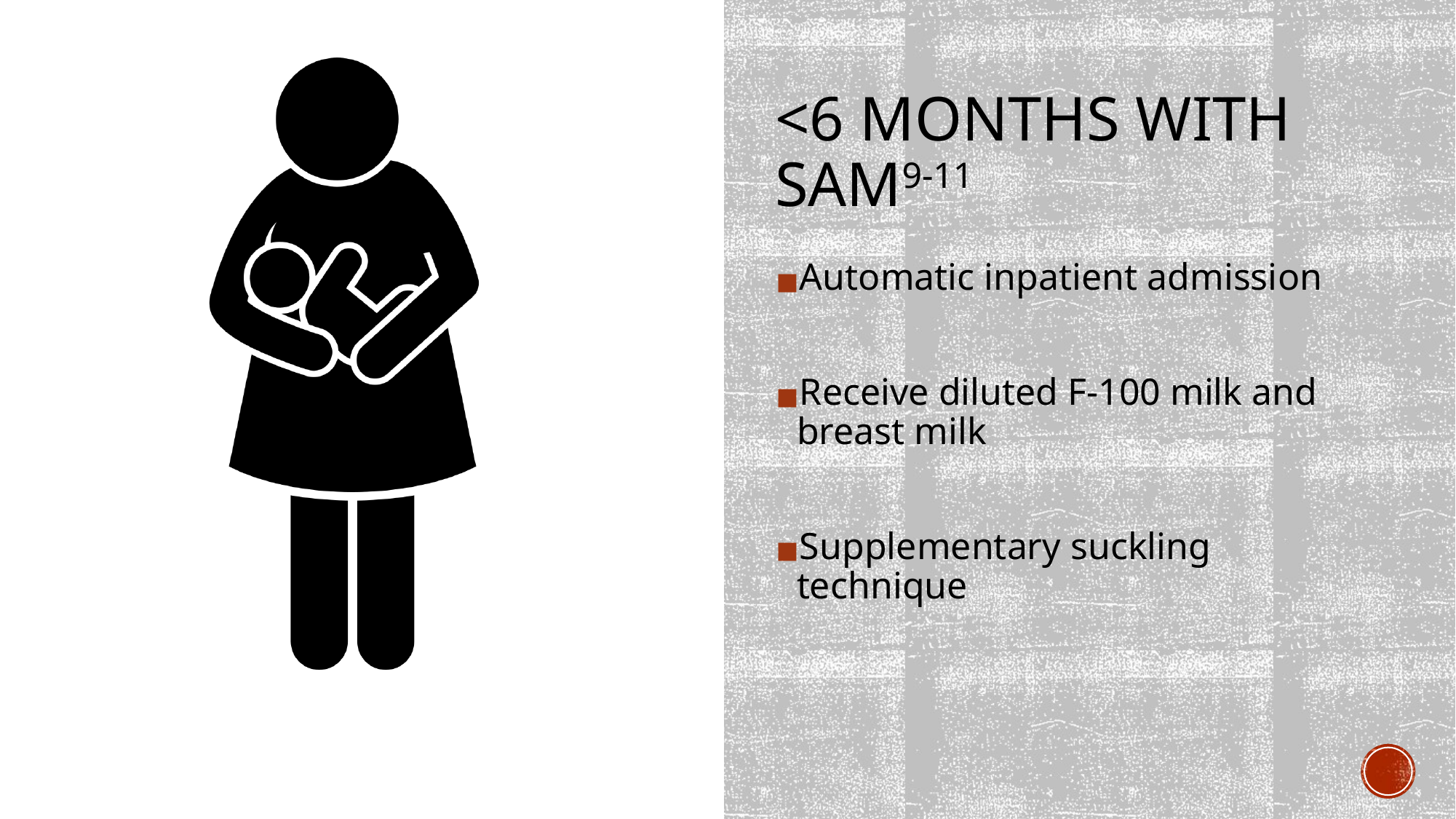

# <6 MONTHS WITH SAM9-11
Automatic inpatient admission
Receive diluted F-100 milk and breast milk
Supplementary suckling technique

## Slide 41
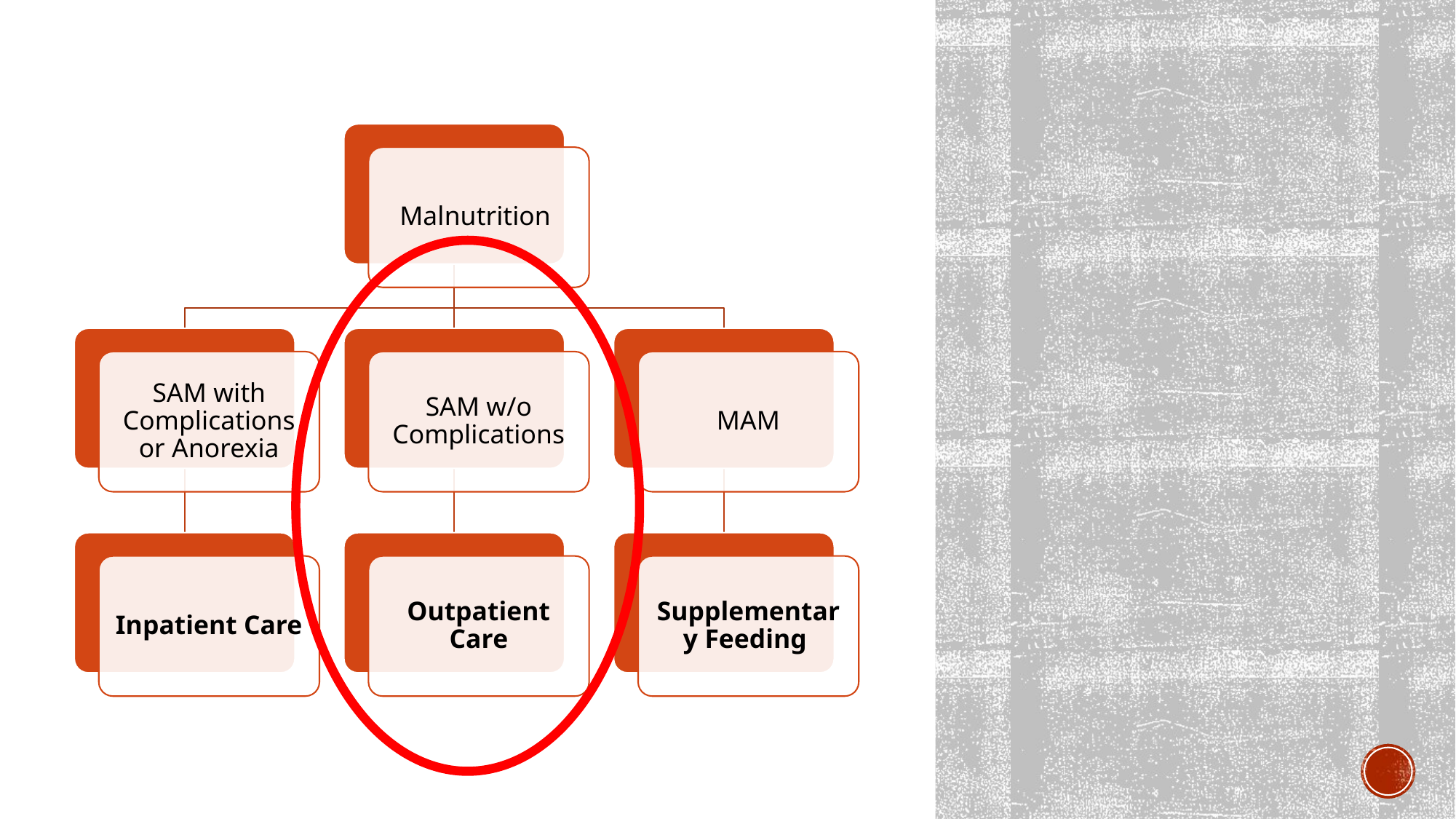

Malnutrition
SAM with Complications or Anorexia
SAM w/o Complications
MAM
Inpatient Care
Outpatient Care
Supplementary Feeding

## Slide 42
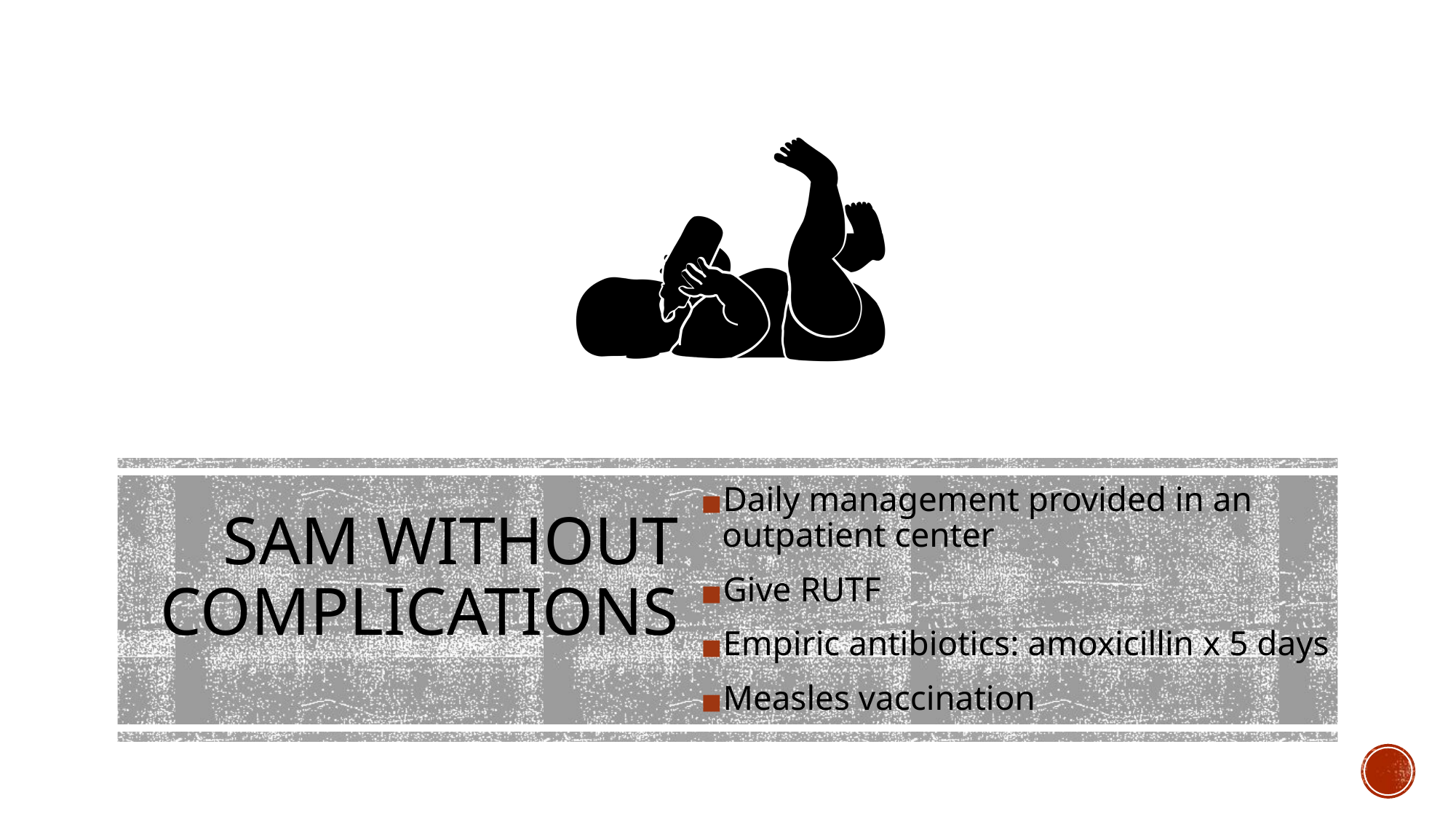

# SAM WITHOUT COMPLICATIONS
Daily management provided in an outpatient center
Give RUTF
Empiric antibiotics: amoxicillin x 5 days
Measles vaccination

## Slide 43
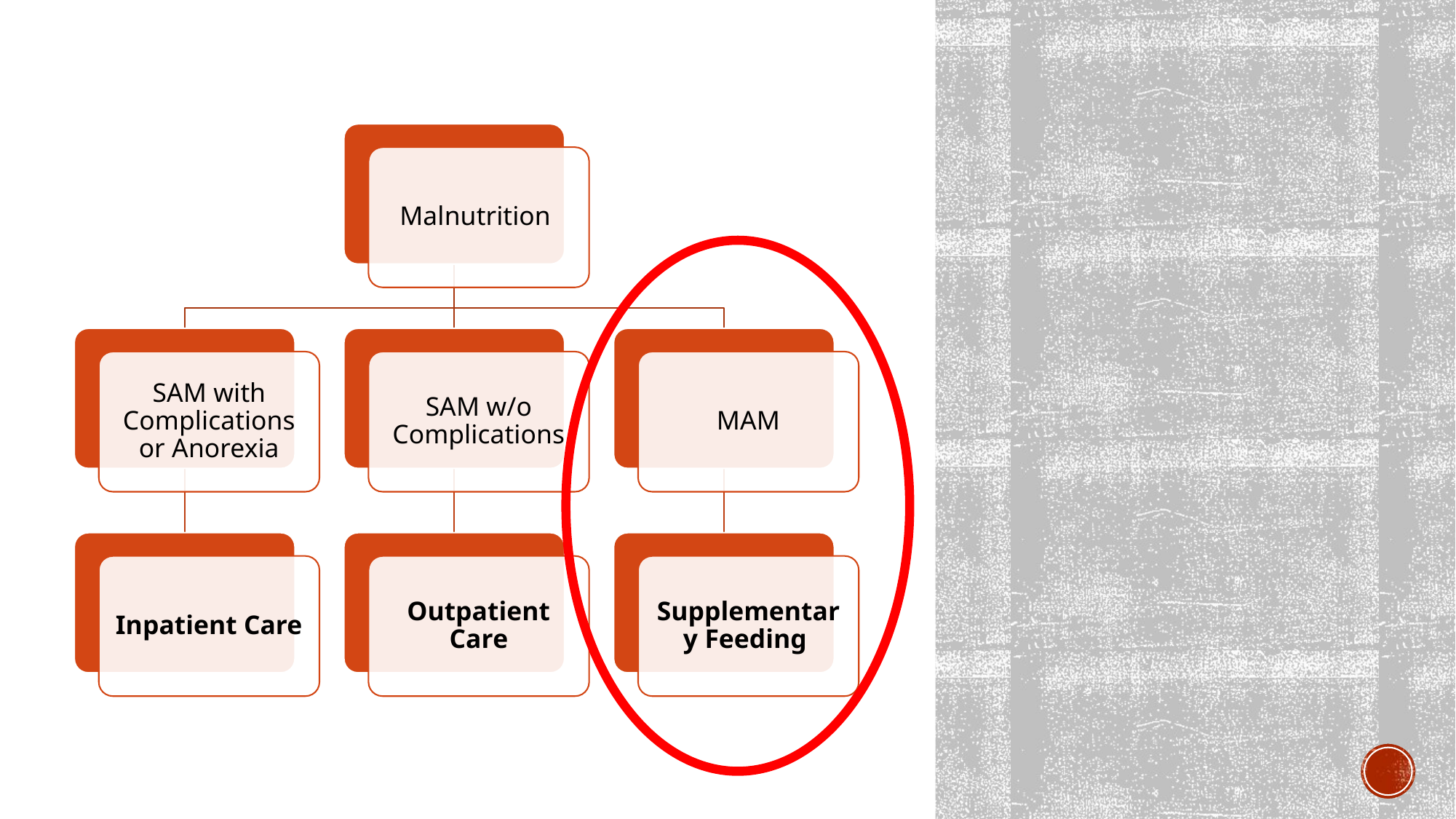

Malnutrition
SAM with Complications or Anorexia
SAM w/o Complications
MAM
Inpatient Care
Outpatient Care
Supplementary Feeding

## Slide 44
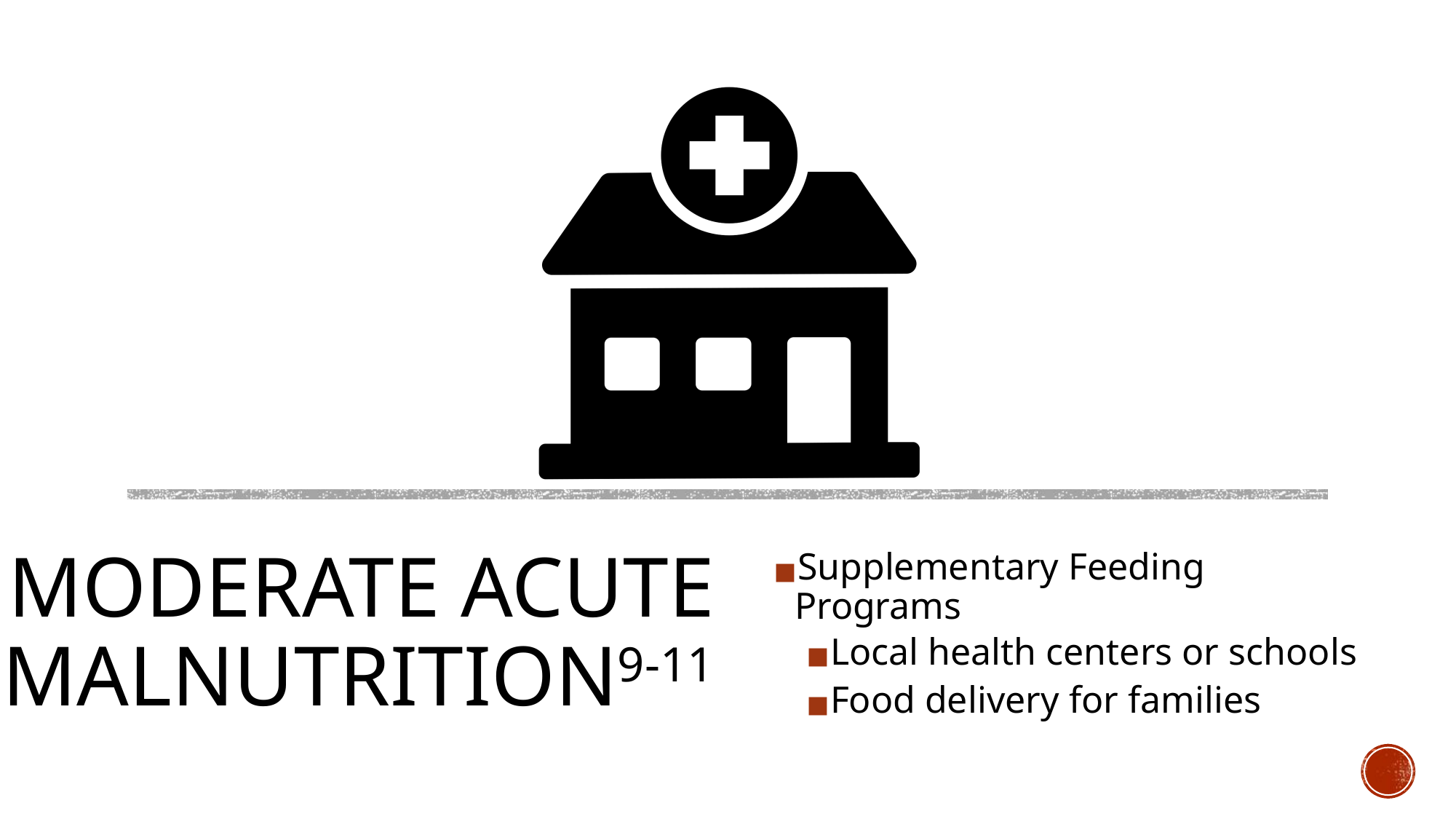

Supplementary Feeding Programs
Local health centers or schools
Food delivery for families
# MODERATE ACUTE MALNUTRITION9-11

## Slide 45
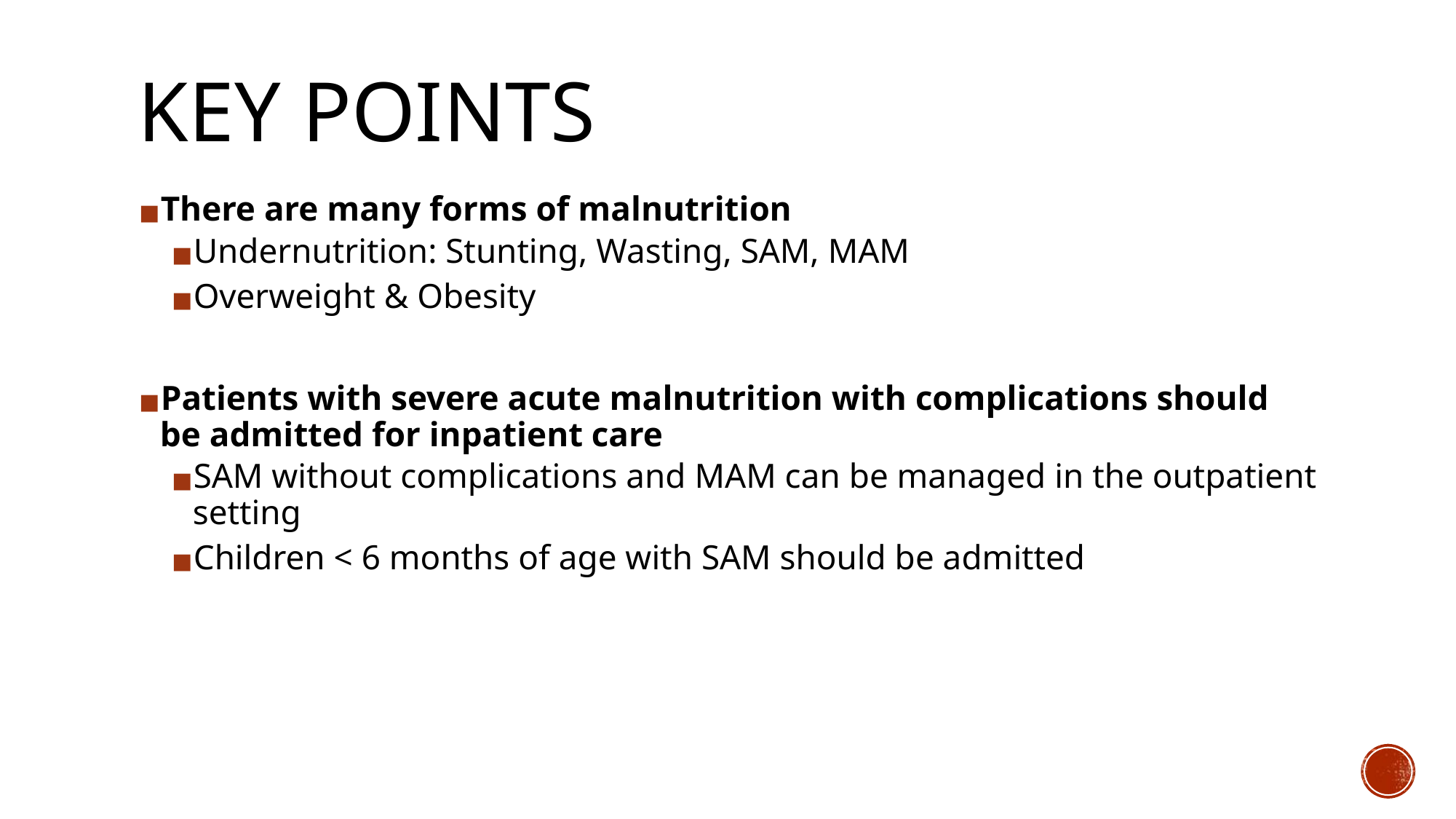

# KEY POINTS
There are many forms of malnutrition
Undernutrition: Stunting, Wasting, SAM, MAM
Overweight & Obesity
Patients with severe acute malnutrition with complications should be admitted for inpatient care
SAM without complications and MAM can be managed in the outpatient setting
Children < 6 months of age with SAM should be admitted

## Slide 46
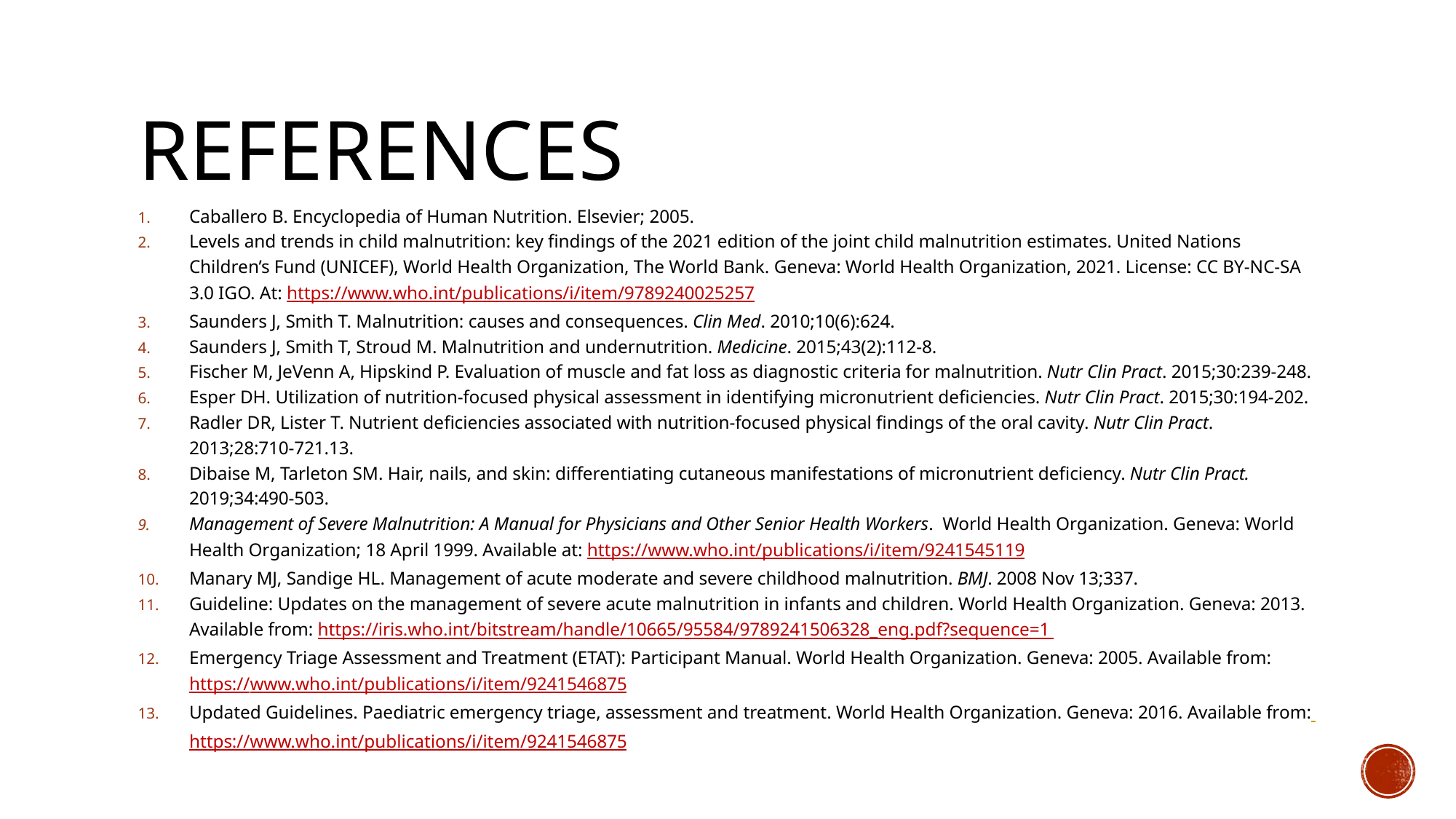

# REFERENCES
Caballero B. Encyclopedia of Human Nutrition. Elsevier; 2005.
Levels and trends in child malnutrition: key findings of the 2021 edition of the joint child malnutrition estimates. United Nations Children’s Fund (UNICEF), World Health Organization, The World Bank. Geneva: World Health Organization, 2021. License: CC BY-NC-SA 3.0 IGO. At: https://www.who.int/publications/i/item/9789240025257
Saunders J, Smith T. Malnutrition: causes and consequences. Clin Med. 2010;10(6):624.
Saunders J, Smith T, Stroud M. Malnutrition and undernutrition. Medicine. 2015;43(2):112-8.
Fischer M, JeVenn A, Hipskind P. Evaluation of muscle and fat loss as diagnostic criteria for malnutrition. Nutr Clin Pract. 2015;30:239-248.
Esper DH. Utilization of nutrition-focused physical assessment in identifying micronutrient deficiencies. Nutr Clin Pract. 2015;30:194-202.
Radler DR, Lister T. Nutrient deficiencies associated with nutrition-focused physical findings of the oral cavity. Nutr Clin Pract. 2013;28:710-721.13.
Dibaise M, Tarleton SM. Hair, nails, and skin: differentiating cutaneous manifestations of micronutrient deficiency. Nutr Clin Pract. 2019;34:490-503.
Management of Severe Malnutrition: A Manual for Physicians and Other Senior Health Workers. World Health Organization. Geneva: World Health Organization; 18 April 1999. Available at: https://www.who.int/publications/i/item/9241545119
Manary MJ, Sandige HL. Management of acute moderate and severe childhood malnutrition. BMJ. 2008 Nov 13;337.
Guideline: Updates on the management of severe acute malnutrition in infants and children. World Health Organization. Geneva: 2013. Available from: https://iris.who.int/bitstream/handle/10665/95584/9789241506328_eng.pdf?sequence=1
Emergency Triage Assessment and Treatment (ETAT): Participant Manual. World Health Organization. Geneva: 2005. Available from: https://www.who.int/publications/i/item/9241546875
Updated Guidelines. Paediatric emergency triage, assessment and treatment. World Health Organization. Geneva: 2016. Available from: https://www.who.int/publications/i/item/9241546875
